# Regulatory Mechanisms That Prevent Re-initiation of DNA Replication Can Be Locally Modulated at Origins by Nearby Sequence Elements

**DOI:** 10.1371/journal.pgen.1004358

**Published:** 2014-06-19

**Authors:** Christopher D. Richardson, Joachim J. Li

**Affiliations:** 1 Department of Biochemistry and Biophysics, University of California San Francisco, San Francisco, California, United States of America; 2 Department of Microbiology and Immunology, University of California San Francisco, San Francisco, California, United States of America; Brown University, United States of America

## Abstract

Eukaryotic cells must inhibit re-initiation of DNA replication at each of the thousands of origins in their genome because re-initiation can generate genomic alterations with extraordinary frequency. To minimize the probability of re-initiation from so many origins, cells use a battery of regulatory mechanisms that reduce the activity of replication initiation proteins. Given the global nature of these mechanisms, it has been presumed that all origins are inhibited identically. However, origins re-initiate with diverse efficiencies when these mechanisms are disabled, and this diversity cannot be explained by differences in the efficiency or timing of origin initiation during normal S phase replication. This observation raises the possibility of an additional layer of replication control that can differentially regulate re-initiation at distinct origins. We have identified novel genetic elements that are necessary for preferential re-initiation of two origins and sufficient to confer preferential re-initiation on heterologous origins when the control of re-initiation is partially deregulated. The elements do not enhance the S phase timing or efficiency of adjacent origins and thus are specifically acting as re-initiation promoters (RIPs). We have mapped the two RIPs to ∼60 bp AT rich sequences that act in a distance- and sequence-dependent manner. During the induction of re-replication, Mcm2-7 reassociates both with origins that preferentially re-initiate and origins that do not, suggesting that the RIP elements can overcome a block to re-initiation imposed after Mcm2-7 associates with origins. Our findings identify a local level of control in the block to re-initiation. This local control creates a complex genomic landscape of re-replication potential that is revealed when global mechanisms preventing re-replication are compromised. Hence, if re-replication does contribute to genomic alterations, as has been speculated for cancer cells, some regions of the genome may be more susceptible to these alterations than others.

## Author Summary

Eukaryotic organisms have hundreds to thousands of DNA replication origins distributed throughout their genomes. Faithful duplication of these genomes requires a multitude of global controls that ensure that every replication origin initiates at most once per cell cycle. Disruptions in these controls can result in re-initiation of origins and localized re-replication of the surrounding genome. Such re-replicated genomic segments are converted to stable chromosomal alterations with extraordinarily efficiency and could provide a potential source of genomic alterations associated with cancer cells. This publication establishes the existence of a local layer of replication control by identifying new genetic elements, termed re-initiation promoters (RIPs) that can locally override some of the global mechanisms preventing re-initiation. Origins adjacent to RIP elements are not as tightly controlled and thus more susceptible to re-initiation, especially when these global controls are compromised. We speculate that RIP elements contribute to genomic variability in origin control and make some regions of the genome more susceptible to re-replication induced genomic instability.

## Introduction

The initiation of eukaryotic DNA replication is tightly regulated so that it occurs at most once per cell cycle [1]. This regulation is critical because re-replication of a chromosomal segment makes that segment highly susceptible to genomic alterations [2]. Preventing re-replication throughout the genome is particularly challenging for eukaryotic cells because their genomes contain hundreds to thousands of replication origins. Hence, each individual origin must be tightly controlled if a genome is to avoid any re-initiation events [3].

The basic strategy eukaryotic cells use to prevent re-initiation is to prevent the reassembly of replication initiation complexes at origins that have fired. The critical assembly step that is regulated is the loading of the core replicative helicase Mcm2-7, which forms a toroidal complex that encircles the origin DNA [4]. This loading is carried out by four factors: the origin recognition complex (ORC), Cdc6, Cdt1, and Mcm2-7 [5], [6]. In the budding yeast, *Saccharomyces cerevisiae*, cyclin dependent kinases (CDKs) use multiple mechanisms targeting each of these proteins to prevent the reloading of Mcm2-7 once cells enter S phase [7]. In other organisms, additional CDK-independent mechanisms have been identified that inhibit Cdt1. The precise mechanisms used differ among species, but the reliance on multiple mechanisms targeting each of the initiation proteins involved in Mcm2-7 loading is highly conserved [3], [5].

The paradigm that has thus developed for the cell cycle control of replication initiation is that a multitude of overlapping mechanisms collaborate to globally inhibit initiation proteins throughout the cell, thereby minimizing the odds of re-initiating at any origin [8]. Consistent with this paradigm, disruption of individual mechanisms often does not lead to measurable re-replication even though the suspected consequences of re-replication, e.g. DNA damage or genomic alterations, have been observed [9]–[11]. Therefore, any investigation into the role of individual regulatory mechanisms in the block to re-initiation must be conducted in a sensitized system where a number of other overlapping mechanisms have been disrupted and re-replication can be readily detected.

Development of such sensitized systems revealed that origins re-initiate with diverse efficiencies and challenged the implicit assumption that all replication origins are uniformly regulated by global inhibition mechanisms [12], [13]. For example, when ORC, Cdc6, and Mcm2-7 are deregulated, many (∼100) origins detectably re-initiate, but many more (∼200) do not. Moreover, the amount of re-initiation from each origin varies widely. This diversity of re-initiation efficiency does not correlate with the diversity of S-phase origin timing and efficiency and thus cannot be explained by the chromosome context effects that are responsible for the latter [14]. Instead, the diversity in re-initiation efficiencies suggests that origins are not solely and uniformly regulated by global controls. Thus, we believe the paradigm for re-initiation control needs to be modified by the addition of a local layer of control that can modulate how tightly the global regulatory mechanisms inhibit re-initiation at specific origins.

Here, we explore the workings of this local control by asking why some budding yeast origins re-initiate more readily than others when global restrictions on re-initiation are partially inactivated. We show that local sequence elements adjacent to these origins specifically promote their re-initiation without enhancing their initiation activity. Furthermore, these elements act independently of the chromosomal context and silencing effects that regulate S-phase origin timing and efficiency. These elements, which we term re-initiation promoters (*RIPs*), map to ∼60 bp segments that work in a distance- and sequence- dependent manner. Analysis of the re-association of Mcm2-7 with origins suggests that these *RIP* elements antagonize an inhibitory mechanism that operates after Mcm2-7 association with origins. These findings provide our first insight into how diversity can be introduced in the regulation of eukaryotic replication origins.

## Results

### Several Origins Preferentially Re-initiate When Origin Controls Are Deregulated

To investigate the mechanisms underlying the diversity of origin regulation in the block to re-initiation, we examined *S. cerevisiae* origins whose ability to escape this regulation stood out the most from other origins. We previously reported that re-initiation occurs predominantly from *ARS317* in a strain where a subset of global replication controls was disrupted [12]. This “*MC2Ao*” strain was deregulated in three ways: (1) (*M*) - the CDK driven export of Mcm2-7 from the nucleus [15]–[17] was blocked by fusing a constitutive nuclear localization signal onto the endogenously expressed Mcm7; (2) (*C2A*) – the CDK inhibition of Cdc6, which occurs through transcriptional regulation [18], phosphorylation-directed degradation [8], [19], [20], and direct CDK binding [21], was completely disrupted by expressing an extra copy of Cdc6 lacking CDK phosphorylation and binding sites under a galactose-inducible promoter; and (3) (*o*) - the CDK inhibition of ORC by phosphorylation of Orc2 and Orc6 was minimally perturbed by eliminating one of four CDK consensus phosphorylation sites on Orc6 [2]. We note that this ORC deregulation was not necessary for the preferential re-initiation of *ARS317*, but enhanced it approximately 3-fold (Figure S1). Importantly, of the known mechanisms preventing re-initiation in budding yeast, two are retained in this strain: (1) CDK phosphorylation of Orc2 and Orc6 (9 out of 10 CDK consensus phosphorylation sites remain unmutated) [7]; and (2) Clb5-Cdc28 binding to an RXL docking site on Orc6 [22].

**Figure 1:**
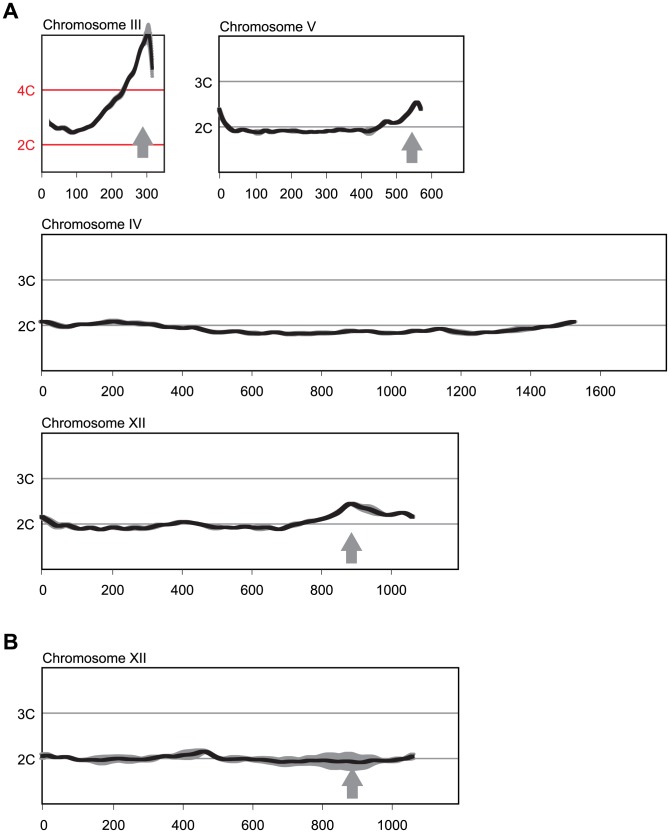
Multiple sites preferentially re-replicate when re-replication controls are deregulated in the *MC2Ao* strain background. (A) Re-replication profiles of Chromosomes III, V, and XII showing apparent sites of re-replication (gray arrows) in isogenic *MC2Ao* strains (YJL3758 and YJL3759) that were induced to re-replicate for 6 hr at an M phase arrest (nocodazole). For each strain, competitive genomic hybridization was performed against genomic DNA from the same strain arrested in M phase but before induction of re-replication (0 hr) (see Materials and Methods). DNA content from array CGH is plotted against chromosome position in kb. Chromosome IV shows the baseline ∼2C DNA content displayed by most of the genome. Data shown as mean of two profiles from the isogenic pair of strains (dark trace) ±SD (light trace). (B) Re-replication peak on Chromosome XII is dependent on *ARS1238*. Re-replication profile of Chromosome XII from YJL9152 was generated as described in A. YJL9152 is congenic to YJL3758/YJL3759 except for deletion of *ARS317* and *ARS1238* (arrow). Data shown as mean of duplicate profiles from YJL9152 (dark trace) ±SD (light trace).

Re-initiation was not detectable in the *MC2Ao* strain until the deregulated Cdc6 was induced. We could thus arrest cells at metaphase with a normal 2C DNA content across the genome, induce the deregulated Cdc6, and detect re-initiation and re-replication as a >2C DNA copy number using array comparative genomic hybridization (aCGH). Although the primary re-initiation event after a 3 hr induction of re-replication was at *ARS317* [12], the re-replication profiles showed hints of additional re-replication peaks at other genomic loci. At least two of these peaks were readily confirmed with a longer 6 hr induction of re-replication, one on the right arm of Chr 5 near position 575 kb, and one on the right arm of chromosome 12 near position 890 kb (Figure 1A). The latter was dependent on *ARS1238*, establishing that this origin also preferentially re-initiated in the *MC2Ao* strain (Figure 1B). Because *ARS317* and *ARS1238* were among the two most efficient re-initiating origins, we focused on them to investigate why some origins are more susceptible to re-initiation than others.

### Preferential Re-initiation of *ARS317* and *ARS1238* Is Conferred by Local Sequence Determinants

We first sought to determine whether the preferential re-initiation of *ARS317* and *ARS1238* was conferred by the origin and immediate surrounding sequences or required a broader chromosomal context that spans kilobases of DNA. An example of the latter is the poorly understood chromosome position effect that has been implicated in the diversity of yeast origin timing and efficiency during normal S phase initiation (discussed in [14], [23]). We and others had previously shown that there was no correlation between this diversity of origin activity in S phase and the diversity of re-initiation efficiency displayed in strains where many origins re-initiate due to complete deregulation of ORC, Mcm2-7, and Cdc6 [12], [13]. Nonetheless, a different chromosomal context could be conferring preferential re-initiation on *ARS317* or *ARS1238* in the *MC2Ao* strain.

**Figure 2:**
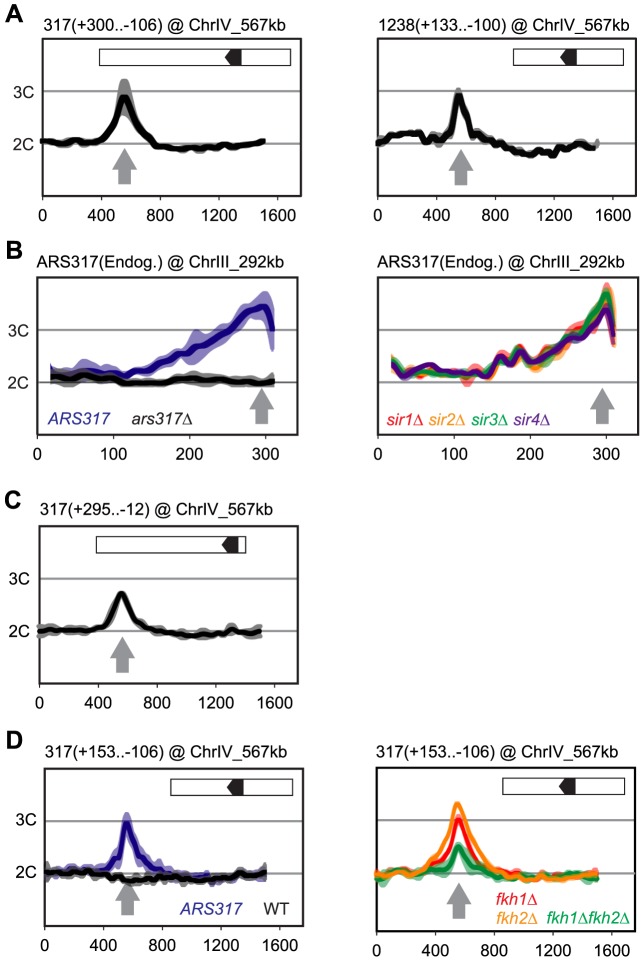
Local determinants and not chromatin context confer preferential re-initiation on *ARS317* and *ARS1238*. Re-replication profiles for *ARS317* at its endogenous location on Chromosome III (ChrIII_292 kb) or at a transplanted location on Chromosome IV (ChrIV_567 kb). Profiles were obtained by array CGH of genomic DNA from MC2Ao strains induced to re-replicate for 3 hr at an M phase (nocodazole) arrest against genomic DNA from an M phase arrested strain (YJL7695; see Materials and Methods). The same protocol was used for *ARS1238* transplanted to ChrIV_567 kb except re-replication was induced for 6 hr. Boundaries of the transplanted fragments are listed in parentheses above their panels using nucleotide positions relative to the T-rich strand of the ORC binding sites (OBS) (+1 to +33 in the 5′ to 3′ direction). Inset shows schematic of transplanted fragments: black arrowhead – OBS pointing in 5′ to 3′ direction of T-rich strand; white – additional sequences adjacent to *ARS317* or *ARS1238* at their endogenous loci that are transplanted along with the origins. (A) DNA fragments containing *ARS317* and *ARS1238* confer preferential re-initiation when transplanted to an ectopic locus. Left panel: *MC2Ao* strains with a 406 bp *ARS317*-containing fragment (YJL7700 and YJL7701) integrated at ChrIV_567 kb (gray arrow). Right panel: *MC2Ao* strains with a 233 bp *ARS1238*-containing fragment (YJL9566 and YJL9567). Data shown as mean of two profiles from the isogenic pair of strains (dark trace) ±SD (light trace). (B) Preferential re-initiation of *ARS317* is independent of the transcriptional silencing genes *SIR1-4*. Re-replication profiles of *ARS317* at endogenous locus on Chromosome III (ChrIII_292 kb, gray arrow). Left panel: *MC2Ao* strains that are wild-type for the *SIR* genes and contain either *ARS317* (YJL3758; n = 5) or *ars317*Δ (YJL8398; n = 10) at the endogenous locus. Data shown is mean of the indicated number of profiles for each strain (dark trace) ±SD (light trace). Right panel: strains congenic to YJL3758 but containing *sir1*Δ (YJL6893 and YJL6894, red), *sir2*Δ (YJL6896 and YJL6897, yellow), *sir3*Δ (YJL6899 and YJL6900, green), or *sir4*Δ (YJL6902 and YJL6903, blue). Data shown as mean of two profiles from each isogenic pair of strains (dark trace) ±SD (light trace). (C) Preferential re-initiation of *ARS317* is independent of the transcriptional silencer element *HMR-E*. A 307 bp fragment that contains the *ARS317 OBS* but lacks the other two essential subelements of *HMR-E* (Rap1 and Abf1 binding sites) was integrated at ChrIV_567 kb (gray arrow) in *MC2Ao* strains YJL8256 and YJL8257 (which are congenic to YJL7700 and YJL7701). Data shown as mean of two profiles from the isogenic pair of strains (dark trace) ±SD (light trace). (D) Forkhead proteins are not essential for preferential re-initiation of *ARS317*. A 259 bp fragment that is sufficient to confer preferential re-initiation of *ARS317* was integrated at ChrIV_567 kb (gray arrow) in *MC2Ao* strains. Left panel: *FKH* strains with (*ARS317*, YJL8398, n = 10) or without (*WT*, YJL3758, n = 5) *ARS317* transplanted to ChrIV_567 kb. Data shown is mean of the indicated number of profiles for each strain (dark trace) ±SD (light trace). Right panel: strains congenic to YJL8398 but containing *fkh1*Δ (YJL8745 and YJL8746, red*), fkh2*Δ (YJL8701 and YJL8702, yellow), or *fkh1*Δ *fkh2*Δ (YJL8749 and YJL8750, green). Data shown as mean of two profiles from each isogenic pair of strains (dark trace) ±SD (light trace).

To distinguish between local sequence determinants and a broader chromosomal context, we investigated whether small fragments containing the *ARS317* or *ARS1238* origins could preferentially re-initiate when transplanted to ectopic genomic loci. We focused initially on fragments that we hoped would be small enough to dissect at the nucleotide level but large enough to encompass the origin and any possible additional sequences that might be needed for preferential re-initiation. A 537 bp fragment previously shown to contain *ARS317* [24] preferentially re-initiated when transplanted from its endogenous location to sites on other chromosomes (ChrIV_567 kb, ChrIV_1089 kb) [2], [12]. In all cases, the amount of re-initiation induced after 3 hr (2.7–3.0 C) at the ectopic locus was comparable to the amount of re-initiation at the endogenous locus (2.8–3.2 C) [2], [12]. Hence, neither the chromatin context nor the replication timing (early or late in S-phase) of the transplant location were key determinants of the re-replication activity on these origins. Consistent with this notion, Figure 2A shows that an even smaller 406 bp fragment containing *ARS317* preferentially re-initiates when transplanted to position ChrIV_567 kb. At this same location, a 233 bp *ARS1238* fragment that contains the ORC binding site (OBS) and 100 bp of flanking sequence on either side [25] also re-initiates (Figure 2A). Thus, the preferential re-initiation of *ARS317* and *ARS1238* is conferred by local sequence determinants and is independent of a broader chromosomal context.

### Preferential Re-initiation at *ARS317* and *ARS1238* Does Not Require Silencing Proteins or Forkhead Transcription Factors

*ARS317* is a core element of a 138 bp transcriptional silencer *HMR-E*, one of several silencers that recruit the silencing proteins Sir1-4 to organize the surrounding DNA into a heterochromatin-like structure (reviewed in [26]). The entire *HMR-E* silencer is included within the transplanted *ARS317*-containing fragments described above, so the preferential re-initiation of this fragment could be associated with its organization into heterochromatin [27], [28]. Such a connection is reminiscent of reports that heterochromatin preferentially re-replicates in budding yeast and Drosophila [13], [29]. To test this possibility, we individually deleted each of the four *SIR* genes and analyzed the re-replication profiles around *ARS317* for each *sir* mutant. These profiles resembled those from the wild-type *SIR* control strains (Figure 2B and Figure S2A), indicating that none of the Sir proteins are required for the preferential re-initiation of *ARS317*. We also observed re-replication in a truncated *ARS317* clone lacking the RapI and AbfI binding sites that are critical for *HMR-E* silencer function [28] (Figure 2C). We conclude that a silent chromatin state is not necessary for the preferential re-initiation of *ARS317*. *ARS1238* is not assembled into heterochromatin, so one would expect its preferential re-initiation to be independent of Sir proteins. Our data are consistent with this expectation (Figure S2C), although the profiles are not as clear-cut.

Other factors known to influence nearby origin function are the forkhead transcription factors Fkh1 and Fkh2. Association of these proteins with origins and ORC has been implicated in the spatial organization of origins in the nucleus. This organization is thought to alter the S phase replication timing of some origins, including *ARS1238* [30]. Although Fkh proteins do not influence *ARS317* replication timing, searches for their proposed binding motifs have identified predicted binding sites within a few kilobases of both *ARS317* and *ARS1238* [31], [32]. To test whether Fkh1 or Fkh2 are critical for re-initiation of either origin, we examined the re-replication profiles in *fkh1*Δ, *fkh2*Δ, and *fkh1*Δ*fkh2*Δ strains. At both *ARS317* (Figure 2D and Figure S2B) and *ARS1238* (Figure S2D), *fkh*Δ strains re-replicated significantly more than negative control strains that lack re-replicating origins at these loci. These results confirm that the forkhead proteins are not essential for the preferential re-initiation of either origin. We did observe a partial reduction of re-replication in the *fkh1*Δ*fkh2*Δ background, so we cannot rule out a role for these proteins in supporting re-initiation. However, we suspect that this reduced re-replication may be an indirect consequence of the severe growth defect and cell clumping exhibited by the double mutant during growth in liquid media [30].

### A Distinct Element Confers Preferential Re-initiation on *ARS317* and *ARS1238*

The preferential re-initiation activity seen in transplanted fragments containing *ARS317* and *ARS1238* could be intrinsic to the origin sequences themselves, or be conferred on these origins by neighboring sequences that are dispensable for initiation activity. The former possibility is particularly relevant for *ARS317*, whose especially tight interaction with ORC appears to govern the activity of this origin in S phase [33]. If this possibility is correct, any minimal segment containing origin activity should also exhibit preferential re-initiation. In contrast, if the latter possibility is correct, the fragments should be separable into an origin segment that can initiate but not preferentially re-initiate, and an adjacent segment that can neither initiate nor preferentially re-initiate on its own but confers preferential re-initiation on the origin segment. To test this separability, of functions for both *ARS317* and *ARS1238* we generated subclones of the transplanted fragments described in Figure 2A and assayed them for both initiation and re-initiation activity.

**Figure 3:**
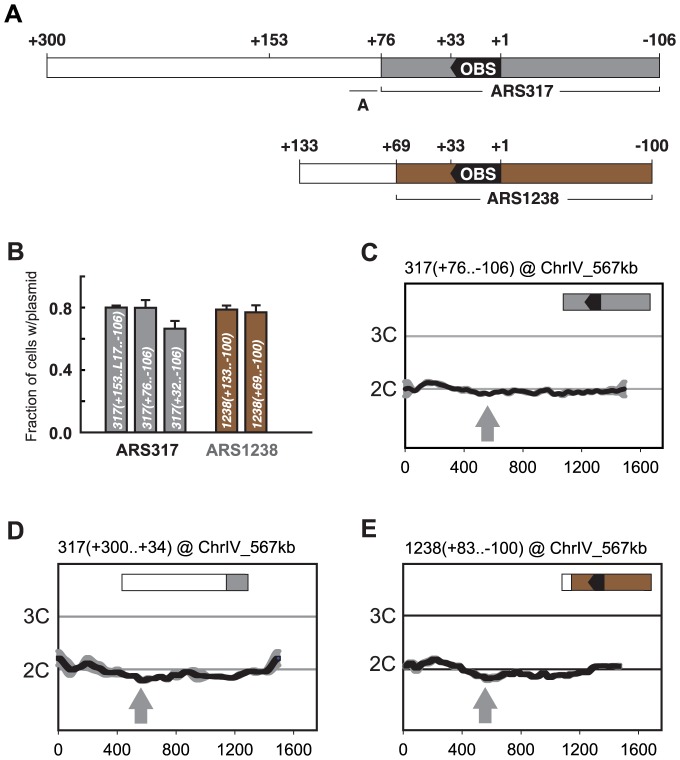
Preferential re-initiation of *ARS317* and *ARS1238* requires additional sequences flanking the origins. (A) Schematic of origin segments contained within preferentially re-initiating fragments. Nucleotide positions are defined relative to the *OBS* for each origin (+1 to +33 on T-rich strand). In the upper schematic, gray indicates sequences (+76…−106) sufficient for optimal *ARS317* ARS activity (see B). The full segment (+300…−106) corresponds to the fragment responsible for preferential re-initiation in Figure 2A, left panel. +153 indicates the left boundary of the minimal sequence required for preferential re-initiation of *ARS317* (see Figure 4A). A indicates the location (+95 to +77) of previously mapped near matches to the ARS consensus sequence that confers cryptic origin activity independent of *ARS317* [36]. In the lower schematic, brown indicates sequences (+69…−100) sufficient for optimal *ARS1238* ARS activity (see B). The full segment (+133…−100) corresponds to the fragment responsible for preferential re-initiation in Figure 2A, right panel. (B) Identification of sequences sufficient for origin activity in preferentially re-initiating fragments. Plasmids containing indicated cloned DNA segments were assayed in wild-type strain YJL310 by measuring their mitotic stability, i.e. fraction of cells growing under selection for the plasmid that contain the plasmid. Plasmids and DNA segments assayed were pCR133, *317(+153..L17..−106)­*; pCR339, *317(+76..−106)*; pCR287, *317(+32..−106)*, pCR221, *1238(+133..−100)*; and pCR321, *1238(+69..−100)*; where the numbers in parentheses indicate nucleotide boundaries of the segment. L17 is an 8 bp linker substitution mutation of nt +86 to +79 (see Figure 4B), which disrupts the cryptic origin activity mentioned in A and allows *ARS317* origin activity to be assayed on its own. pCR287 contains the *HMR-E* silencer fragment that originally identified *ARS317* [28], but this fragment contains suboptimal origin activity. Mitotic stabilities presented as mean ±SD, n = 3. (C) *ARS317* origin cannot preferentially re-initiate by itself. The 182 bp *ARS317* origin segment shown in A and B, *317(+76..−106)*, was integrated at ChrIV_567 kb (gray arrow) in strains YJL10444 and YJL10445. Re-replication profiles were generated and displayed as in Figure 2. (D) The sequence flanking the *ARS317* origin cannot preferentially re-initiate by itself. A 267 bp segment (nt +300 to +34) containing the 224 bp segment flanking the *ARS317* origin mapped in B (nt +300 to +77) was integrated at ChrIV_567 kb (gray arrow) in YJL7717. Re-replication profiles were generated and displayed as in Figure 2 for *ARS317*, except the mean of two profiles, both from YJL7717, is shown. (E) *ARS1238* origin cannot preferentially re-initiate by itself. A 183 bp segment (nucleotides +83 to −100), containing the 169 bp *ARS1238* origin segment assayed in B, *1238(+69..−106)*, was integrated at ChrIV_567 kb (gray arrow) in strains YJL9707 and YJL9708. Re-replication profiles were generated and displayed as in Figure 2 for *ARS1238* in the congenic strains YJL9566 and YJL9567.

Initiation activity requires a 33 bp consensus ORC binding site (*OBS*) and less well-defined flanking sequences [34], [35]. The *OBS* is comprised of a 17 bp extended *ARS* consensus sequence (e*ACS*), formerly known as the A domain, and a WTW sequence [36] formerly known as the B1 subdomain. The required flanking sequences usually lie 3′ of the T-rich strand of the *OBS*, where they comprise the rest of the B domain (B2 and B3), but occasionally can lie 5′ of the *OBS*, where they are referred to as C domain sequences [37]. We numbered nucleotides in our subclones relative to the *OBS* [35], assigning +1 and +33 to the first and last nucleotide, respectively, of the T-rich strand of the *OBS*. In this scheme, B domain sequences outside the *OBS* are numbered +34 and higher, and C domain sequences have negative coordinates (Figure 3A). The 406 bp preferentially re-initiating fragment containing *ARS317* is thus designated *317(+300..-106)*, and the equivalent 233 bp fragment for *ARS1238* is designated *1238(+133..-100)*.

The initiation activity of an origin can be assayed by the ability of a plasmid containing the origin to be maintained in cells. One measure of this ability is the mitotic stability assay, which measures the steady state percentage of cells containing the plasmid in a culture grown under selection for the plasmid [38], [39]. The mitotic stability of several subfragments containing *ARS317* showed that full origin activity was retained by *317(+76..−106)* (Figure 3B). This origin segment failed to re-initiate when inserted at ChrIV_567 kb (Figure 3C), demonstrating that *ARS317* does not have an intrinsic ability to re-initiate. The adjacent segment *317(+300..+77)* was also not able to re-initiate when examined in the context of a slightly larger fragment *317(+300..+34)* at ChrIV_567 kb (Figure 3D). This adjacent segment does contain sequences that are essential for a weak cryptic origin (Figure 3A) [36], but a mutation that disrupts this cryptic origin did not reduce the ability of these adjacent sequences to induce re-initiation (Figure S3; mutant A). In contrast, a mutation of the *ARS* consensus sequence in the *ARS317 OBS* did eliminate re-initiation, confirming that the re-initiation is dependent on *ARS317* ([12], also Figure S3 mutant E). These data show that the preferentially re-initiating fragment *317(+300..−106)* can be separated into an *ARS317* origin segment *317(+76..−106)* and an adjacent segment *317(+300..+77)* that confers preferential re-initiation on *ARS317* in the *MC2Ao* strain. We call the sequence element that confers this activity a re-initiation promoter (*RIP*) and will refer to it as *RIP317*.

We used a similar approach to identify a subsegment of *1238(+69..−100)* that retains full *ARS1238* origin activity (Figure 3B) but is not sufficient to preferentially re-initiate. This inability to re-initiate was demonstrated in the context of a slightly larger segment *1238(+83..−100)* at ChrIV_567 kb (Figure 3E). Further evidence that neither origin segment nor adjacent segment have re-initiation activity on their own comes from insertion mutations (discussed later) that separate the two segments by 153 bp, and abolish re-initiation. In addition, the adjacent segment *1238(+133..+70)* does not contain the origin activity needed to support maintenance of an autonomous plasmid. Thus, like *ARS317*, *ARS1238* acquires its ability to preferentially re-initiate from an adjacent re-initiation promoter, which we will refer to as *RIP1238*.

### Mapping the Re-initiation Promoters

**Figure 4:**
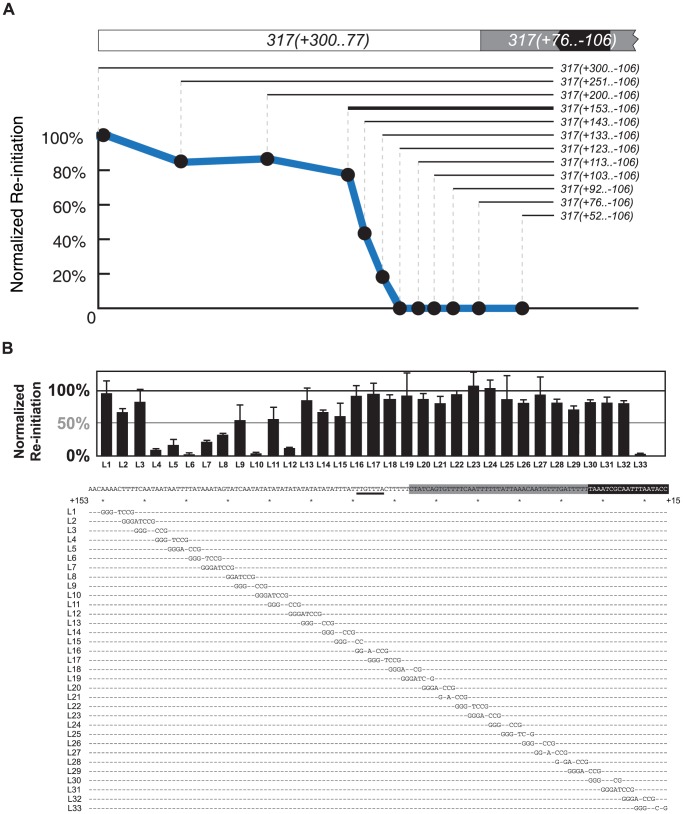
Mapping the re-initiation promoter for *ARS317*. (A) Identifying the left boundary of the Re-Initiation Promoter for *ARS317*. A nested series of left-side deletions (lines with nucleotide coordinates) of the *ARS317* re-initiating fragment *317(+300..−106)* were individually inserted at ChrIV_567 kb. Bold line represents deletion segment used for linker scan analysis in B. Mean re-replication profiles were obtained as described in Figure 2, except each mean profile was calculated from duplicate experiments of the same strain. Re-initiation efficiency was calculated by normalizing the mean peak height for each deletion fragment against the mean peak height for the full length *317(+300..−106)* fragment in the congenic reference strain YJL7700. The strains used for each deletion are listed in Table S2. (B) Structure of Re-Initiation Promoter for *ARS317*. An overlapping series of linker substitution mutations (L1–L33) constructed with an 8 bp GGGATCCG linker in the preferentially re-initiating segment *317(+153..−106)* were assayed for re-initiation efficiency as described in A, except efficiencies were normalized against the congenic reference strain YJL8398, which contains the wild-type *317(+153..−106)* sequence (partially shown below graph). Sequences of linker mutations are represented by letters for changed nucleotides and dashes for unchanged nucleotides. Position of the linker mutant HMRE-A (See Figure S3) used to disrupt cryptic origin activity is indicated by a thick black line at position +89..+83. The strains tested in duplicate for each linker substitution are listed in Table S2.

In order to map *RIP317* with finer resolution, we first analyzed the re-initiation efficiency of a nested series of deletions extending into the left border (plus side) of the 406 bp *317(+300..−106)* fragment. These deletion constructs were introduced into ChrIV_567 kb, and their re-initiation efficiency measured by normalizing the amount of re-initiation for each deletion (i.e. the copy number increase above 2C) against the amount of re-initiation for the full-length fragment. Deletions up to nucleotide +153 had limited effect on re-initiation efficiency, but further deletion into the fragment caused a precipitous drop (Figure 4A). Thus, nucleotide +153 in the 259 bp deletion fragment *317(+153..−106)* defines a left-hand boundary for *RIP317*.

To further map *RIP317* we used *317(+153..−106)* as the parent sequence for a linker scan analysis of *RIP317* structure (Figure 4A; bold line). Most of the linker mutations that showed a noticeable reduction in *ARS317* re-initiation efficiency were from L4 to L15, which covers the 51 bp from nucleotide +137 to +87 (Figure 4B). On the left end of this 51 bp region were linker mutations (L4–L7), which drastically reduced or eliminated *ARS317* re-initiation and identified sequences that are critical for *RIP* function. Other linker mutations (L8–L15) showed less striking reductions in re-initiation individually (Figure 4B), but eliminated re-initiation when combined together (Figure S4A). Thus, the sequences mutated by linkers L8–L15 are also important for *RIP* function but may contain partially redundant sequence elements.

In contrast to linker mutations L4–L15, the remaining linker mutations from L16–L32 each had limited effects on *ARS317* re-initiation (Figure 4B). We note that *ARS317* differs from most yeast origins in that the WTW sequence of its *OBS* is dispensable for initiation activity [36], [40]. Linker L29, which mutates the WTW sequence, and linkers L30 and L31, which intrude further into the OBS, still leave intact the 17 bp extended ARS consensus sequence (*eACS*), which forms the core of the ORC binding site [41]. Thus, although these linkers mutate parts of the *OBS*, they presumably do not disrupt *ARS317* re-initiation efficiency because they leave *ARS317* origin activity intact. Linker L33, on other hand, does mutate part of the *eACS*, so its partial disruption of *ARS317* re-initiation is likely due to impairment of origin function. Replacement of the entire sequence covered by L17–L31 (nucleotides +86 to +23) with sequence of similar AT content did not have much effect on *ARS317* re-initiation (Figure S4B). Additional replacement of sequences covered by L1–L3 decreased re-initiation efficiency by a third, indicating that these sequences contribute to optimal *RIP317* activity (Figure S4B). These results suggest that *RIP317* resides in the 67 bp from nucleotides +153 to +87 and contains a core region of approximately 19 bp (+137 to +119) that is crucial for its function.

As discussed above, we had narrowed down *RIP1238* to a 64 bp segment from nucleotide +133 to +70. Linker scan analysis revealed that linker mutations spanning 40 bp (+117 to +78) of this segment abolished *ARS1238* re-initiation, while the remaining mutations showed a more modest reduction (Figure S4C). Thus, like *RIP317*, *RIP1238* has a core segment that is crucial for RIP function and surrounding sequences that enhance this function.

### *RIP* Function Is Not Simply Dependent on High AT Content

The most obvious common feature of *RIP317* and *RIP1238* is the high AT-content of these sequences (92% and 84% AT respectively). Regions of high AT-content have been postulated to exclude nucleosomes (Reviewed in [42]) or to provide regions of reduced helical stability that facilitate DNA unwinding during replication initiation [43]. Therefore, we wondered if *RIP* elements were stimulating re-initiation through such a positioning or thermodynamic mechanism.

To test this possibility we generated various mutants that preserved the AT content of *RIP317* while altering its sequence identity. Neither predicted nucleosome exclusion [44] nor predicted DNA helical stability [45] of *RIP317-ARS317* is changed by these mutations. These mutations profoundly compromised re-initiation activity, with many of the mutants showing no re-initiation even after 6 hours of induction (Figure S5). These findings suggest that *RIP* elements do not simply act as a DNA unwinding element or a nucleosome exclusion site. We do note that many of the mutations disrupted a palindrome in *RIP317* (5′-TTTATAAA-3′) that is also present in shorter form in *RIP1238* (5′-TTATAA-3′). However, the palindrome in *RIP1238* is not necessary for *RIP* function (Figure S4C, mutant B), and the palindrome in *RIP317* is not sufficient (Figure S5, mutant D2). Thus, although our mutational data does not rule out a role for the palindrome that is specific for *RIP317*, the sequence dependence we observed is consistent with the *RIP* acting as a recruitment site for factors that promote re-initiation.

### *RIP* Function Is Distance Dependent

**Figure 5:**
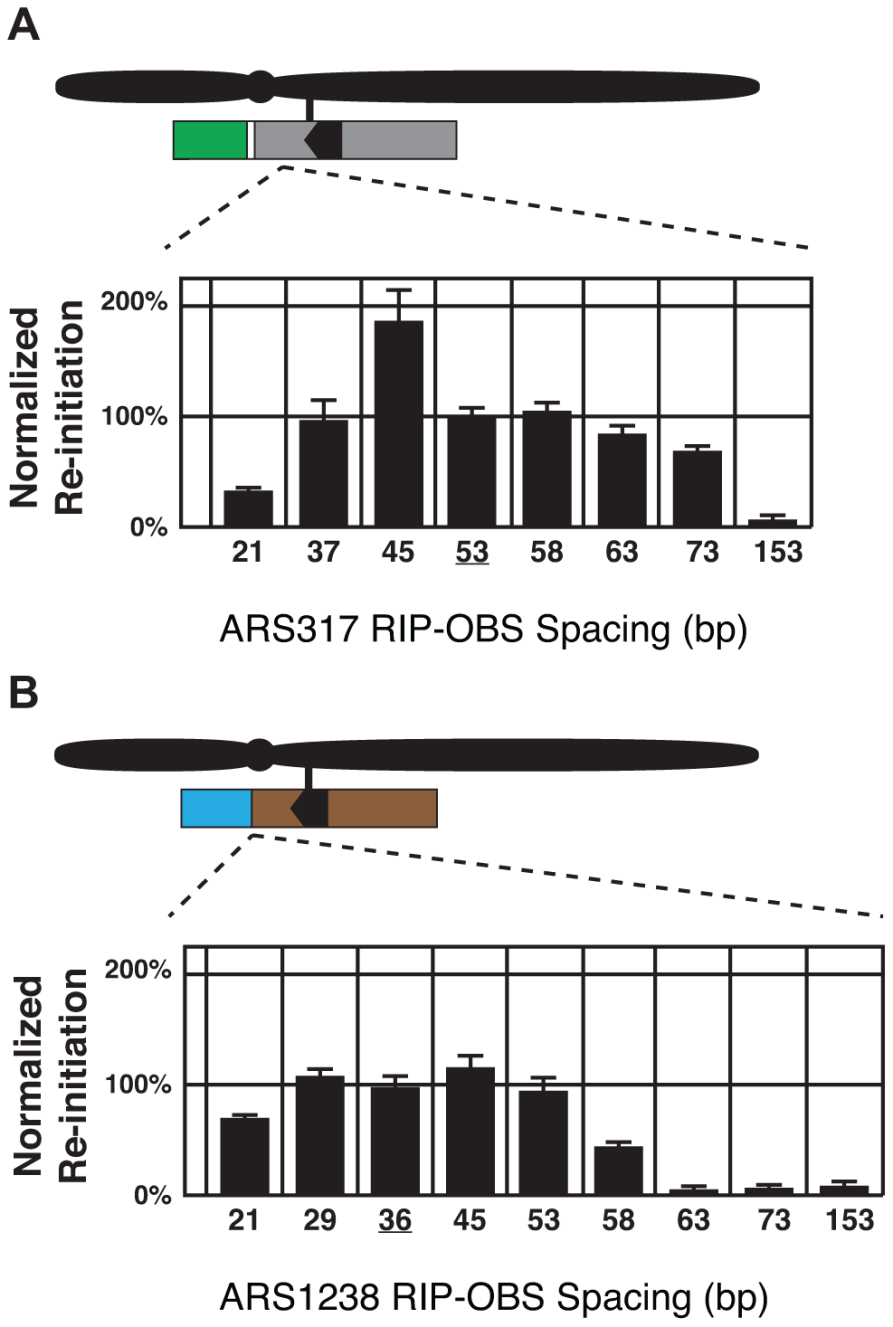
Re-initiation promoters function in close proximity to their origins. (A) Re-initiation efficiency for insertion and deletion mutants that alter the distance between the 67 bp *RIP317* (nt +153 to +87) and the *ARS317 OBS* (nt +33 to +1) in the context of the preferentially re-initiating segment *317(+153..−106)* were determined as described in Figure 4B. These mutant strains change the spacing between the *RIP* and the *OBS* from the wild-type spacing of 53 bp (YJL8398) to 153 bp (YJL8785), 73 bp (YJL8783), 63 bp (YJL8781), 58 bp (YJL8779), 45 bp (YJL8912), 37 bp (YJL8910), or 21 bp (YJL8908). (B) Re-initiation efficiency for insertion and deletion mutants that alter the distance between the 64 bp *RIP1238* (nt +133 to +70) and the *ARS1238 OBS* (nt +33 to +1) in the context of the preferentially re-initiating segment *1238(+133..−100)* were determined as described in A, except re-replication was induced for 6 hr and isogenic strain pairs were normalized against YJL9566 and YJL9567 to obtain efficiencies. Mutant strains change the spacing between the *RIP* and the *OBS* from the wild-type spacing of 36 bp (YJL9566 and YJL9567) to 153 bp (YJL10287 and YJL10288), 73 bp (YJL10289 and YJL10290), 63 bp (YJL10291 and YJL10292), 58 bp (YJL10293 and YJL10294), 53 bp (YJL10158 and YJL10159), 45 bp (YJL10295 and YJL10296), or 21 bp (YJL10299 and YJL10300).

The origin proximal boundary of *RIP317* is 53 bp away from the B-side boundary of the *ARS317 OBS*. To determine whether the size of this spacing is important for *RIP317* function, *RIP-OBS* spacing was increased by inserting randomly generated DNA of 38% AT-content (the average AT-content of genomic DNA in *S. cerevisiae*) between *RIP317* and *ARS317* and decreased by deleting portions of *ARS317* in this 53 bp spacing (See Materials and Methods and Table S1). The resulting clones were analyzed for re-initiation efficiency (Figure 5A). Re-initiation declined with increased spacing and was abolished by 153 bp, suggesting that *RIP317* must be relatively close to the origins to confer preferential re-initiation. Re-initiation could tolerate a decline in spacing to 37 bp but was significantly reduced by a spacing of 21 bp. The latter reduction, however, could simply be a secondary consequence of excessive removal of the B domain, which lies in the 53 bp spacing. Nonetheless, the overall finding is that re-initiation requires close but not precise spacing (within ∼35 to ∼75 bp) between the *RIP* and the *OBS.*

A spacing of only 36 bp separates *RIP1238* from the *OBS* of *ARS1238*. This short spacing suggested that *ARS1238* might re-initiate less efficiently than *ARS317* because the spacing is suboptimal. We thus performed a similar analysis of the spacing requirements between *RIP1238* and the *OBS* of *ARS1238* (Figure 5B). Like *ARS317*, re-initiation of *ARS1238* also required relatively close spacing of the *RIP* and *OBS* (∼25 to ∼55 bp). Moreover, re-initiation levels were relatively constant across this range of spacings, indicating that the lower levels of re-initiation for *ARS1238* versus *ARS317* cannot be attributed to suboptimal *RIP-OBS* spacing for the former. This requirement for close proximity between *RIP* and origin raise the possibility that proteins bound to both sites must closely interact in some manner to facilitate re-initiation.

### *RIP* Elements Confer Preferential Re-initiation on Heterologous Origins

If the *RIP* elements promote preferential re-initiation by influencing common regulatory pathways controlling origins, they should be able to promote re-initiation from heterologous origins. To test this possibility, we fused *RIP317* and *RIP1238* to other replication origins, keeping the spacing between *RIP* and origin *OBS* between 46–53 bp, within the optimal range of spacing determined for both *ARS317* and *ARS1238*. These *RIP*-origin chimeras were then assayed at ChrIV_567 kb for re-initiation in an *MC2Ao* strain.

**Figure 6:**
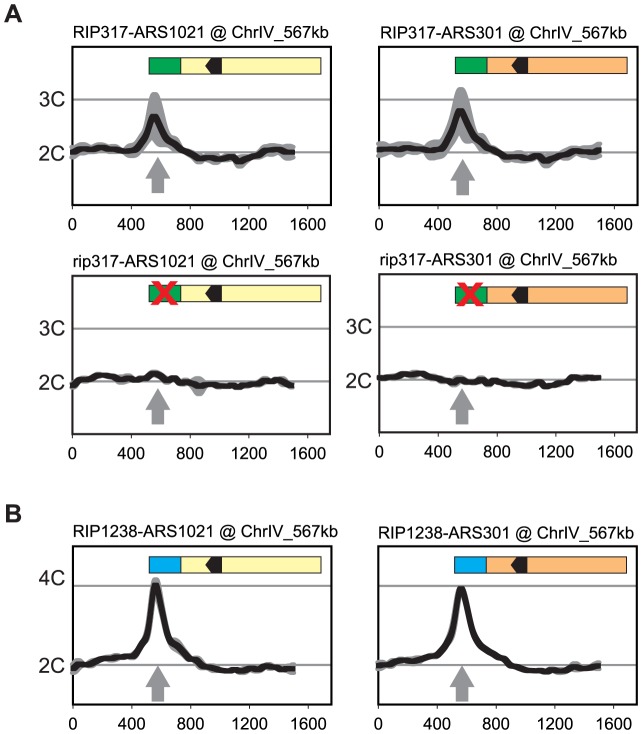
Re-initiation promoters can confer preferential re-initiation on exogenous origins. (A) *RIP317* confers preferential re-initiation on *ARS1021* and *ARS301*. *RIP317-ARS1021* fusion (top left, YJL9078), *rip317-ARS1021* fusion (bottom left, YJL9221), *RIP317-ARS301* fusion (top right, YJL9080), and *rip317-ARS301* fusion (bottom right, YJL9225) were inserted at ChrIV_567 kb (gray arrow) in an MC2Ao strain. The mutant *rip317* has linker L6Xho disrupting the same nucleotides as linker L6 (see Figure 4B). Re-replication profiles (shown for Chromosome IV) were obtained as described for *ARS317* in Figure 2 except the mean profile was generated from duplicate experiments of the indicated strains. (B) *RIP1238* confers re-replication when fused to *ARS1021,* and *ARS301*. A *RIP1238-ARS1021* fusion (left, YJL9999 and YJL10000) or a *RIP1238-ARS301* fusion (right, YJL10001 and YJL10002) was inserted at ChrIV_567 kb (gray arrow) in an *MC2A0* strain. Re-replication profiles were obtained as described for *ARS1238* in Figure 2 using a 6 hr induction of re-replication.

*RIP317* promoted preferential re-initiation from *ARS1021* and *ARS301* (Figure 6A) at levels comparable to the re-initiation it promoted from *ARS317* (Figure 2D) following a 3 hr induction of re-replication (2.8–3C), while fusions to a non-functional rip317 (equivalent to Figure 4B linker 6) failed to re-initiate. *RIP317* also stimulated re-replication from *ARS305, ARS209, and ARS1238*, but a longer 6 hr induction of re-replication was needed to show an unequivocal stimulation (Figure S6A and S6B). *RIP1238* was similarly able to promote preferential re-initiation from *ARS1021* and *ARS301* (Figure 6B). In these cases the re-initiation levels (4-4.5C) were comparable to the re-initiation *RIP317* promoted at *ARS317* following a 6 hr induction of re-replication (compare to Figure 1). Thus, both *RIPs* can promote preferential re-initiation on heterologous origins.

We did observe some origins (*ARS306*, *ARS702*) that exhibited no detectable preferential re-initiation when fused to *RIP317* (Figure S6B). One possible reason is that the optimal spacing between the origin *OBS* and the *RIP* element places constraints on the size of the B domain that can fit between these two elements. Origins requiring larger B domains would be expected to have their initiation, and thus any re-initiation, compromised in their corresponding RIP fusion constructs. Consistent with this explanation, the truncated *ARS306* and *ARS702* fragments fused to *RIP317* displayed defective origin function when assayed by plasmid mitotic stability (Figure S6C).

### *RIP* Elements Do Not Enhance the S Phase Initiation Activity of Adjacent Origins

**Figure 7:**
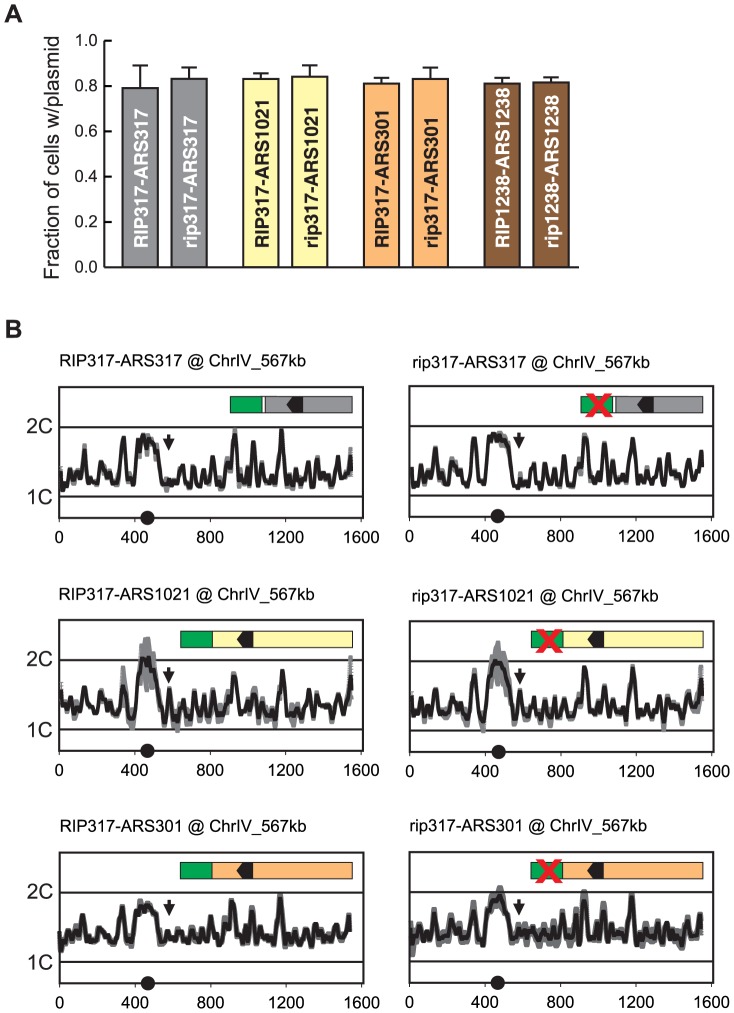
Re-initiation promoters do not alter the initiation activity of origins. (A) *RIP* elements do not enhance mitotic stability of adjacent origins. DNA segments containing combinations of *RIP* elements and origin sequences were cloned into the following plasmids: pCR133 (*RIP317-ARS317*); pCR165 (*rip317-ARS317*); pCR136 (*RIP317-ARS1021*); pCR169 (*rip317-ARS1021*); pCR137 (*RIP317-ARS301*); pCR171 (*rip317-ARS301*); pCR221 (*RIP1238-ARS1238*); pCR313 (*rip1238-ARS1238*). Mitotic stability of these plasmids was measured as described in Figure 3B. (B) *RIP*-origin fragments described in A were inserted at ChrIV_567 kb (arrow) in the following MC2Ao strains: YJL9175 (*RIP317-ARS317*); YJL9248 (*rip317-ARS317*); YJL9177 (*RIP317-ARS1021*); YJL9229 (*rip317-ARS1021*); YJL9179 (*RIP317-ARS301*); YJL9233 (*rip317-ARS301*). Inset shows schematic of *RIP-ARS* fusions: green – *RIP317*; green with cross – *rip317*; black arrowhead – OBS. Strains were synchronously released from an alpha factor arrest into media containing 0.1 M HU and collected in S phase when 30-60% of the genome was replicated. Replicating DNA from these strains was hybridized against nonreplicating DNA from M phase arrested YJL7695. Profiles show DNA content (1C to 2C) from array CGH of each strain plotted against position (in kb) of Chromosome IV. Data shown as mean of two profiles from duplicate experiments of each strain (dark trace) ±SD (light trace).

Just as compromising origin function can reduce re-initiation efficiency, one can imagine that *RIP* elements might promote re-initiation by simply enhancing the intrinsic initiation efficiency of an origin. Such an effect was difficult to detect by plasmid mitotic stability because origins that re-initiate when fused to *RIP317 (ARS317, ARS1021, and ARS301*) appear to have maximal mitotic stability in this assay (Figure 7A). However, when integrated in the chromosome, *ARS317*, *ARS1021*, and *ARS301* exhibited much lower initiation activity, allowing us to look for stimulation of this activity by *RIP317*.

We used array CGH analysis of S phase replication to assay the activity of these origins with, and without, a functional *RIP317* element. In the resulting replication profiles, the heights of the peaks represent a combination of the efficiency and timing of origin initiation in S phase. Low but measurable peak heights for the origins are ideal, because they leave open the maximal dynamic range for detecting a stimulation of origin activity by *RIP317.*

We observed no measurable difference in replication peak heights for *ARS317*, *ARS1021*, and *ARS301* with or without a functional *RIP317* (Figure 7B). At its endogenous location *ARS317* initiates in approximately 10–15% of cells each S phase based on 2-dimensional gel analysis of initiation bubble intermediates [46], [47]. Such origin activity at ChrIV_567 kb would be at the limit of detection for our aCGH replication assay, and any significant *RIP317* stimulation of *ARS317* activity should have been detectable as a larger peak. More striking is the detection of clear origin activity from *ARS1021* and the absence of any stimulation of this activity from *RIP317*. These results argue that *RIP317* does not advance the timing or enhance the initiation efficiency of adjacent origins. We thus favor a model in which *RIP* elements specifically promote re-initiation by antagonizing a mechanism(s) that prevents re-initiation.

### *RIP* Elements Facilitate a Step after Mcm2-7 Association with Origins

*In vitro* studies have shown that the loading of Mcm2-7 at origins can be subdivided into a sequence of discrete steps: (1) binding of ORC to origins; (2) recruitment of Cdc6 to ORC; (3) recruitment of Cdt1-Mcm2-7 to ORC-Cdc6; and (4) loading of a double hexamer of Mcm2-7 as a ring around the duplex origin DNA [48]. The numerous global mechanisms used by CDKs to prevent Mcm2-7 loading are thought to inhibit one or more of these steps, because once Mcm2-7 loading is complete, origins are primed to be activated by CDKs [49]–[51]. The partial deregulation of these mechanisms in the *MC2Ao* strain presumably allows some but not all of these steps to proceed, accounting for why the majority of origins do not re-initiate. *RIP* elements could therefore function by locally releasing an origin from the remaining block(s), allowing the origin to complete a re-initiation cycle. Thus, to gain insight into the mechanism of *RIP* action, we investigated which step in the loading process was blocked for the majority of origins that do not re-initiate in *MC2Ao* strains.

**Figure 8:**
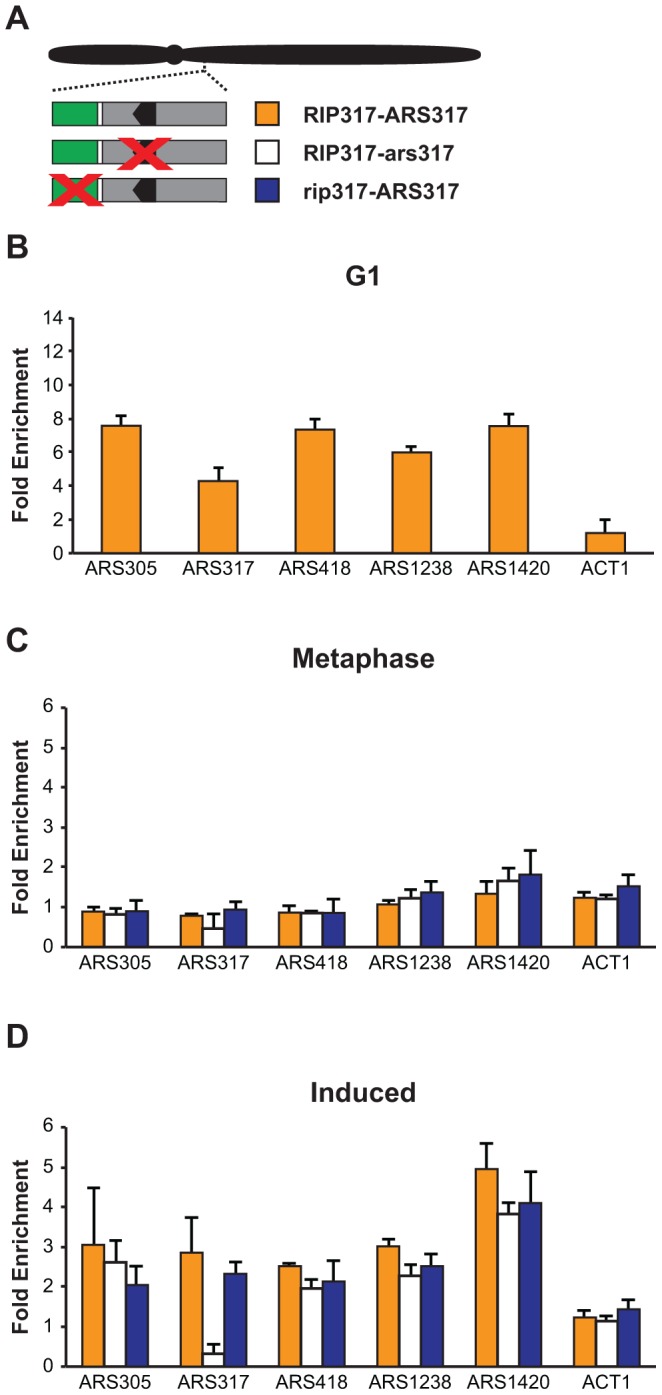
Deregulation of Cdc6 and Mcm2-7 allows Mcm2-7 to re-associate with origins that do not preferentially re-initiate. (A) Schematic of *RIP-*origin fragments inserted at ChrIV_567 kb In *MC2Ao* strains. YJL8398 (*RIP317-ARS317,* orange) is described in Figure 4B. YJL8541 (*RIP317-ars317*; white) has *ACSs* of both *ARS317* and the nearby cryptic origin disrupted using the mutations *HMRE-A* and *HMRE-E* (described in Figure S3). YJL9244 (*rip317-ARS317*; blue) has the *RIP* disrupted with linker L6 as described in Fig 4B. In all strains, the endogenous *ARS317* is deleted. (B) Mcm2-7 associates with origins at a G1 arrest. ChIP association with the indicated origins and non-origin control ACT1 was measured using anti-Mcm2-7 polyclonal antibodies in alpha factor arrested cells. Data shown as mean +/- SD (n = 3) of DNA enrichment relative to two non-origin segments (ADH1 and SLH1). (C) Mcm2-7 does not associate with origins in an M phase arrest. Mcm2-7 association with DNA segments shown in A was measured as described in B at a metaphase arrest before induction of re-replication. (D) Mcm2-7 associates with origins when re-replication is induced. Mcm2-7 association with DNA segments shown in A was measured as described in B at a metaphase arrest after 1.5 hr of re-replication induction.

We examined Mcm2-7 ChIP association at three origins that do not re-initiate in *MC2Ao* strains: *ARS305*, *ARS418*, and *ARS1420*. As expected, Mcm2-7 associated more with these origins relative to nonspecific DNA in G1 phase (Figure 8B) but not in M phase (Figure 8C). After a 90 minute induction of re-replication, Mcm2-7 became enriched 2–4× at these origins but not at a non-origin locus *ACT1* (Figure 8D). ChIP also detected a similar degree of re-replication-induced association of Mcm2-7 with the two re-initiating origins, *ARS317* and *ARS1238* (Figure 8D). As expected, given the association of Mcm2-7 with origins that cannot re-initiate, preventing re-initiation of *ARS317* by disrupting its adjacent *RIP317* did not prevent the association of Mcm2-7 with *ARS317* (Figure 8D). On the other hand, disrupting the ORC binding site in *ARS317*, did lead to loss of Mcm2-7 association, specifically with this origin (Figures 8C, 8D). This result is consistent with the *in vitro* dependence of Mcm2-7 origin association on ORC binding [52].

Taken together, our data indicate that the global deregulation of re-initiation in the *MC2Ao* strain allows Mcm2-7 to associate with most origins. Thus, in this strain the *RIP* elements must promote re-initiation at adjacent origins by facilitating or deregulating a step that is blocked after this association. As discussed below, determining more precisely which step is involved will require better *in vivo* tools to distinguish between the two types of association (Mcm2-7 recruitment versus loading) that have been identified *in vitro*.

## Discussion

### *RIP* Elements Contribute to the Diversity of Origin Re-replication Control

Preventing re-initiation at the hundreds to thousands of replication origins in a eukaryotic genome is critical for preserving genome stability [2]. Models for how such tight regulation can be achieved emphasize the importance of using numerous overlapping inhibitory mechanisms to reduce the probability that any origin will re-initiate [3], [8]. These mechanisms all inhibit the loading of the Mcm2-7 core replicative helicase onto origins, and each does so by reducing the total cellular activity of one of the four proteins required for this step: ORC, Cdc6, Cdt1, or Mcm2-7 [5], [6]. Given their global nature, these regulatory mechanisms are presumed to act equally at all origins throughout the genome. Thus, current models cannot account for the broad range of efficiencies with which origins re-initiate when global mechanisms are compromised. This diversity suggests that the models may be missing the contribution of local factors that can modulate the regulation of individual origins.

Our work here demonstrates that such a local layer of regulation does indeed exist by identifying a local control that makes *ARS317* and *ARS1238* more susceptible to re-initiation when global regulation of Cdc6 and Mcm2-7 is removed. Our analysis of this control establishes some of its key mechanistic properties and constraints. First, this control specifically enhances the propensity of an origin to re-initiate and not its efficiency or timing during normal S phase initiation. Second, this preferential re-initiation is not imposed by a diffuse chromosomal context but is conferred by discrete sequence elements that are adjacent to but distinct from the origin. Third, these elements, which we call re-initiation promoters (*RIPs*), have specific sequence requirements and function best within a narrow range of distances close to the origin. Finally, these *RIPs* appear to overcome inhibitory mechanisms that block a step in initiation that follows the association of Mcm2-7 with origins. These results provide a paradigm for the local control of origin re-initiation and lay the groundwork for a more detailed molecular analysis of this control.

Our results do not address the question of whether the presence and activity of these *RIPs* is incidental to some other genomic function of these elements or whether they arose for the purpose of modulating replication control in cells with intact replication controls. Nonetheless, as discussed below, the existence of *RIP* elements has potential biological ramifications in both mutant and wild-type settings.

### Is *RIP* Function Mediated by Protein Binding?

One of the questions raised by our results is whether *RIP* function is mediated by proteins that specifically recognize these sequences or is mediated by some other property of these elements. The two *RIP* sequences we identified, *RIP317* and *RIP1238*, are both AT-rich, especially in their core regions. They do not share an obvious consensus sequence, and in fact, their AT-rich character makes it difficult to find meaningful conservation of these elements throughout the genome. Importantly, this AT-rich character raises the possibility that these elements just act thermodynamically to facilitate the DNA unwinding needed to re-initiate DNA replication. Another possibility is that they simply influence nucleosome positioning around origins, as AT-rich DNA tends to be excluded from nucleosomes [53]. These hypotheses, however, are not sufficient to account for *RIP* function, because we were able to abrogate *RIP317* function using mutations that preserved AT content without significantly perturbing their calculated unwinding potential or predicted propensity to exclude nucleosomes [44], [45].

These considerations suggest to us that *RIP* elements may act through proteins that bind to them. Such a possibility is compatible with the poor nucleosome occupancy over *RIP317* that has been observed at its endogenous chromosomal location [35], [54], [55]. A quick attempt to uncover such proteins by screening through yeast transcription factors with potential binding motifs [56], [57] in both *RIP317* and *RIP1238* did not yield any promising candidates (See Materials and Methods); deletions in *NHP6A NHP6B, YAP1, SUM1, YNR063W, GAT4, SMP1, or YOX1* failed to disrupt the function of either *RIP*. Hence, we are pursuing more systematic studies to identify proteins that bind *RIP* elements in vivo and are essential for *RIP* function. If *RIPs* do indeed work by recruiting proteins near an origin, the distance dependence of *RIP* function suggests that these proteins may have to interact in close proximity with specific initiation or regulatory protein that assemble at origins.

### *RIP* Elements Deregulate a Block to Re-initiation That Occurs after Mcm2-7 Associates with Origins

Our work also demonstrates that origins that do not re-initiate in the MC2Ao strain associate with Mcm2-7 by ChIP analysis and thus can at least recruit Mcm2-7 to origins. Apparently, these origins are blocked at an initiation step subsequent to Mcm2-7 recruitment, and the *RIP* elements confer preferential re-initiation on neighboring origins by deregulating this step.

Exactly which step is deregulated by *RIP* elements is not resolved by our experiments, but there are two major possibilities. The elements could be deregulating the transition between Mcm2-7 recruitment and Mcm2-7 loading, which has been defined *in vitro* [58] but not yet demonstrated *in vivo*. Alternatively, they could be deregulating a step following Mcm2-7 loading. We favor the former possibility because the latter requires us to violate a fundamental principle of the current paradigm for re-initiation control [3], [5], namely that this control only targets steps preceding Mcm2-7 loading. Nonetheless, resolution of this question must await the development of more sophisticated *in vivo* protein-DNA binding assays that are capable of distinguishing recruited from loaded Mcm2-7 at individual origins.

Importantly, this role in enabling a step of initiation subsequent to Mcm2-7 origin association distinguishes *RIP* elements from B2 elements, one of the core elements of budding yeast origins. Both elements are AT rich, positioned 3′ of the T-rich strand of the *OBS*, and have relaxed positioning requirements relative to the *OBS*. However, the B2 elements are needed for Mcm association with origins [59], and *RIP* elements are not. This distinction provides further support for a model in which *RIP* elements antagonize an inhibitory mechanism, rather than simply promote a normal initiation function.

### Possible Inhibitory Pathways Targeted by *RIP* Elements

How might *RIP317* and *RIP1238* locally override a block to Mcm2-7 loading that prevents origins from re-firing in the *MC2Ao* background? The simplest model is that the block is imposed by one or more of the regulatory mechanisms that remain intact in *MC2Ao* strains, e.g. CDK phosphorylation of Orc2 and Orc6 [7] or CDK binding to Orc6 [22]. According to this model, *RIP* elements locally antagonize some or all of these mechanisms, relieving enough of the block to allow detectable re-initiation at *RIP*-associated origins. This model is consistent with *in vitro* studies that indicate these inhibitory mechanisms still permit ORC binding and some Mcm2-7 recruitment to origins, but completely block Mcm2-7 loading onto origins [58]. The model is also consistent with our observation that globally antagonizing CDK phosphorylation of ORC in the *MC2Ao* background by mutating all CDK consensus phosphorylation sites on Orc2 and Orc6 allows many origins to join *ARS317* and *ARS1238* in re-initiating at detectable levels [12]. However, direct support for this model will require analysis of ORC phosphorylation and *CDK* binding at origins to determine if they are indeed reduced at *RIP*-associated origins as might be predicted by the model.

We note that the induction of re-initiation in the *MC2Ao* strain is limited and slow compared to the usual efficiency of origin initiation in a normal S phase. After 3 hr of induction, over one and a half cell cycles for this strain, only 50% and 25% of *ARS317* and *ARS1238*, respectively, have re-initiated. This inefficient re-initiation suggests that *RIP317* and *RIP1238* only partially antagonize the inhibitory mechanisms blocking Mcm2-7 loading. Such incomplete relief of inhibition may explain why completely antagonizing inhibitory phosphorylation of Orc6 on one CDK consensus site (S116A) can further enhance *ARS317* and *ARS1238* re-initiation in the *MC2Ao* strain relative to the *MC2A* strain (Figure S1).

### Parallels to Localized Re-initiation during Development

The preferential re-initiation of *ARS317* and *ARS1238* is reminiscent of the localized re-initiation that occurs in several cases of developmentally programmed gene amplification [60]. One of the best characterized is the amplification of the chorion gene locus in Drosophila ovarian follicle cells during oogenesis. Like the *RIP* elements identified in this work, an Amplification Control Element (*ACE3*) of ∼320 bp has been identified that has little origin function on its own and confers preferential re-initiation on a nearby origin (*ori-beta*). However, the mechanism by which *ACE3* and other potential ACE elements promote re-initiation at a select group of origins remains a mystery [60].

Our work in budding yeast offers a conceptual framework for exploring the mechanism of developmentally regulated gene amplification, even if the details prove to be different. For example, characterizing how far the initiation reaction can proceed on the majority of origins that don't re-initiate may give insight into the key step that allows amplification origins to re-initiate. Similarly, it may be informative to investigate the status of inhibitory modifications on initiation proteins associated with re-initiating origins to see if these modifications are reduced relative to the bulk protein population.

### Preferential Re-initiation May Predispose Genomic Regions to Re-replication Induced Genetic Variation

In addition to its established role in developmentally programmed gene amplification, there are several hints that DNA re-replication may also contribute to the amplifications and abundant duplications observed in cancer cells. First, we have shown in budding yeast that re-replication arising from deregulated replication initiation proteins can be an extremely efficient source of segmental amplification [2]. Second, overexpression of initiation proteins in murine models has been shown to promote oncogenesis [61]–[63]. Third, overexpression of replication initiation proteins has been observed in some human cancer cells [64]–[67]. And finally, the tandem direct repeat structure of some oncogene amplifications and many of the duplications detected in cancer cells is consistent with the structures that could arise from re-replication [68].

Should re-replication prove to be a new source of copy number variation (and possibly other genomic alterations) in cancer cells, local modulation of origin control, such as that described in this work, could make some regions of the genome more susceptible to re-replication induced genetic alterations than others. One can therefore imagine that an irregular genomic landscape of re-initiation susceptibility could give rise to an irregular genomic landscape of genetic instability in cancer cells. Preliminary indication for such position dependent variability in genetic instability has been obtained by experiments showing that the frequency and structure of DHFR amplification in a cancer cell line was different for different genomic positions of DHFR [69].

Copy number variation may also play an important role in normal cells. For example, gene duplications are thought to provide the functional redundancy that enables the functional diversification of genes during molecular evolution [70]. In addition, copy number increases, which occur with high prevalence in normal human genomes [71], may directly provide phenotypic variation that can be selected for during evolution. In both examples, the mechanism of copy number change is not clear. We speculate that extremely rare re-initiation events may occur despite the presence of normal re-initiation controls and contribute to copy number increases. Should re-initiation drive some of these copy number increases, variable susceptibility of origin re-initiation throughout the genome would be expected to make some regions of the genome more subject to evolutionary change than others. Thus, the presence of a local layer of re-initiation control provided by *RIP*-like elements may have far reaching ramifications on oncogenesis and evolution.

## Materials and Methods

### Plasmids

Integrative plasmids were used to test *RIP*-origin re-replication or replication activity in a chromosomal context. These plasmids were all derived from pBJ2889 [2]. This plasmid contains a portable re-replication integration cassette made up of the following elements: Homology Left (sequences centromere proximal to *ARS419,* which is located at 567 kb on Chromosome IV), the *kanMX6* reporter gene [72], the *ade3-2p* color reporter gene [73], a polylinker, which includes the XbaI restriction site, and Homology Right (sequences centromere distal to *ARS419*). SpeI – XbaI fragments containing *RIP*-origin inserts and additional restriction sites were integrated into the XbaI site of the pBJL2889 polylinker, creating a SpeI/XbaI fusion site (TCTAGT) on the *ade3-2p* side of the insert and re-creating an XbaI (TCTAGA) site on side adjacent to Homology Right. We report the sequence of these clones in Table S1 from the SpeI/XbaI fusion site to the intact XbaI site. The re-replication integration cassette was excised from the plasmid using SacI-NotI or SacI-SalI and introduced into yeast using standard techniques. Integration of these cassettes at *ARS419* destroyed its origin activity.

The *ARS* activity of RIP-origin constructs was measured by mitotic stability assays utilizing centromere-containing plasmids. These *CEN-ARS* plasmids were derived from pFJ11 [36], a plasmid containing *ARS317* and *CEN4*. As a preliminary step, the BamHI site adjacent to *CEN4* was destroyed by BamHI digestion, klenow fill-in of the cut overhangs, and blunt-end ligation. The *ARS317* in this modified pFJ11 was then replaced with our origin or *RIP-*origin constructs by cloning these constructs into the HinDIII and EcoR1 sites of the plasmid (exact sequences listed in Table S1). These plasmids were transformed into YJL310 [74] using standard techniques.

### Altering *ARS317* and *ARS1238 RIP*-*OBS* Spacing

The full sequence of all insertion and deletion mutants used to alter RIP-OBS spacings are listed in Table S1. They were generated as follows:

#### Inserting sequence

To increase the distance between *RIP1238* and the *OBS* of *ARS1238* we first randomly generate a 117 nucleotide sequence of 38% GC content (matching the average GC content of *S. cerevisiae*) DNA sequence then manually altered it to be free of yeast transcription factor binding motifs:

5′-ATAGCCTGCCCATAGGATATAGAGATACCAATAGTTGTTTGTGAACAGCAAAGAAGGATCCAGAAGATCAGTCGCACGATATTGATGTGAATACTAGGTTTATAGGATAGTCGTACA - 3′

Various sized segments of this sequence, all spanning the BamHI site (underlined), were inserted between nucleotides +69 and +70 of *ARS1238*. A 100 nucleotide sequence was similarly generated to insert sequences between *RIP317* and the *OBS* of *ARS317*:

5′–CCCATAGGATATAGAGATACCAATAGTTGTTTGTGAGCAACAAAGAAGGATCCAGAAGGTCGATCGCACGATATTGATGTGAATACTAGTTGTAGTAATG – 3′

#### Deleting sequence

For *ARS317* BamHI linker mutants L19, L21, L23, and L27 described in Figure 4B were digested with BamHI and ligated together to produce 8 bp (L23–L27), 16 bp (L21–L27), and 32 bp (L19–L27) deletions. For *ARS1238*, sequences +69..+55 and +69..+51 were deleted from the left border of *ARS1238*.

### Strains

Genotypes and derivations for all strains used in this manuscript can be found in Table S2. Almost all the *MC2Ao* yeast strains in this paper were generated from the previously published strain YJL3758 [2] by one or more of the following genetic alterations: (1) integration of a re-replication cassette (described in Plasmids above and detailed in Table S1); (2) deletion of *ARS317*, *ARS418*, or *ARS1238* (Table S3); (3) deletion of *SIR* or *FKH* genes (Table S3) [72], [75], [76]. *MC2A* strains YJL8923 and YJL8924 are congenic to YJL3758 but have wildtype *ORC6* instead of *orc6(S116A)*.

### Oligonucleotides

Oligonucleotides used to PCR marked deletion fragments for deleting origins or genes encoding transcription factors are listed in Table S3. Oligonucleotides used in quantitative PCR are listed in Table S4.

### Strain Growth and Induction of Re-replication

Synthetic complete medium containing 2% wt/vol dextrose (SDC) was made up as described [77] except that we used twice the concentration of amino acids and purines for all but leucine, which was added to a final concentration of 120 µg/mL, and serine, which was added to a final concentration of 200 µg/mL. Drop out media like SDC-URA, simply lacked the indicated component. For nonselective rich media cells were grown in YEPD (YEP +2% wt/vol dextrose) or YEPRaf (YEP +3% wt/vol raffinose +0.05% wt/vol dextrose). All cell growth was performed at 30°C.

To induce re-replication, freshly thawed log phase cultures in YEPD were extensively diluted into YEPRaff and grown for 12–15 hr until they reached an OD600 of 0.2–0.8. At this cell density (approximately 1×10^7^ cells/ml), nocodazole (US Biological N3000) was added to a final concentration of 15 µg/mL for 120–135 min to arrest cells in metaphase. *GAL1* promoter driven *pGAL-Δntcdc6,2A* was then expressed by the addition of 2–3% galactose for 3 hr or 6 hr where indicated.

### Strain Growth for Replication Arrays

Strains were grown overnight in YEPD at 30°C to an OD600 of 0.2–0.4. At this cell density, 50 ng/mL alpha factor was added to arrest cells in G1 phase. Arrested cells were released into fresh YEPD media containing 0.1 M hydroxyurea (US Biological H9120), 100 µg/mL pronase (EMD 53702), and 15 µg/mL nocodazole (US Biological N3000) to permit a single, slowed S phase to occur. Cultures were harvested after 135 minutes when 30–60% of the genome was replicated as verified by FACS analysis [78]. To increase the sensitivity of detecting initiation activity from the integrated re-replication cassettes, we deleted the closest early origin *ARS418* so that its forks would not run through the origins in the cassettes and preclude their initiation.

### Genomic DNA Preparation for aCGH Analysis

#### Method 1

Genomic DNA was extracted from yeast as described [2], [79]. Briefly, 10–25 OD units of cells were harvested and lysed by bead beating. DNA was extracted by phenol:chloroform:isoamyl extraction, ethanol precipitated, and resuspended in 50 µL of 2 mM Tris-Cl (pH 7.8). Typical yields were 2–5 µg of DNA.

#### Method 2

Larger cultures (>100 OD units) were subjected to a more extensive purification consisting of organic extraction, enzymatic removal of protein and RNA, detergent (cetyltrimethylammonium bromide) treatment, and DNA isolation using anion-exchange columns (Qiagen #10243 100/G tips). Typically, this protocol was performed to produce 50–120 µg of M phase arrested DNA for aCGH. Full details of this protocol are described in [2] and [12].

### Array CGH: DNA Labeling, Hybridization, and Scanning

#### aCGH analysis of whole genome (Used in Figure 1)

A single large (>250 OD units) culture was the initial source for both reference (non-induced) and re-replicated DNA. Half of this culture was harvested at the arrest (0 hr) to generate the uninduced reference DNA. The remaining culture was induced with galactose for 6 hr before harvesting to generate the induced re-replicated DNA. 1.5–2 µg of reference DNA (prepared using Method 2 above) was labeled with Cy3, and 1.5–2 µg of 6 hr induced DNA was labeled with Cy5 essentially as described [12]. The labeled DNA was hybridized as previously described [12].

#### aCGH analysis of re-replication (Used in Figures 2–6 & Figures S1–S6)

2–2.5 µg of each DNA sample (prepared using Method 1 above) was labeled with Cy5 and 1.5–2 µg of purified M phase reference DNA from YJL7695 (prepared using Method 2 above) was labeled with Cy3 using a low-throughput [12] or high-throughput [80] method. All samples were hybridized as described [12].

#### aCGH analysis of replication (Used in Figure 7)

1.5–2 µg of each experimental DNA sample (prepared using Method 2 above) was labeled with Cy5, and 1.5–2 µg of purified M phase reference DNA from YJL7695 (prepared using Method 2 above) was labeled with Cy3 essentially as described [12]. The labeled DNA was hybridized as previously described [12].

### Array CGH: Data Analysis

Full details of array CGH data analysis are described in [12]. Briefly: arrays were scanned on a GenePix 4000B scanner and quantified using GenePix 6.0 (Axon Instruments). The Cy5/Cy3 ratios were normalized such that the average ratio was equivalent to DNA content for that specific point in the cell cycle (e.g. 2C for M arrested or induced samples, and 1.5C for S phase samples). Medians for these raw normalized data were then calculated across a 10 kb moving window. Smoothed curves were calculated from this moving median using Fourier Convolution Smoothing (FCS). The degree of smoothing is determined by a parameter called the convolution kernel [81], and for the chromosomes we display we used the following values optimized for re-replication profiles: Chromosome III, 9; Chromosome IV, 11.25; Chromosome V, 9; Chromosome XII, 10.75. For S phase replication profiles, the convolution kernel for Chromosome IV was set to 6.25. For presentation purposes, smoothed lines for each individual re-replication or S-phase profile were averaged into one composite profile. Most figures in the manuscript show these composite profiles as black lines surrounded by a gray zone representing ±1 standard deviation. The raw data and the smoothed lines for each individual experiment performed for this work can be seen in Document S1.

We note that, because of cross hybridization among the various repetitive sequence elements, these elements (tRNA genes, subtelomeric repeats, Ty elements and long terminal repeats) were removed from the analysis. In the Saccharomyces Genome Database, the two rDNA genes representing the large rDNA repeat arrays are adjacent to a Ty element and additional repeated sequences, so the entire ∼44 kb region between YLR153C and YLR163C was omitted from the analysis.

Also, because each chromosome was effectively circularized during the calculation of the moving window median and the FCS, deviations of the smoothed curve from baseline values at one chromosome end can artifactually cause the curve to deviate from baseline at the other end [82]. Thus, when *ARS317* preferentially re-initiated at its endogenous location near the right end of Chromosome III, it caused the smoothed re-replication curves to rise at the left end. We have masked the left 20 kb of the smoothed re-replication curves for Chromosome III in Figures 1A, 2B, and S1A, but left the curves unmasked in the individual experimental profiles shown in Document S1.

### Comparison of Array Profiles in Bar Graph Format

Bar graphs were generated to compare the amount of re-initiation seen in experimental vs control strains. aCGH re-initiation peak heights were measured relative to the expected G2/M copy number (2C) for both experimental and control strains. Replicates of each array were then averaged (x_exp_ and x_cont_) and a standard deviation calculated (s_exp_ and s_cont_). The ratio x_ratio_ formed by x_exp_ divided by x_cont_ was converted to a percentage and plotted as shown. The error for this ratio was calculated by solving the equation:
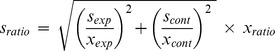



### Statistical Analysis of Array Profiles for sirΔ and fkhΔ Strains

Re-replication of each experimental strain (n = 2) was measured at one of the following re-replicating loci: ChrIII_292 kb (endogenous *ARS317*), ChrIV_567 kb (transplanted locus), or ChrXII_889 kb (endogenous *ARS1238*). Relevant control strains lacking (negative control) a re-replicating origin at each location were measured to provide a background (i.e. non re-replicating) baseline. Sample size for these negative control strains ranged from n = 5 to n = 10 as indicated in figure legends. Mean profile heights of the experimental and negative control strains were compared using Welch's t-test. Significant (p<0.05) results reject the null hypothesis and confirm that re-replication of *sir*Δ and *fkh*Δ strains is significantly different from re-replication of the relevant negative control strain.

### Mitotic Stability Assay

*CEN-ARS* plasmids containing *RIP*-origin, *RIP,* or origin constructs were transformed into YJL310 [77], a strain with intact re-replication controls. Three independent transformants were inoculated into media selective for the plasmids (SDC-URA) and grown overnight to saturation. Cultures were subsequently diluted back into fresh selective media and grown overnight to an optical density of 0.1–0.6. Each log phase culture was plated to five selective (SDC-URA) and five non-selective plates (SDC) at a density of 200–400 cfu/plate. Plates were grown for 3-4 days and the fraction of cells harboring a plasmid was determined by dividing the number of colonies on the selective plates over the number on non-selective plates. Values reported are averaged from the three independent plasmid transformants.

### Chromatin Immunoprecipitation (ChIP)

ChIP experiments were performed with approximately 20 OD units of cells in a media volume of 50 mL. Cultures were handled as described above for re-replication cultures except induction was restricted to 90 minutes. We reasoned that anti-Mcm ChIP would work best immediately after Mcms were re-loaded onto origin DNA but before most of these origins had re-fired and distributed Mcms throughout the genome. Thus, we selected the 90-minute induction time point as this was the latest induction time before re-replication became visible by array CGH. This rationale is similar to that used in earlier ChIP-chip analysis of re-replicating strains [13].

Terminal cultures were fixed by addition of formaldehyde (37% w/v) to a final concentration of 1%. Fixation proceeded for 15 minutes at room temperature and was quenched by the addition of glycine to a final concentration of 0.125 M. Fixed cells were harvested by centrifugation, washed once in 1× TE pH 7.5, and frozen at −80C.

Cell pellets were resuspended in 500 µL lysis buffer (50 mM HEPES/KOH pH 7.5, 140 mM NaCl, 1 mM EDTA, 1% Triton, 0.1% Na-Deoxycholate) with protease inhibitors (Roche mini complete #04693159001+2 mM PMSF) and transferred into 2 mL screw-cap tubes (Sarstedt #72.694.006). 0.5 mm glass beads (Biospec Products 11079–105) were added to the level of the meniscus and cells were disrupted using a FastPrep 24 for two cycles of 45 sec at 6.0 m/s with 2 min incubation on ice in between. All subsequent steps were performed in low adhesion DNAse/RNAse free 1.5 mL microfuge tubes at 4°C unless otherwise indicated. Lysates were cleared by centrifugation at 20,000 rcf for 10 min and pellets (containing chromatin) were resuspended in 500 µL of fresh lysis buffer + protease inhibitors. Each pellet was sonicated using a 1/8” tapered microtip attached to a Branson 450 sonicator for 4 cycles of 30 sec at setting 1.5 with >2 min on ice in-between. The resulting slurry was cleared again by centrifugation at 20,000 rcf for 10 min and the supernatant was retained as whole cell extract (WCE).

Immunoprecipitation, washes, and elution were performed on 80–90% of the WCE volume using methods described in [83]. These extracts were exposed to UM174 antibodies (rabbit polyclonal anti-Mcm2-7, 1∶500 dilution) [58] (generous gifts from Steve Bell) in the presence of 30 uL slurry of Protein G Dynabeads (Life Technologies, 10004D). Immunoprecipitations were performed for 20 hr at 4°C. Beads were washed 3× with 1 mL of Wash Buffer (10 mM Tris-Cl pH 8, 250 mM LiCl, 0.5% NP-40, 0.5% Na-Deoxycholate, 1 mM EDTA) and 1× with 1 mL of TE (10 mM TrisCl pH 8, 1 mM EDTA) with 50 mM NaCl. DNA was eluted from the beads by incubating them in 100 µL of 65°C Elution Buffer (50 mM Tris-Cl pH 8, 10 mM EDTA, 1% SDS) for ten minutes.

Crosslink reversal and DNA purification was performed essentially as described in [84]. Briefly, IP samples were digested in proteinase K (final concentration 1 mg/mL) for 2 hr at 37°C and incubated at 65°C for 6 hr to reverse crosslinks. WCE samples omitted the proteinase K but were otherwise subjected to the same incubation conditions. DNA from both IP and WCE were purified using PCR purification columns (Qiagen Inc 28106) and eluted into 300 µL of 1× TE pH 8.

### Quantitative Real-Time PCR (qPCR)

For each genotype, three independent cultures were analyzed and the average fold enrichments of origin DNA by ChIP were reported. The IP and WCE DNA samples from each individual culture were analyzed in triplicate on a Stratagene MX3000P qPCR machine using primer pairs listed in Table S4. Each reaction was performed using Power SYBR Green PCR Master Mix (Applied Biosystems) in a total volume of 20 µL with primers at a final concentration of 300 nM. Because of the AT-rich nature of template origin DNA, we used an annealing temperature of 57°C and an extension temperature of 65°C. Fold enrichment of the assayed DNA segments over the average of two non-origin DNA segments (*ADH1* and *SLH1*) was calculated using the 2^-ΔΔCt^ method essentially as described [85].

### Identifying and Testing Candidate RIP Binding Factors

The UNIPROBE database of *in vitro* DNA binding specificities [57] was searched using *RIP317* and *RIP1238* sequences. The search was restricted to *S. cerevisiae* datasets and the stringency filter was set to the lowest setting. Nonessential candidate RIP-binding proteins found in both sequences were *NHP6A NHP6B, YAP1, SUM1, YNR063W, GAT4, SMP1,* and *YOX1*. These factors were knocked out genetically and the resulting strains were tested for re-replication activity at *ARS317* and *ARS1238.*

### Accession Numbers

All array CGH data from this study have been deposited in the Gene Expression Omnibus (GEO) (http://www.ncbi.nlm.nih.gov/geo) database (Series Accession #GSE55420).

## Supporting Information

Figure S1 Preferential re-replication is enhanced by, but does not require, the *orc6-S116A* allele. Re-replication profiles for Chromosome III containing *ARS317* at its endogenous locus were generated from isogenic *MC2A* strains (YJL8923 and YJL8924, black trace) and isogenic *MC2Ao* strains (YJL3758 and YJL3759, gray trace) and displayed as in Figure 2. Data shown as mean of two profiles (dark trace) ±SD (light trace).
(EPS)

Figure S2 Preferential re-initiation of *ARS1238* does not require *SIR* and *FKH* genes. (A) Quantification of re-replication profile peak heights for *sir*Δ strains described in Figure 2B. DNA copy number at the endogenous *ARS317* locus (ChrIII_292 kb) was plotted as a bar graph. Black lines indicate significant difference (p<0.05 for Welch's t-test) between experimental and negative control (*ars317*Δ) strains. (B) Quantification of re-replication peak heights for *fkh*Δ strains described in Figure 2D. DNA copy number at ChrIV_567 kb was plotted as a bar graph. Black lines indicate significant difference (p<0.05 for Welch's t-test) between experimental and negative control (*ars317*Δ) strains. (C) Preferential re-initiation of *ARS1238* is independent of transcriptional silencing genes *SIR1-4*. Left panel: re-replication profiles of Chromosome XII containing *ARS1238* at its endogenous location (gray arrow). Profiles for positive (WT, YJL8398, n = 10) and negative (*ars1238*Δ, YJL9152, n = 7) control strains are overlaid with *sir*Δ strains described in Figure 2B. Right panel: DNA copy number at the endogenous *ARS1238* locus (ChrXII_889 kb) plotted as a bar graph. Black lines indicate significant difference (p<0.05 for Welch's t-test) between experimental and negative control (*ars1238*Δ) strains. Culture conditions as described in Figure 2B. (D) Preferential re-initiation of *ARS1238* does not require forkhead proteins. Left panel: re-replication profiles of Chromosome XII containing *ARS1238* at its endogenous location (gray arrow). Profiles for positive (WT, YJL8398, n = 10) and negative (*ars1238*Δ, YJL9152, n = 7) control strains are overlaid with *fkh*Δ strains described in Figure 2D. Right panel: DNA copy number at the endogenous *ARS1238* locus (ChrXII_889 kb) plotted as a bar graph. Black lines indicate significant difference (p<0.05 for Welch's t-test) between experimental and negative control (*ars1238*Δ) strains. Culture conditions as described in Figure 2D.
(EPS)

Figure S3 Preferential re-initiation of *ARS317* does not require a nearby cryptic origin. Mutations that disrupt the cryptic origin close to *ARS317* (*HMRE-A*, YJL8526) or the OBS of *ARS317* (*HMRE-E*, YJL8538) (see [36]) were introduced into the preferentially re-initiating fragment *317(+167..-105)* and integrated at ChrIV_567 kb of an *MC2Ao* strain. Re-replication profiles of Chromosome IV were generated and displayed as in Figure 2, except the mean of two profiles was obtained from duplicate experiments on individual mutant strains and not isogenic strain pairs.
(EPS)

Figure S4 Mapping re-initiation promoters for *ARS317* and *ARS1238*. (A) Partial disruption of preferential re-initiation of *ARS317* by linkers L9, L11, and L15 is additive. Combinations of linker mutation L9, L11, and L15 were introduced into the preferentially re-initiating fragment *317(+153..-106)* and integrated at ChrIV_567 kb of an *MC2Ao* strain (gray bars). As controls L9, L11, and L15 were combined with linkers L13 or L17, which have no effect on preferential re-initiation (black and gray bars), and linkers with no effect were combined with each other (L13 L17, L21 L23; black bars). Re-initiation efficiencies were obtained as described for Figure 4B. Strains used are listed in Table S2. (B) Sequences from nucleotide +153 to +87 are sufficient to confer preferential re-initiation on *ARS317*. Sequences covered by linkers L17 to L31 (nucleotides +86 to +23), each of which alone had little or no effect on preferential re-initiation of ARS317, were replaced by randomly generated sequence of similar AT content (25%) in the preferentially re-initiating fragment *317(+153..−106)* fragment. The resulting clone was integrated at ChrIV_567 kb in *MC2Ao* strain YJL8838 (top panel). A similar strain YJL9713 (bottom panel) was generated that in addition had nucleotides +153 to +135 (covering linkers L1 to L3) replaced. Re-replication profiles of Chromosome IV were generated and displayed as in Figure 2, except for each mutant strain, the mean of two profiles was obtained from duplicate experiments on the single strain. (C) Structure of Re-Initiation Promoter for *ARS1238*. A series of linker substitution mutations (A–H) constructed with an 8 bp GGGATCCG linker were introduced into the segment adjacent to the *ARS1238* origin in the preferentially re-initiating fragment *1238(+133..−100)* and integrated at ChrIV_567 kb of an *MC2Ao* strain. The mutant fragments were assayed for re-initiation efficiency as described in Figure 5B. Wild type sequence is shown beneath graph. Sequence of linker mutations are represented by letters for changed nucleotides and dashes for unchanged nucleotides. Isogenic strain pairs used for each linker substitution are listed in Table S2.
(EPS)

Figure S5 *RIP317* AT-content is not sufficient for re-initiation. Re-initiation efficiency for *317(+153..L17..−106)* fragments with mutations in region *317(+134..+109)* that preserve AT-content while altering sequence. Wild type sequence of *RIP317* (nucleotides +153..+87) is shown beneath graph. Mutated sequence is represented by letters for changed nucleotides and dashes for unchanged nucleotides. Mutant fragments were assayed for re-initiation efficiency as described in Figure 5B, except that normalization was performed against the mean peak height for the full length *317(+153..−106)* fragment in reference strains YJL8398 and YJL8399. Isogenic strain pairs used for each mutation are listed in Table S2.
(EPS)

Figure S6 Susceptibility of additional origins to RIP function. (A) Hybrid *RIP317-ARS1238* fusion fragments re-initiate, but not as efficiently as the endogenous *RIP317-ARS317* fragment. *RIP317* was fused to *ARS1238* while maintaining the spacing between *RIP* and *OBS* that normally occurs between *RIP317* and *ARS317* (53 nt). The fragment was integrated at ChrIV_567 kb in isogenic *MC2Ao* strains YJL10160 and YJL10161 and a re-replication profile for Chromosome IV was generated and displayed as in Figure 1. (B) *RIP317* does not confer significant re-replication on all origins. *RIP317* was fused to the following origin fragments and the fusion constructs integrated at ChrIV_567 kb in the *MC2Ao* strain background. For each origin, nucleotide boundaries, *RIP-OBS* spacing, and yeast strain analyzed are indicated in parentheses. Nucleotide numbering is based on +1 to +33 for the T-rich strand of the OBS. *ARS209* (nt +91..−241, 58, YJL9088), *ARS305* (nt +83..−249, 50, YJL9082), *ARS306* (nt +89..−245, 56, YJL9084), *ARS702* (+84..−247, 51, YJL9086). Re-replication profiles of Chromosome IV were generated and displayed as in Figure 2 except the mean of two profiles was obtained from duplicate experiments on each individual mutant strain. Re-replication was induced for 3 hr (black borders) or 6 hr (red border). (C) Mitotic stability of origin fragments fused to *RIP317*. The *ARS* activity of the origin fragments described in B was assayed by mitotic stability in plasmids containing *CEN4* and *URA3*. *ARS317* (pCR339), *ARS1238* (pCR321), *ARS1021* (pCR146), *ARS301* (pCR147), *ARS209* (pCR154), *ARS305* (pCR149), *ARS306* (pCR150), or *ARS702* (pCR152) were assayed for mitotic stability as described in Figure 3B.
(EPS)

Table S1 Description of integrative and mitotic stability plasmids used in this manuscript. Each entry lists plasmid name, key plasmid features, and the sequence of the *RIP*-origin fragment inserted into XbaI (Integrative *RIP*-origin plasmids; pBJL2889 derived [2]) or HindIII-EcoRI (mitotic stability; pFJ11 derived [36]) restriction sites. See Materials and Methods for a complete description of plasmid construction.
(XLSX)

Table S2 Description of all yeast strains used in this publication. Yeast strain numbers are presented with: 1) the genotype for each strain, and 2) the plasmid used to integrate the re-replication cassette at ChrIV_567 kb. Sequence of the *RIP*-origin region of the re-replication cassette plasmid can be found in Table S1.
(XLSX)

Table S3 Primers used in strain generation. Transcription factors and origin DNA were disrupted by one-step gene replacement. The primers, targets, sequences, and template DNA used for PCR amplification of these disruption fragments are listed along with the marker used (in parentheses).
(XLSX)

Table S4 Primers used in qPCR analysis. Primer names, targets, and sequences are listed.
(XLSX)

Document S1 Raw normalized data (red dots) and smoothed line (black line) used to generate composite profiles or percent re-replication efficiency for all the other figures of this manuscript.
(PDF)
