# Supplementary material for: Regulatory Mechanisms That Prevent Re-initiation of DNA Replication Can Be Locally Modulated at Origins by Nearby Sequence Elements
Source: PLoS Genet. 2014 Jun 19;10(6):e1004358. doi: 10.1371/journal.pgen.1004358 (PMC4063666; doi:10.1371/journal.pgen.1004358)

Figure 1A

YJL3758 Chr_III

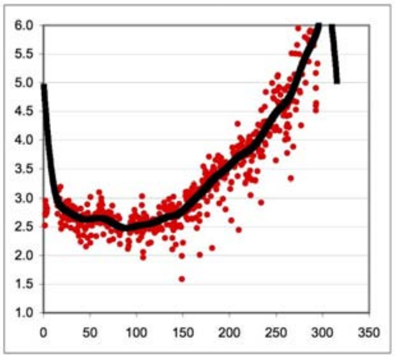

YJL3758 Chr_IV

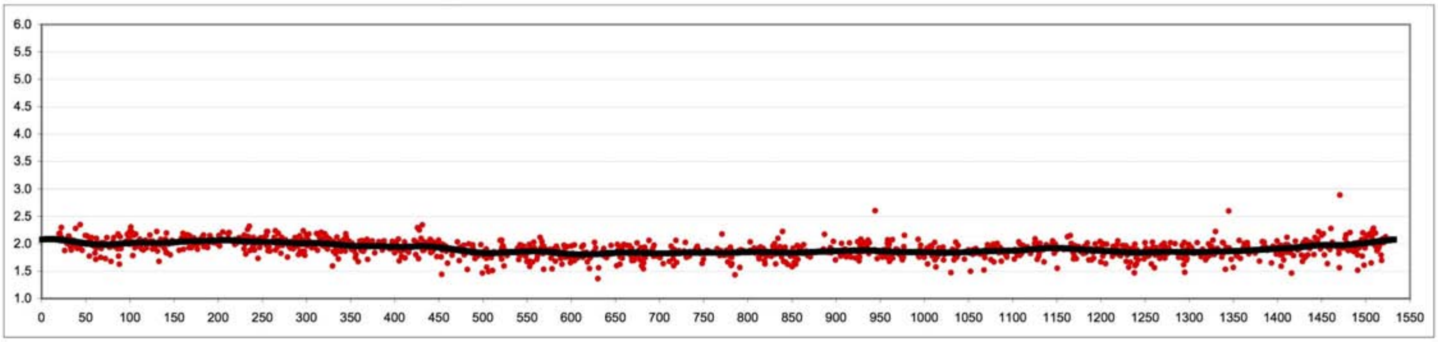

YJL3758 Chr_V

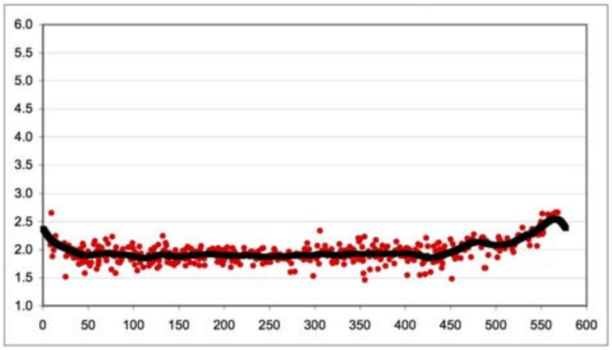

YJL3758 Chr_XII

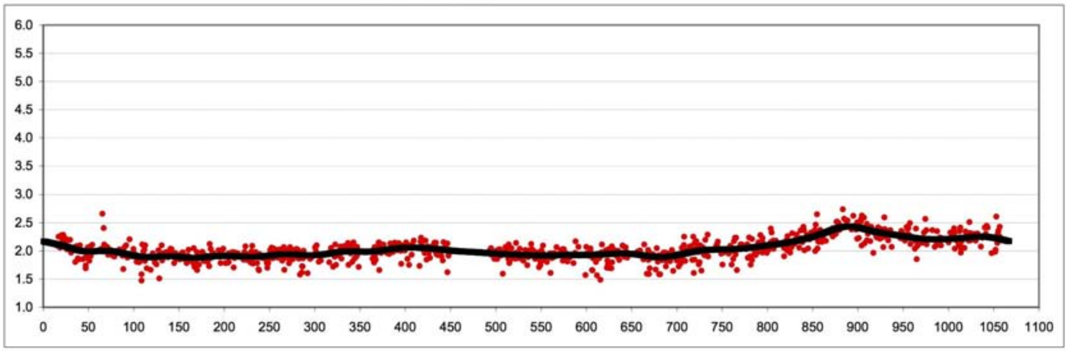

YJL3759 Chr_III

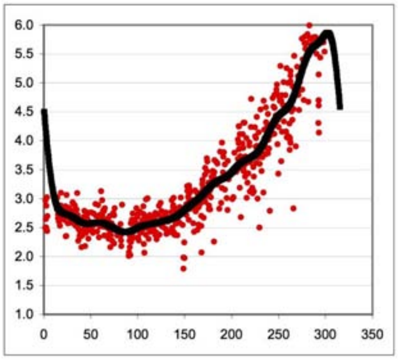

YJL3759 Chr_IV

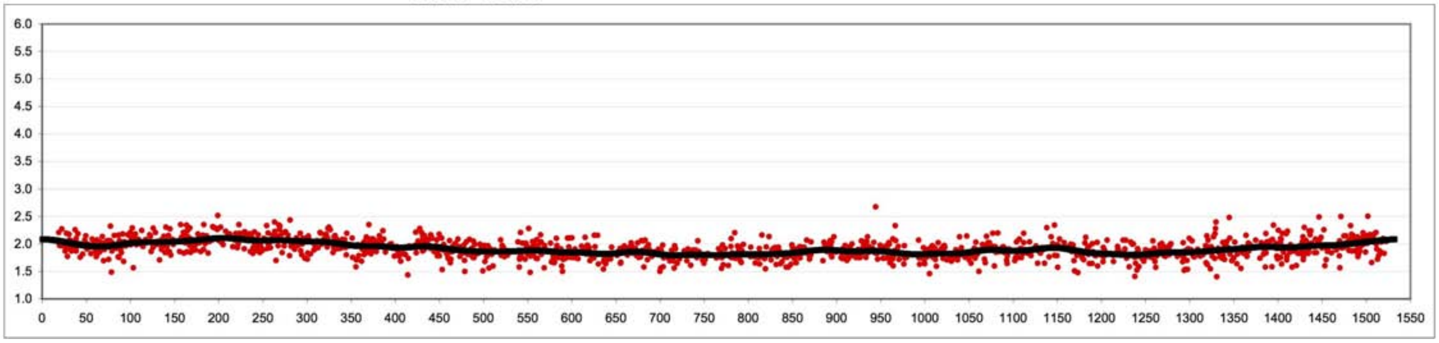

YJL3759 Chr_V

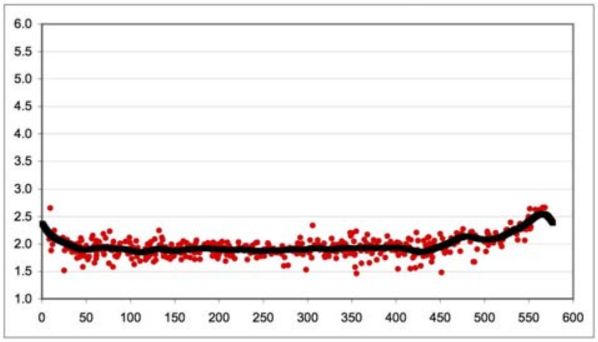

YJL3759 Chr_XII

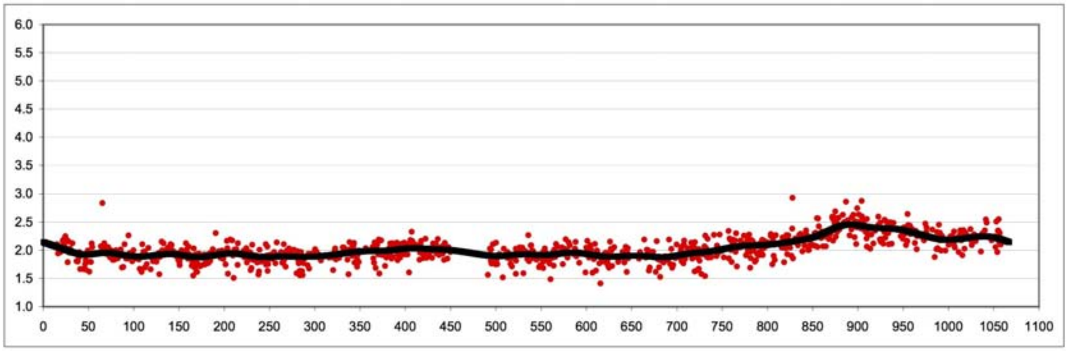

Figure 1B
YJL9152 Chr_XII

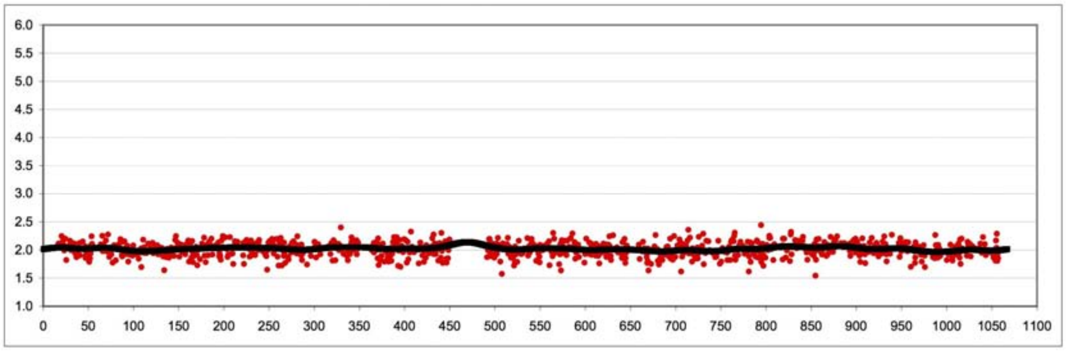

YJL9152 Chr_XII

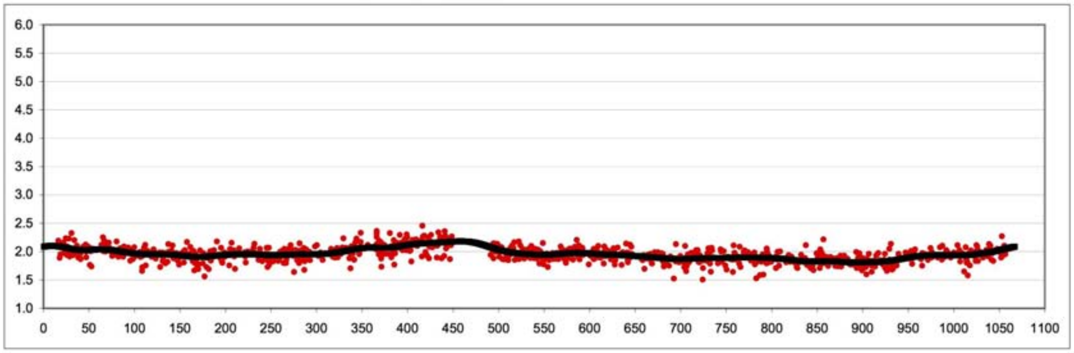

Figure 2A
YJL7700

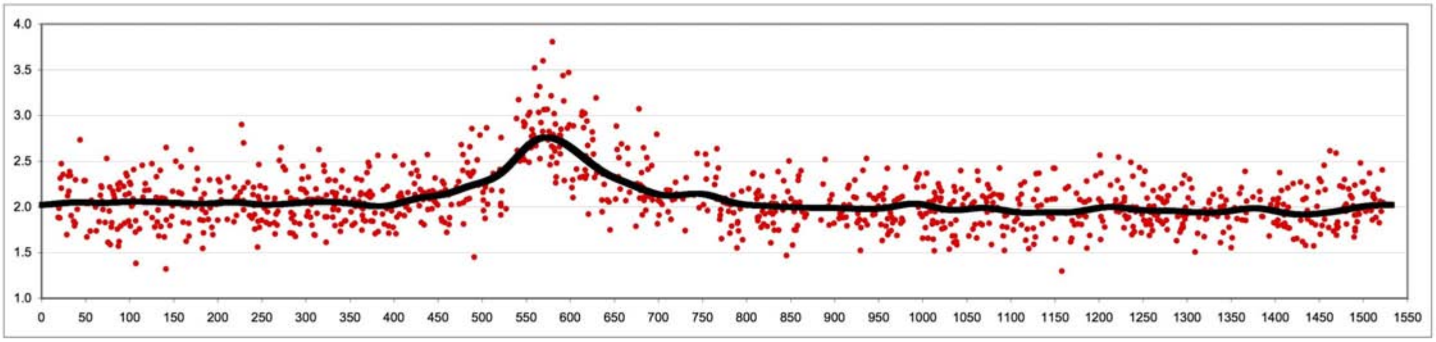

YJL7701

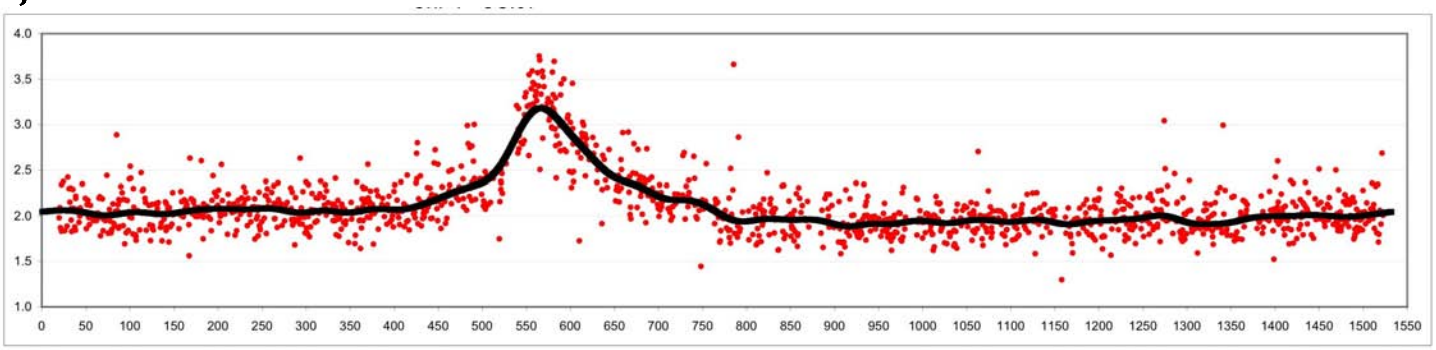

YJL8256

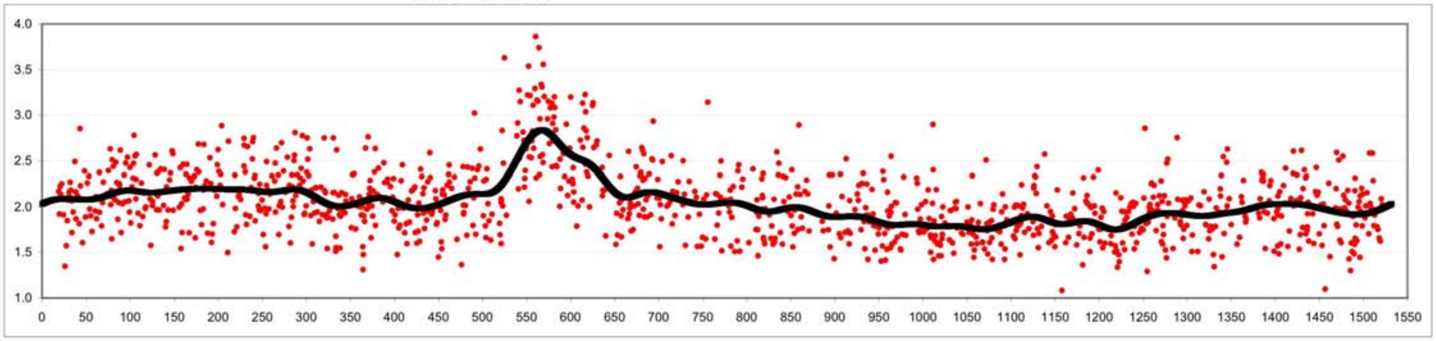

YJL8257

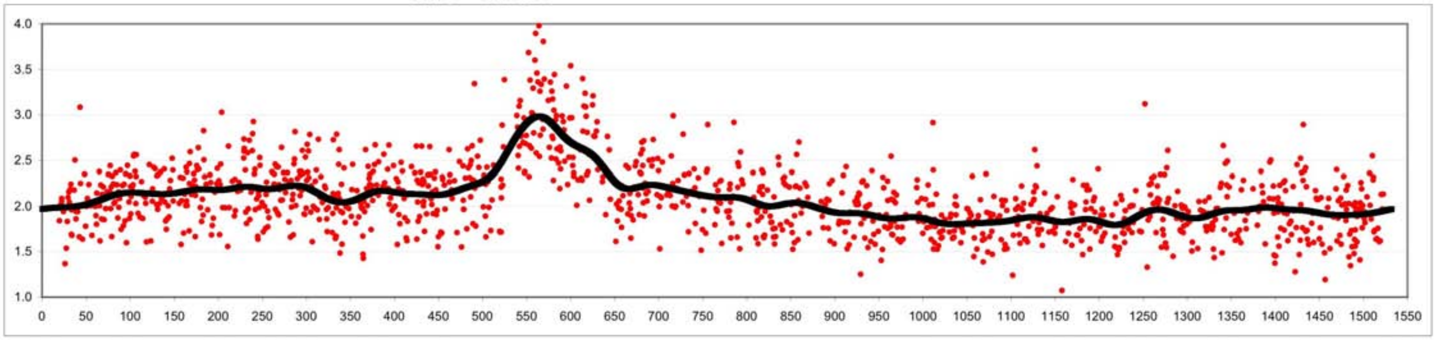

Figure 2B

YJL3758 – positive control

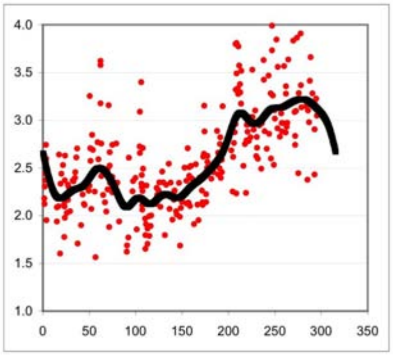

YJL3758 – positive control

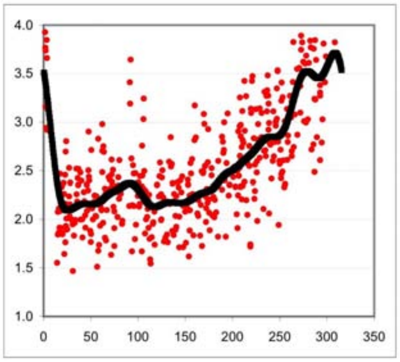

YJL3758 – positive control

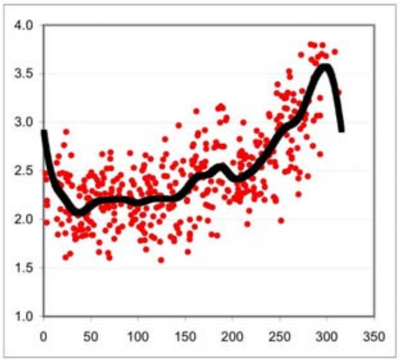

YJL3758 – positive control

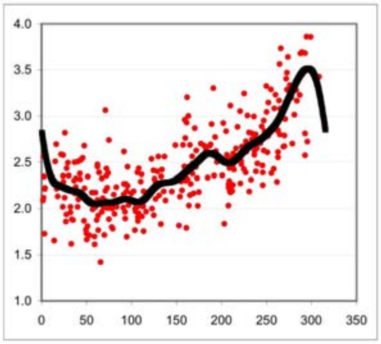

YJL3758 – positive control

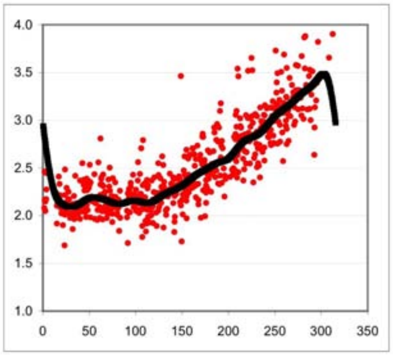

YJL8398 – negative control

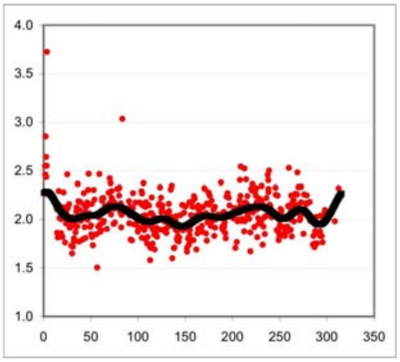

YJL8398 – negative control

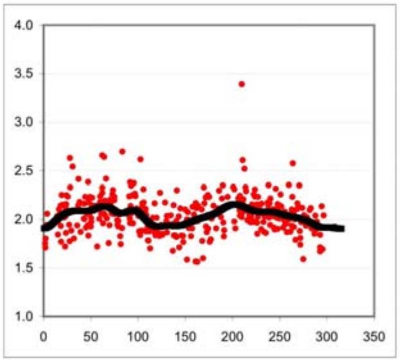

YJL8398 – negative control

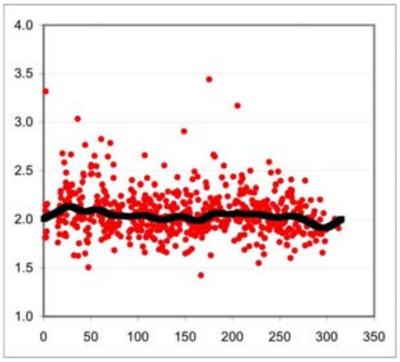

YJL8398 – negative control

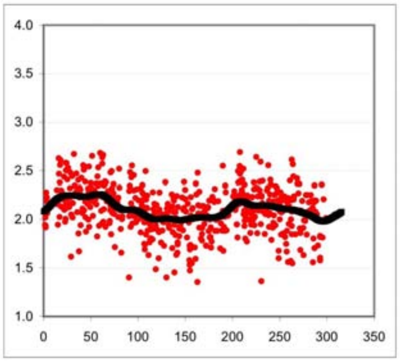

YJL8398 – negative control

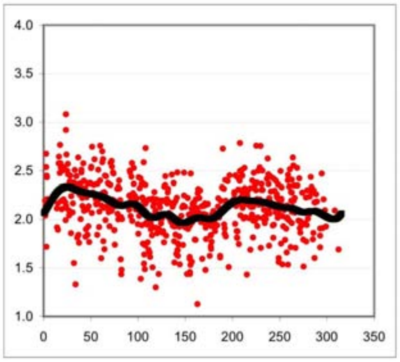

YJL8398 – negative control

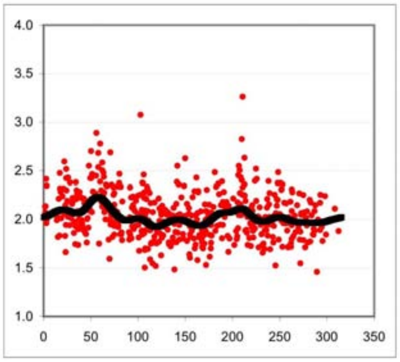

YJL8398 – negative control

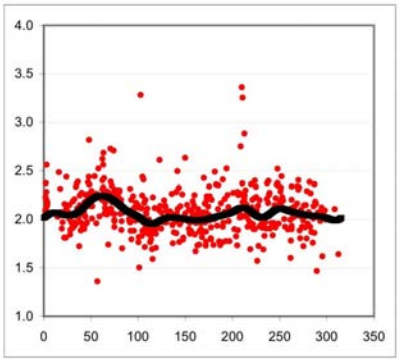

YJL8398 – negative control

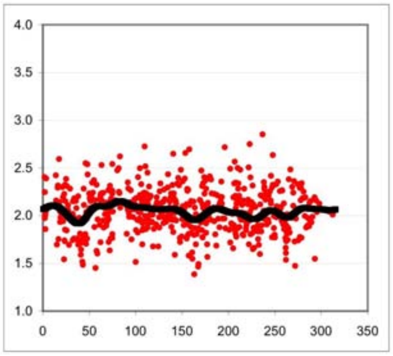

YJL8398 – negative control

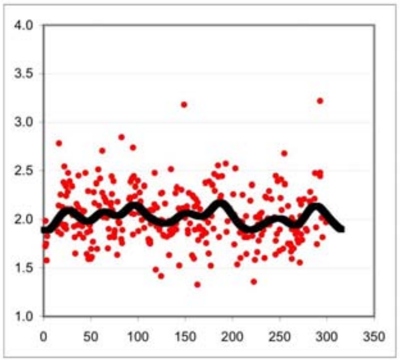

YJL8398 – negative control

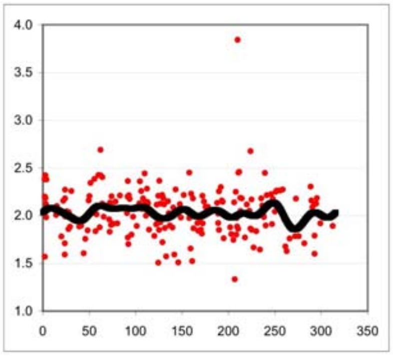

YJL6893

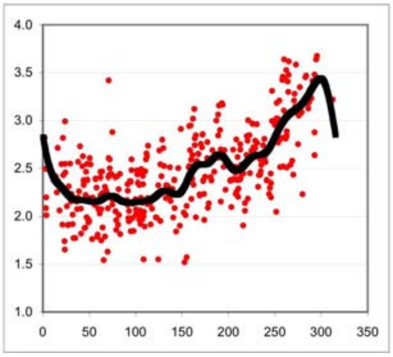

YJL6894

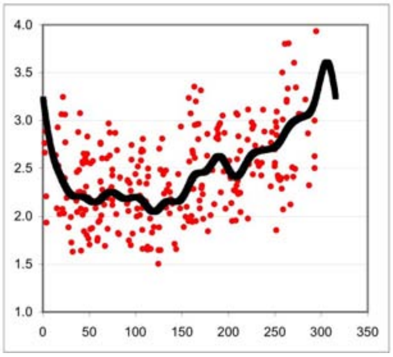

YJL6896

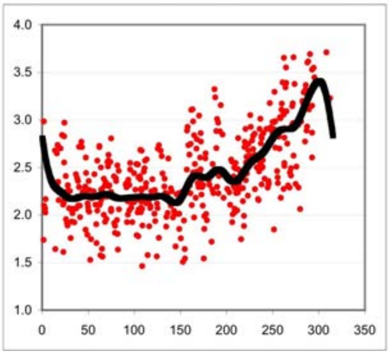

YJL6897

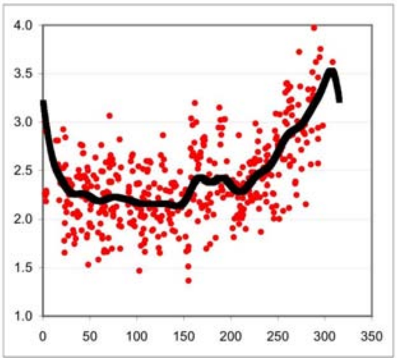

YJL6899

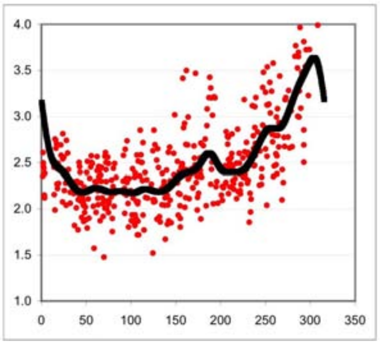

YJL6900

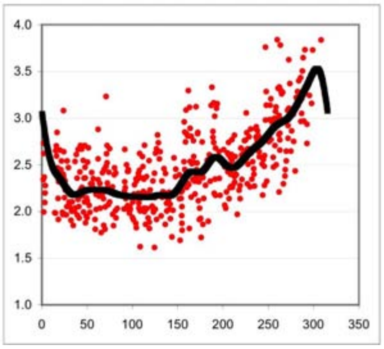

YJL6902

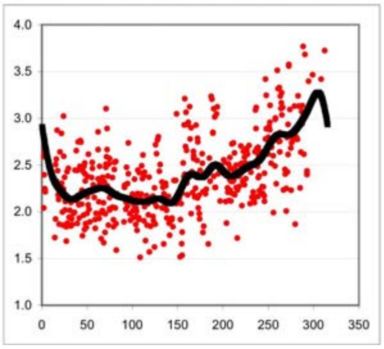

YJL6903

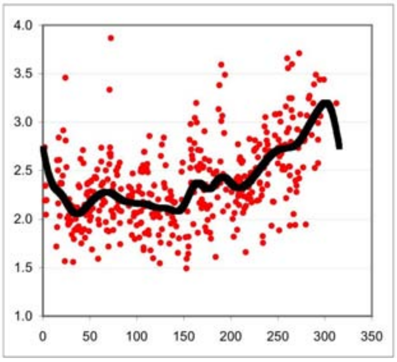

Figure 2C
YJL8256

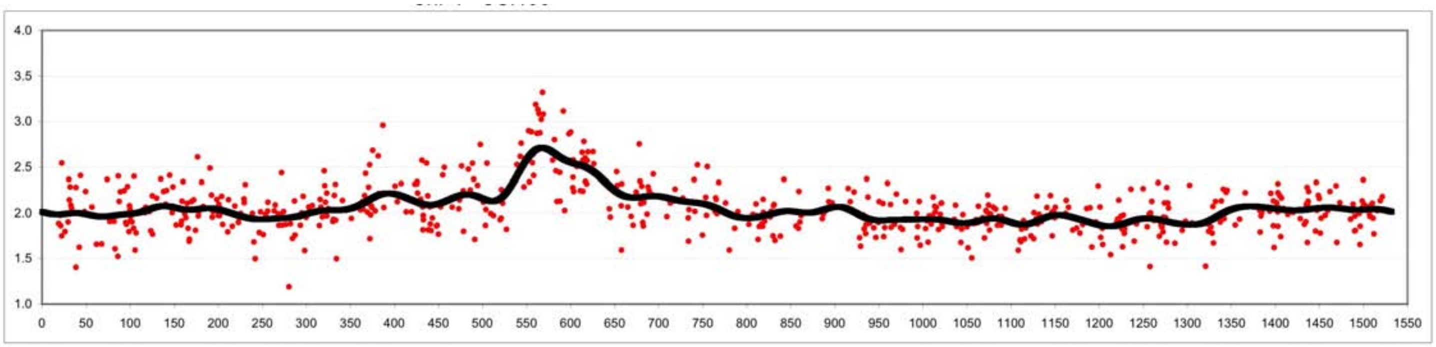

YJL8257

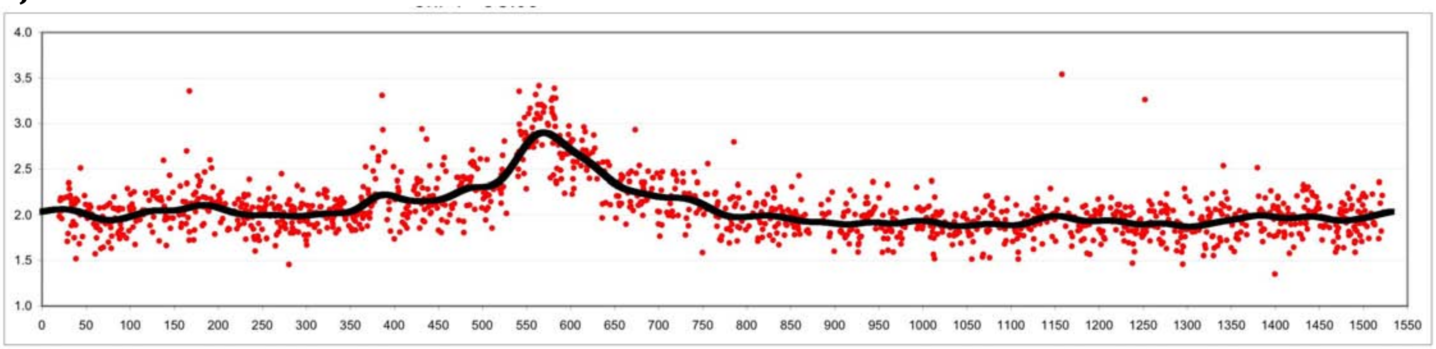

Figure 2D
YJL8398 – Positive Control

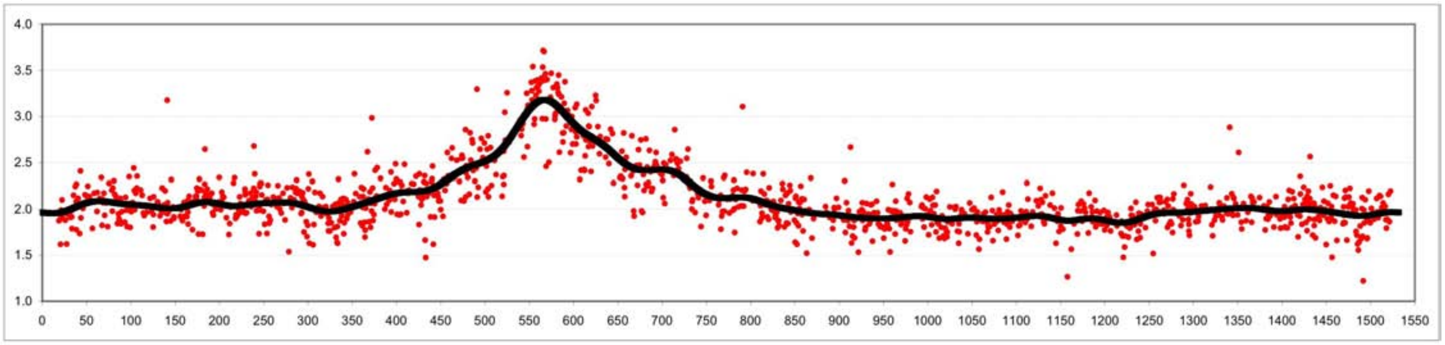

YJL8398 – Positive Control

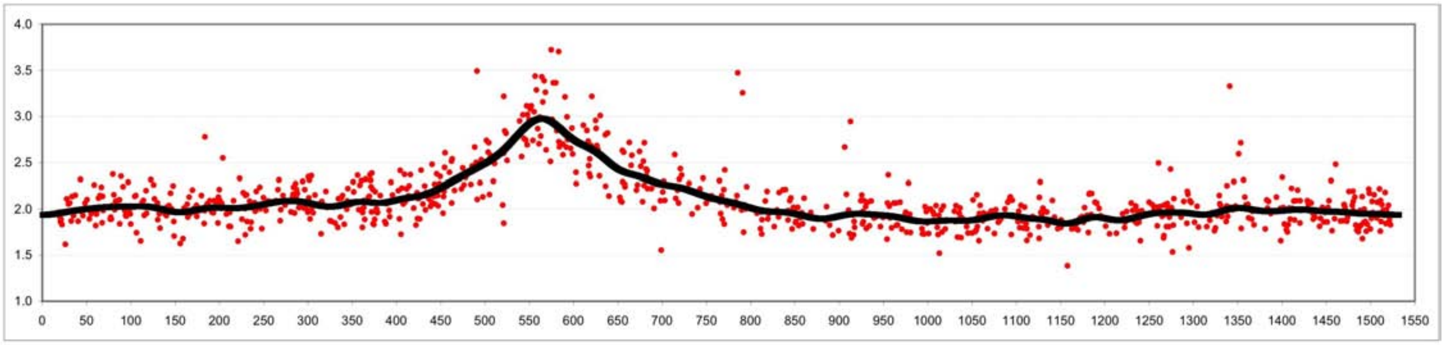

YJL8398 – Positive Control

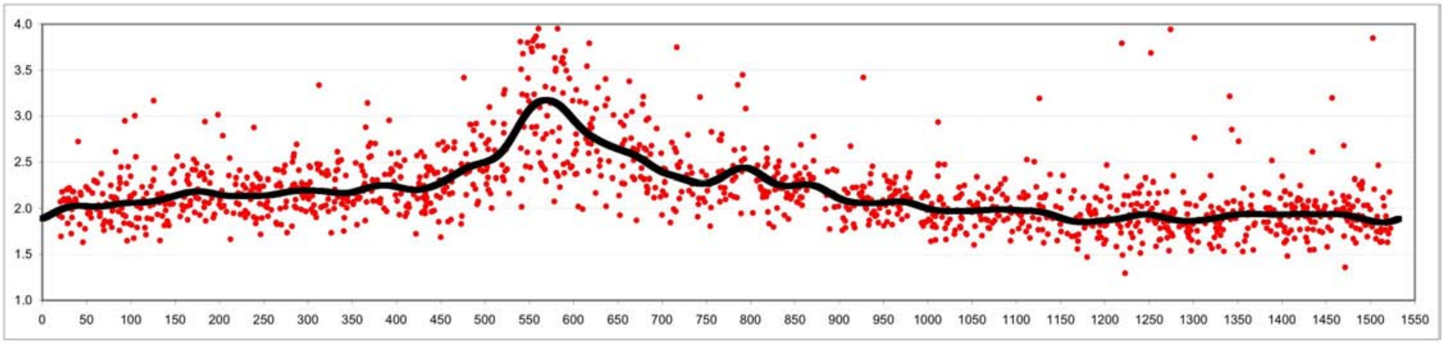

YJL8398 – Positive Control

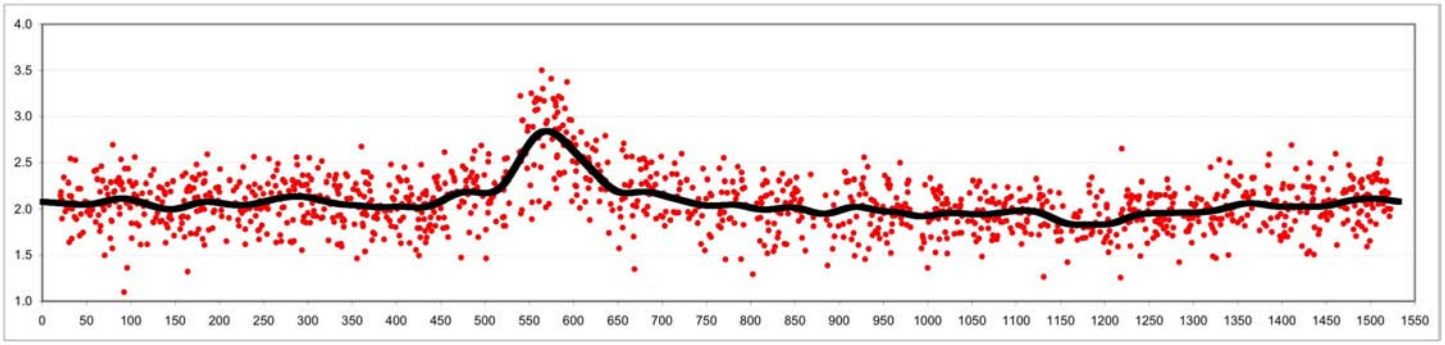

YJL8398 – Positive Control

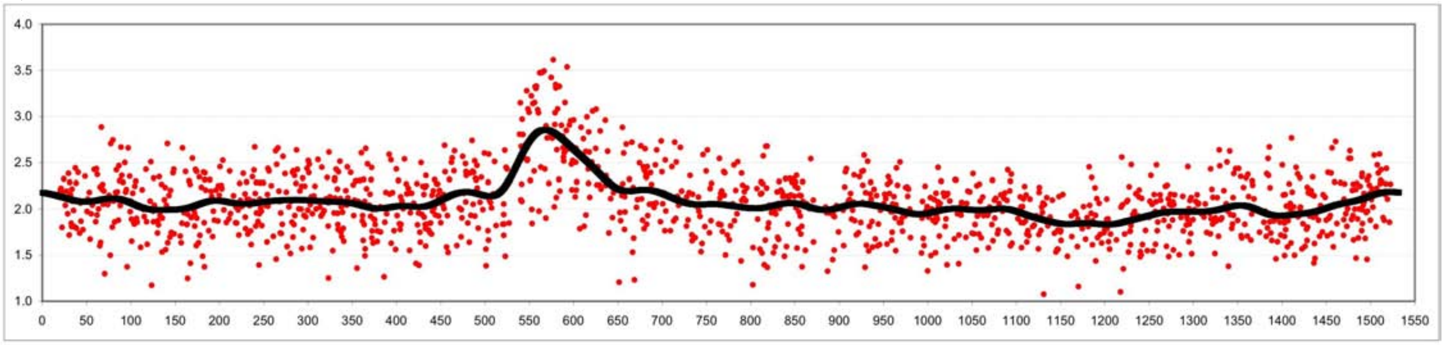

YJL8398 – Positive Control

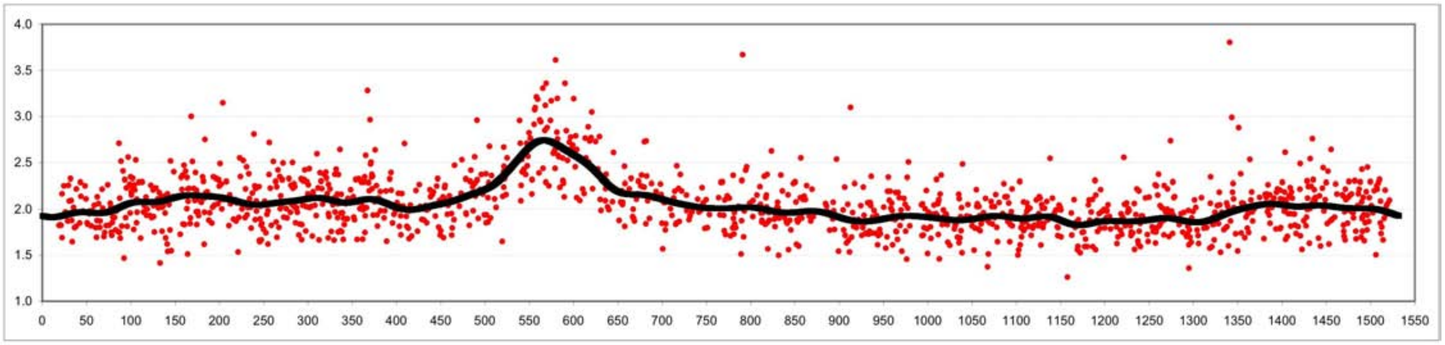

YJL8398 – Positive Control

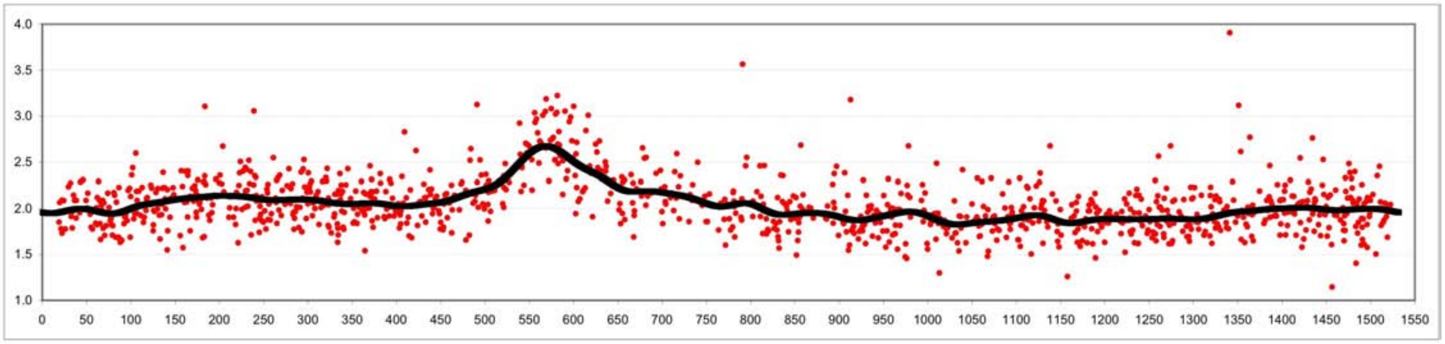

YJL8398 – Positive Control

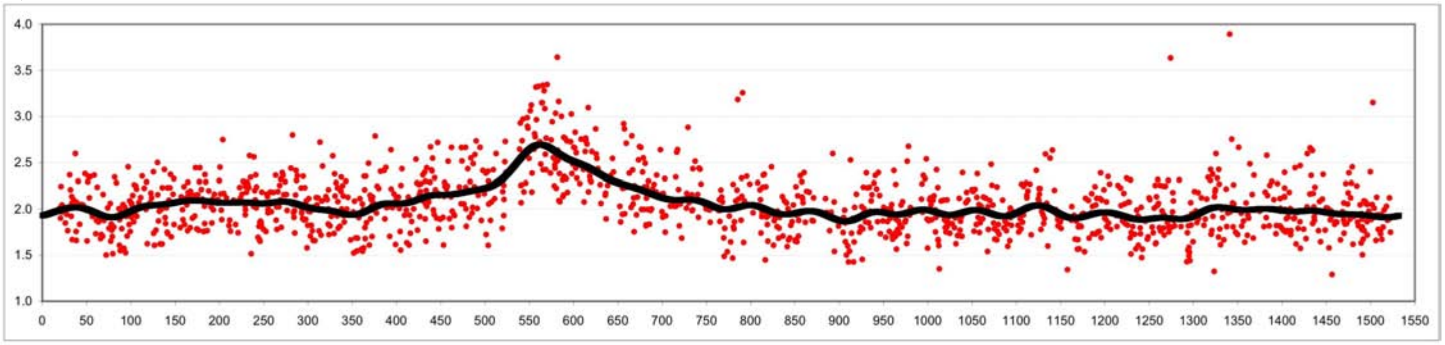

YJL8398 – Positive Control

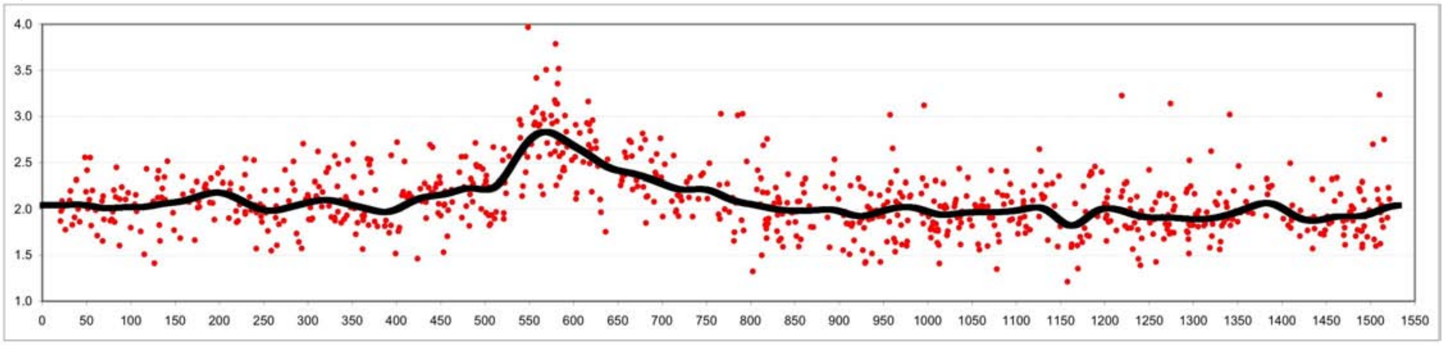

YJL8398 – Positive Control

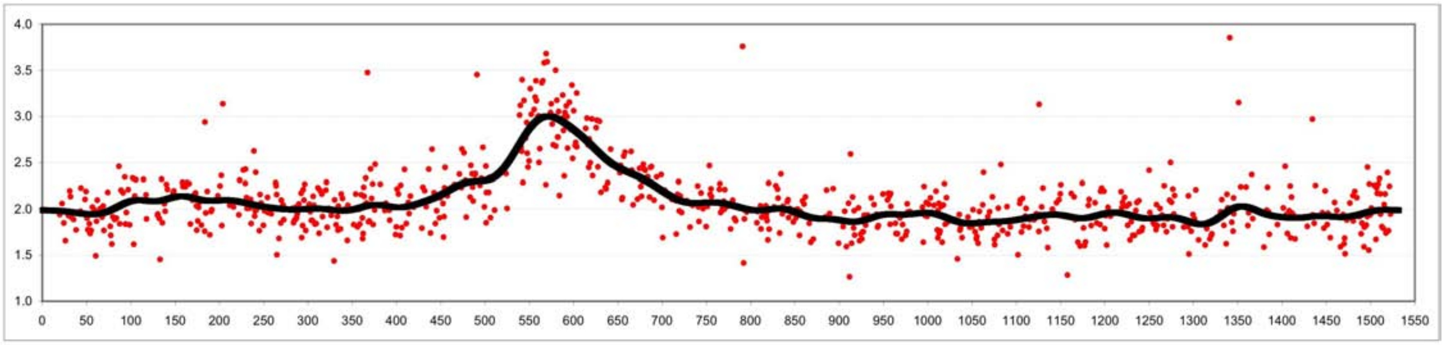

YJL3758 – Negative Control

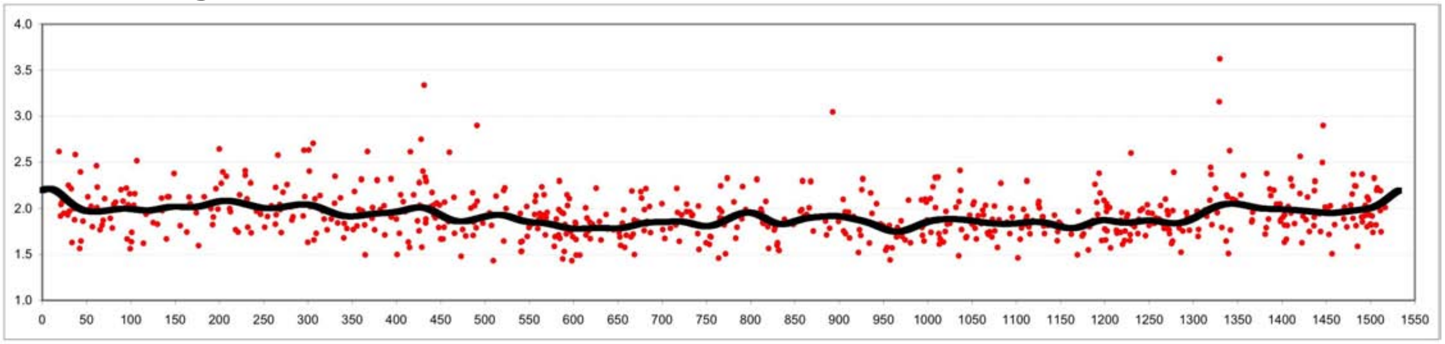

YJL3758 – Negative Control

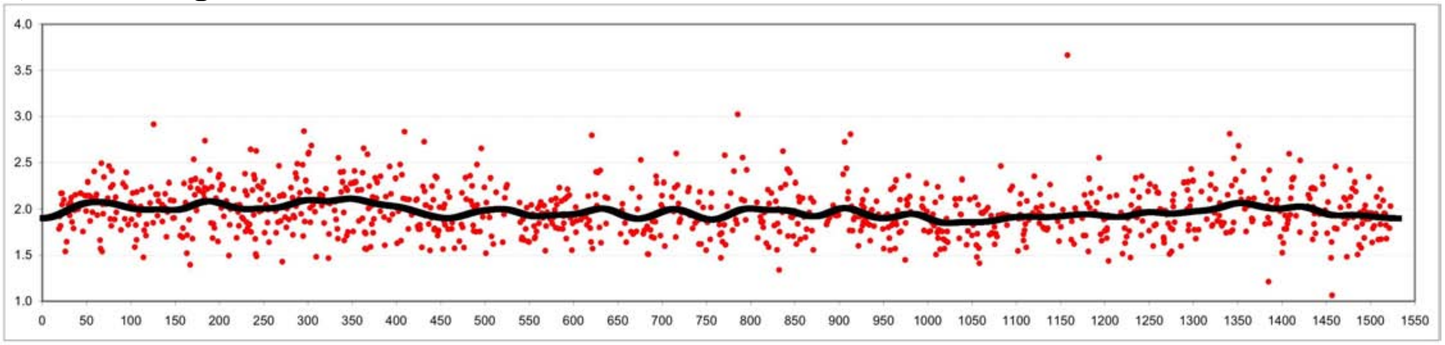

YJL3758 – Negative Control

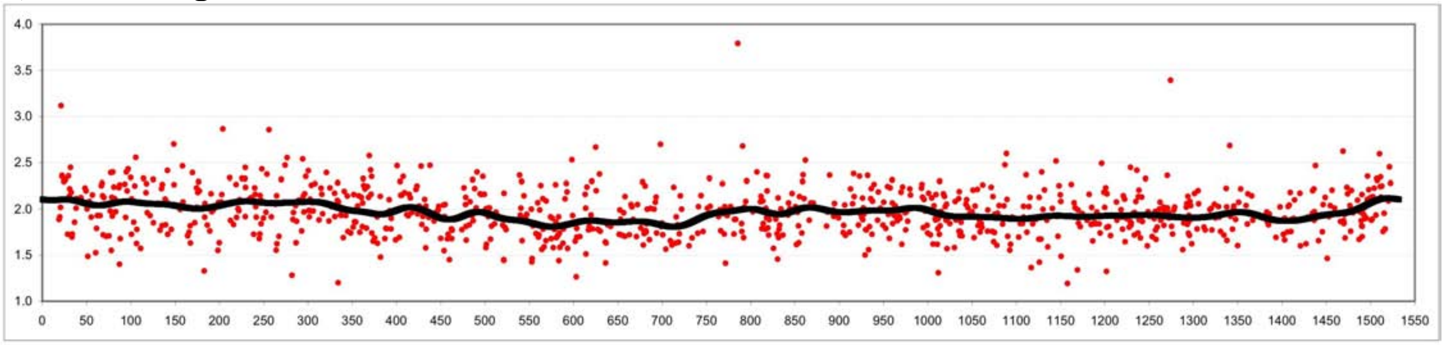

YJL3758 – Negative Control

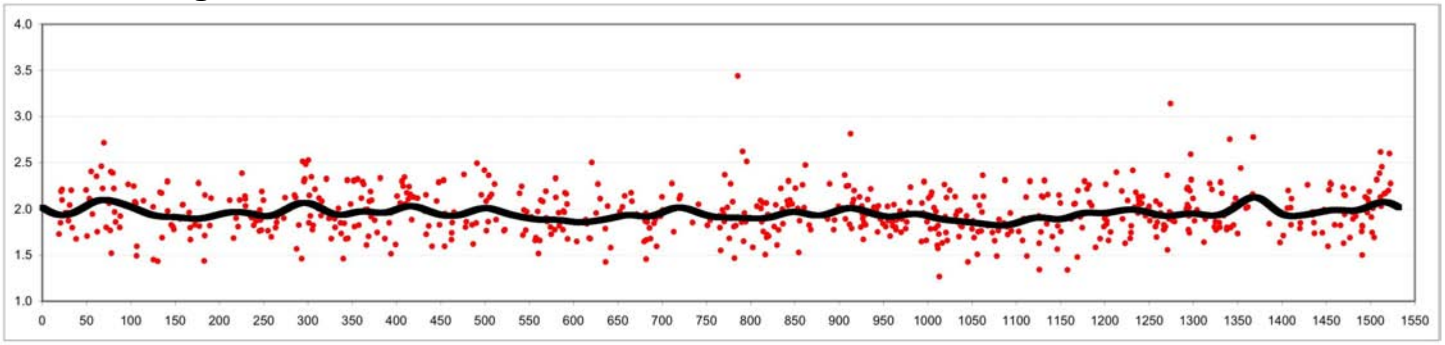

YJL3758 – Negative Control

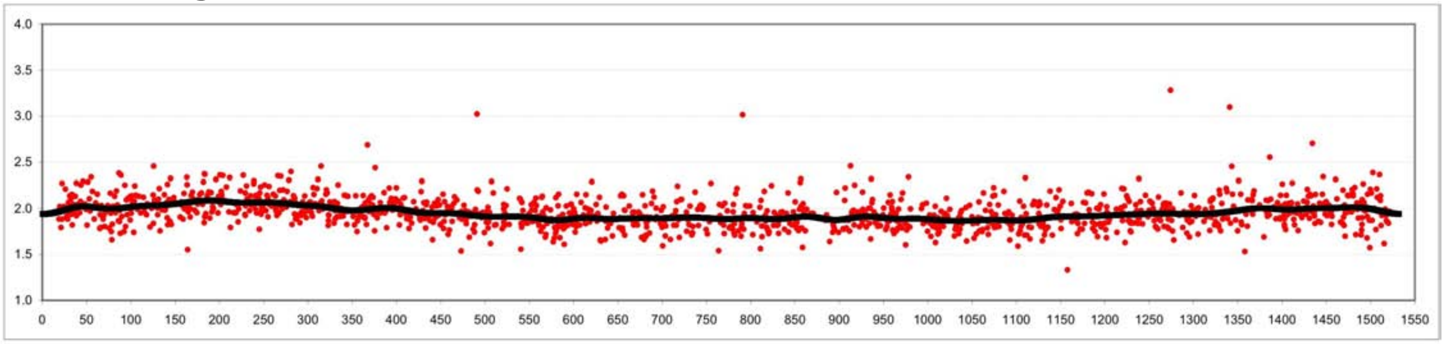

YJL8701

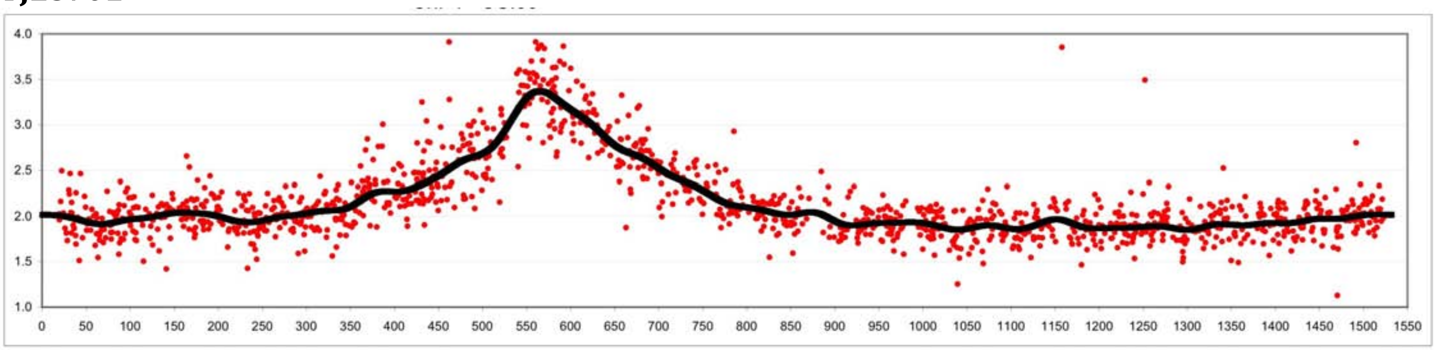

YJL8702

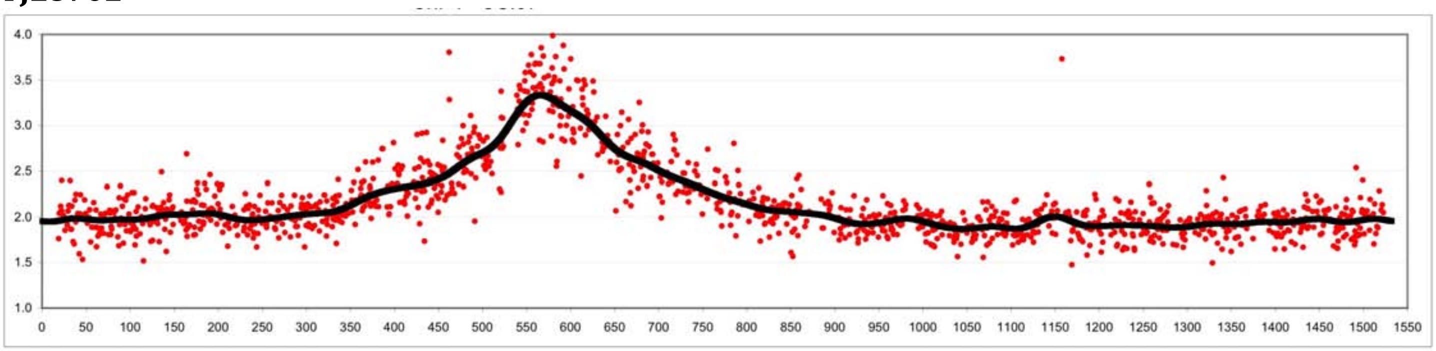

YJL8745

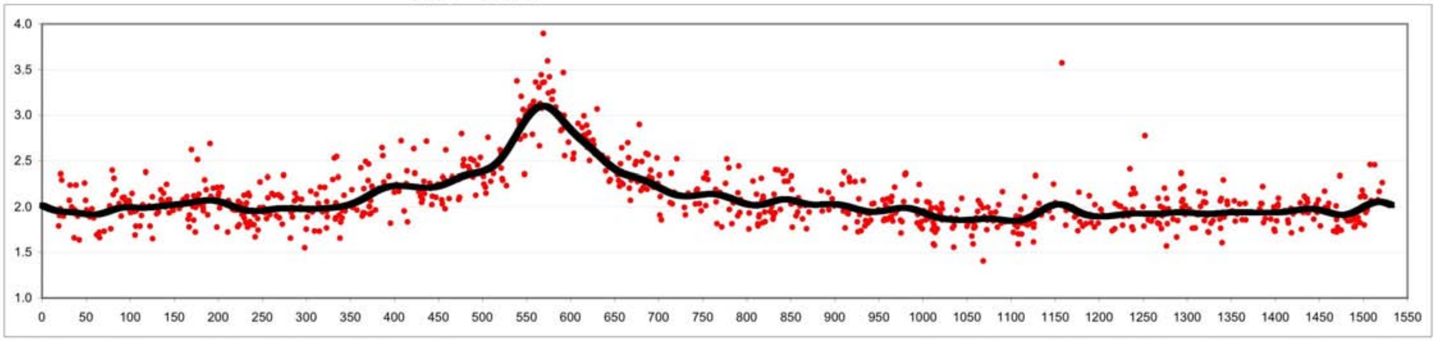

YJL8746

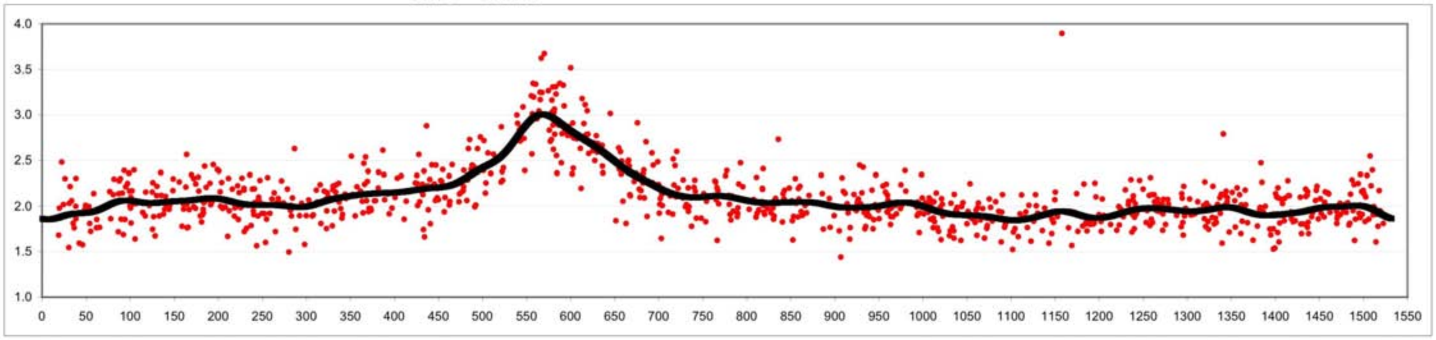

YJL8749

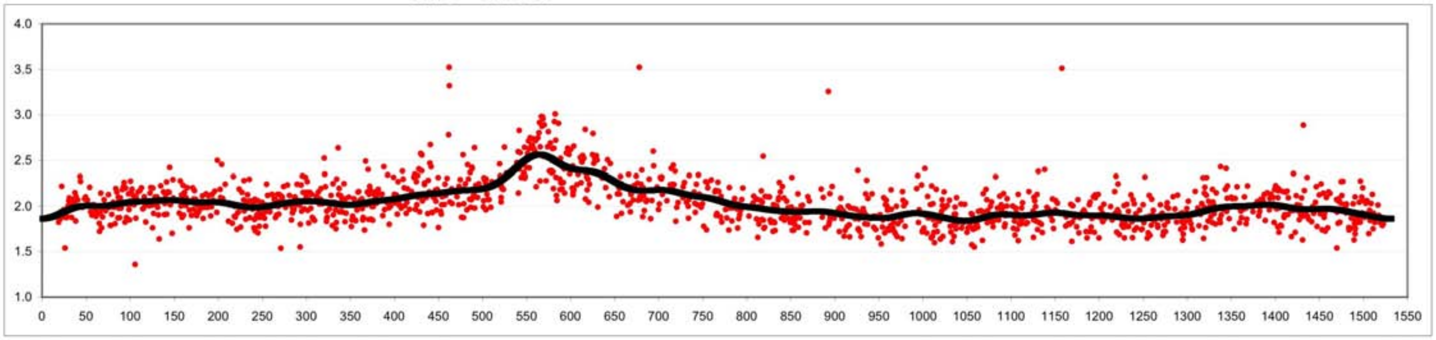

YJL8750

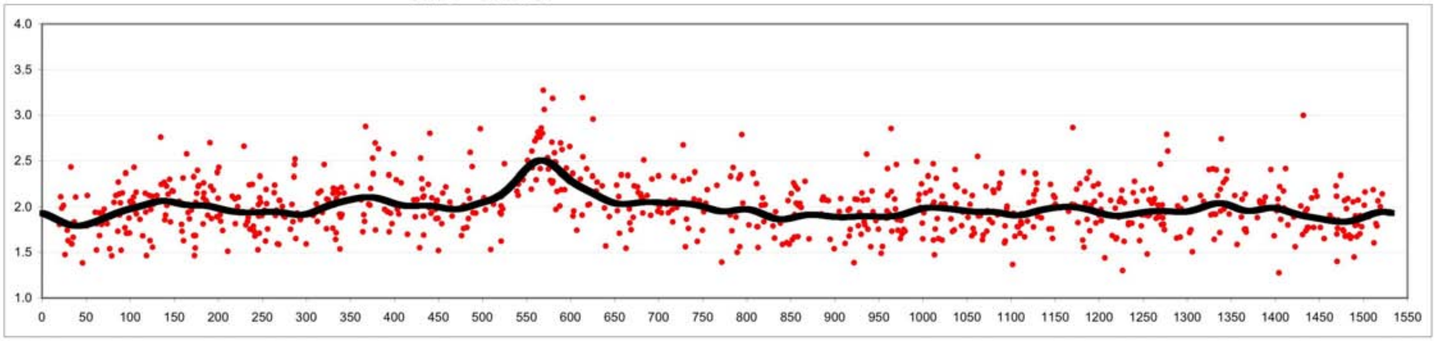

Figure 3C
YJL10444

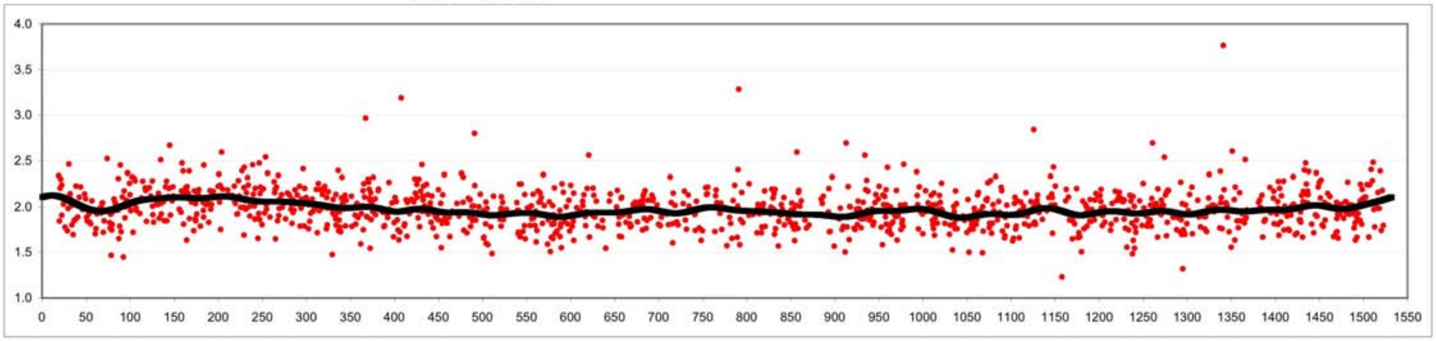

YJL10445

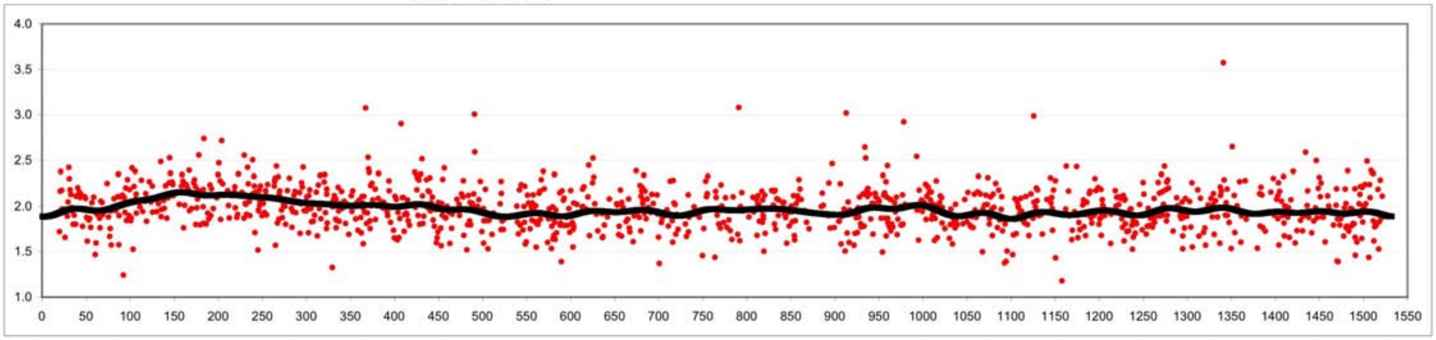

Figure 3D
YJL7717

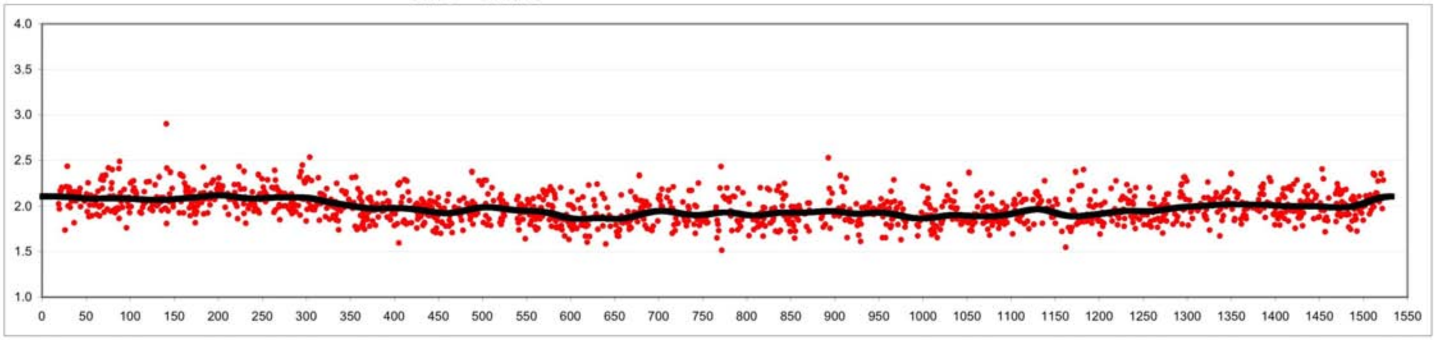

YJL7717

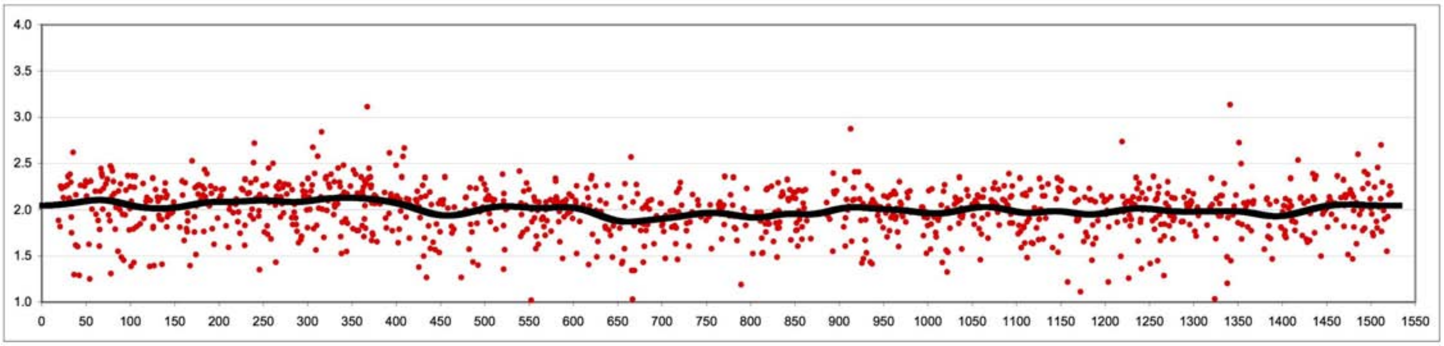

Figure 3E
YJL9707

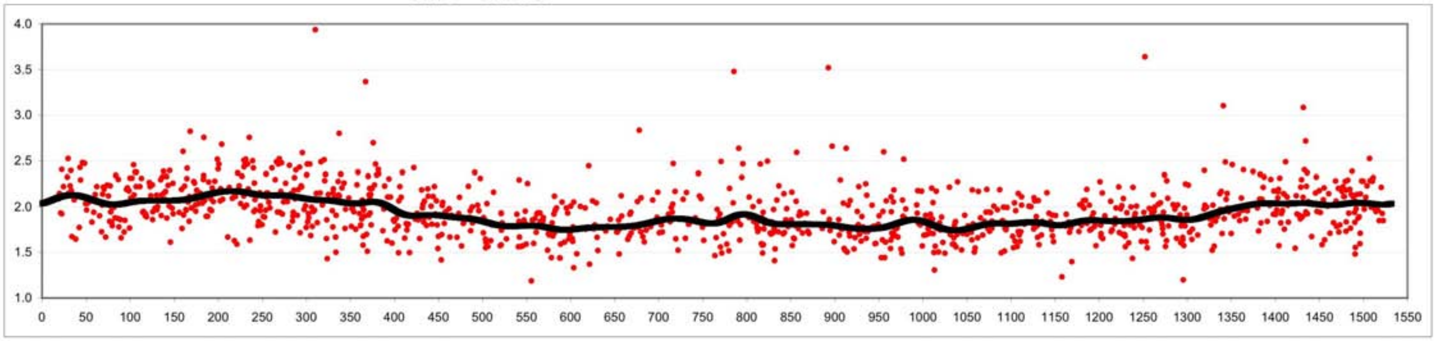

YJL9708

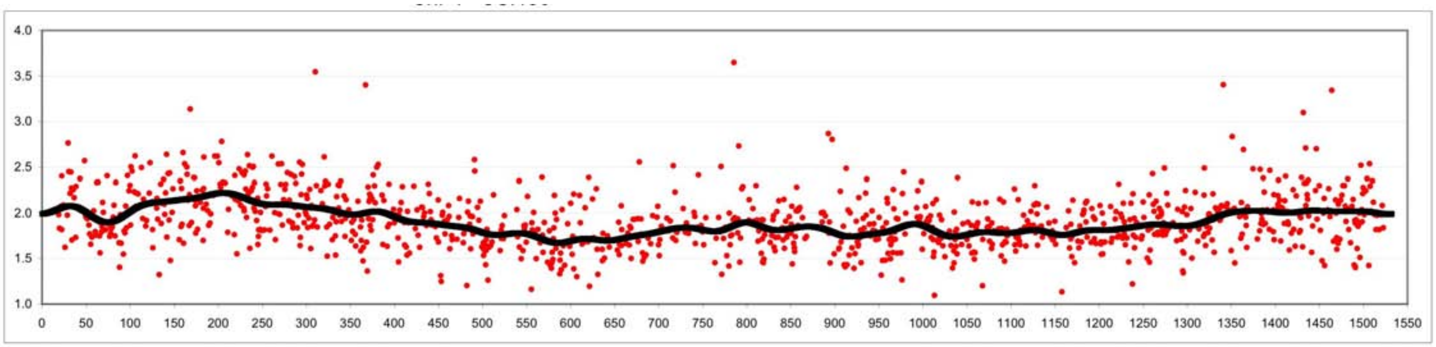

Figure 4A
YJL7700 – control

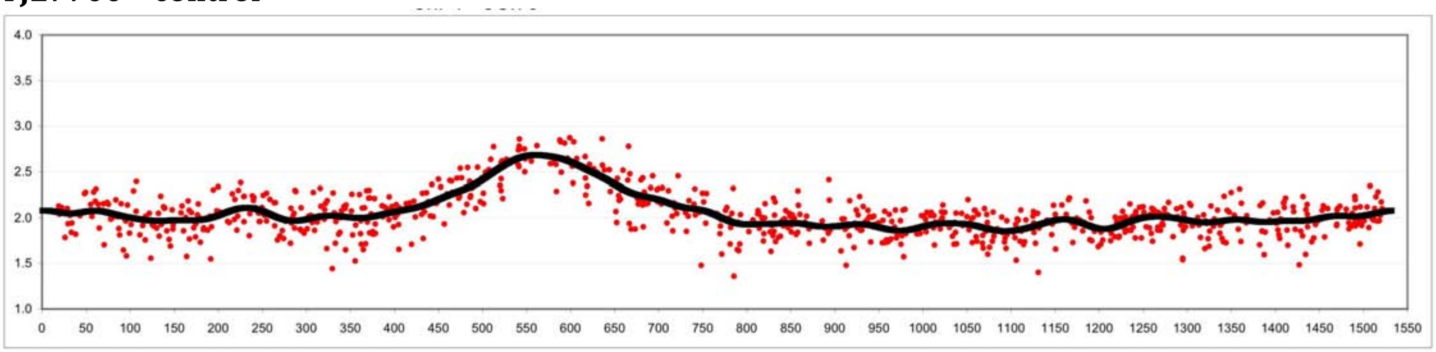

YJL7701 – control

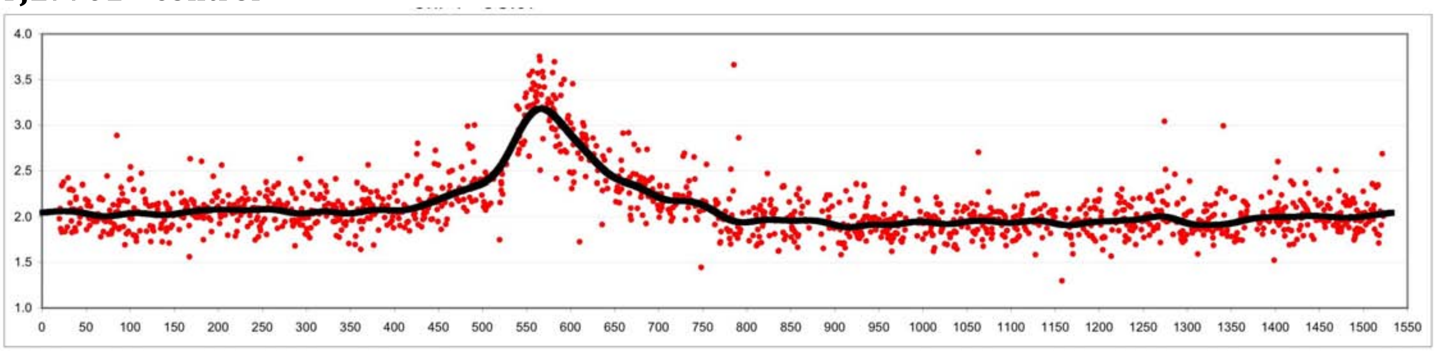

YJL8386

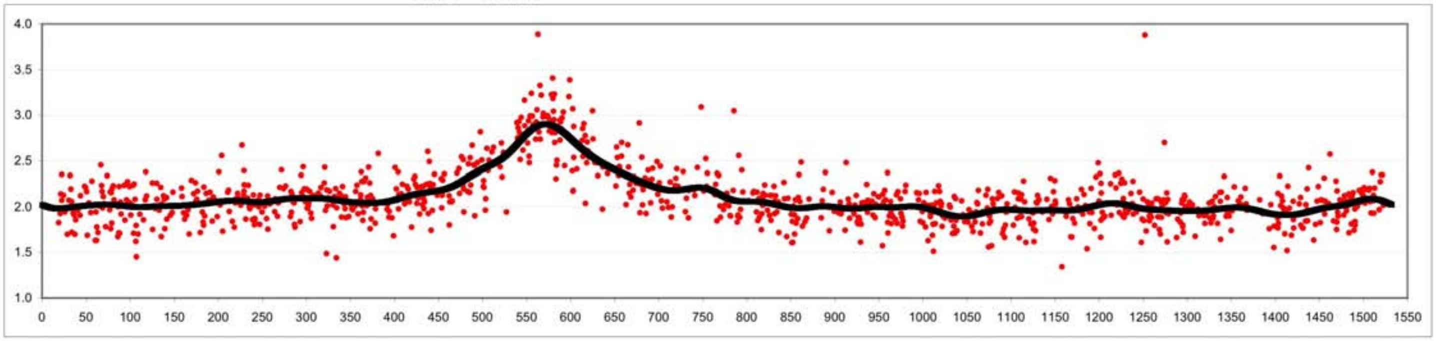

YJL8387

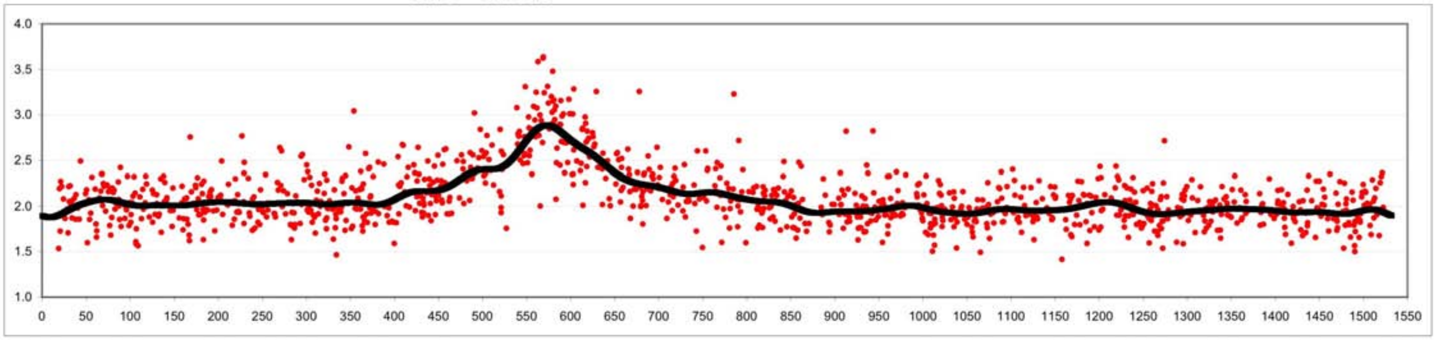

YJL8392

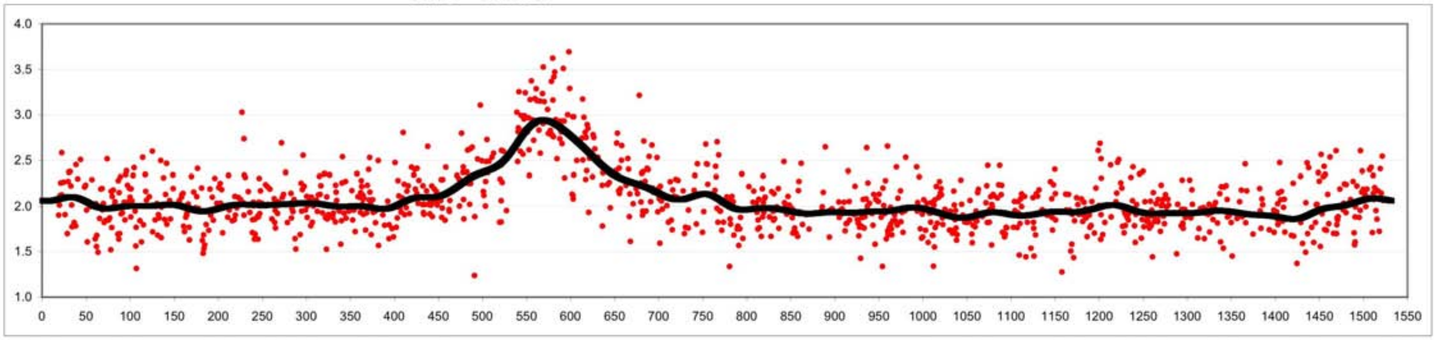

YJL8393

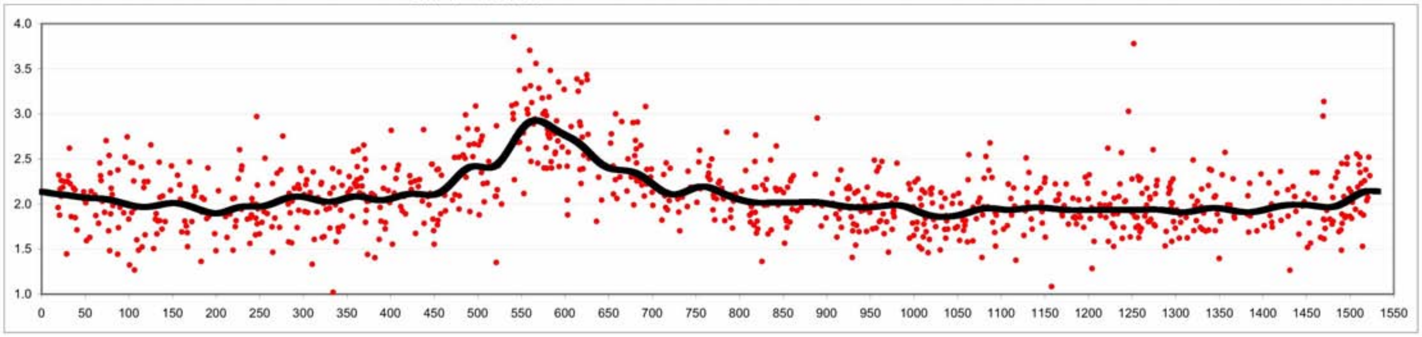

YJL8398

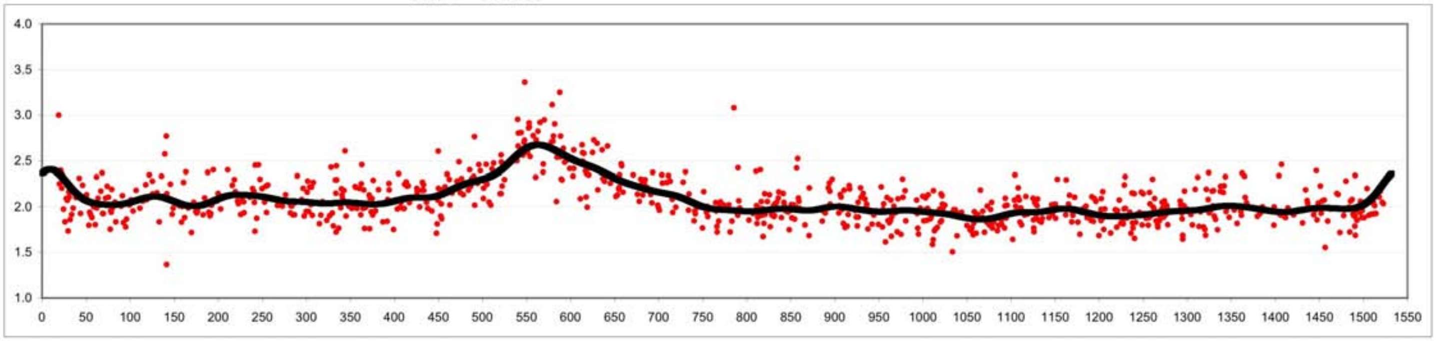

YJL8398

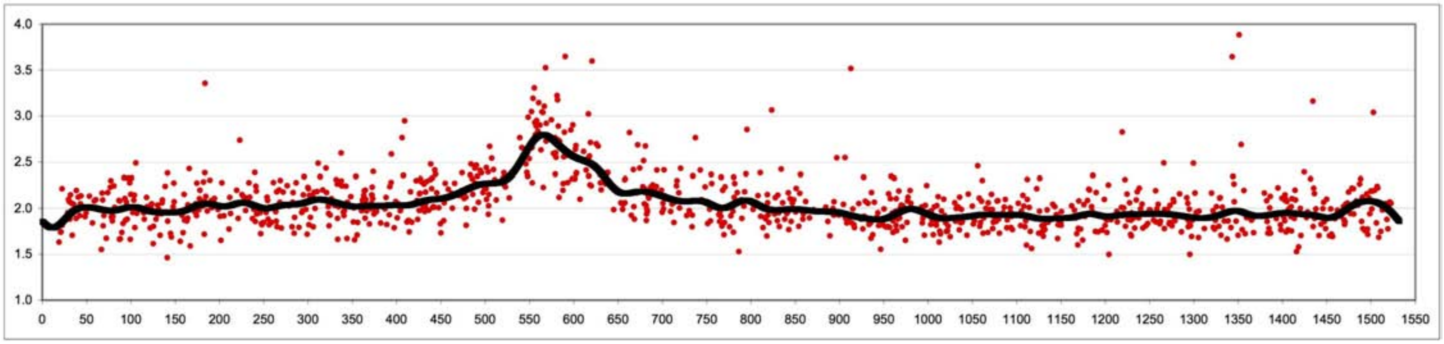

YJL8401

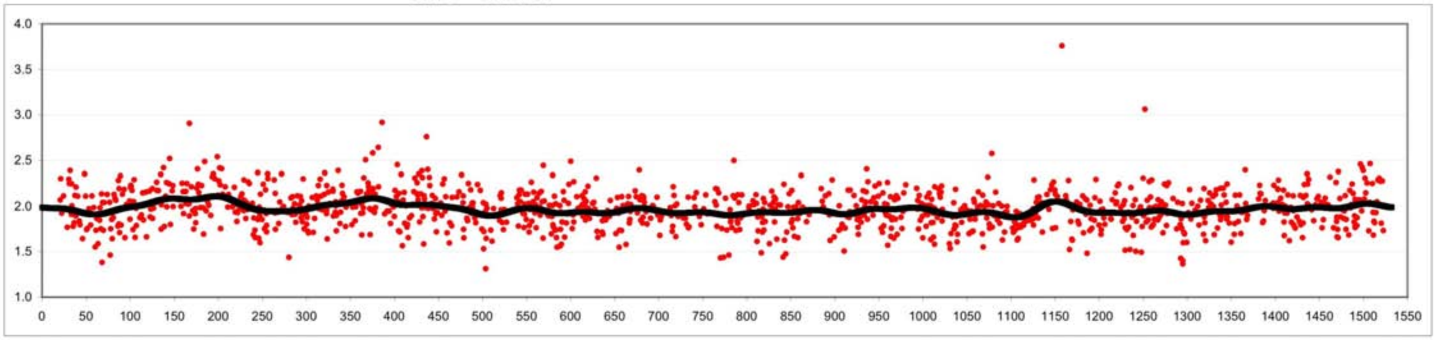

YJL8402

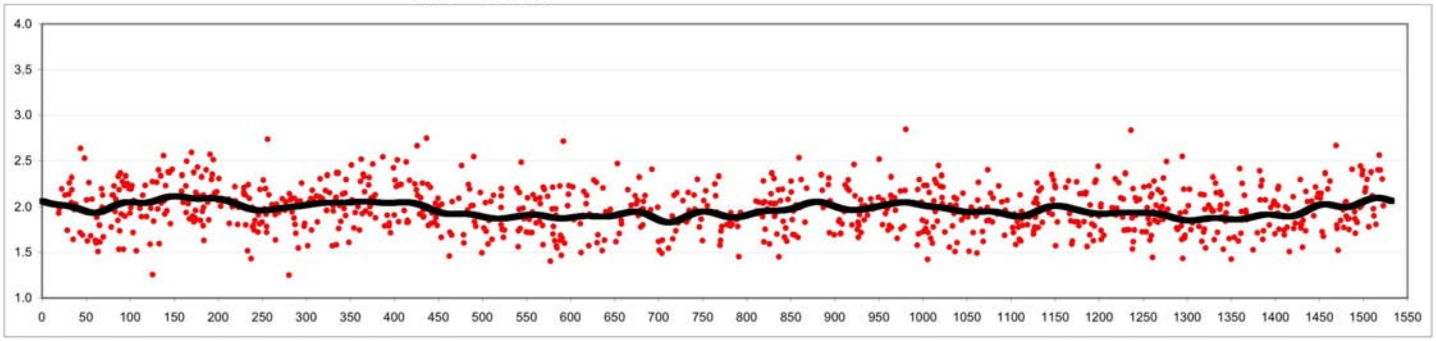

YJL8404

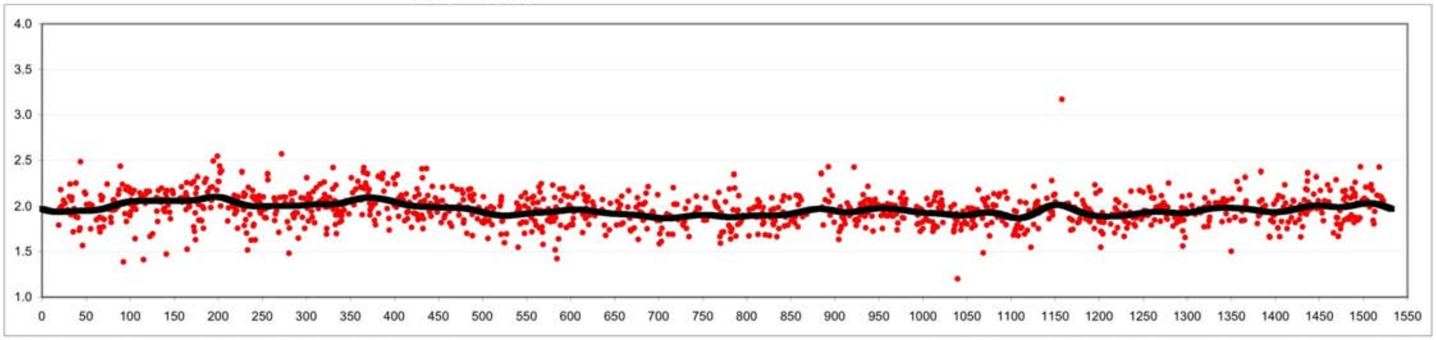

YJL8405

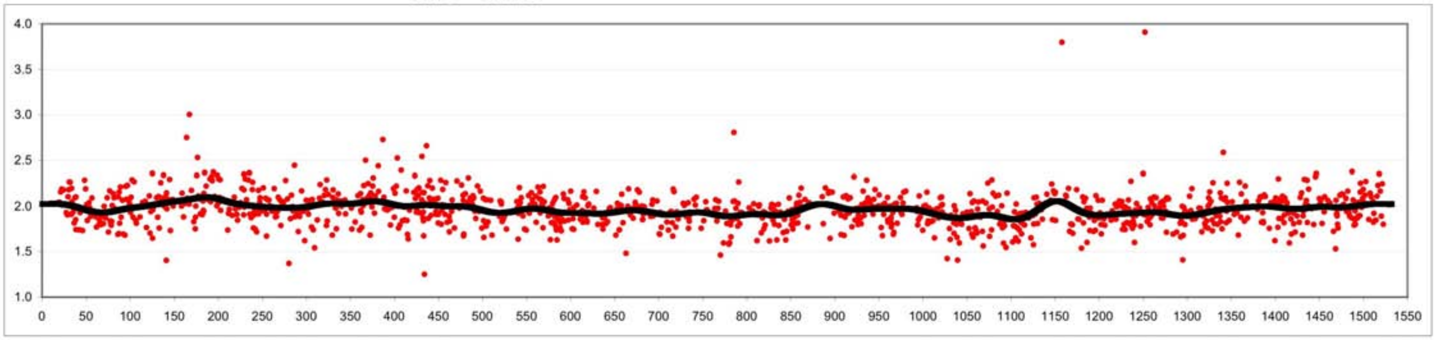

YJL8553

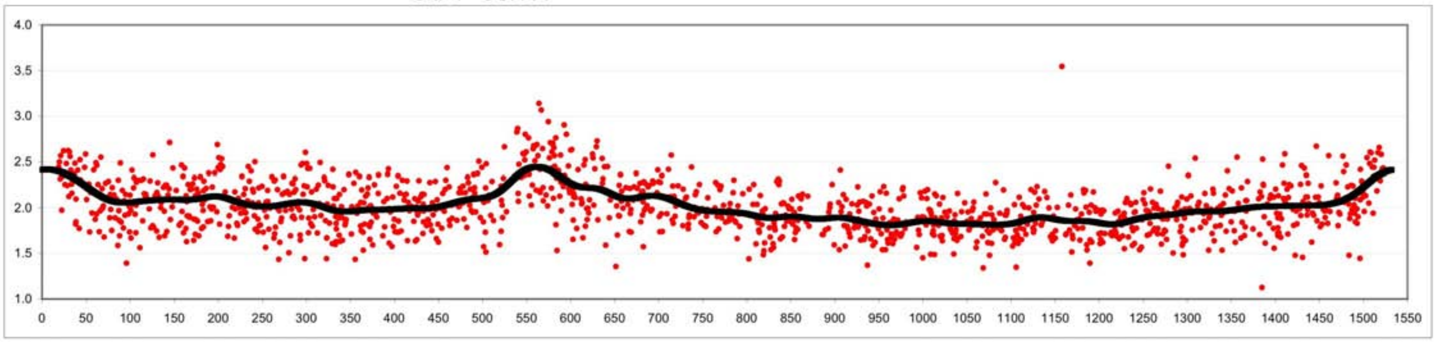

YJL8553

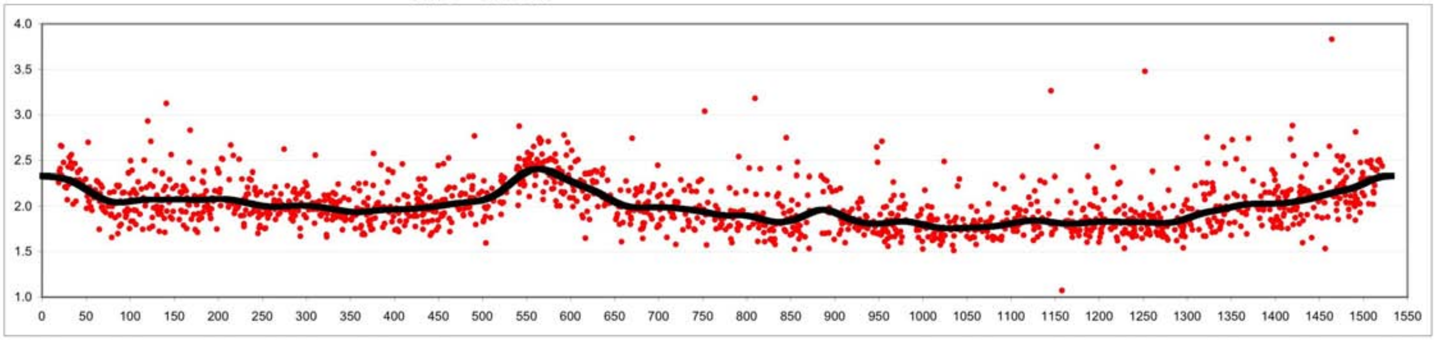

YJL8556

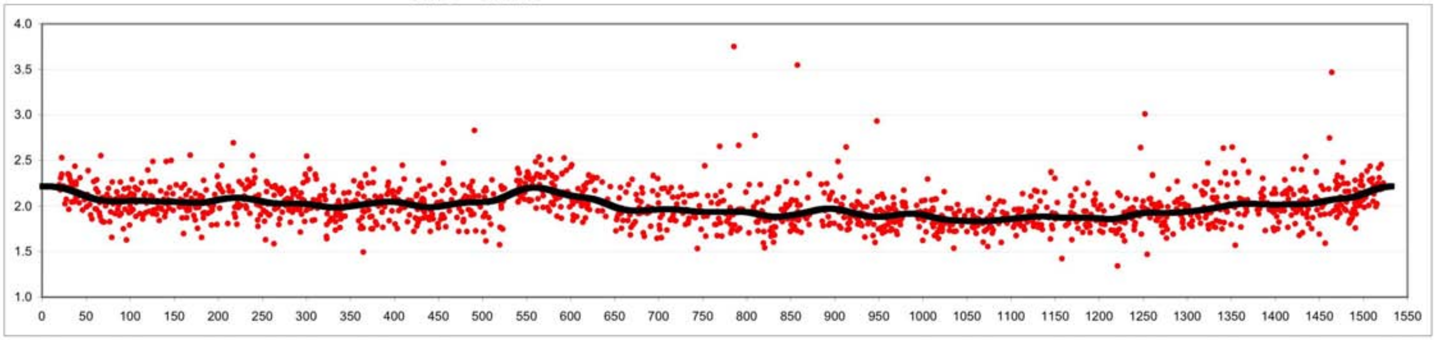

YJL8556

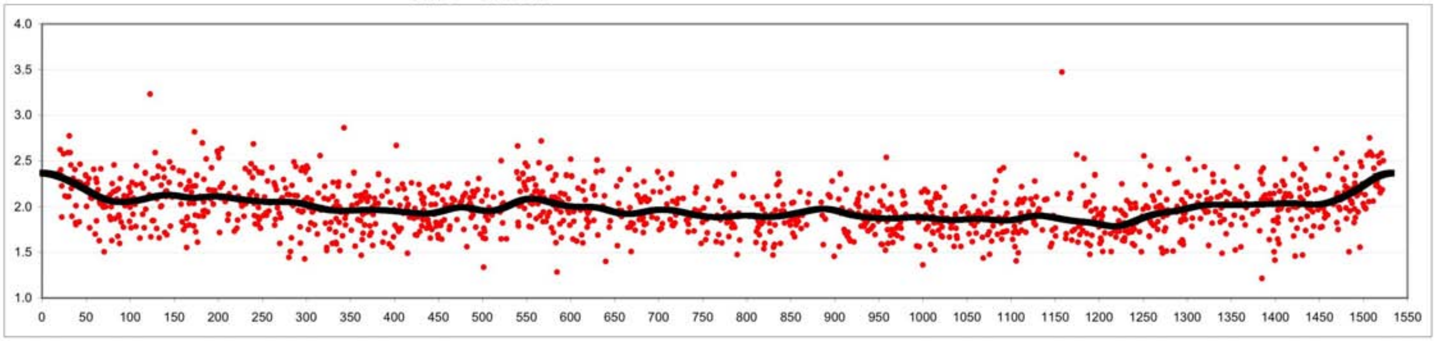

YJL8559

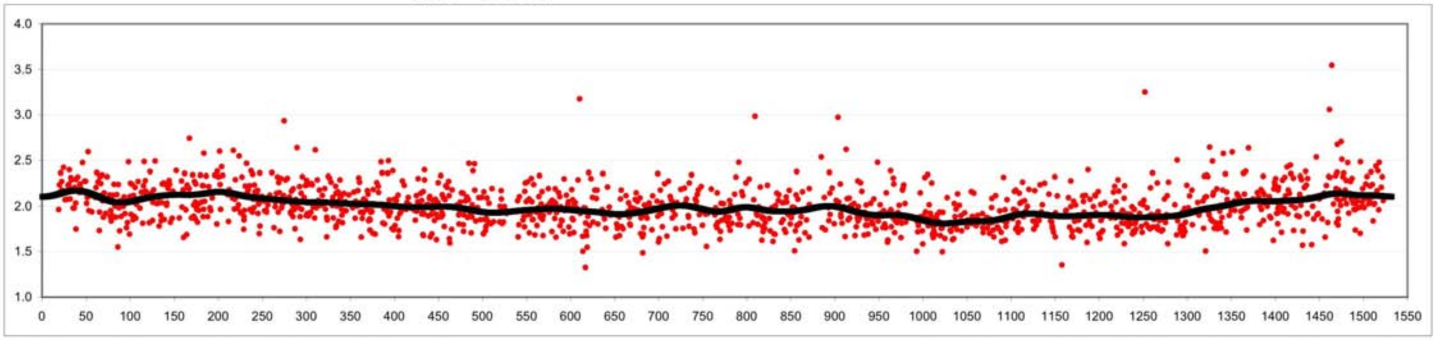

YJL8559

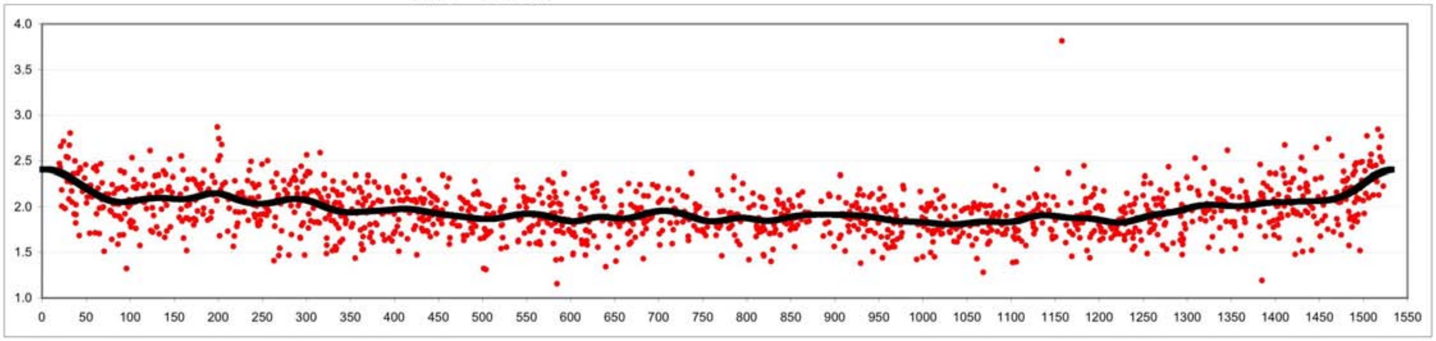

YJL8562

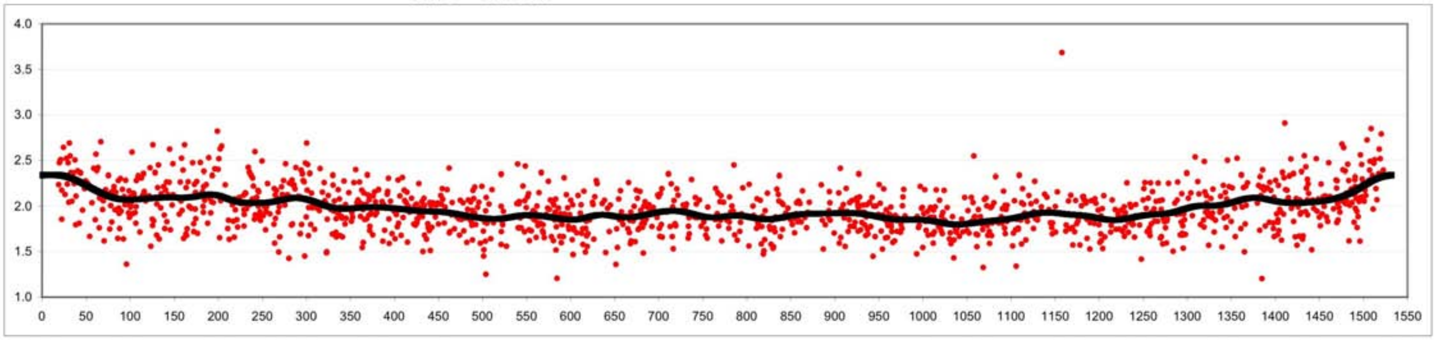

YJL8562

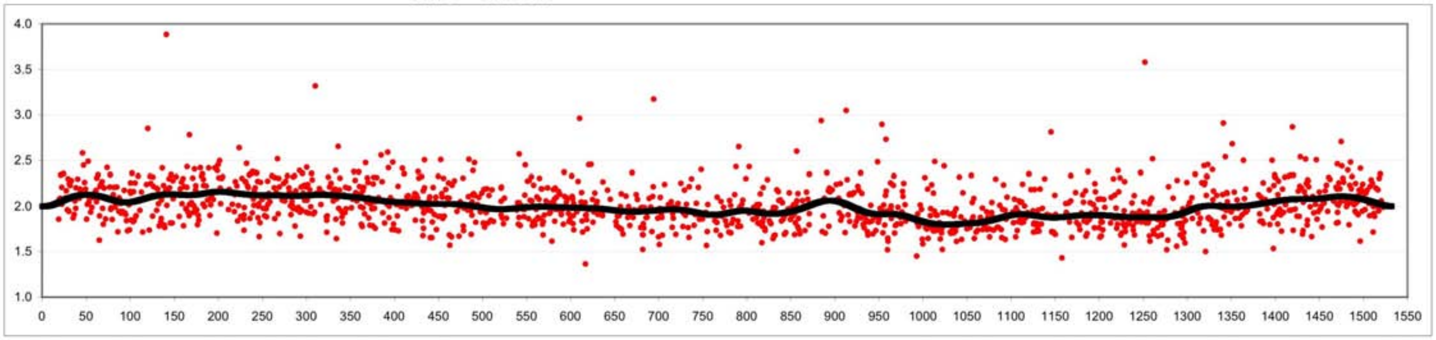

YJL8565

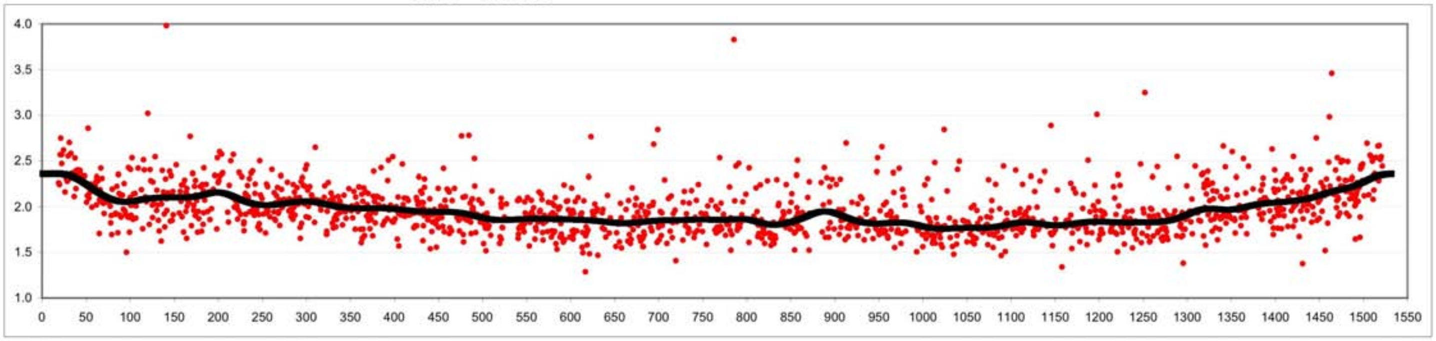

YJL8565

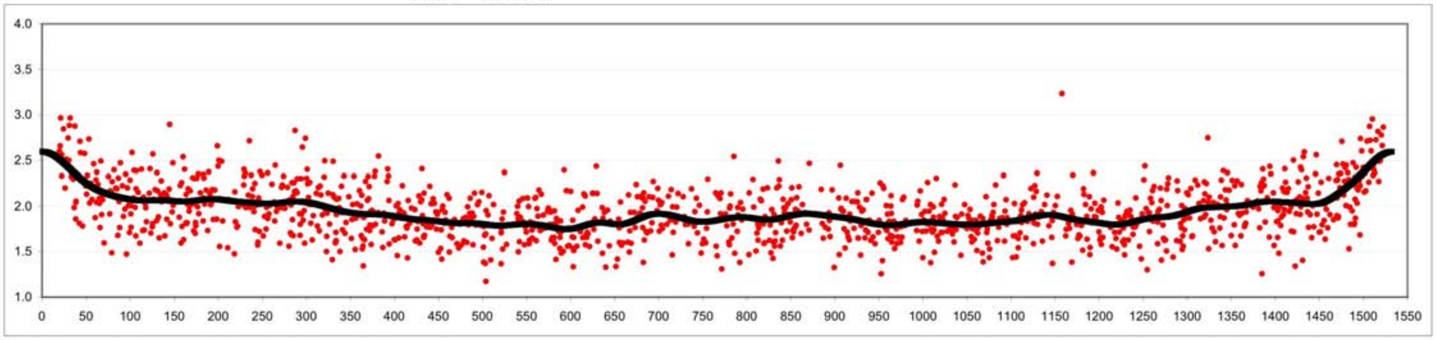

YJL10444

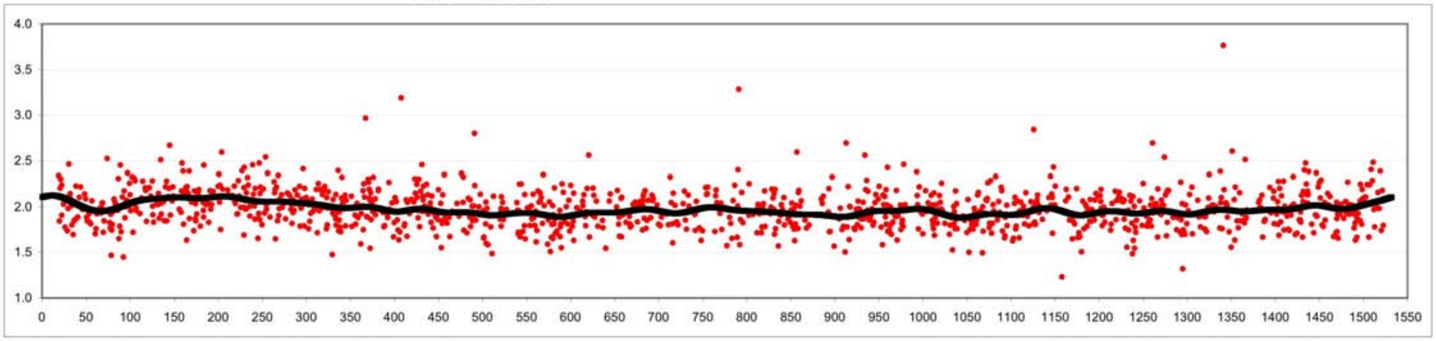

YJL10445

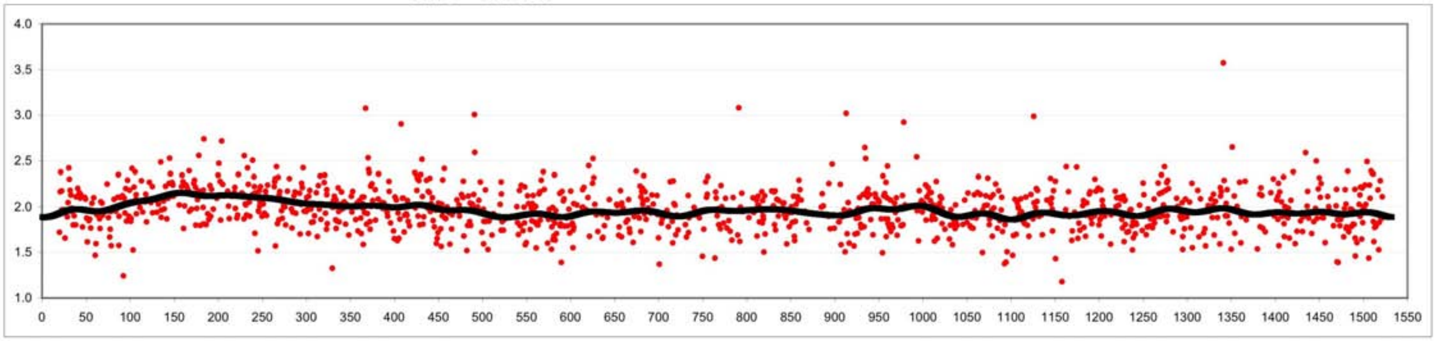

Figure 4B
YJL8398 – Control for odd linkers

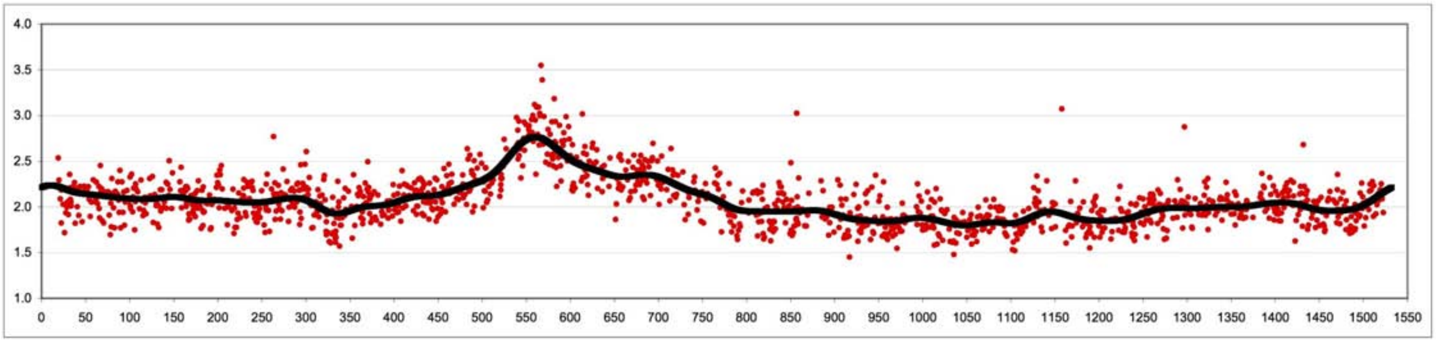

YJL8398 – Control for odd linkers

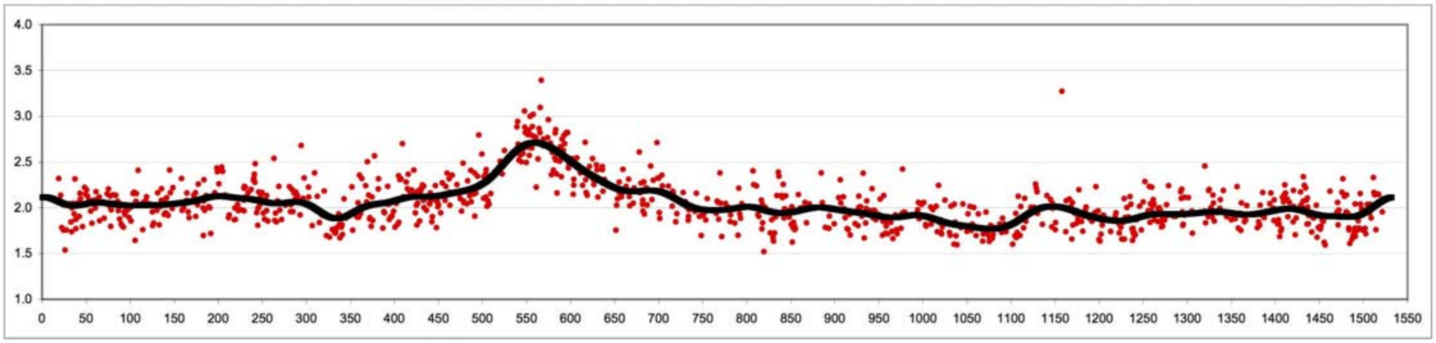

YJL8644 – Linker L1

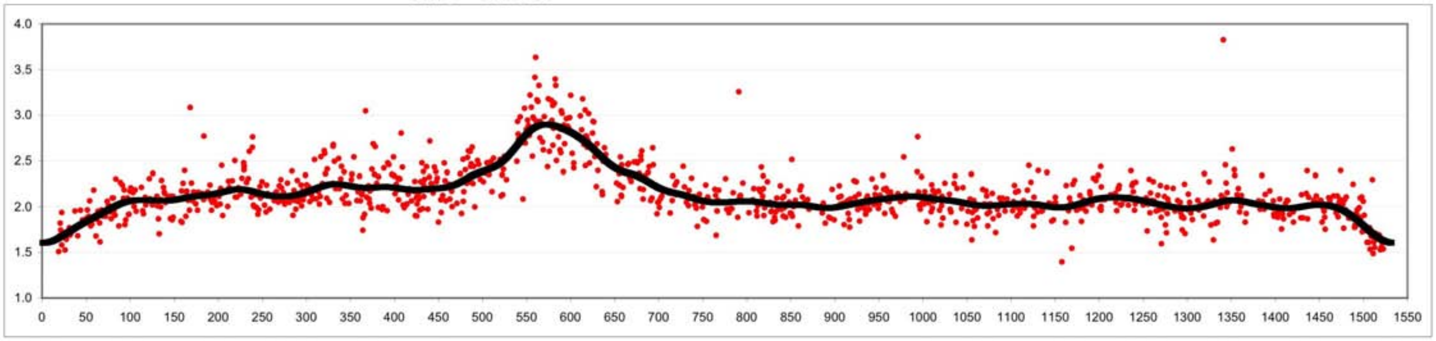

YJL8644 – Linker L1

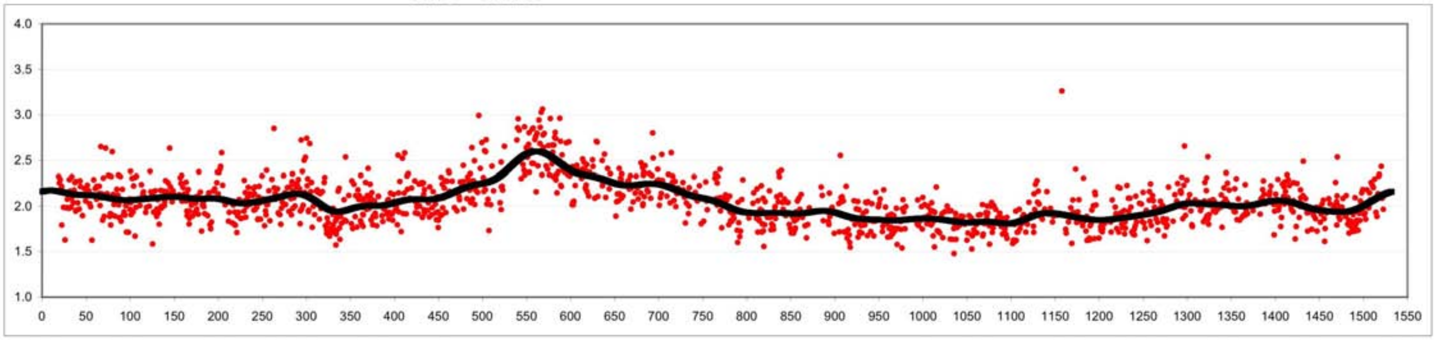

YJL8647 – Linker L3

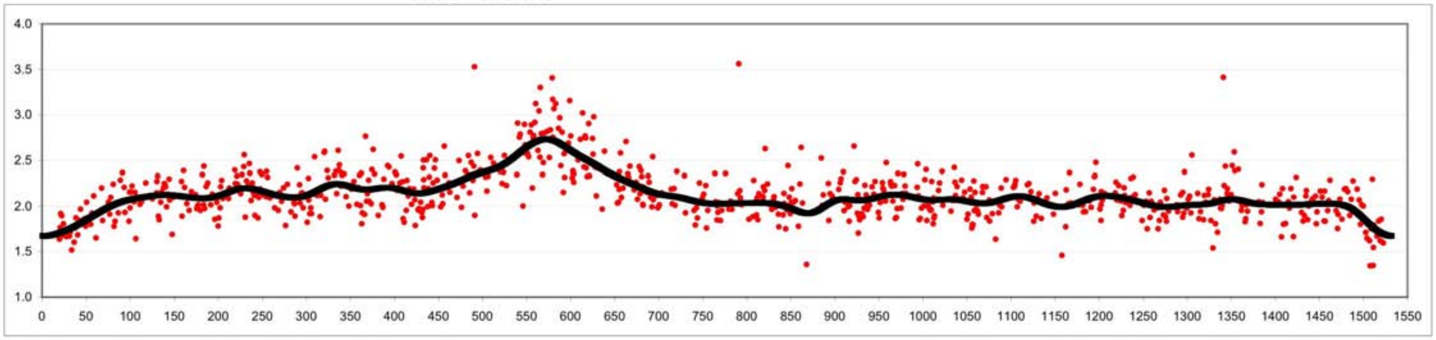

YJL8647 – Linker L3

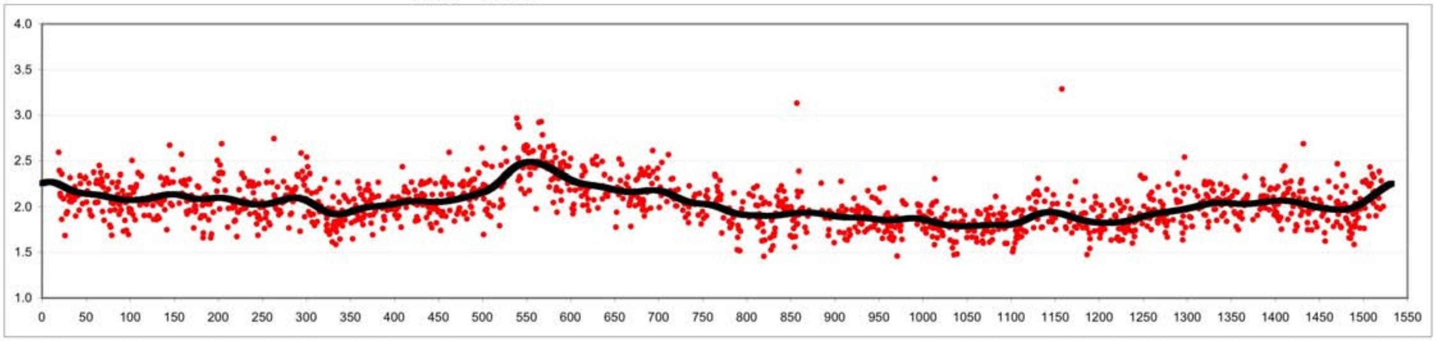

YJL8650– Linker L5

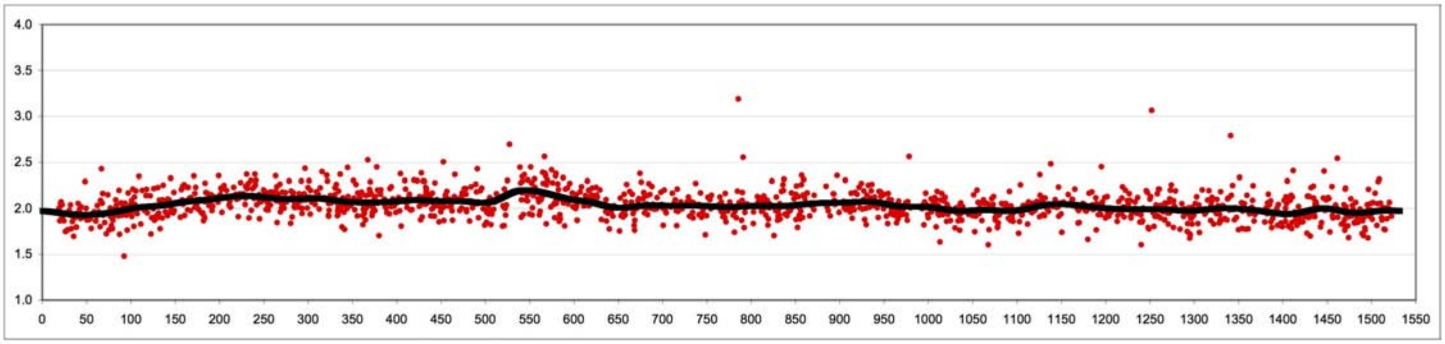

YJL8650 – Linker L5

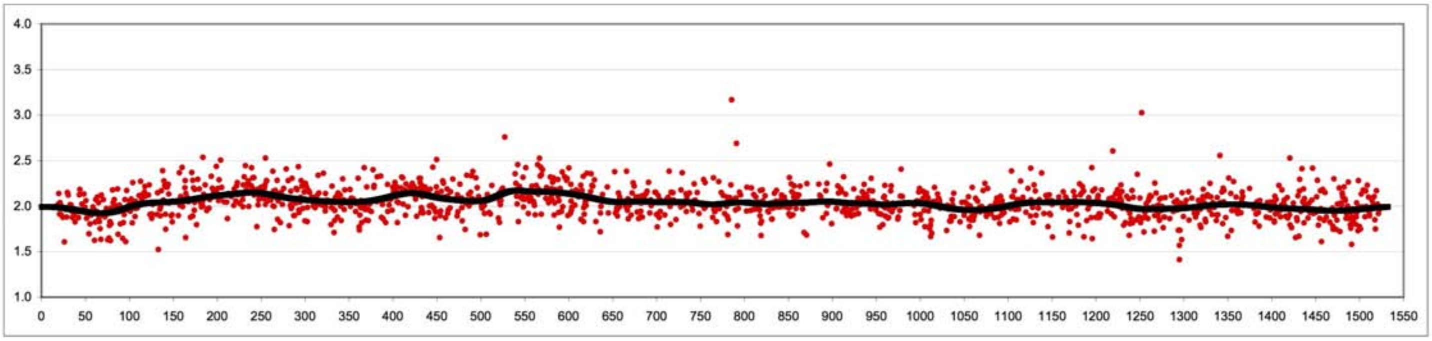

YJL8653 – Linker L7

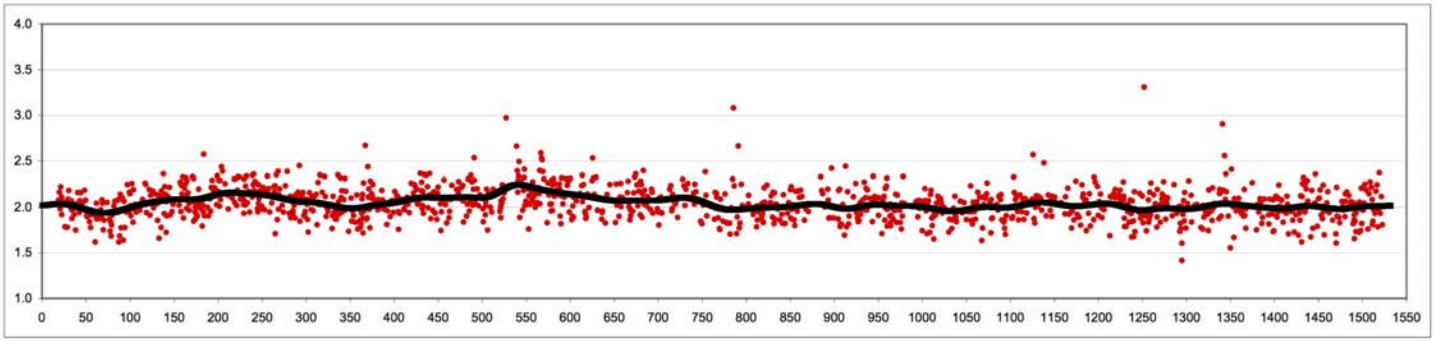

YJL8653 – Linker L7

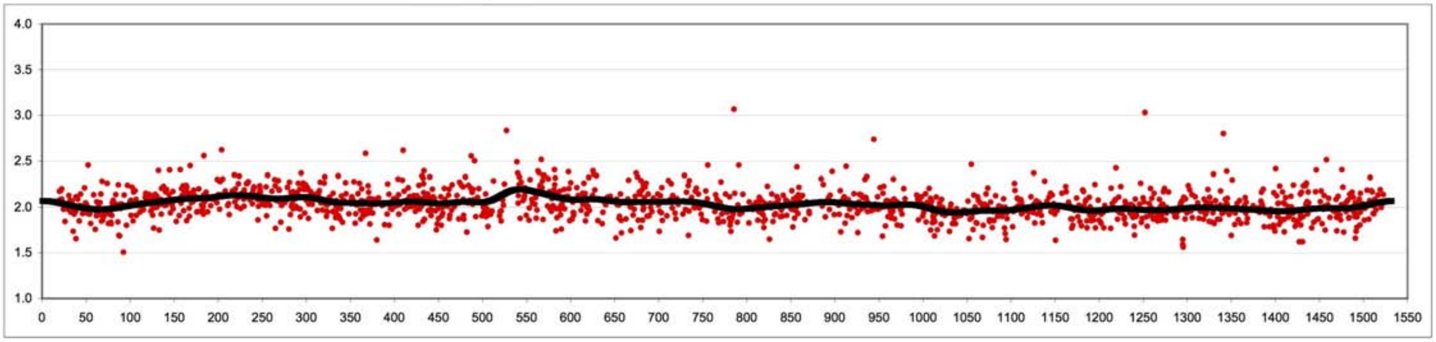

YJL8656- Linker L9

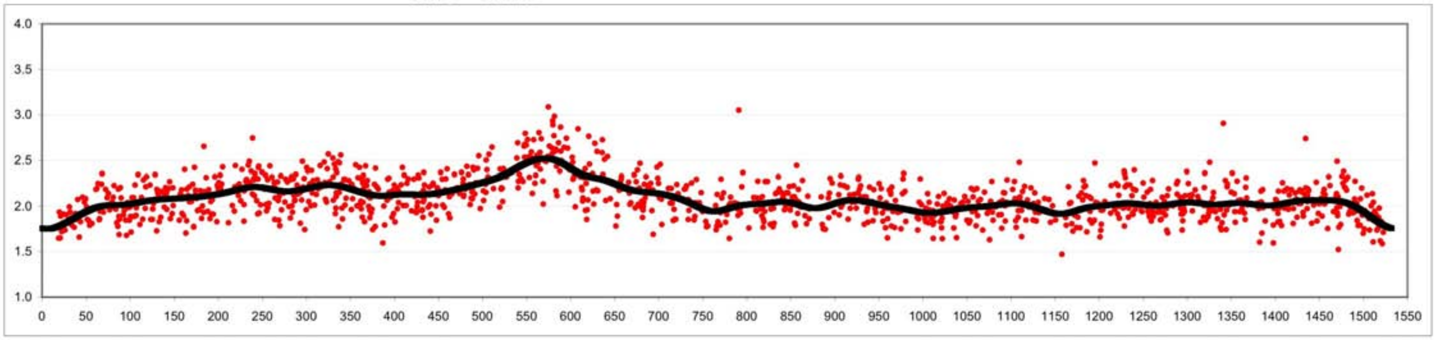

YJL8656- Linker L9

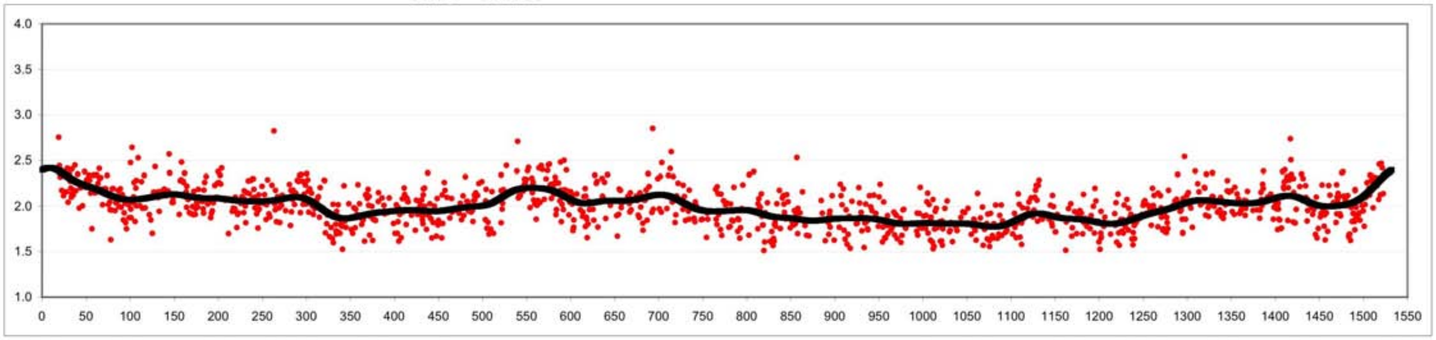

YJL8677 – Linker L11

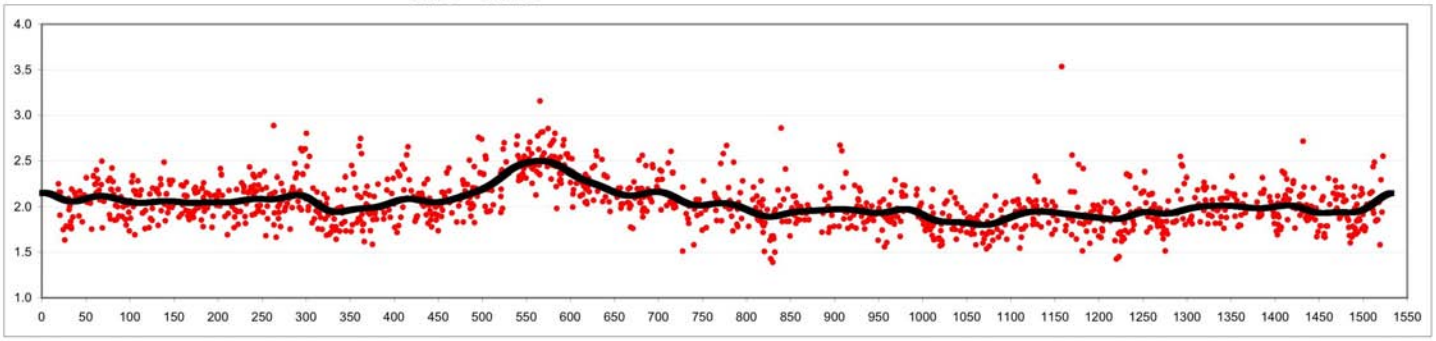

YJL8677 – Linker L11

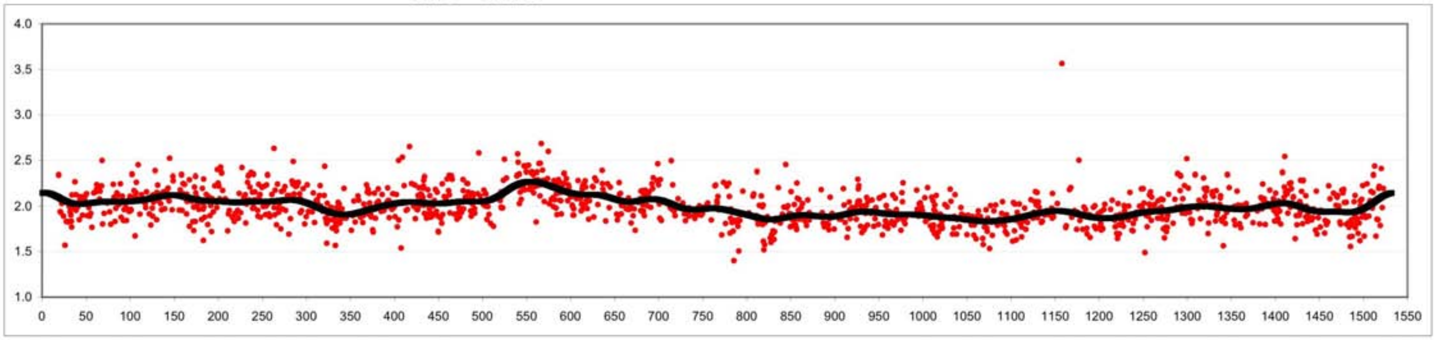

YJL8680 – Linker L13

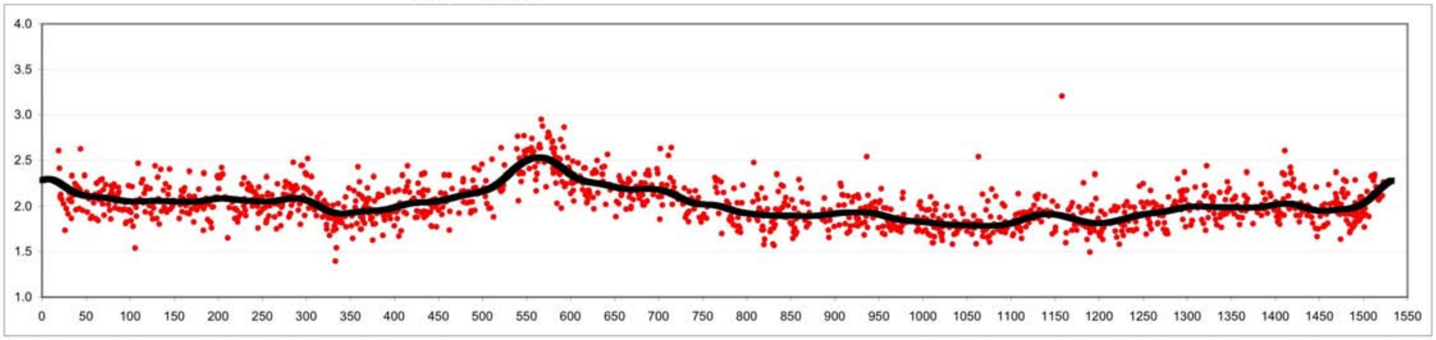

YJL8680 – Linker L13

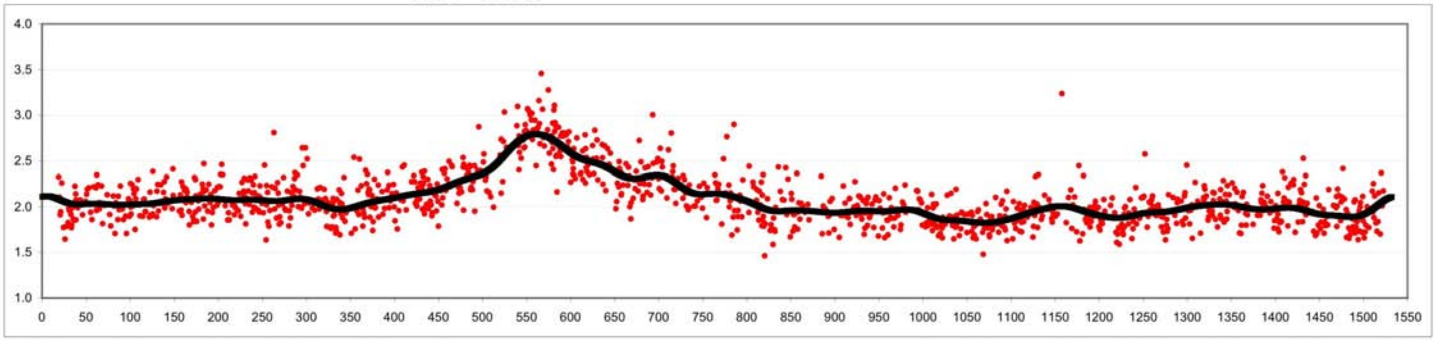

YJL8659 – Linker L15

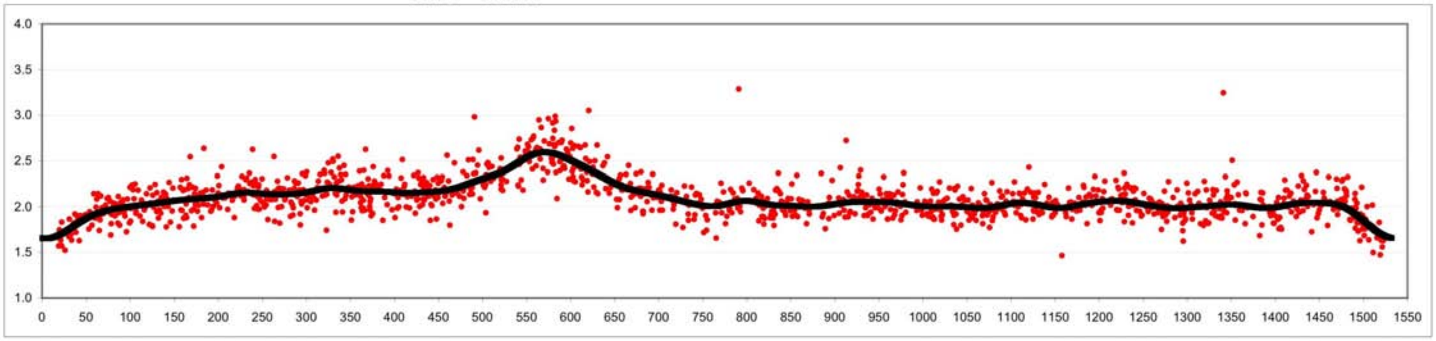

YJL8659 – Linker L15

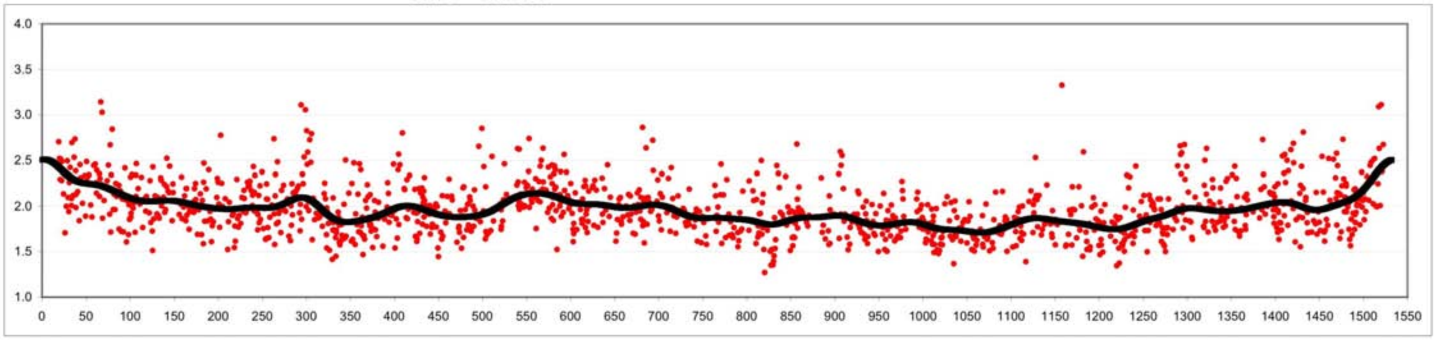

YJL8683 – Linker L17

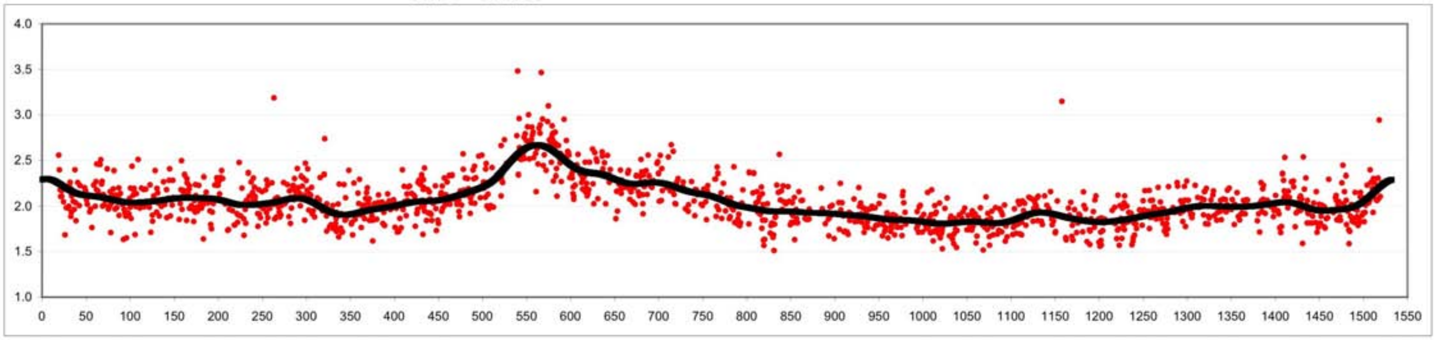

YJL8683 – Linker L17

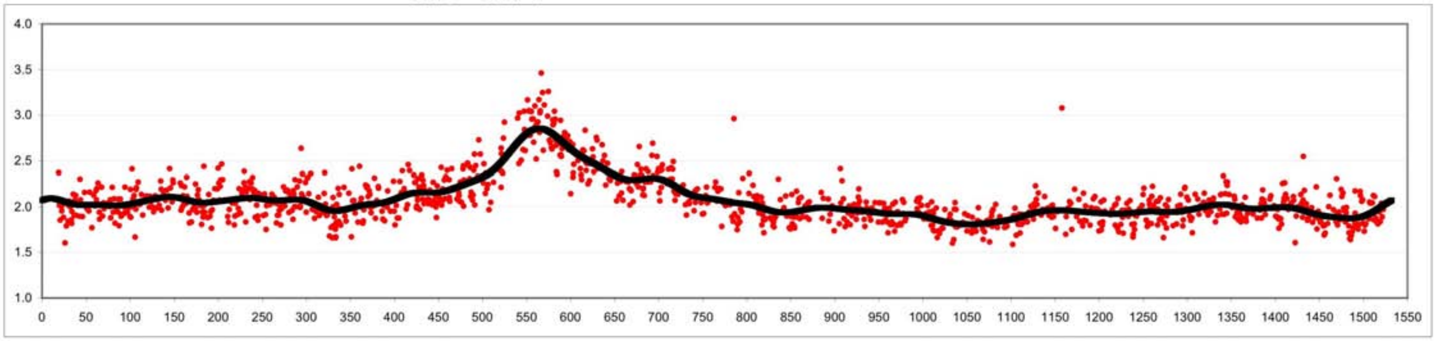

YJL8662 – Linker L19

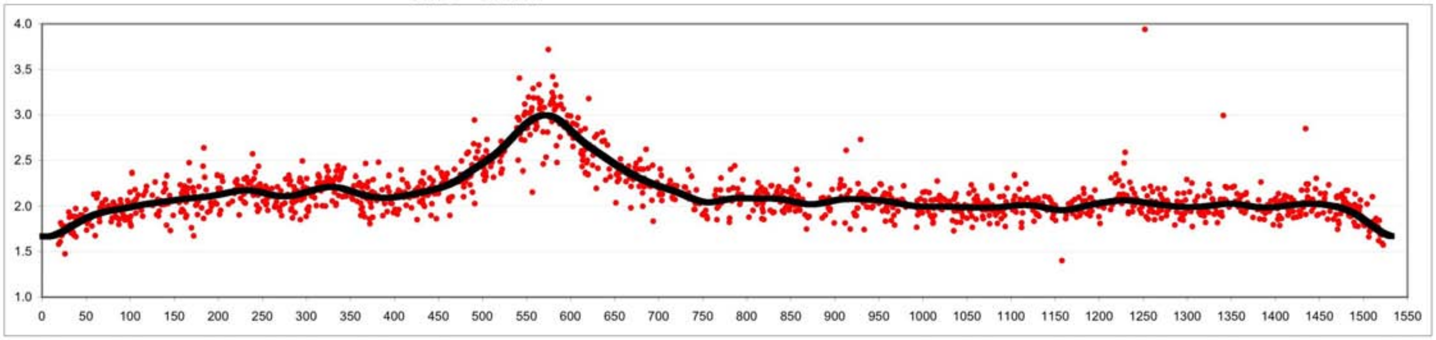

YJL8662 – Linker L19

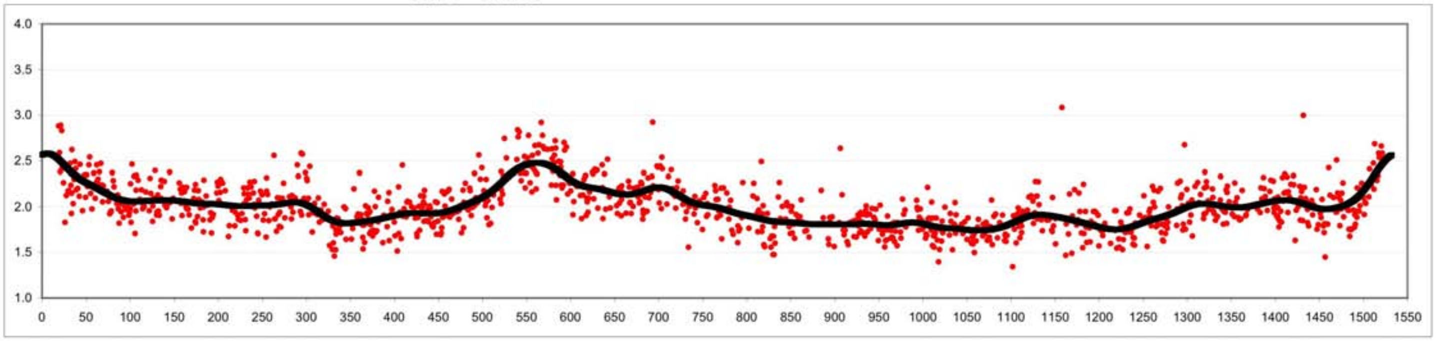

YJL8686 – Linker L21

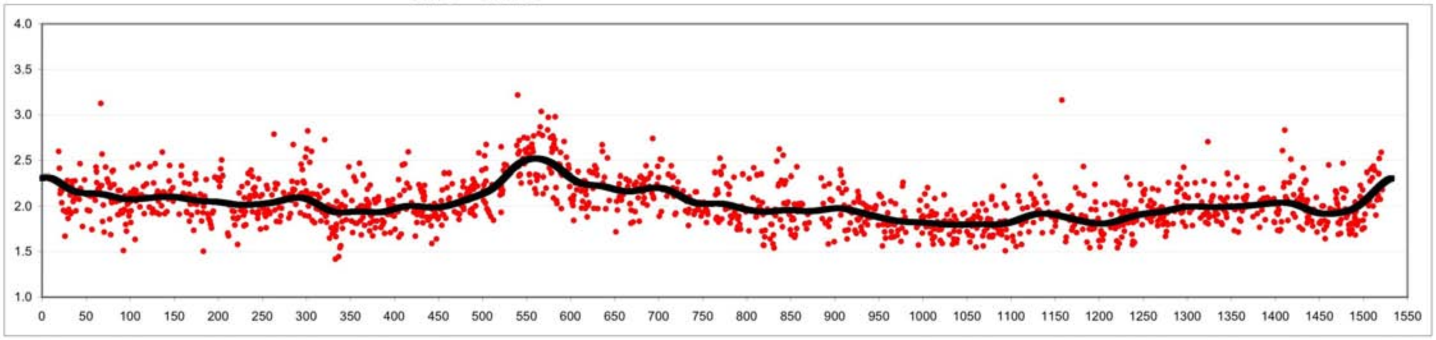

YJL8686 – Linker L21

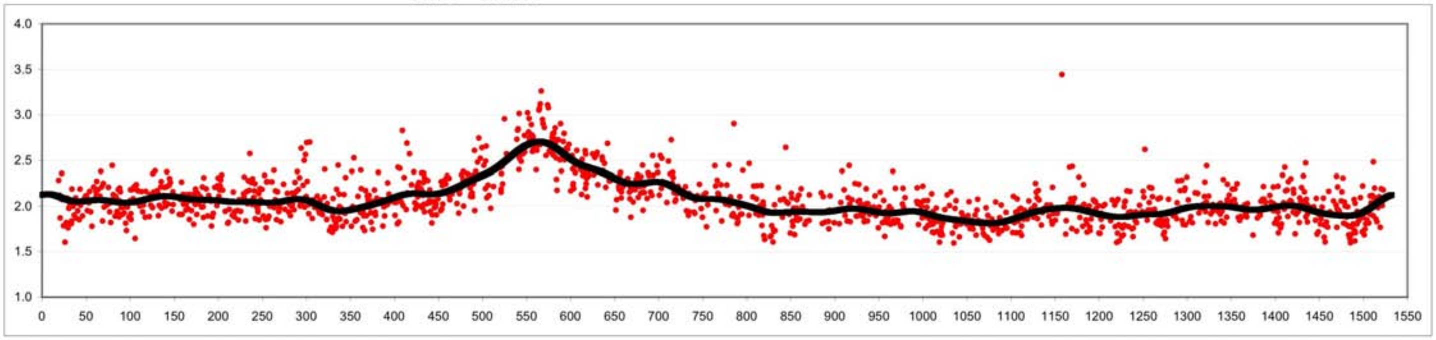

YJL8665 – Linker L23

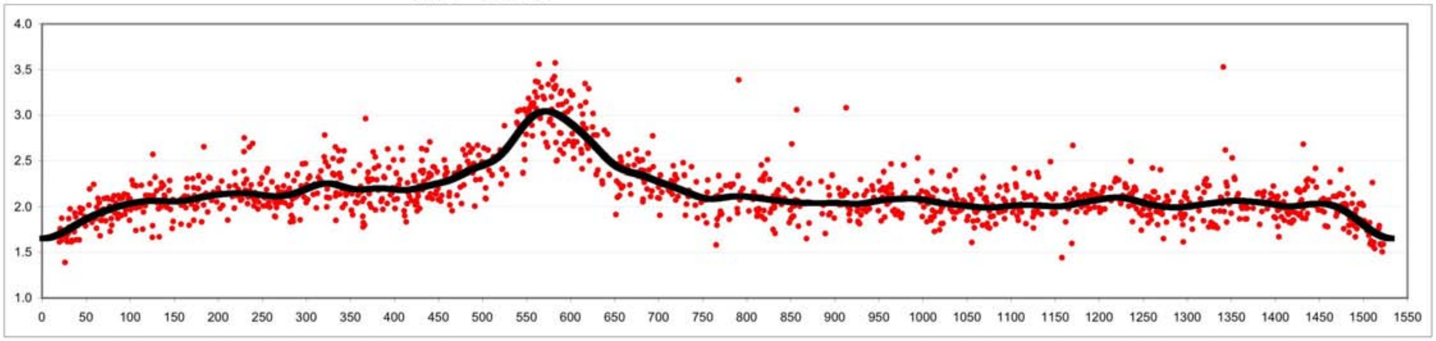

YJL8665 – Linker L23

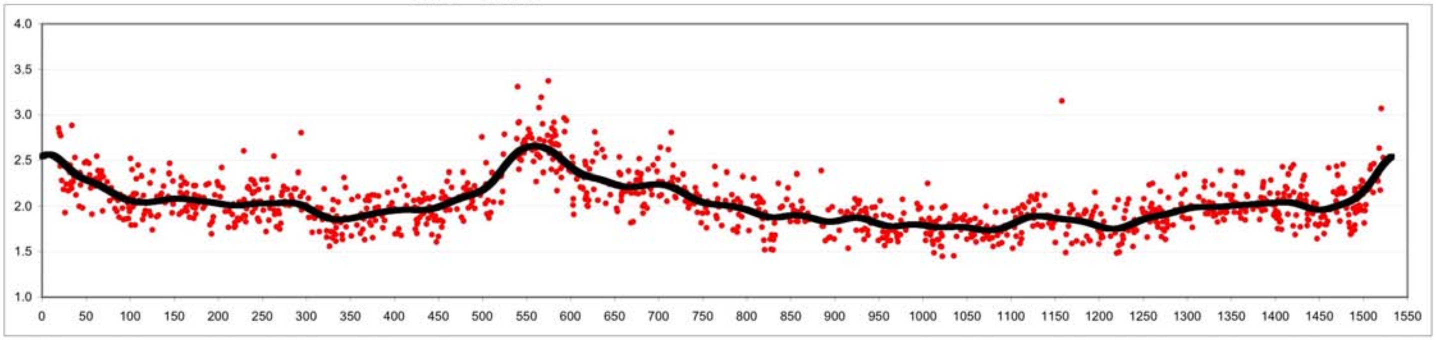

YJL8668 – Linker L25

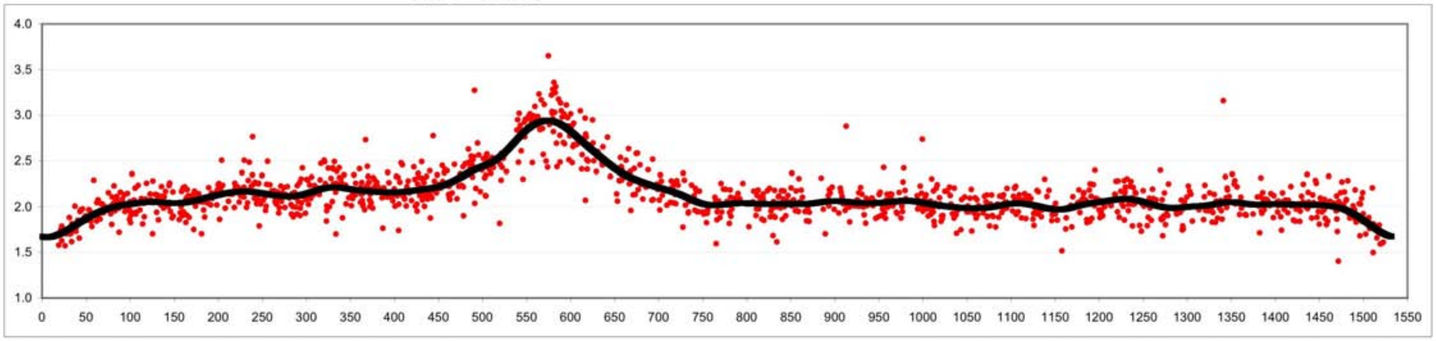

YJL8668 – Linker L25

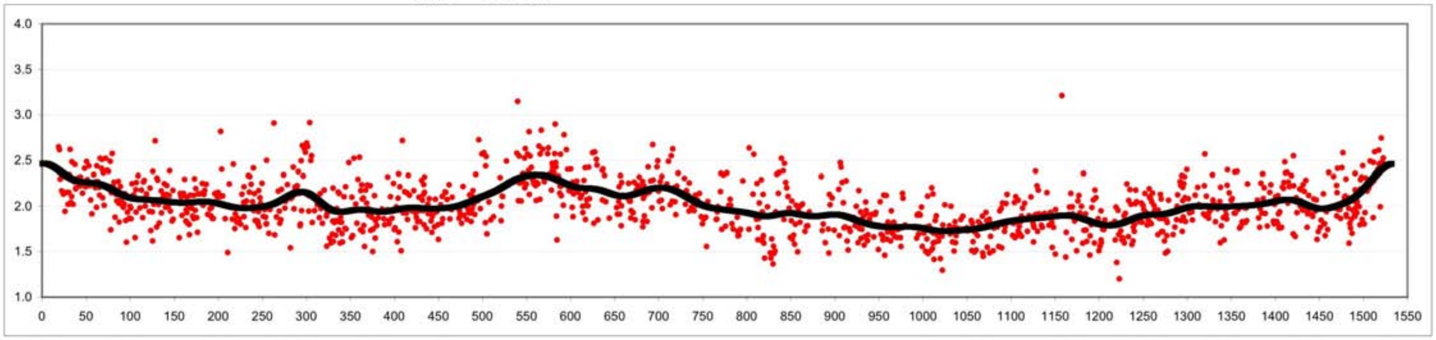

YJL8671 – Linker L27

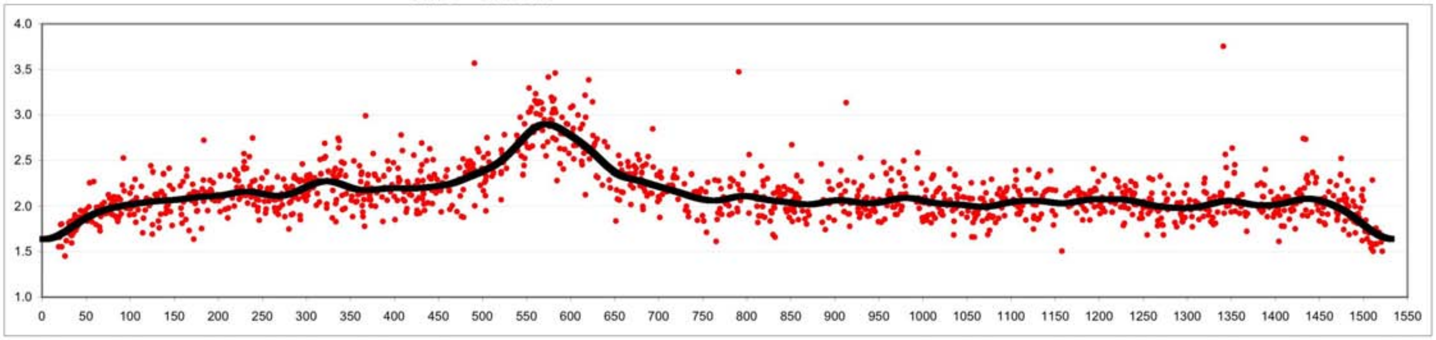

YJL8671 – Linker L27

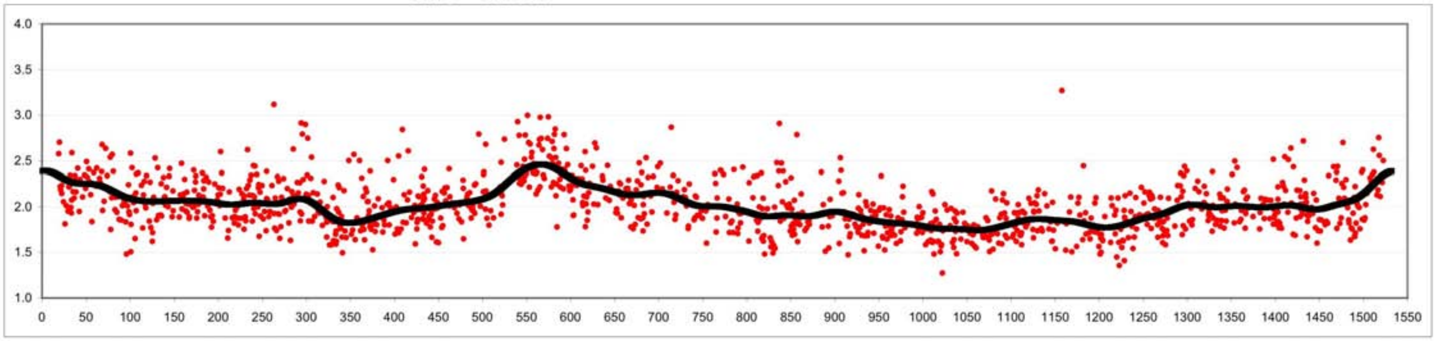

YJL8689 – Linker L29

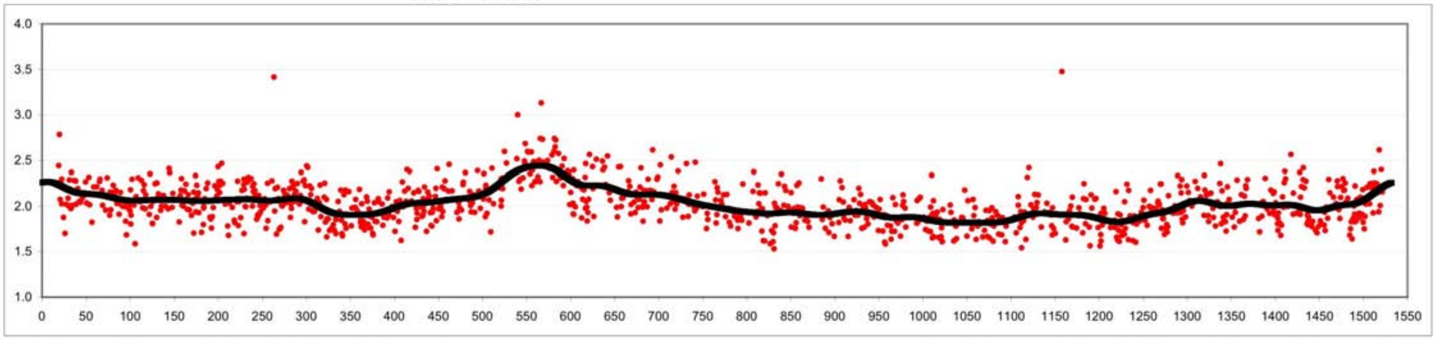

YJL8689 – Linker L29

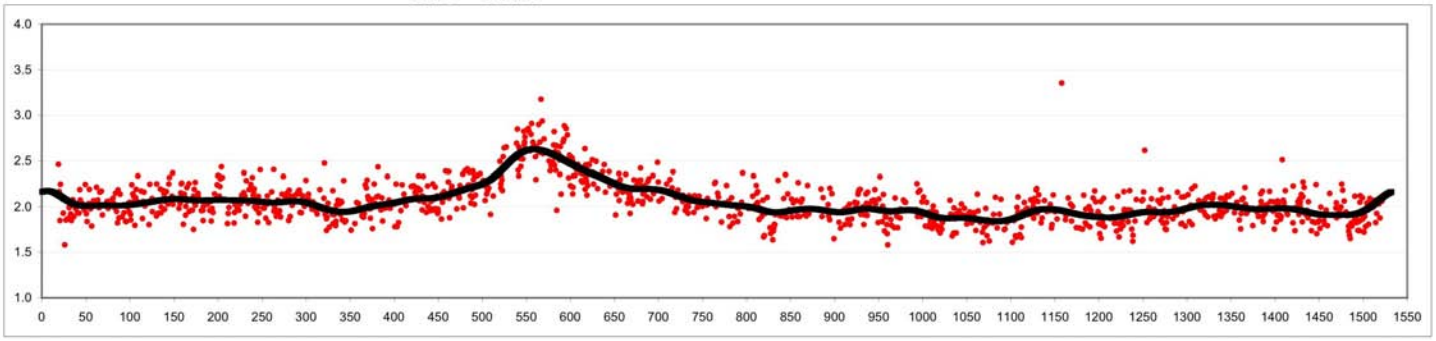

YJL8692 – Linker L31

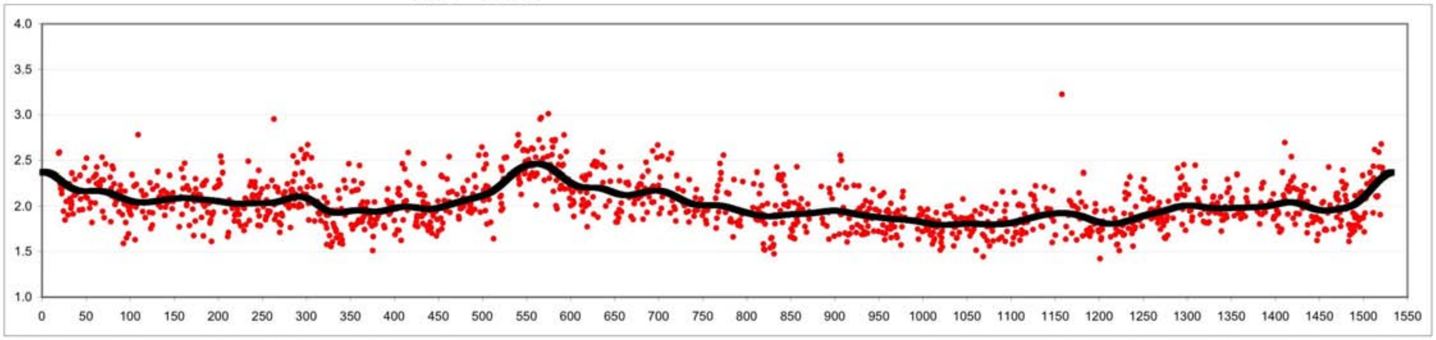

YJL8692 – Linker L31

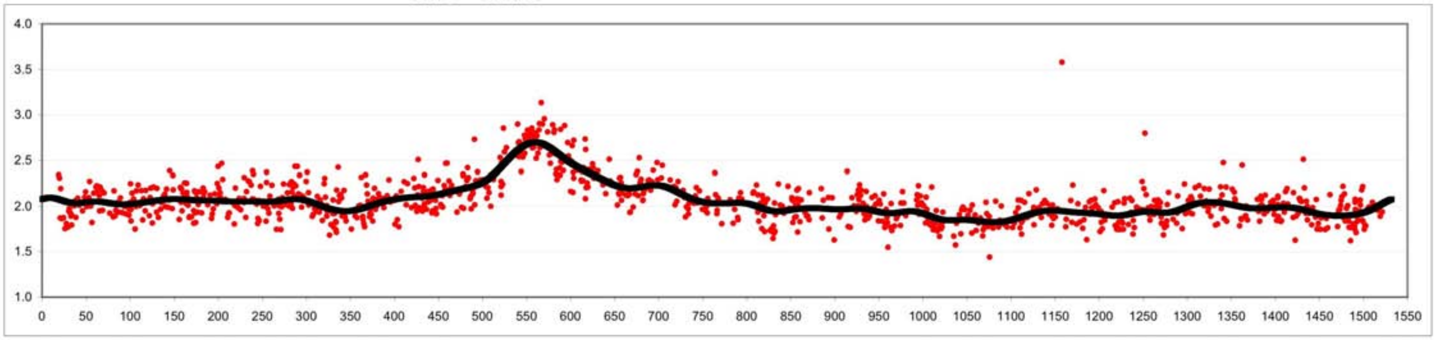

YJL8695 – Linker L33

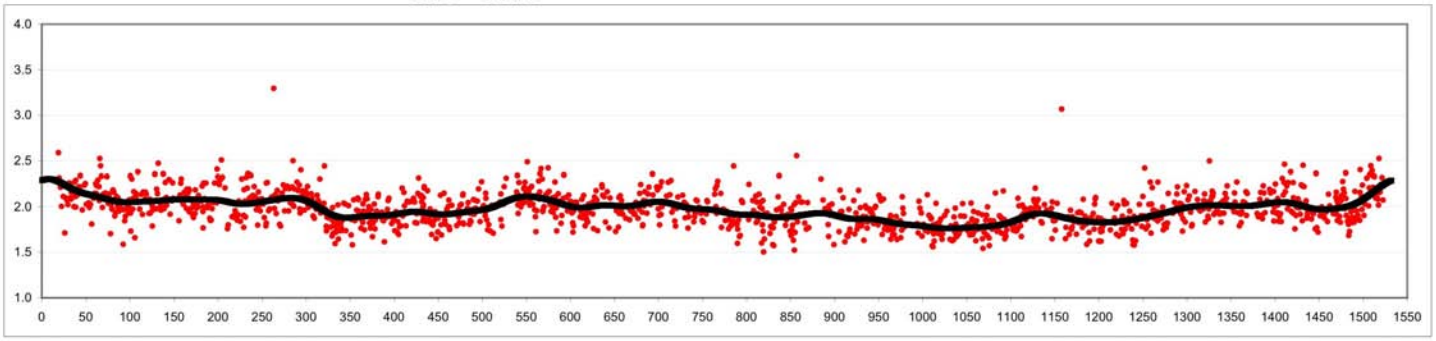

YJL8695 – Linker L33

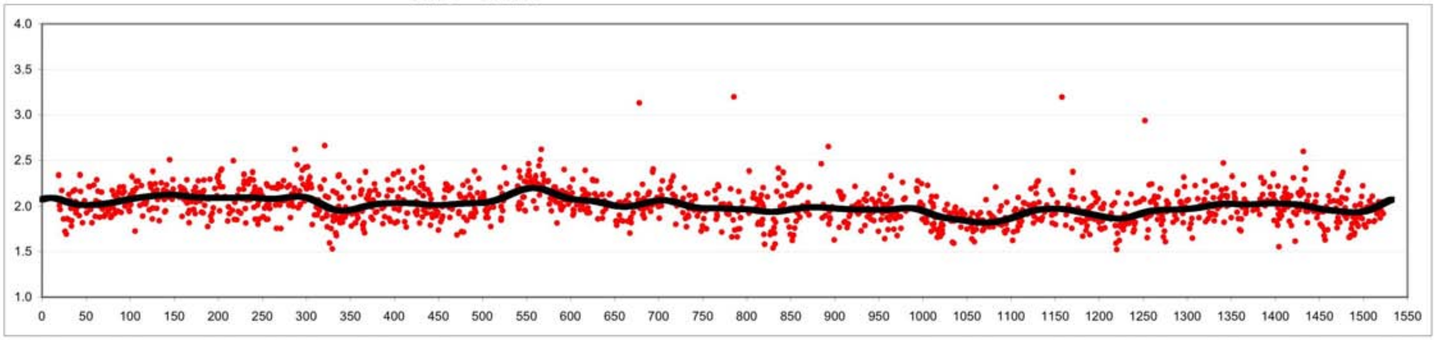

YJL8398 – control for even linkers

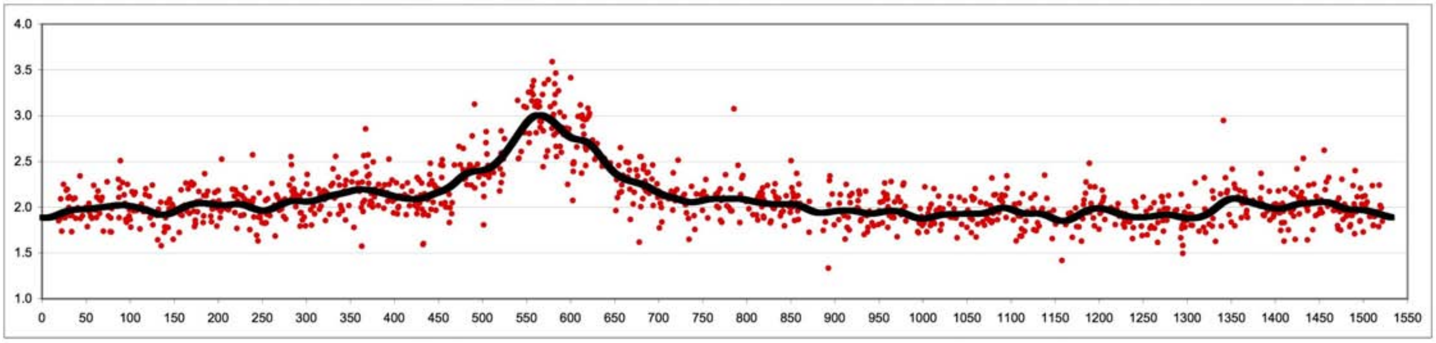

YJL8398 – control for even linkers

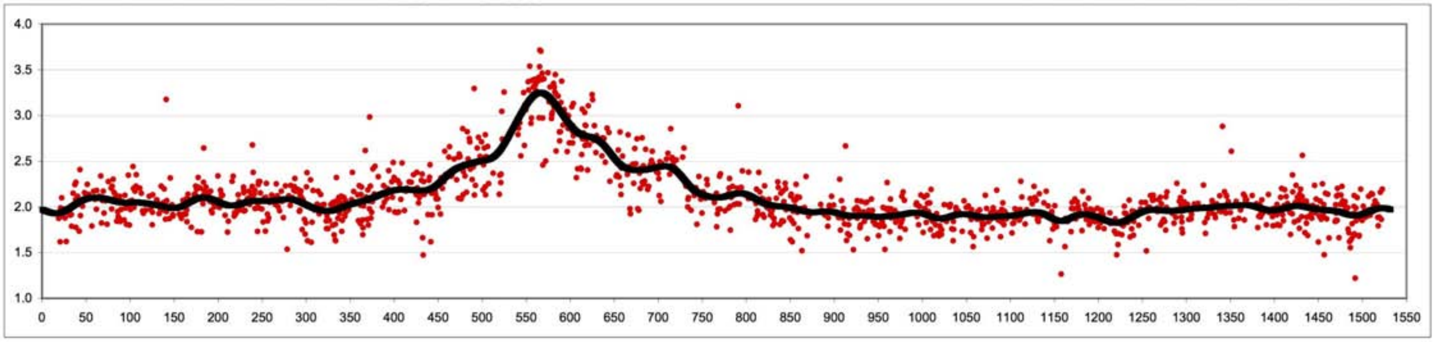

YJL8973 – Linker L2

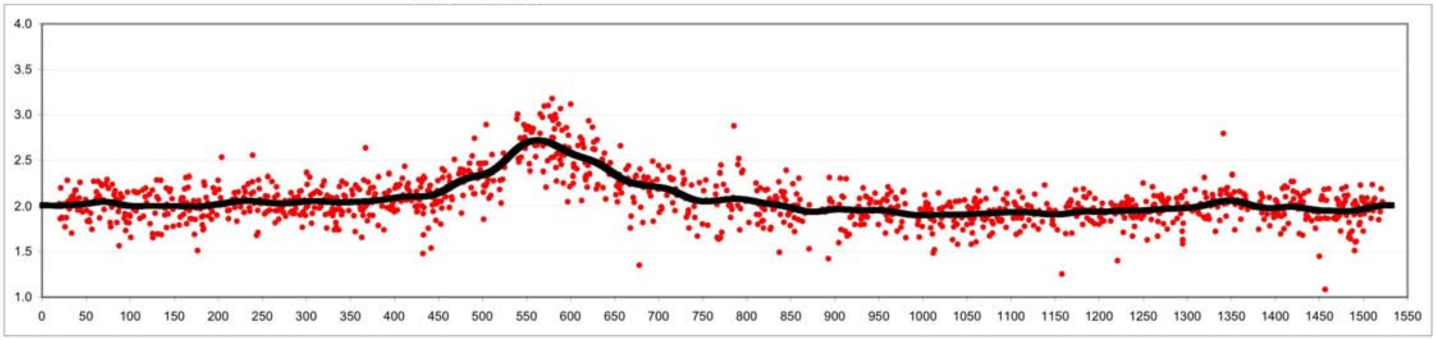

YJL8973 – Linker L2

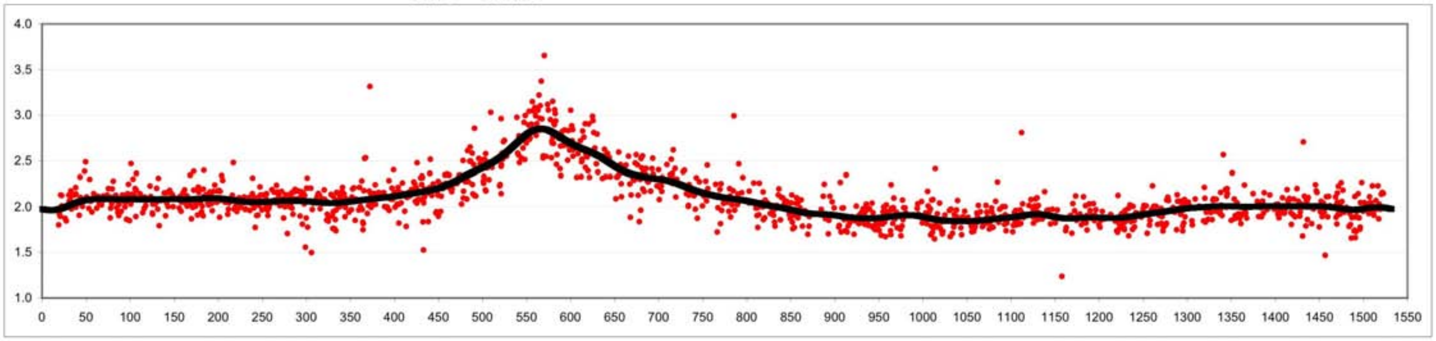

YJL8975 – Linker L4

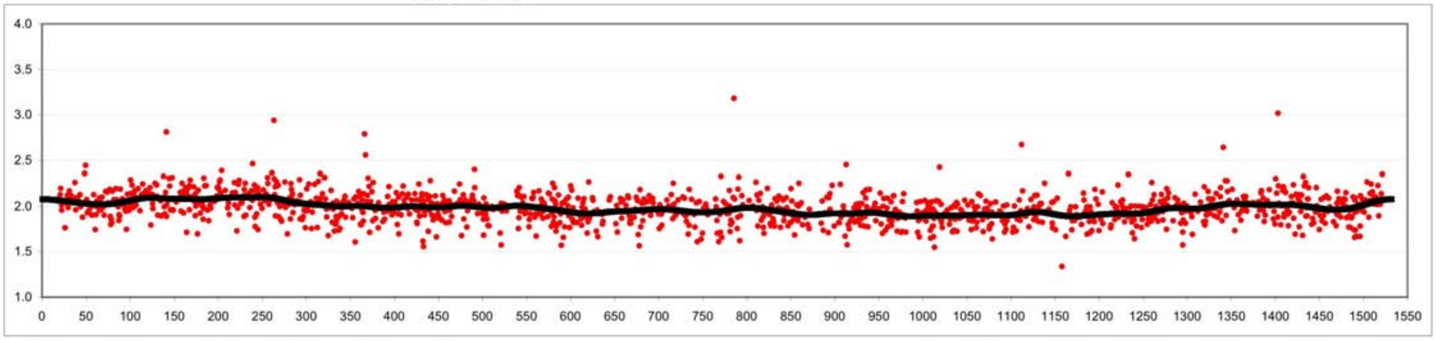

YJL8975 – Linker L4

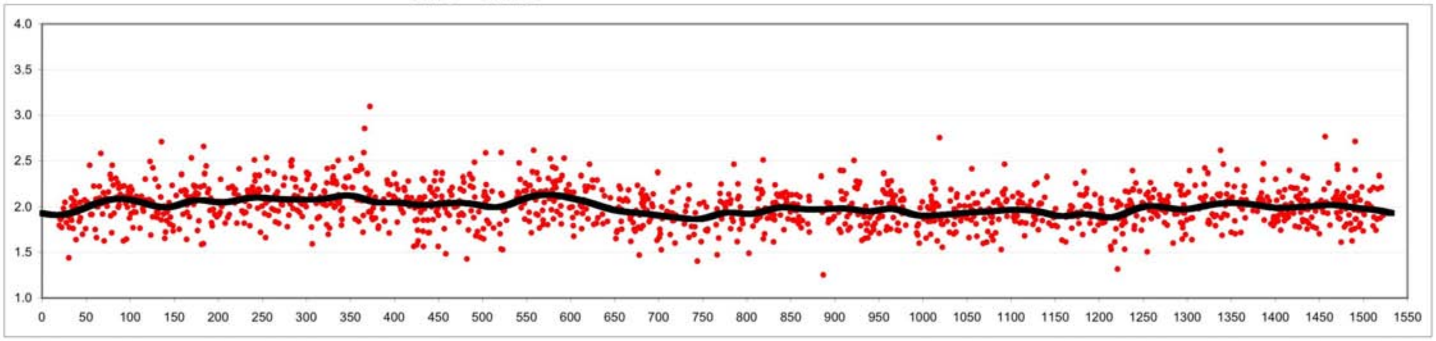

YJL9016 – Linker L6

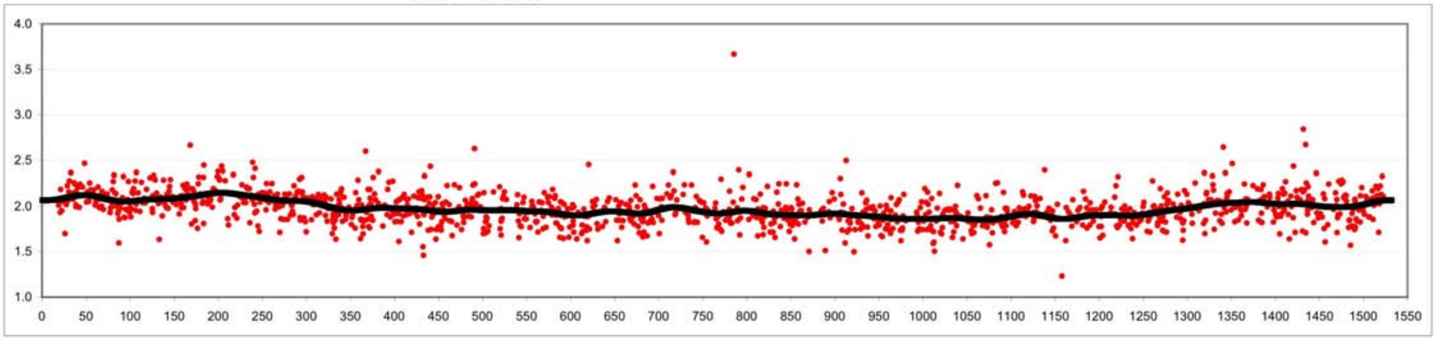

YJL9016 – Linker L6

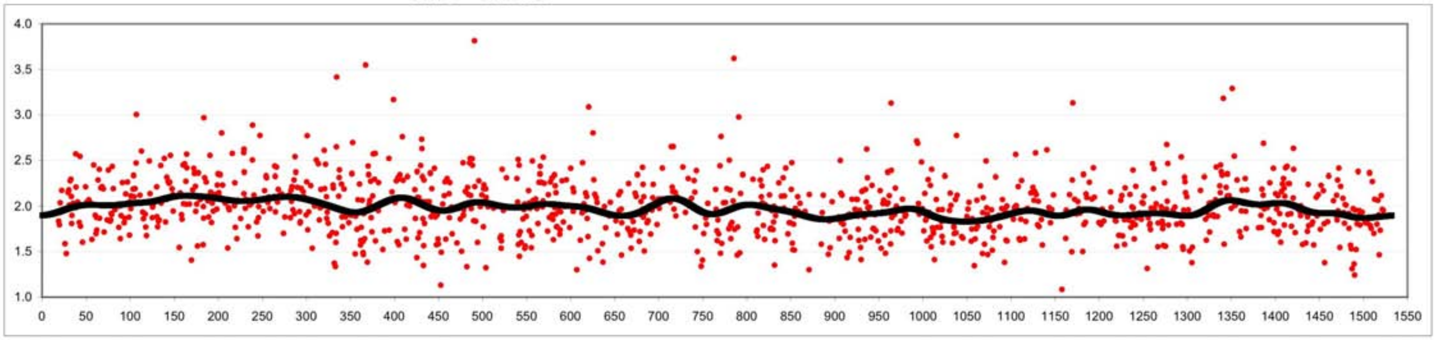

YJL8977 – Linker L8

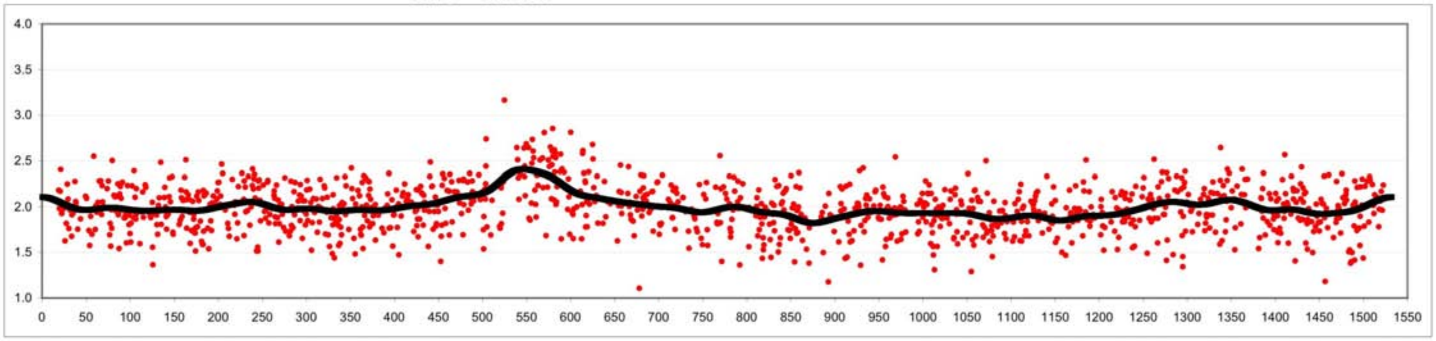

YJL8977 – Linker L8

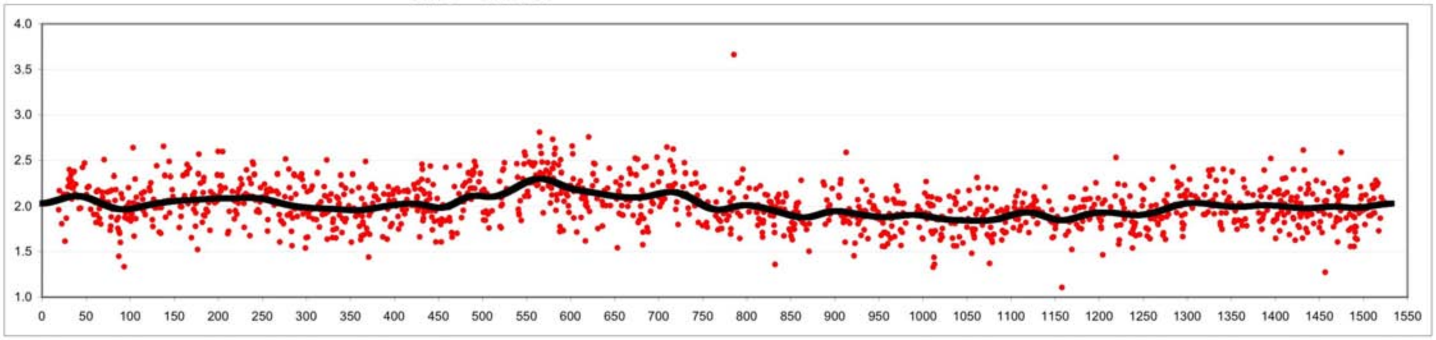

YJL8979 – Linker L10

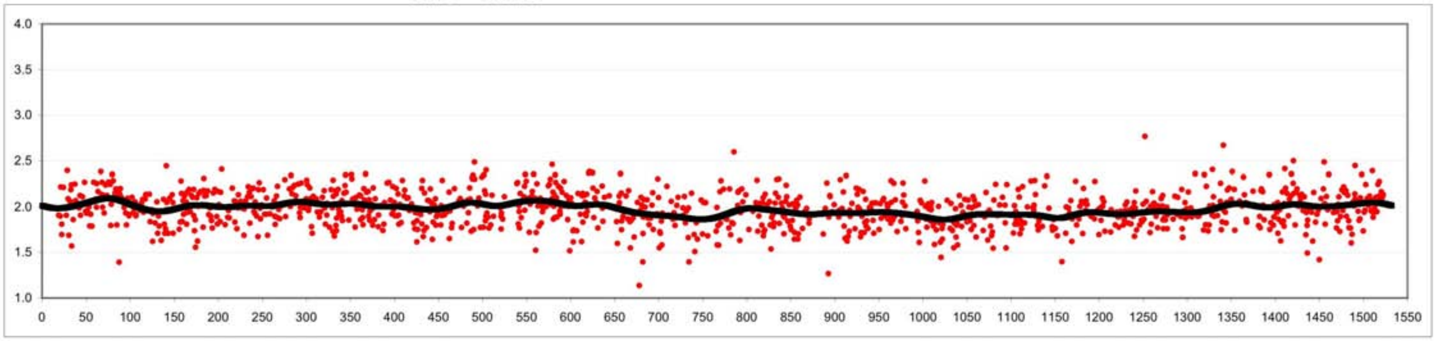

YJL8979 – Linker L10

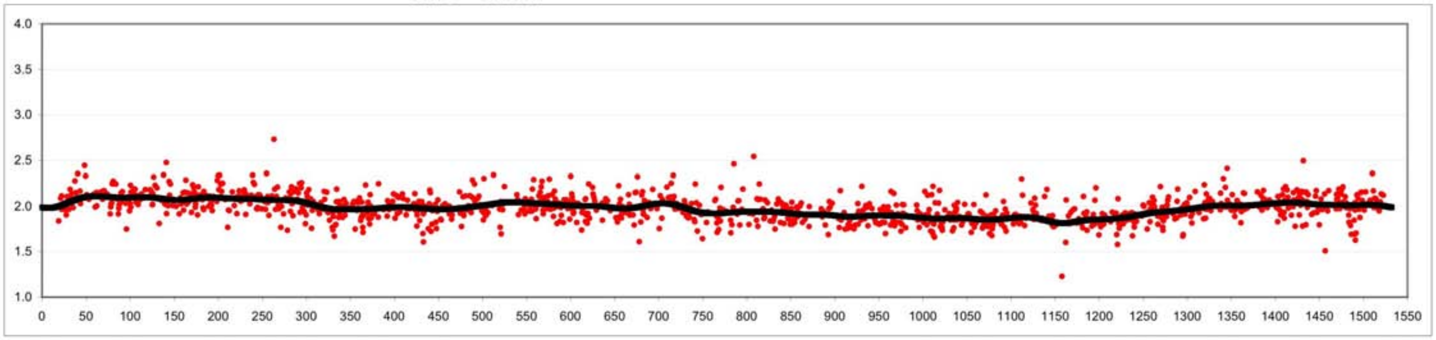

YJL8981 – Linker L12

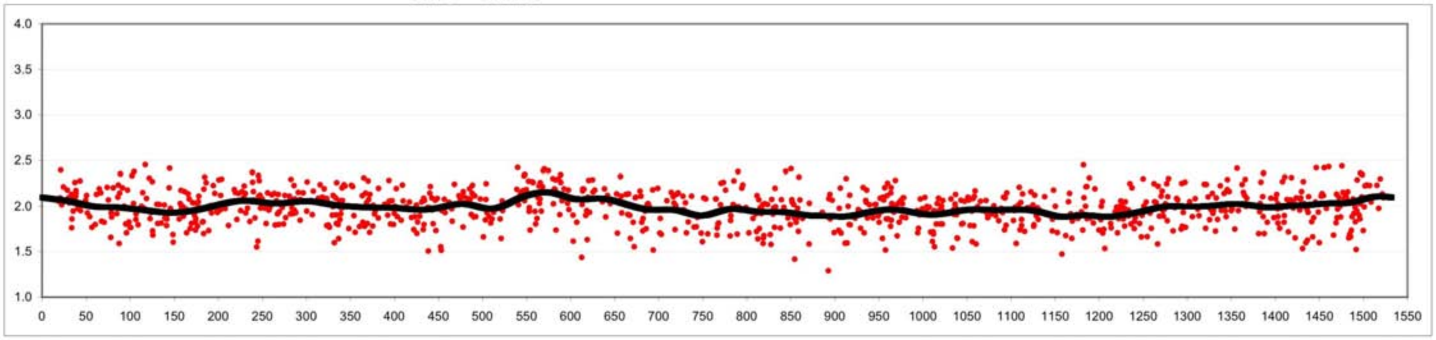

YJL8981 – Linker L12

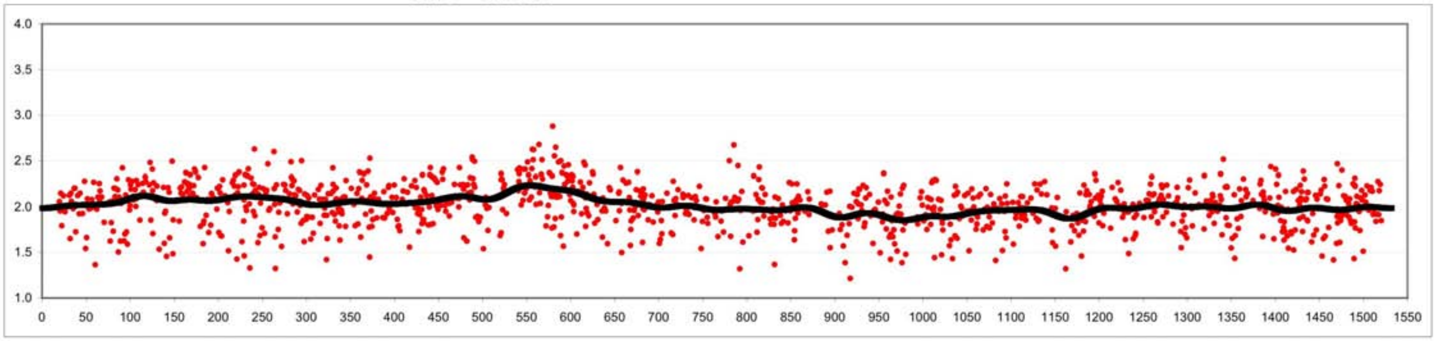

YJL8983 – Linker L14

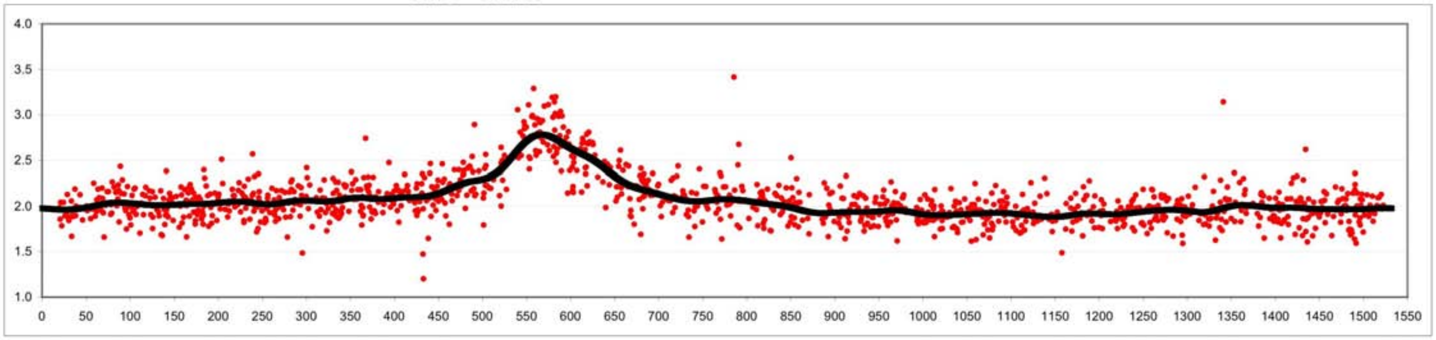

YJL8983 – Linker L14

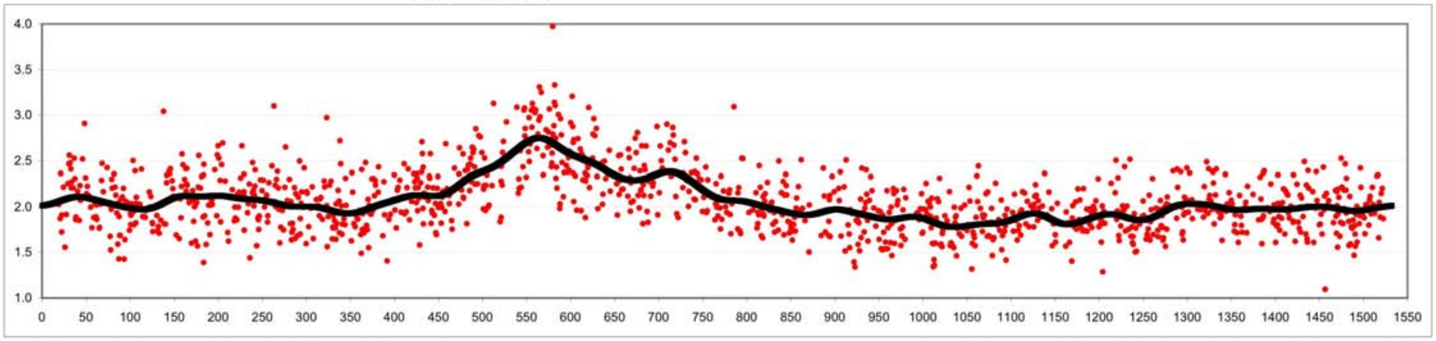

YJL9018 – Linker L16

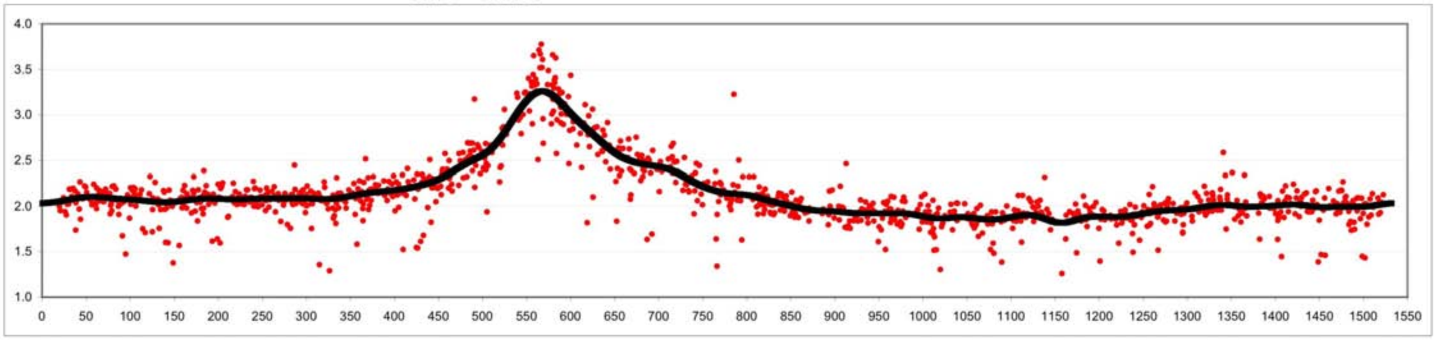

YJL9018 – Linker L16

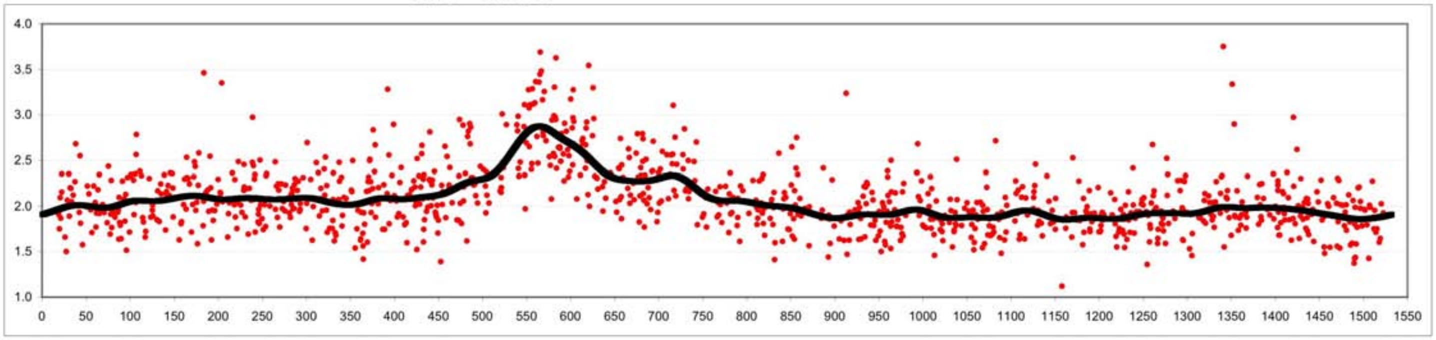

YJL8985 – Linker L18

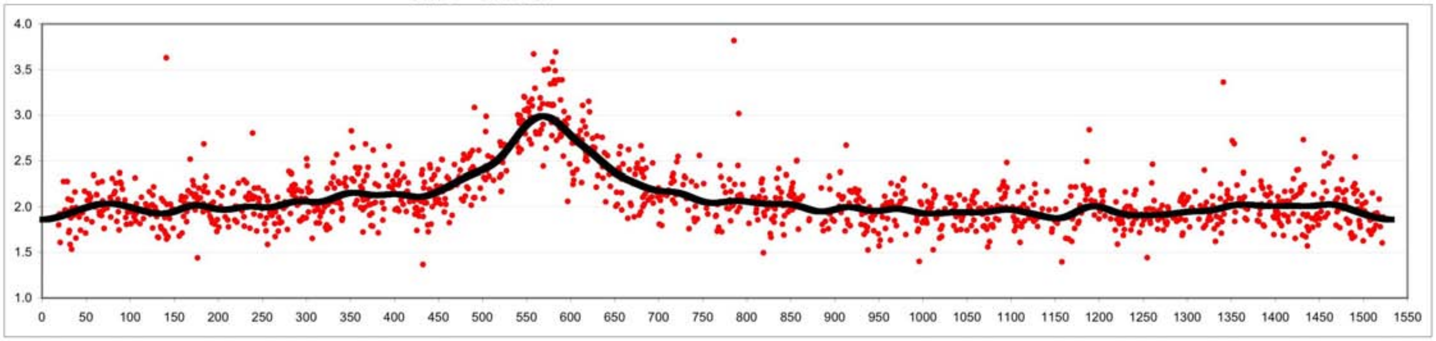

YJL8985 – Linker L18

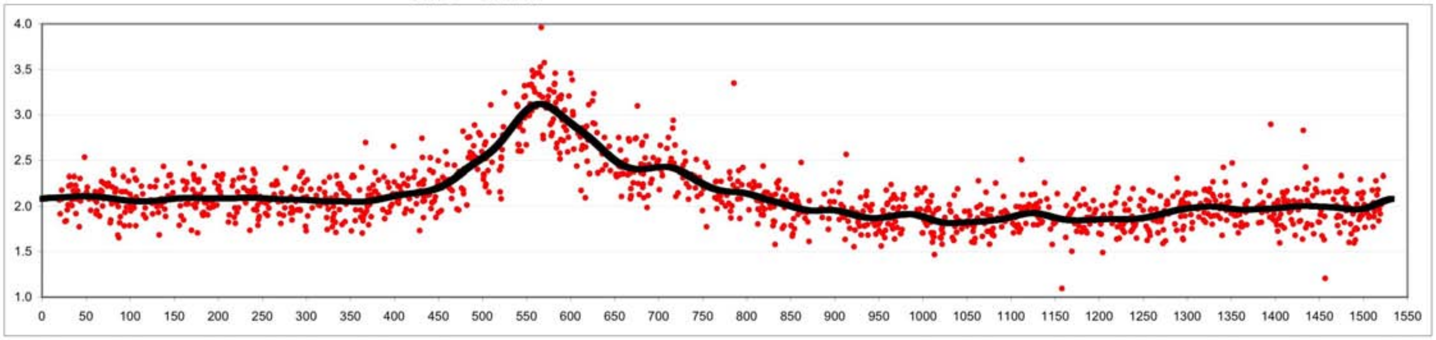

YJL8987 – Linker L20

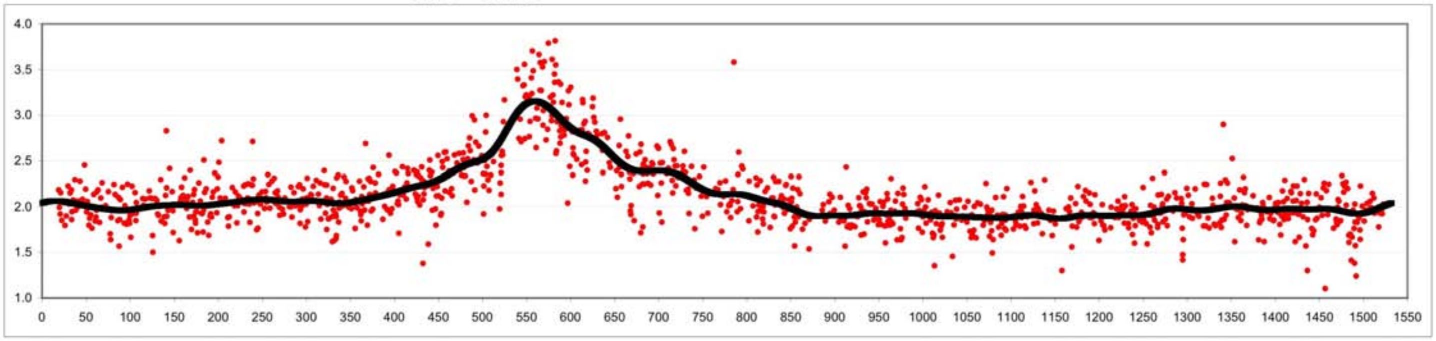

YJL8987 – Linker L20

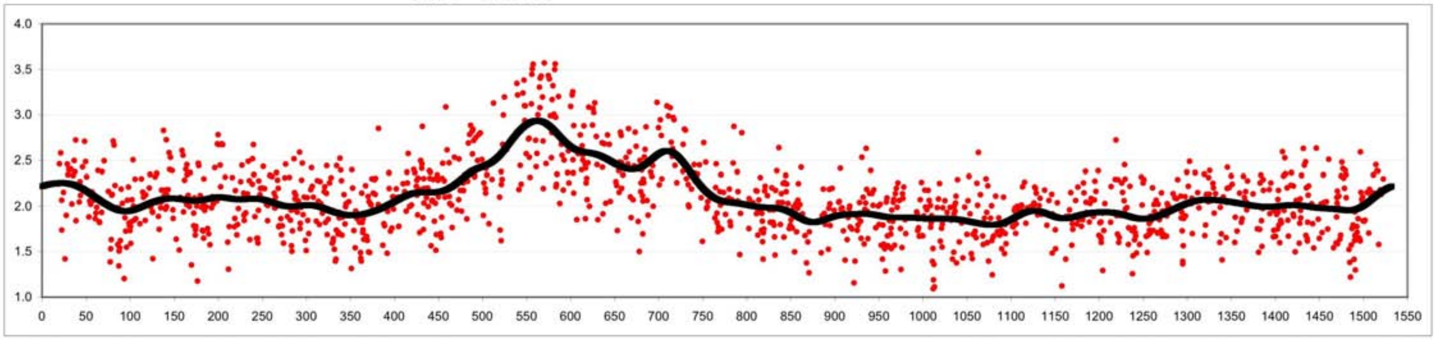

YJL8989 – Linker L22

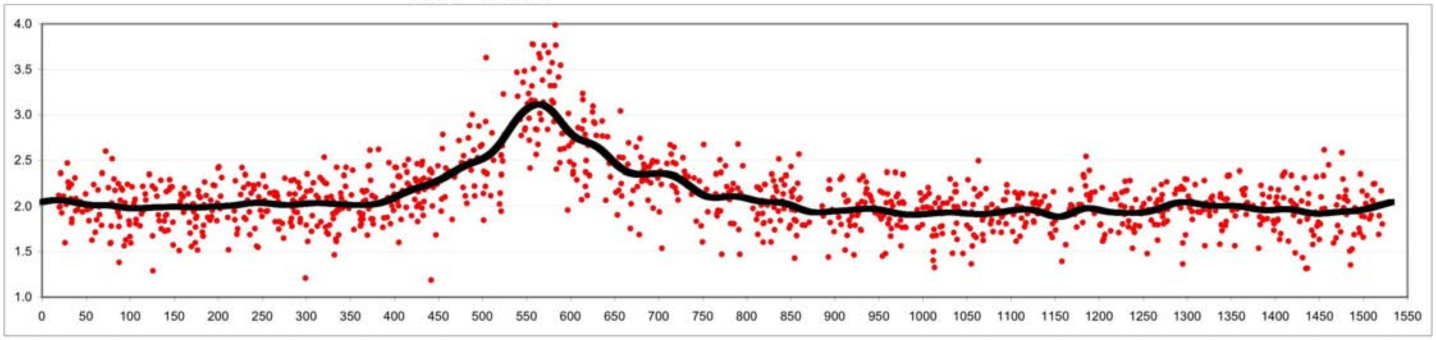

YJL8989 – Linker L22

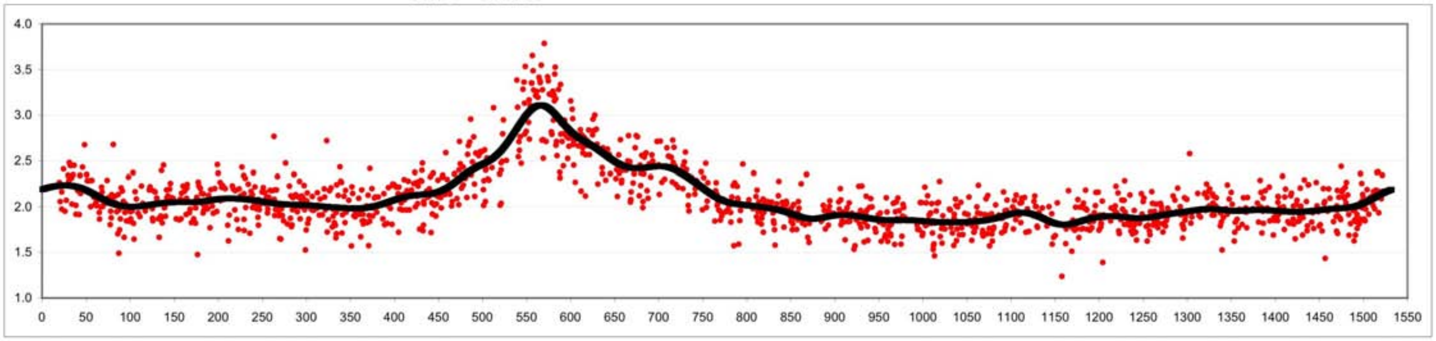

YJL9020 – Linker L24

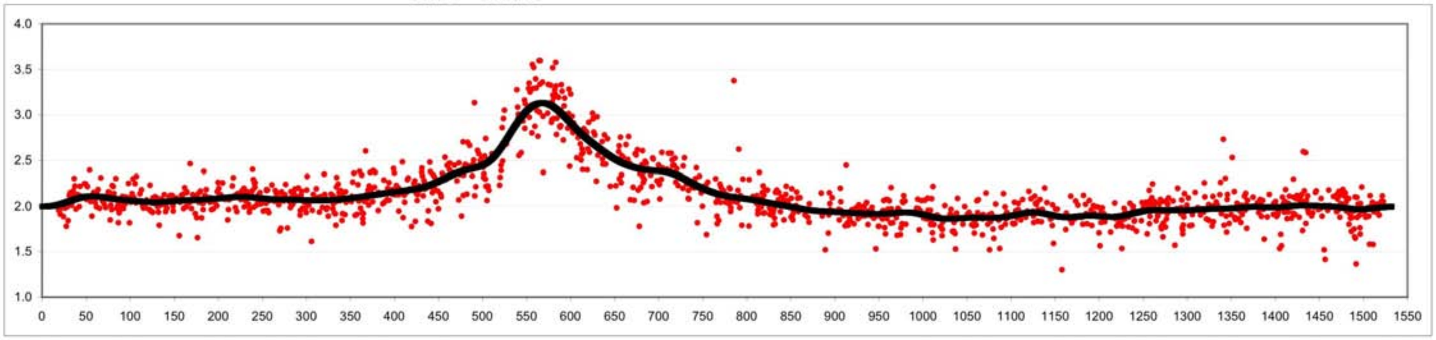

YJL9020 – Linker L24

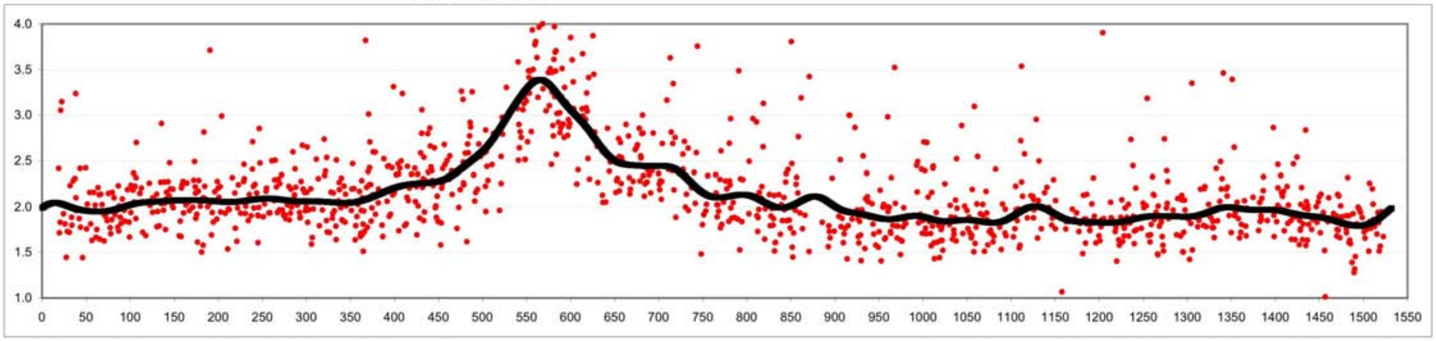

YJL8991 – Linker L26

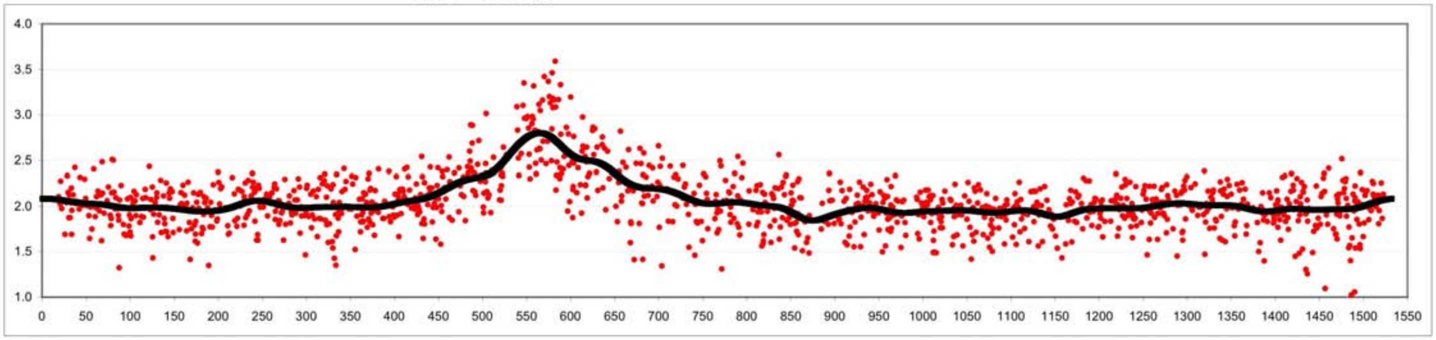

YJL8991 – Linker L26

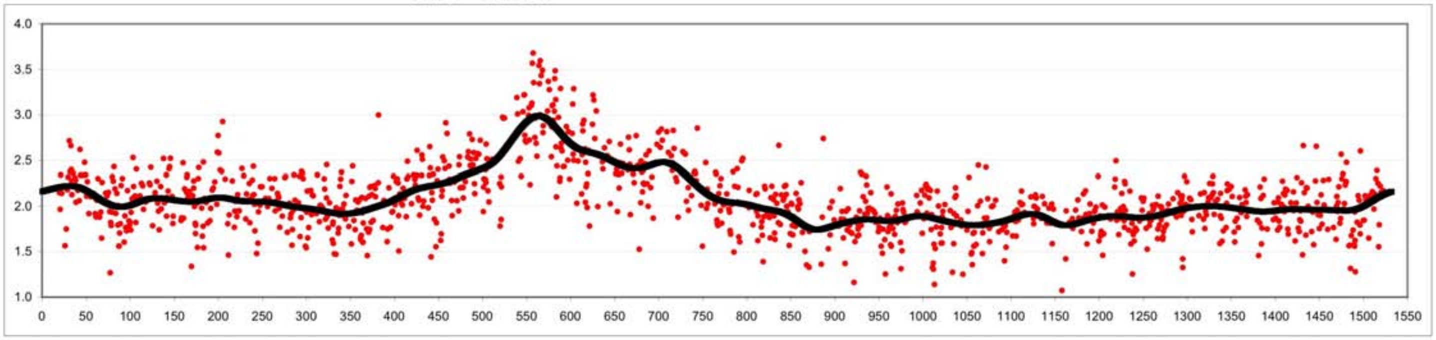

YJL8993 – Linker L28

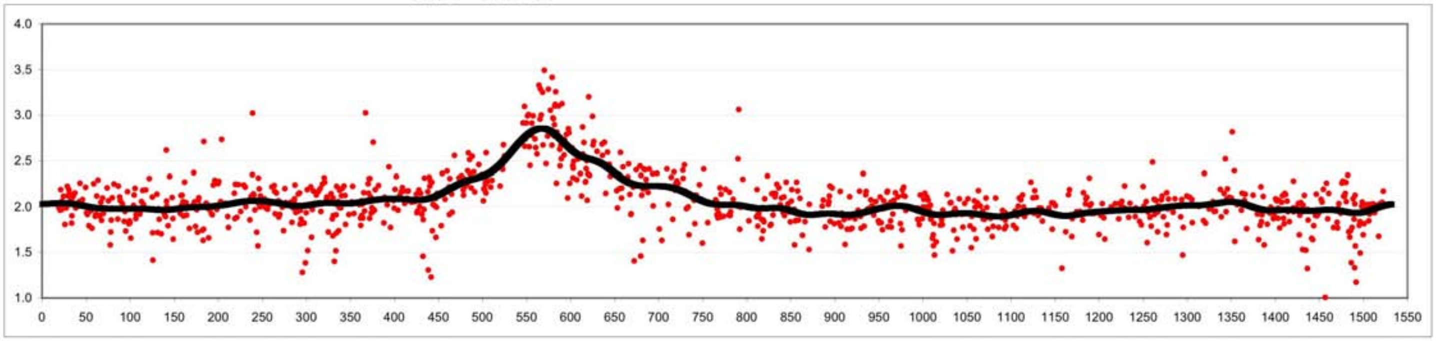

YJL8993 – Linker L28

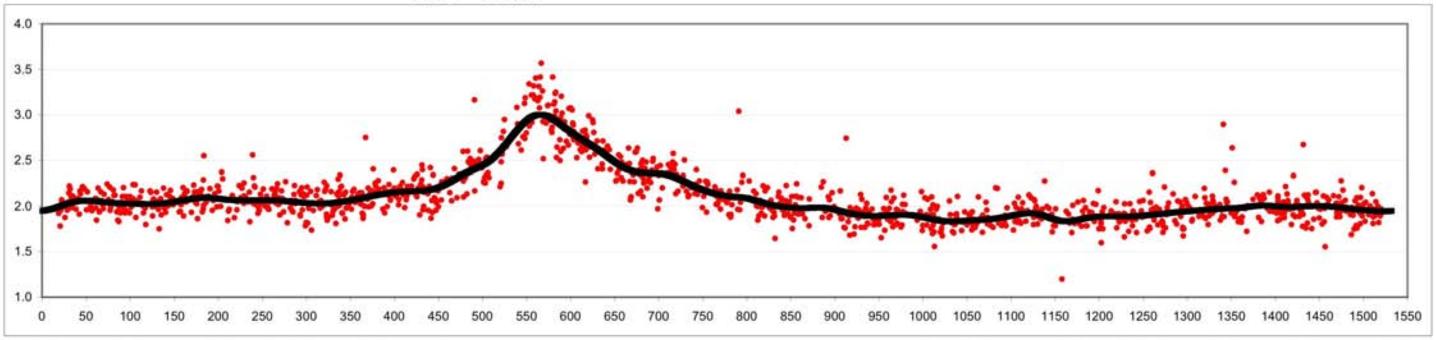

YJL9022 – Linker L30

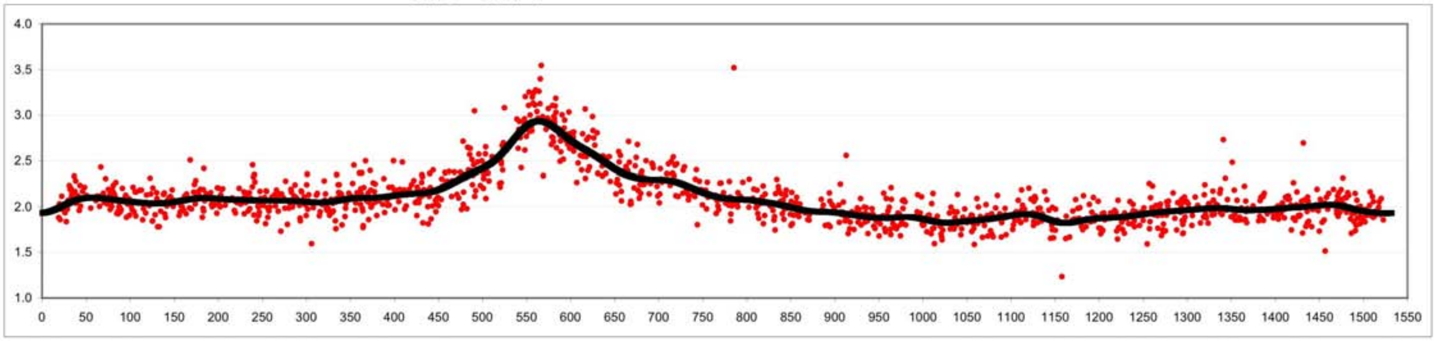

YJL9022 – Linker L30

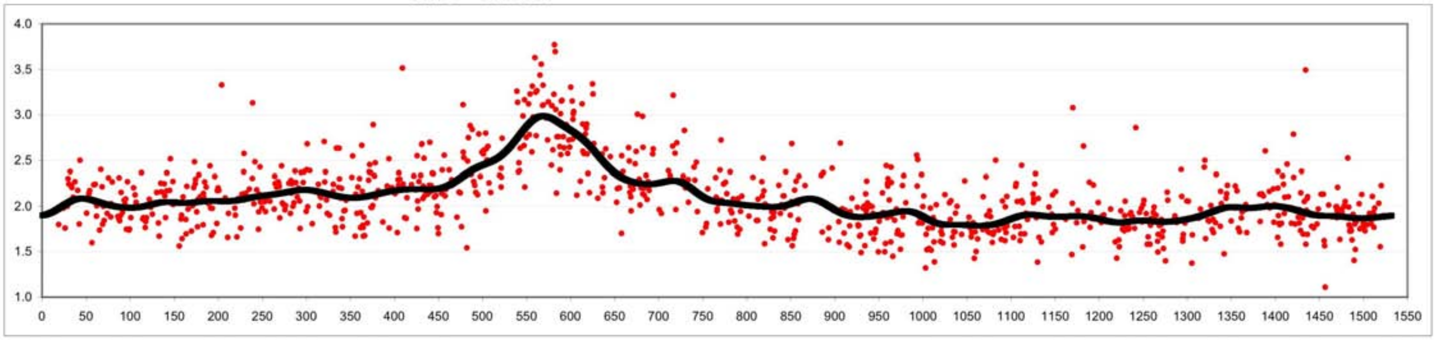

YJL8995 – Linker L32

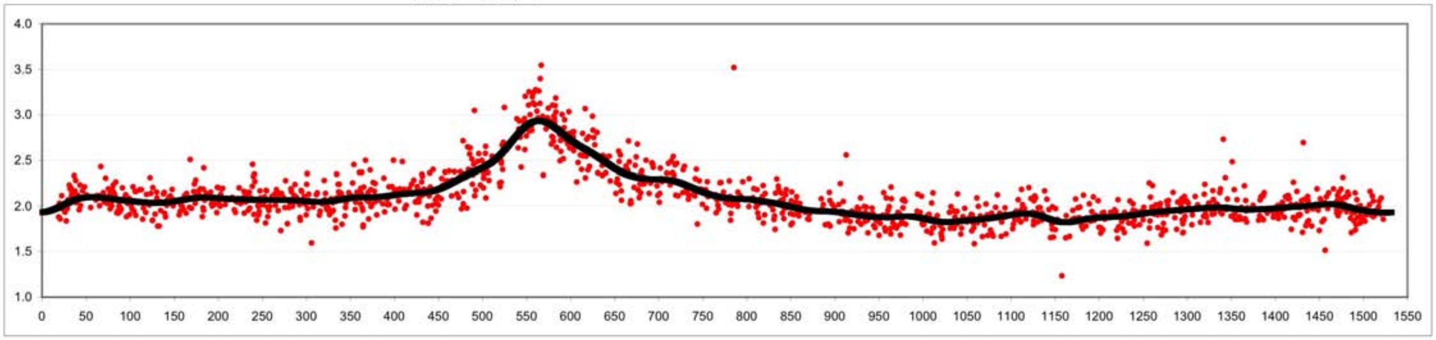

YJL8995 – Linker L32

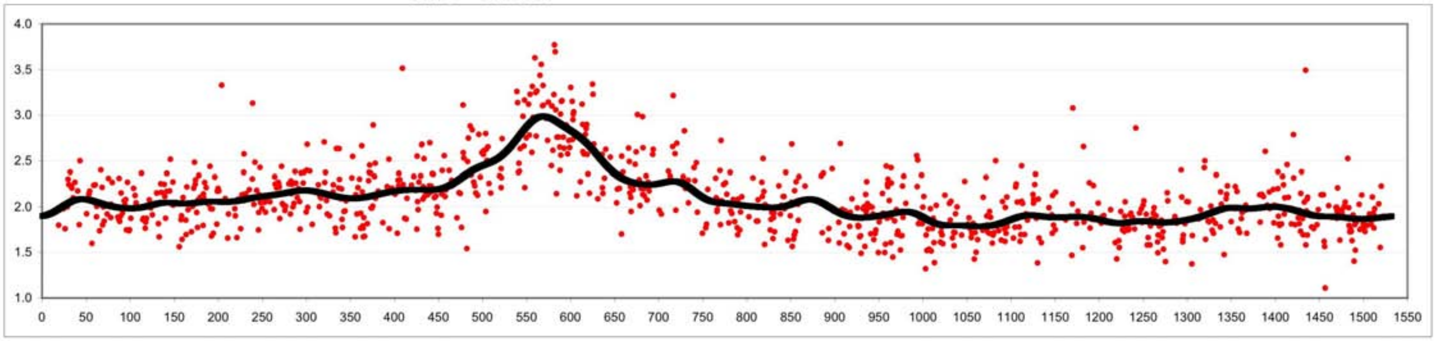

Figure 5A

YJL8398 – Control Strain

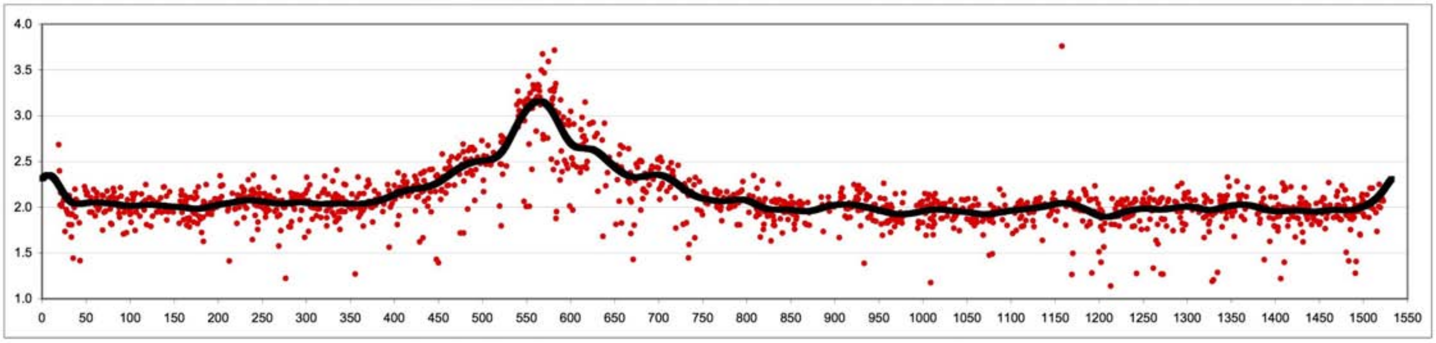

YJL8398 – Control Strain

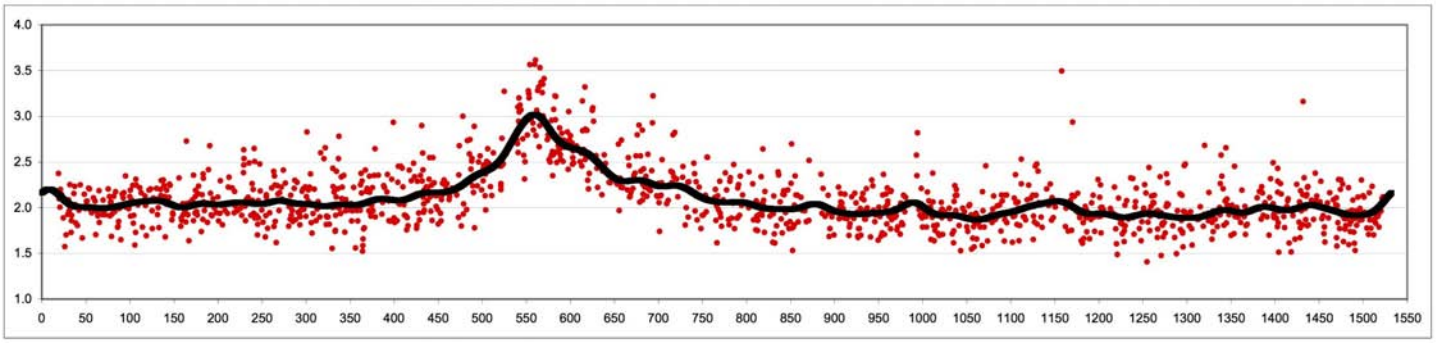

YJL8779 – 58bp spacing

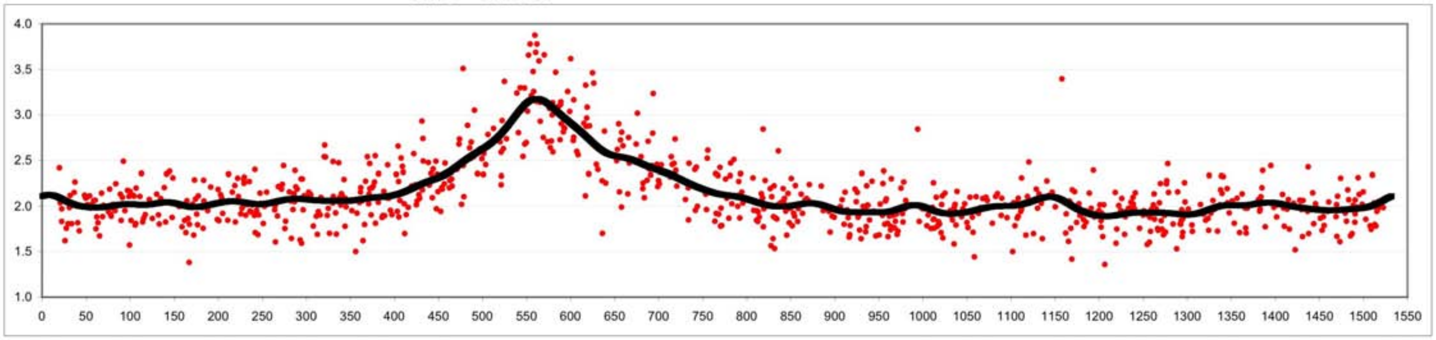

YJL8779 – 58bp spacing

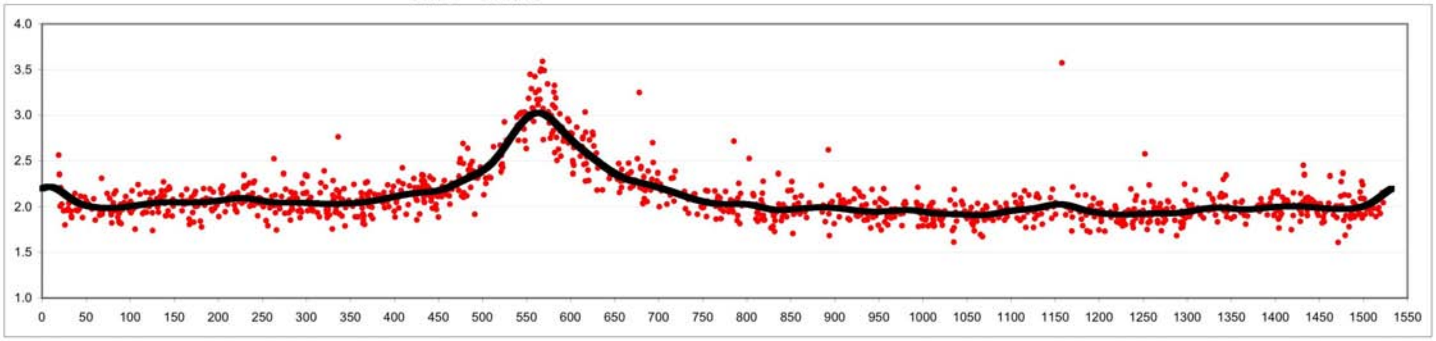

YJL8781 – 63bp spacing

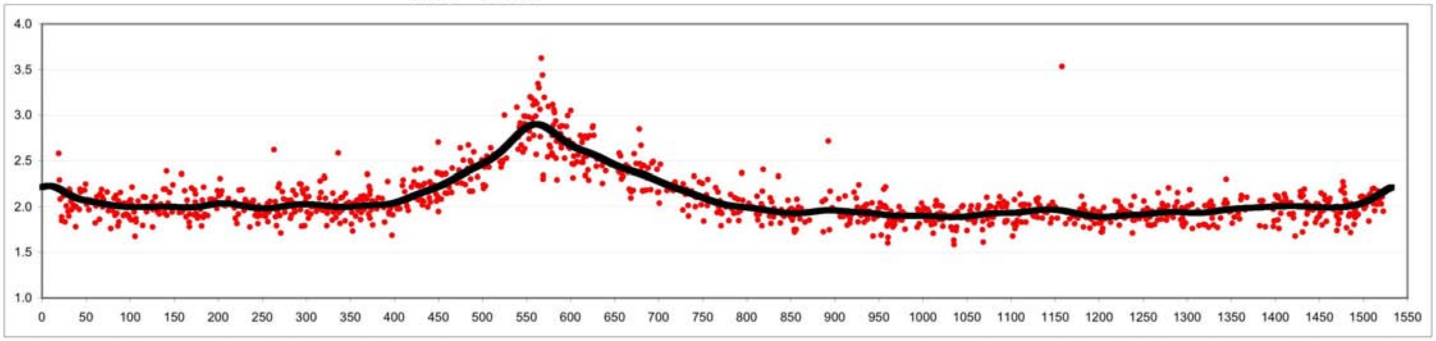

YJL8781 – 63bp spacing

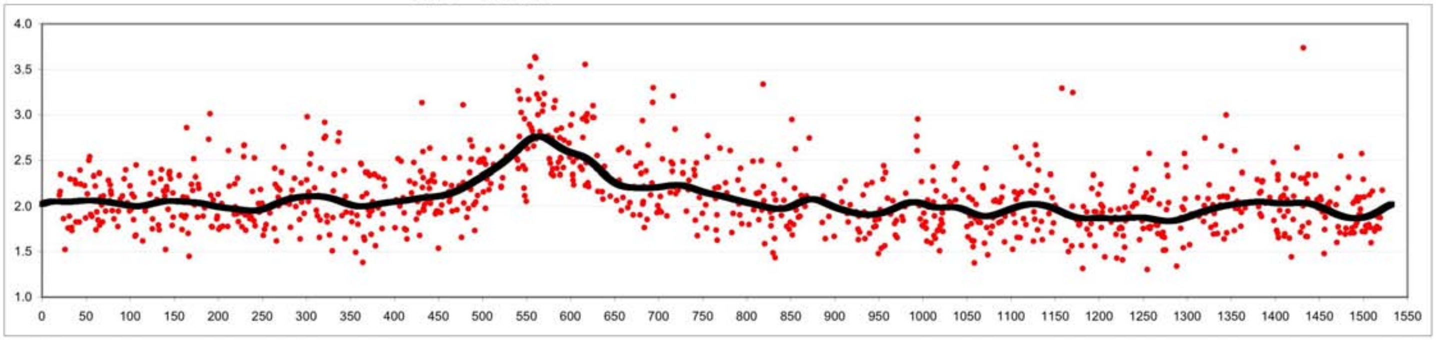

YJL8783 – 73bp spacing

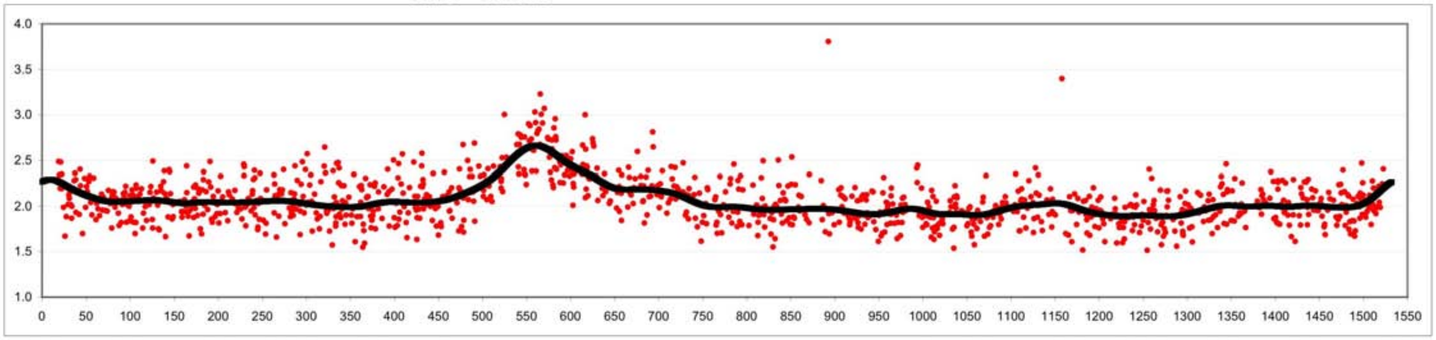

YJL8783 – 73bp spacing

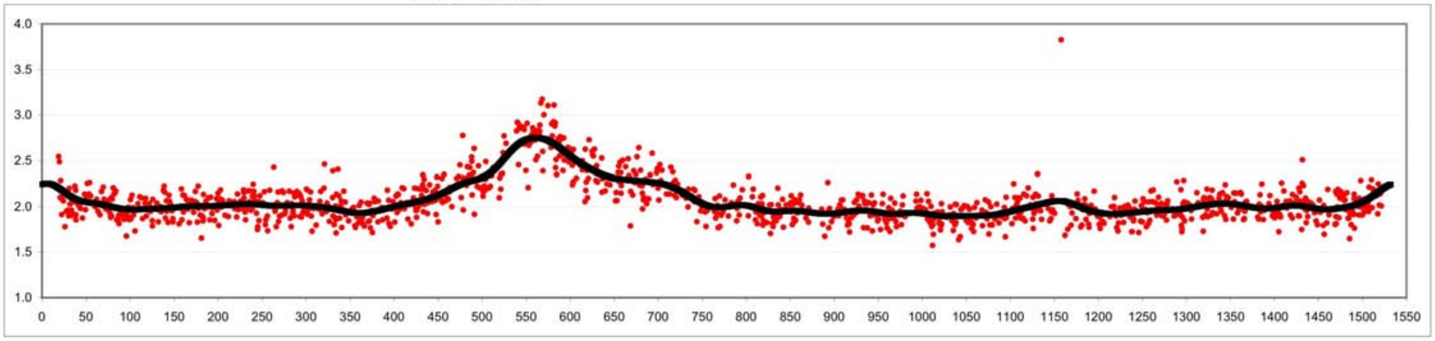

YJL8785 – 153bp spacing

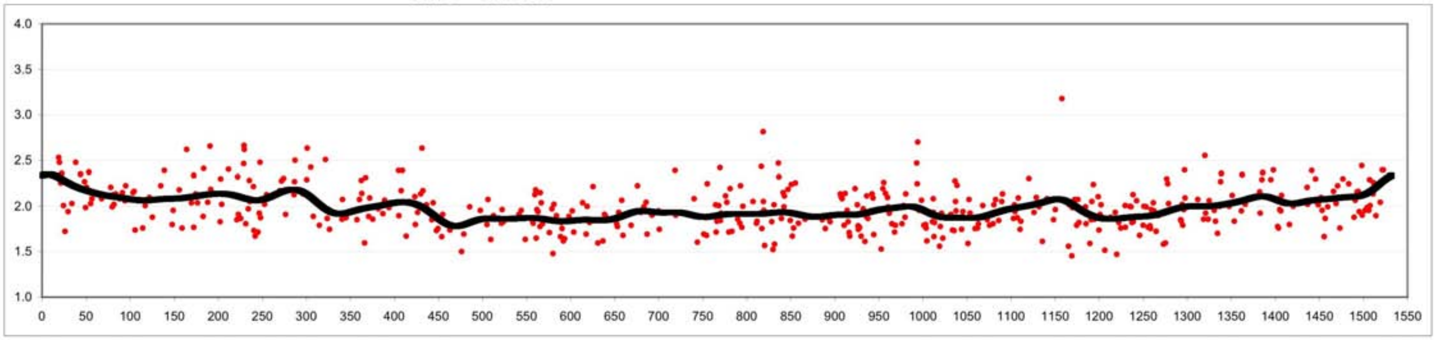

YJL8785 – 153bp spacing

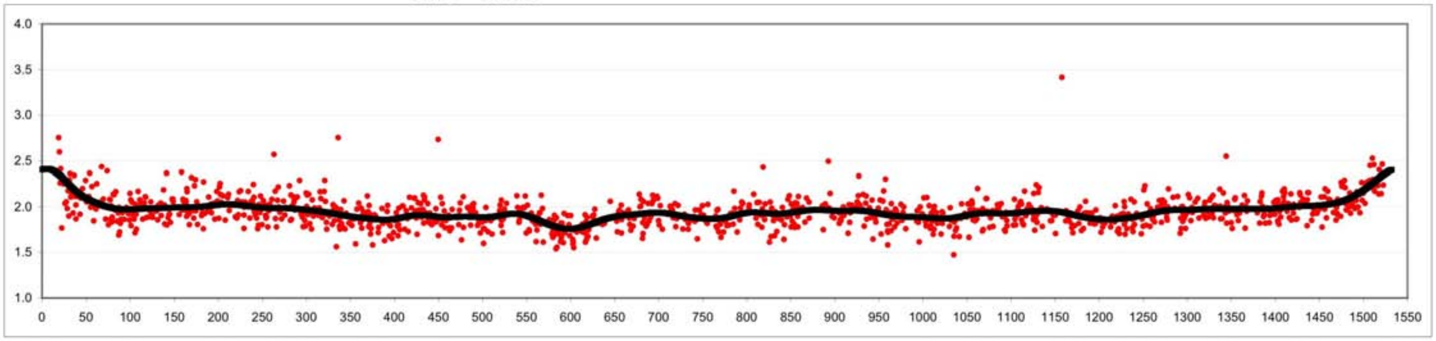

YJL8908 – 21bp spacing

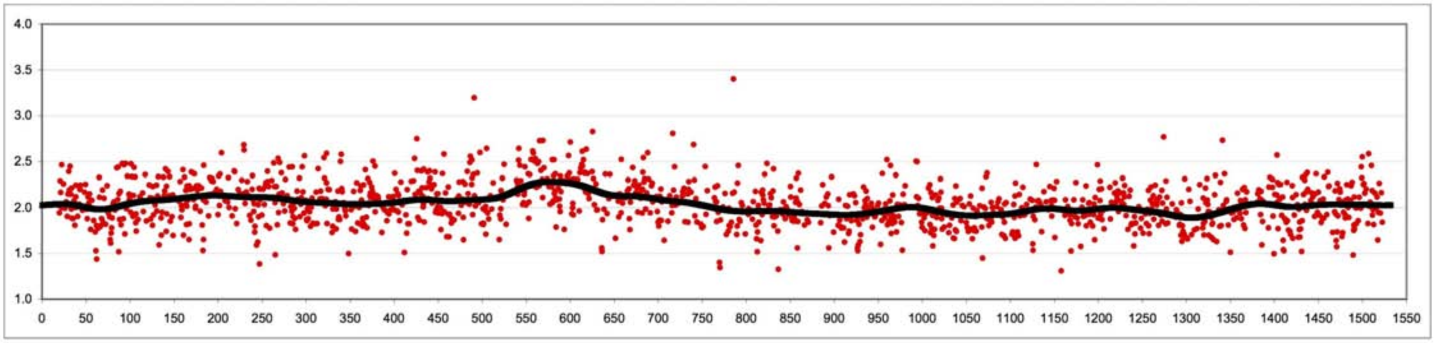

YJL8908 – 21bp spacing

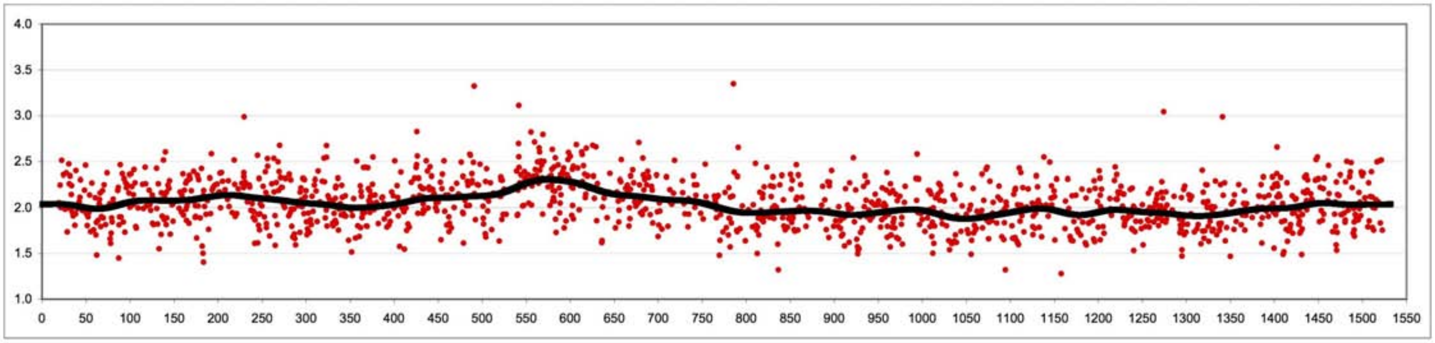

YJL8910 – 37bp spacing

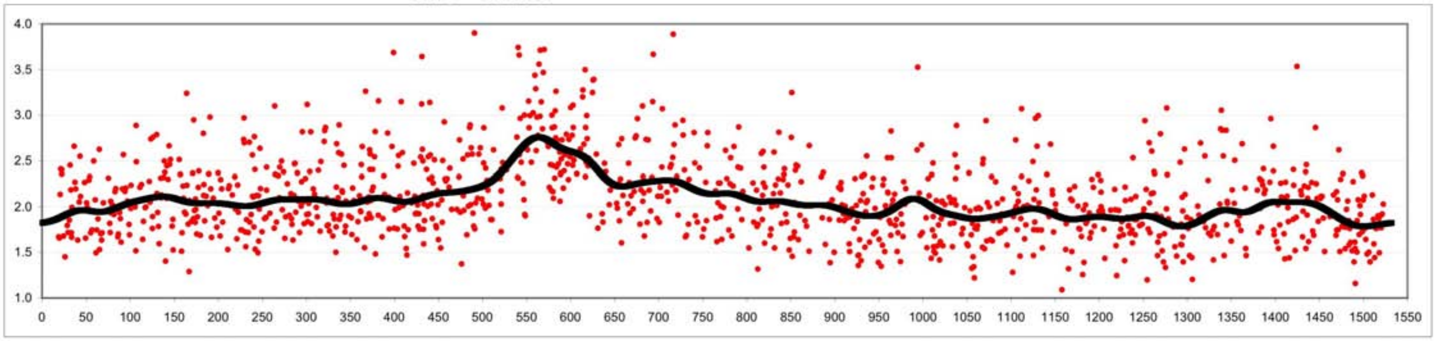

YJL8910 – 37bp spacing

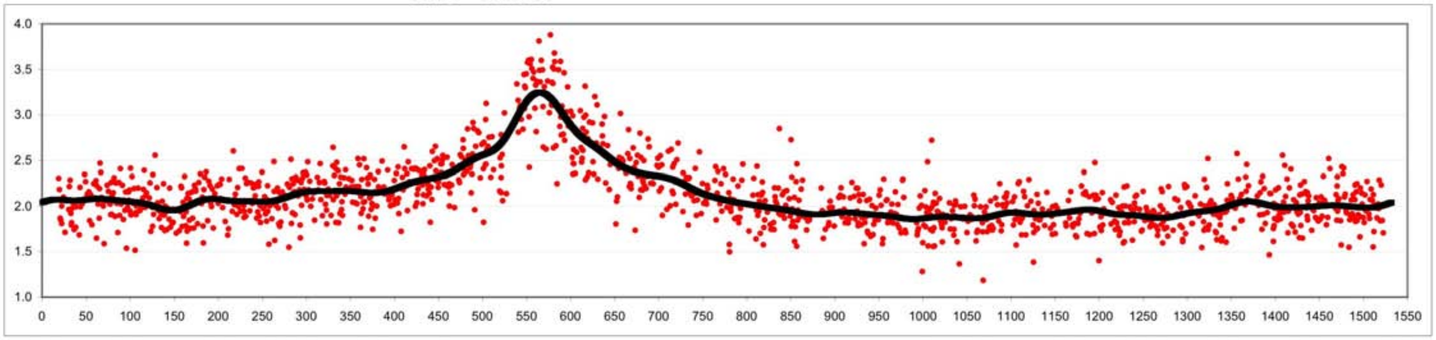

YJL8912 – 45bp spacing

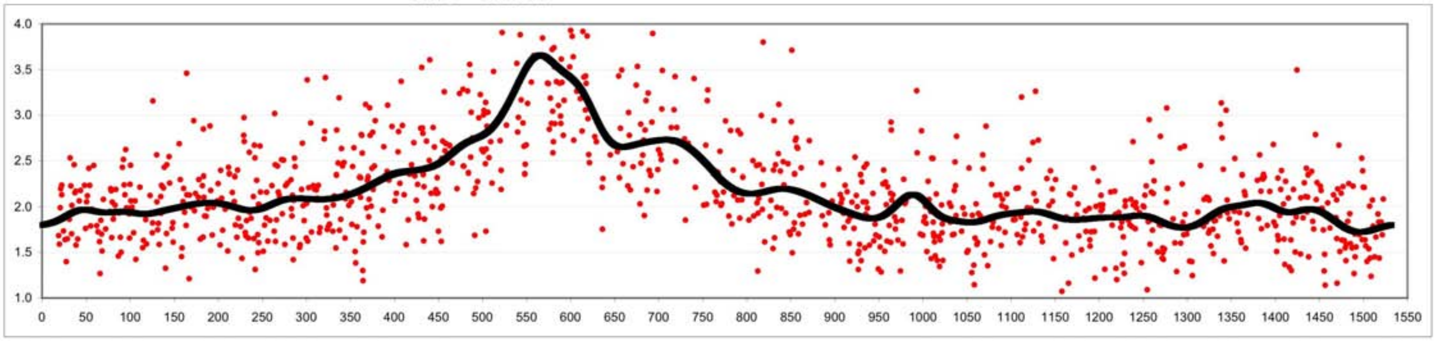

YJL8912 – 45bp spacing

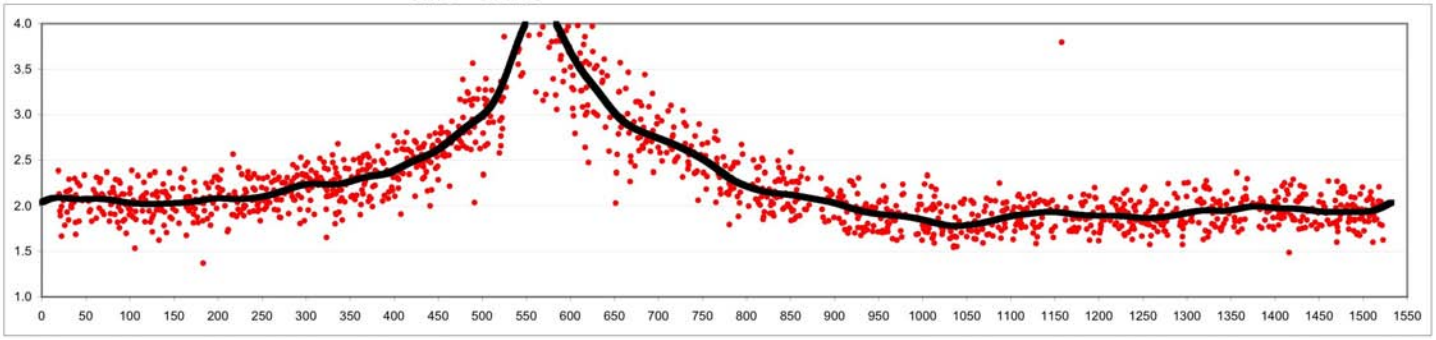

Figure 5B
YJL9566 – Control

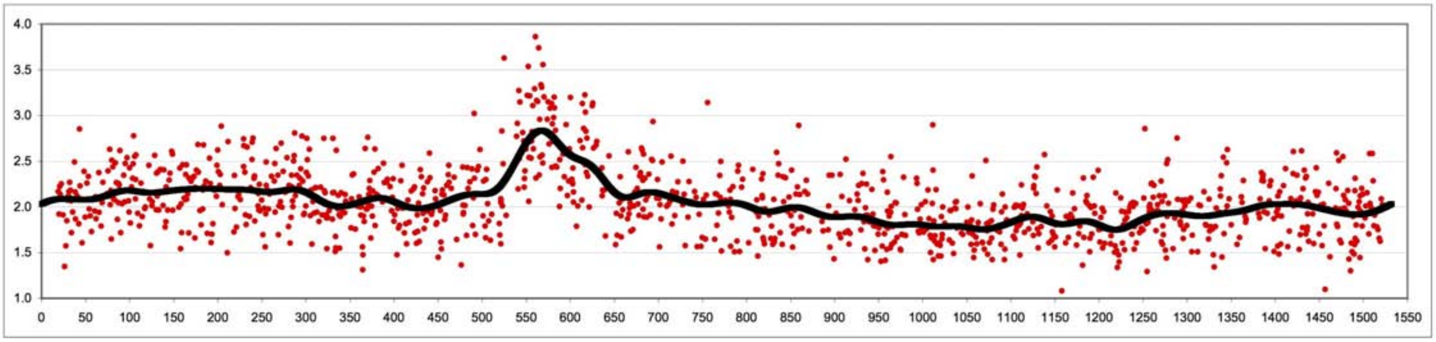

YJL9567 – Control

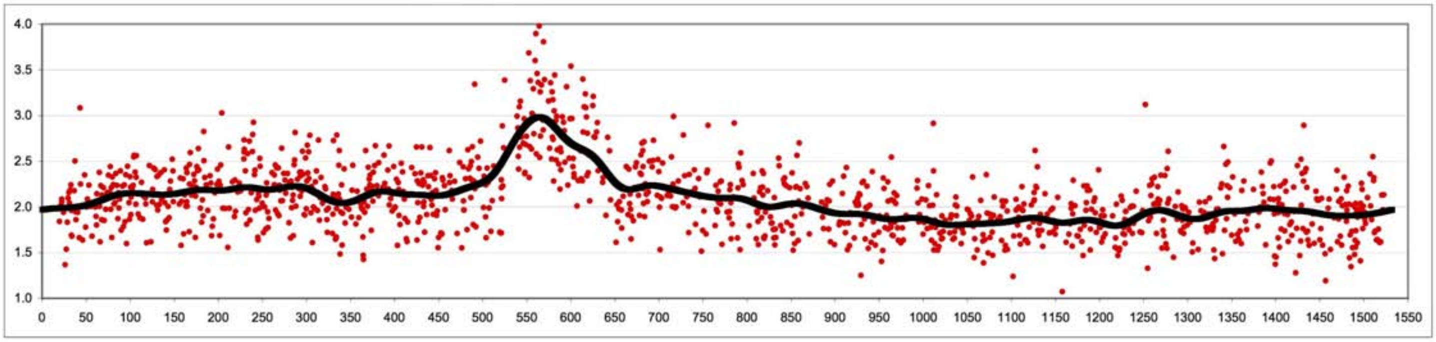

YJL10158 – 53bp spacing

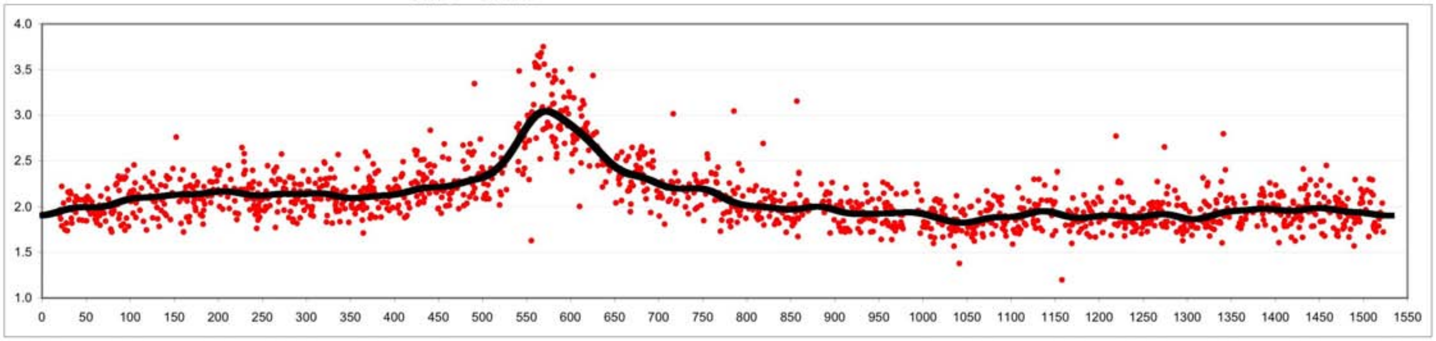

YJL10159 – 53bp spacing

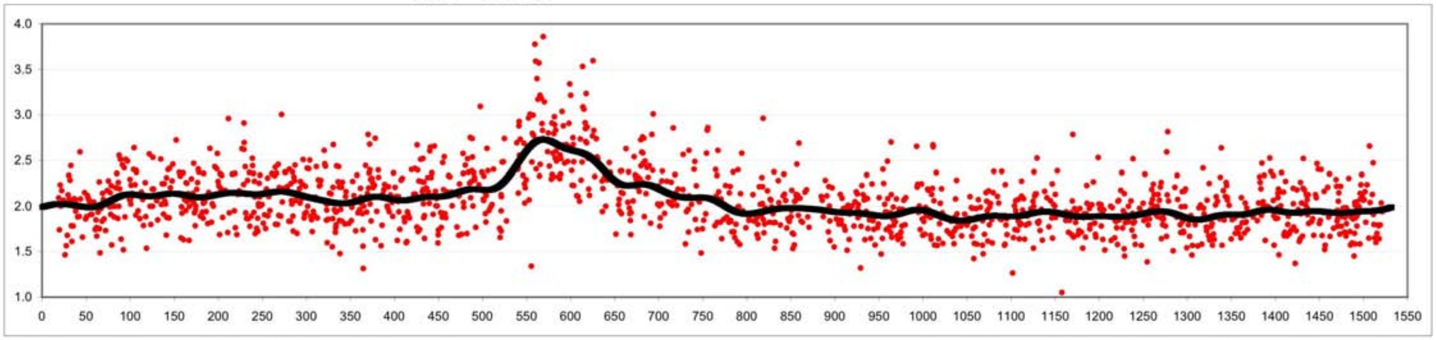

YJL10287 – 153bp spacing

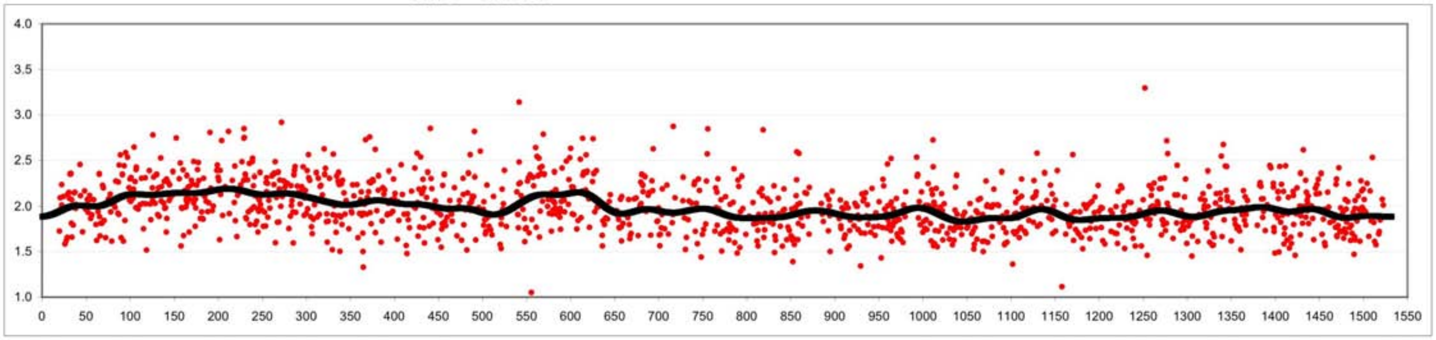

YJL10288 – 153bp spacing

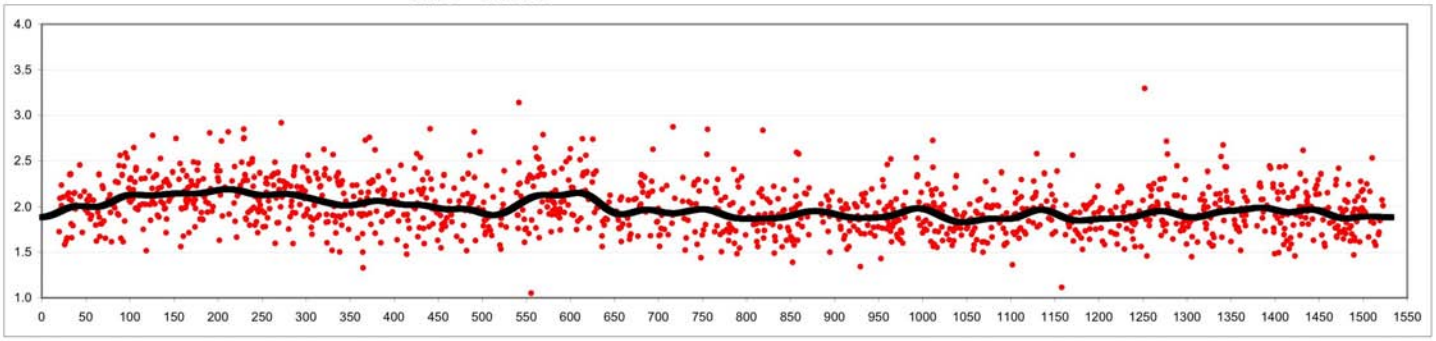

YJL10289 – 73bp spacing

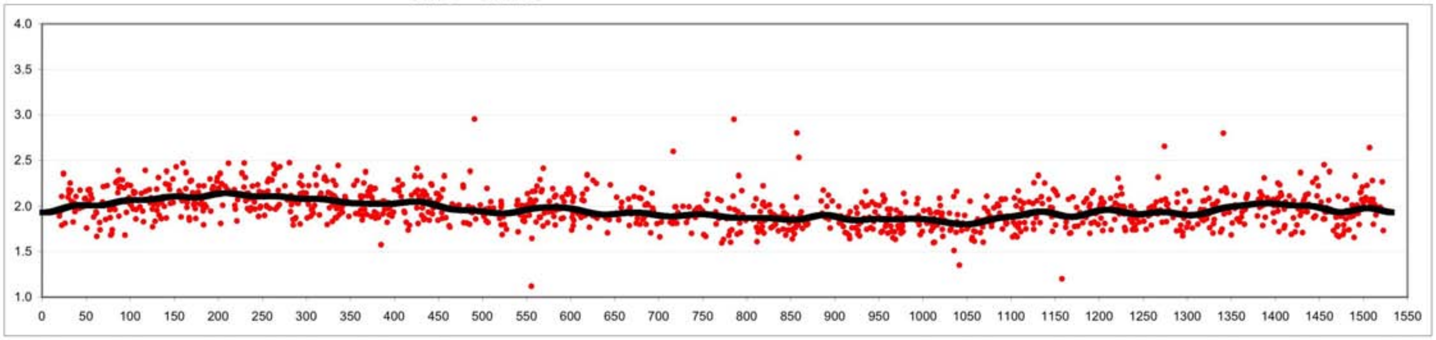

YJL10290 – 73bp spacing

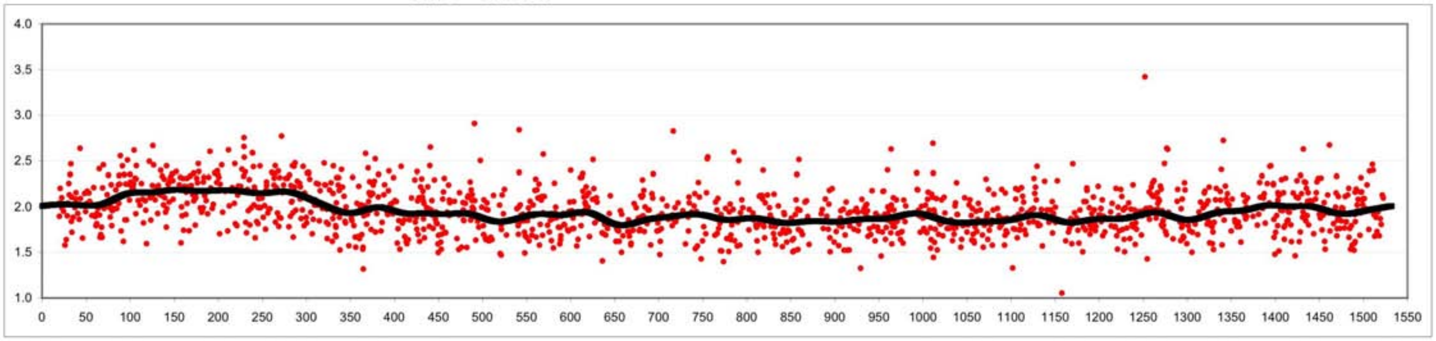

YJL10291 – 63bp spacing

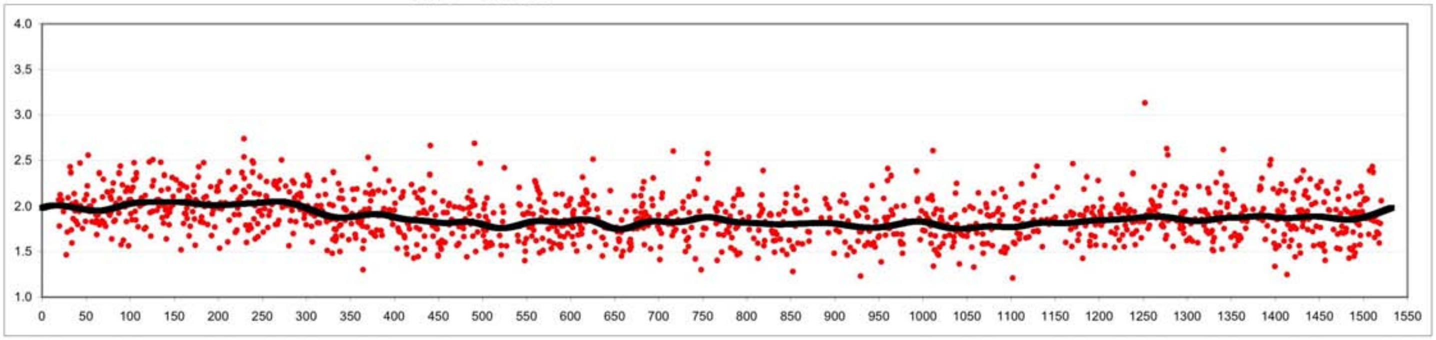

YJL10292 – 63bp spacing

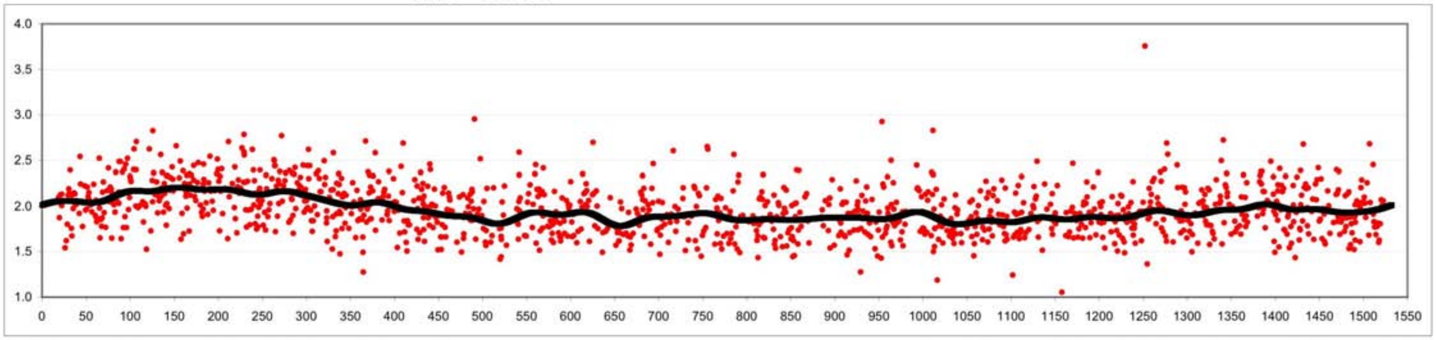

YJL10293 – 58bp spacing

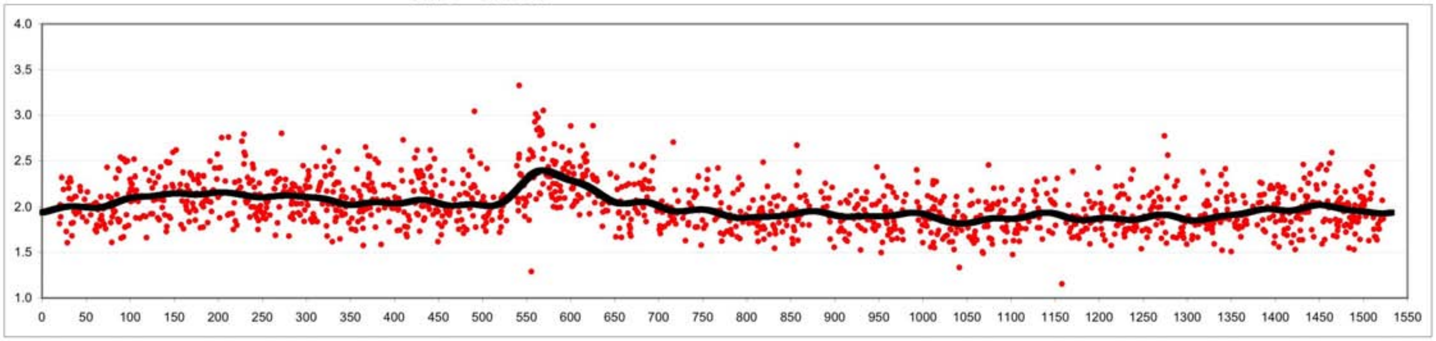

YJL10294 – 58bp spacing

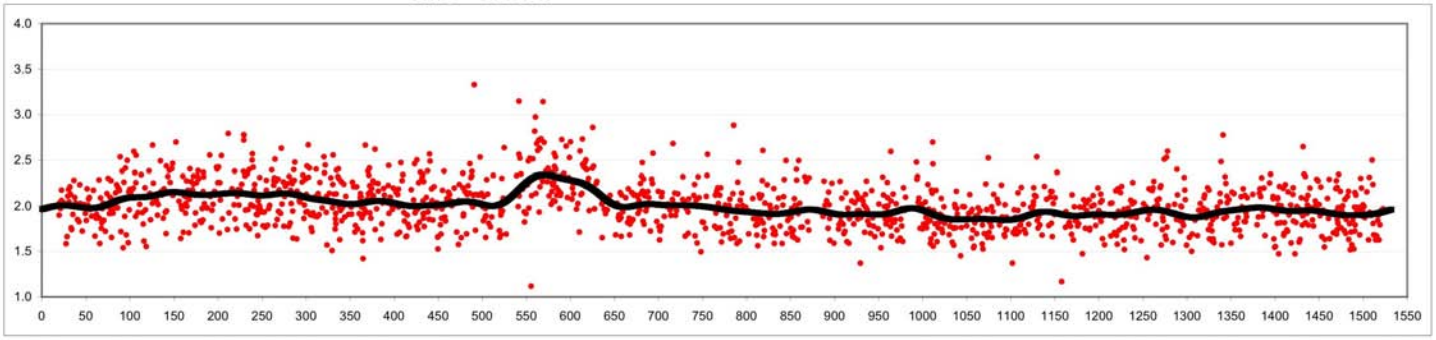

YJL10295 – 45bp spacing

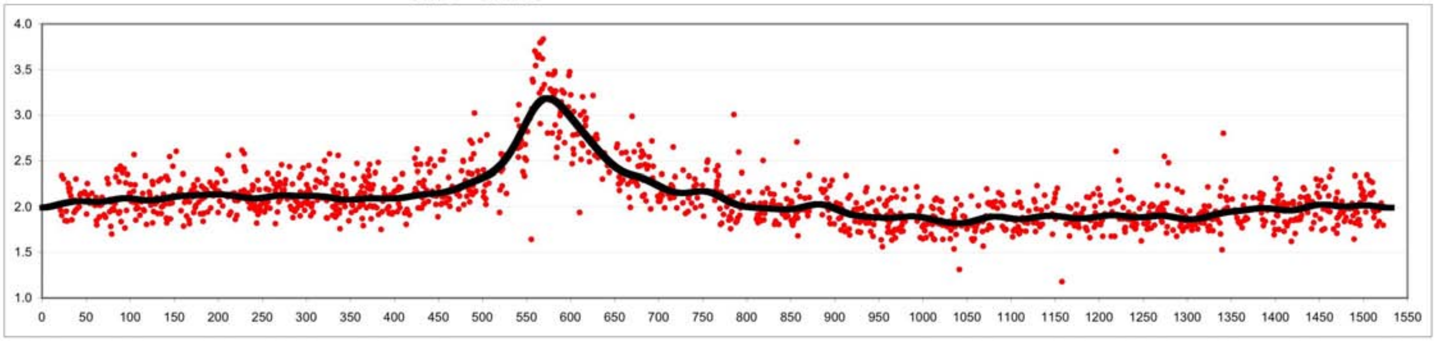

YJL10296 – 45bp spacing

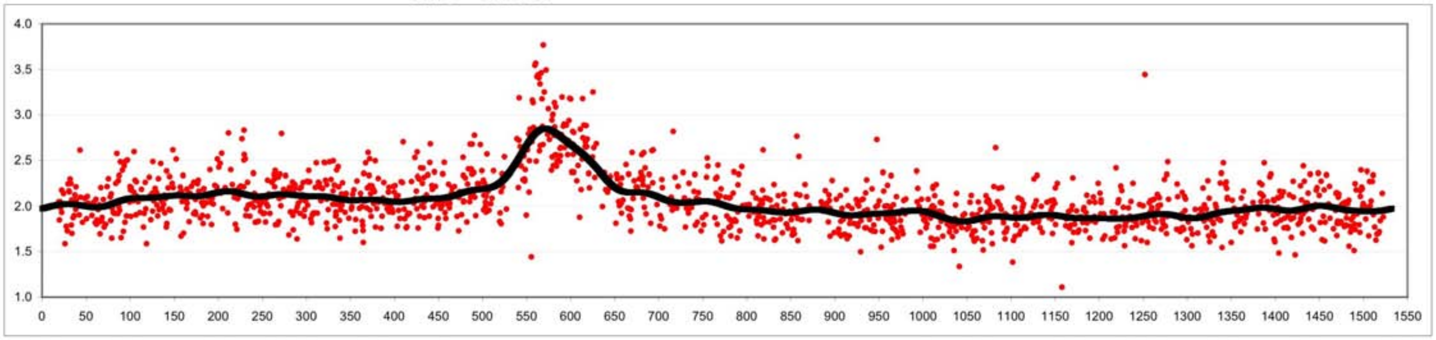

YJL10297 – 27bp spacing

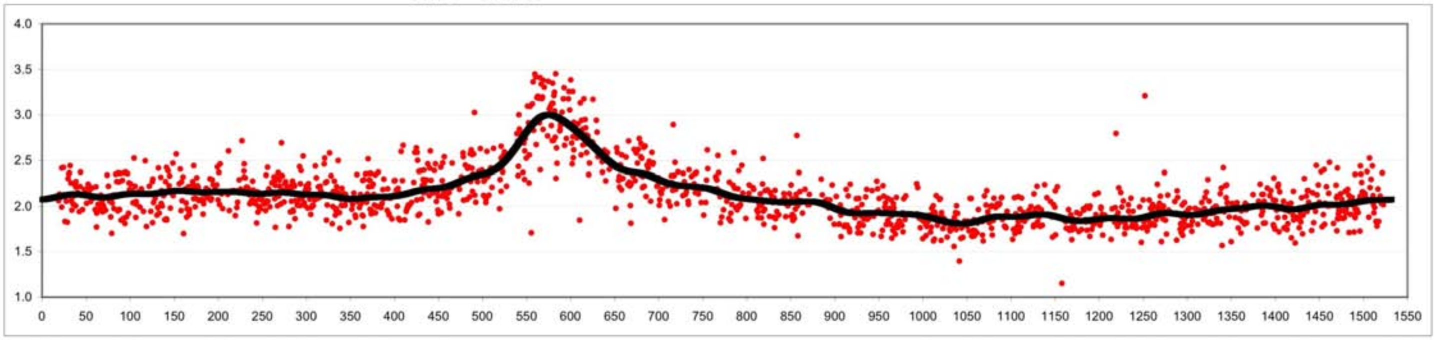

YJL10298 – 27bp spacing

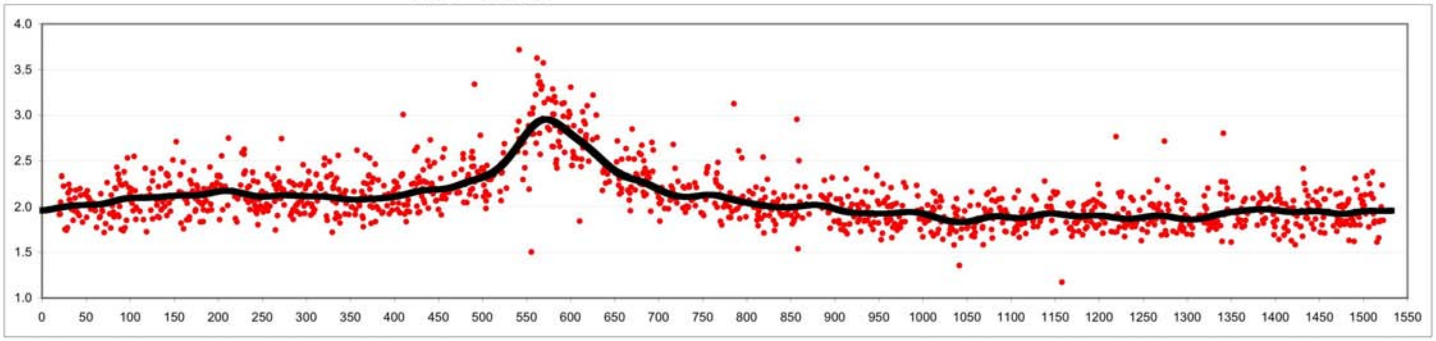

YJL10299 – 21bp spacing

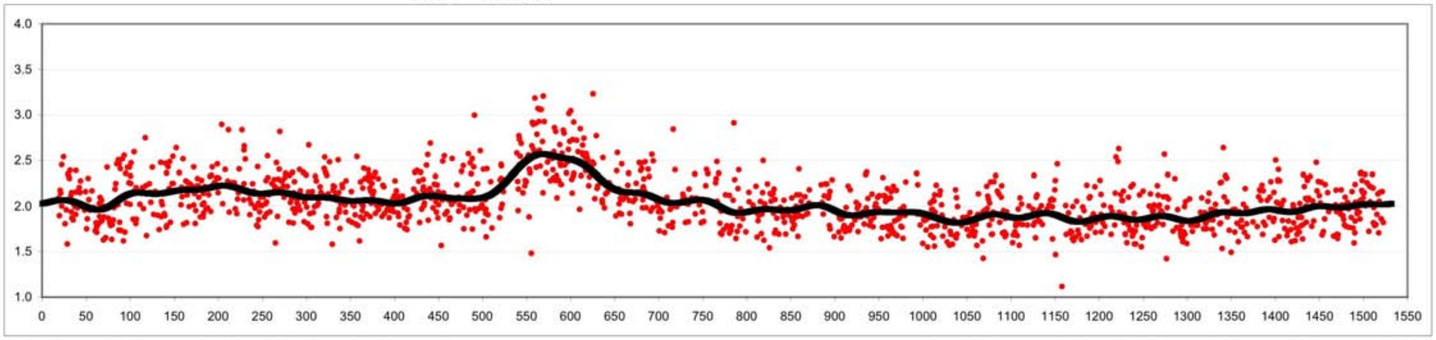

YJL10300 – 21bp spacing

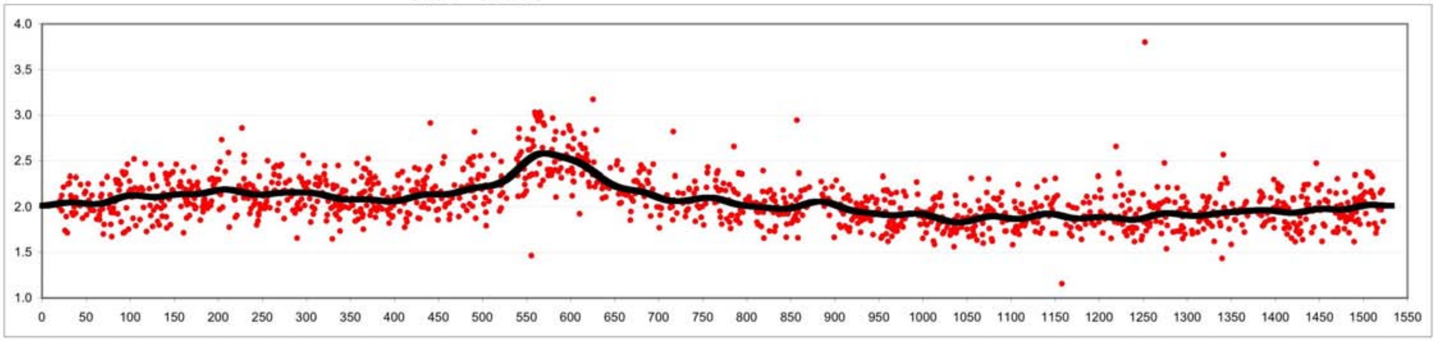

Figure 6A
YJL9078

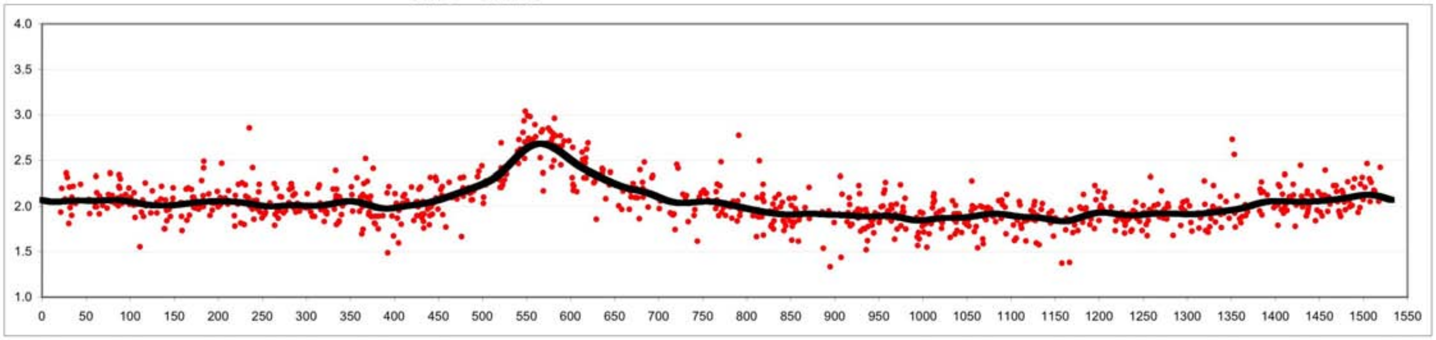

YJL9078

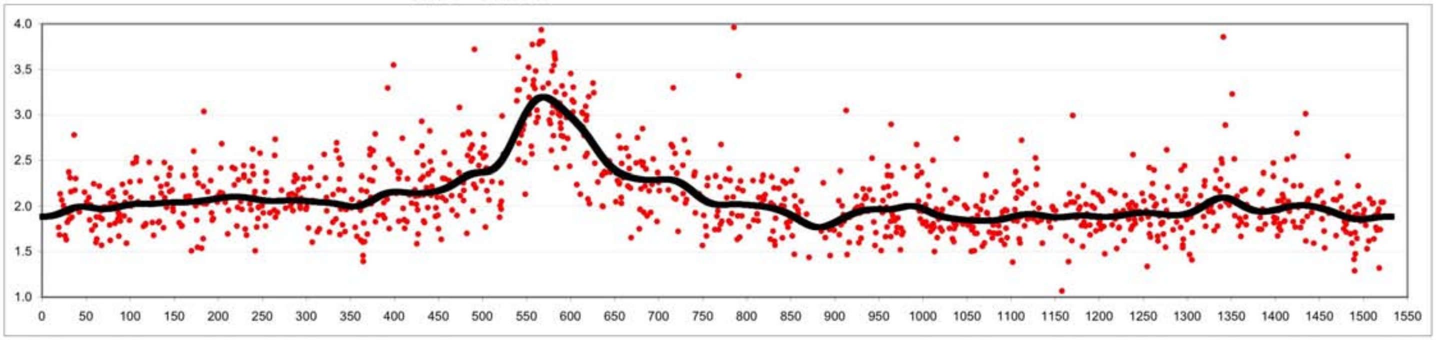

YJL9080

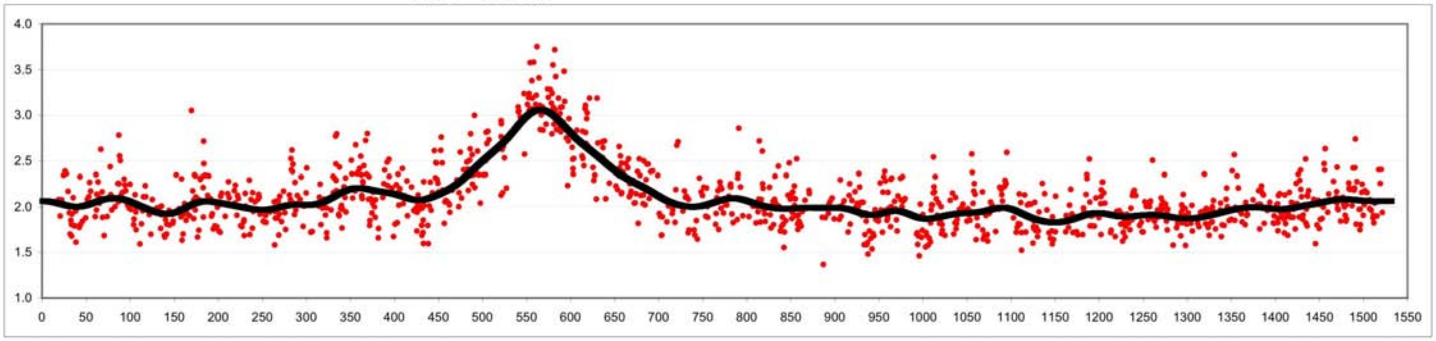

YJL9080

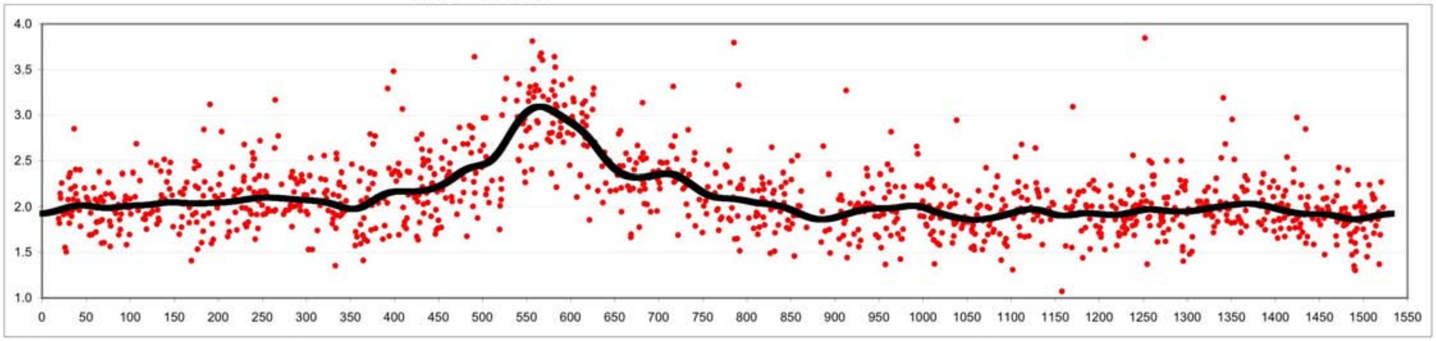

YJL9221

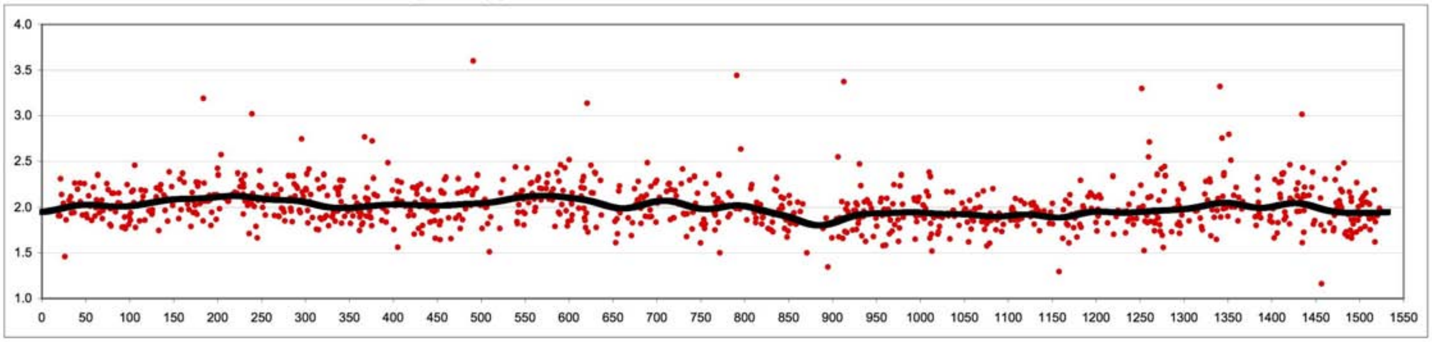

YJL9221

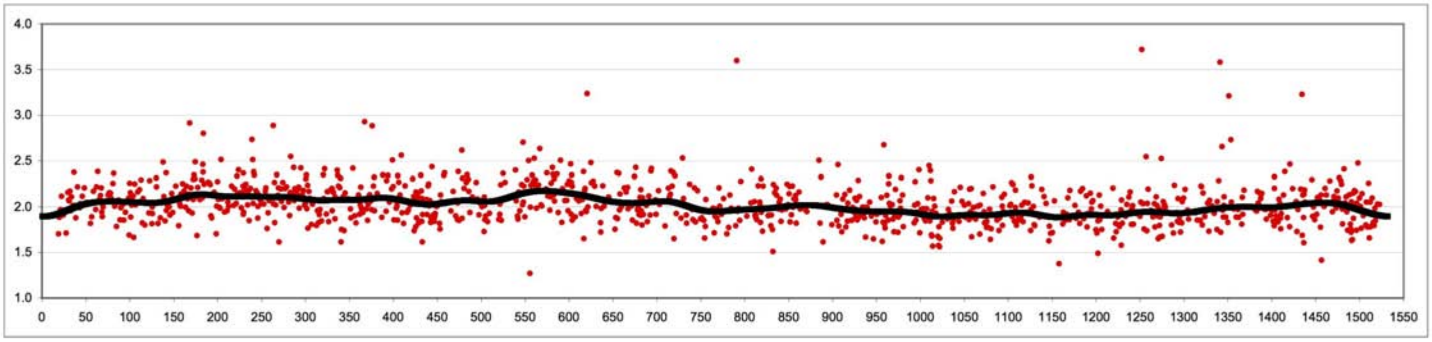

YJL9225

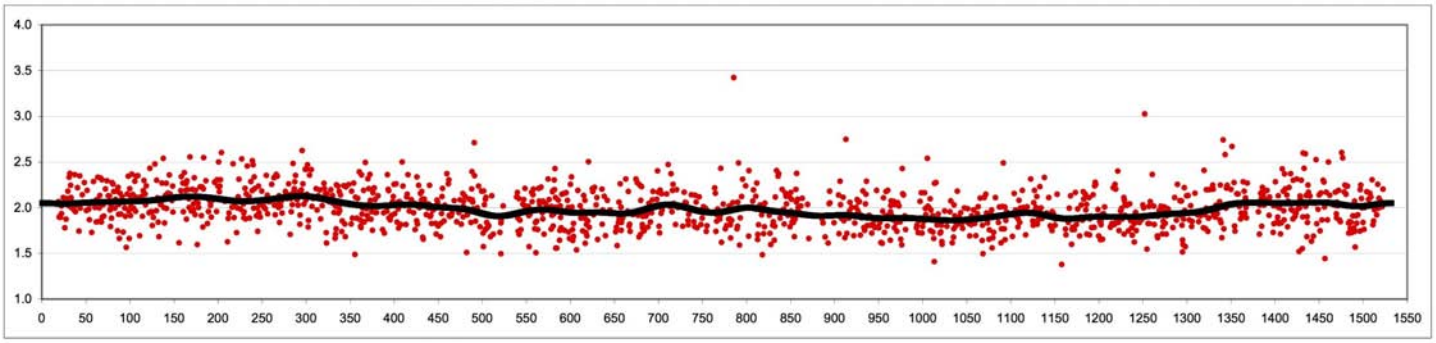

YJL9225

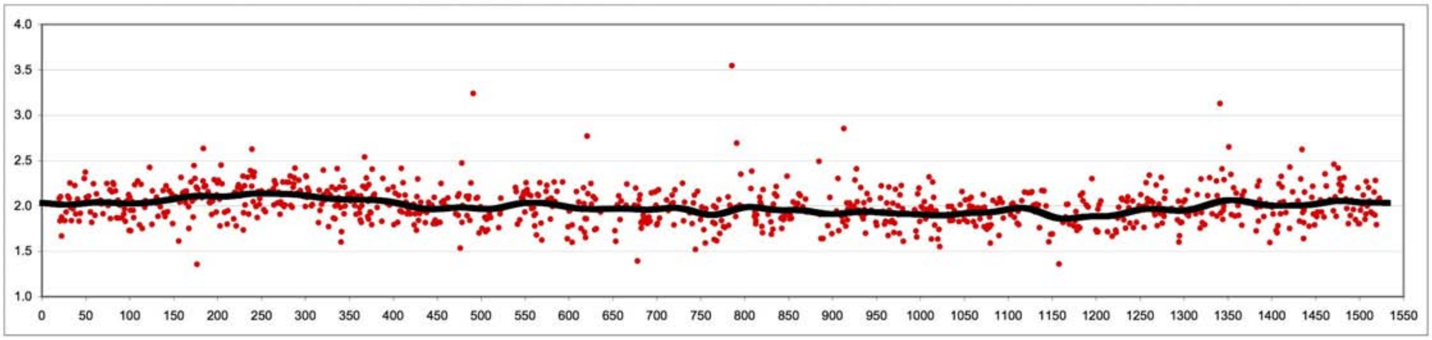

Figure 6B

YJL9999

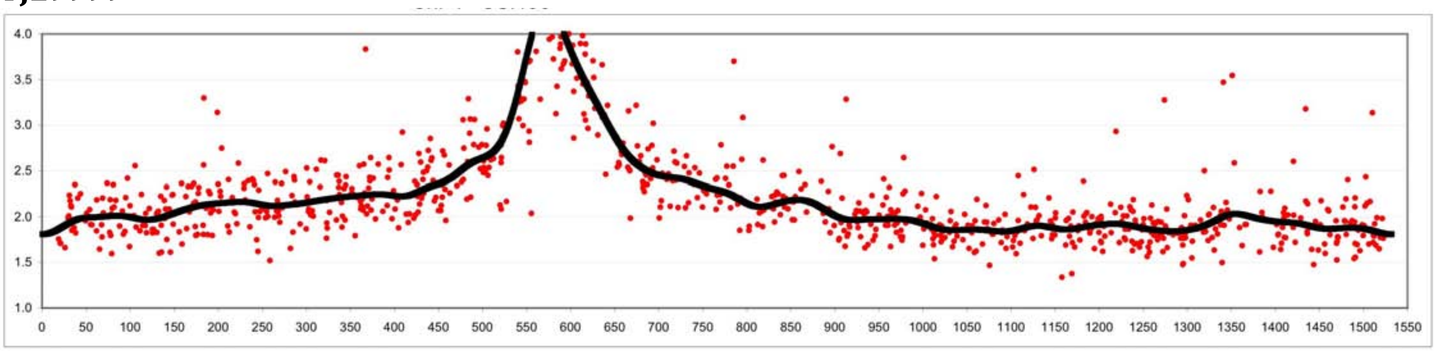

YJL10000

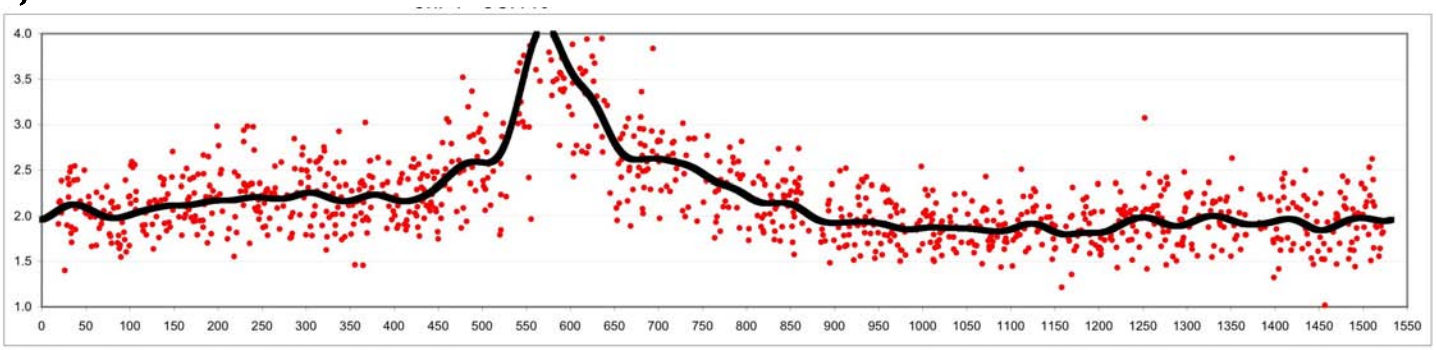

YJL10001

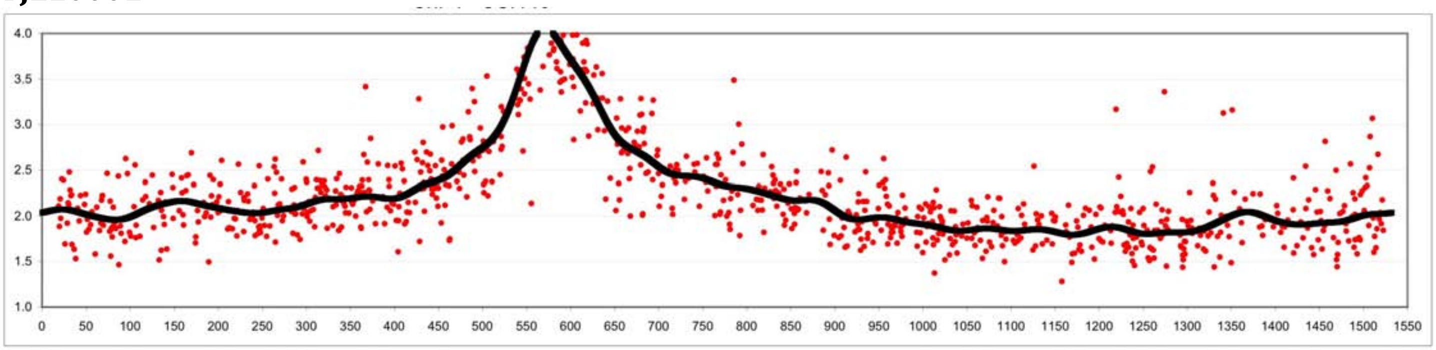

YJL10002

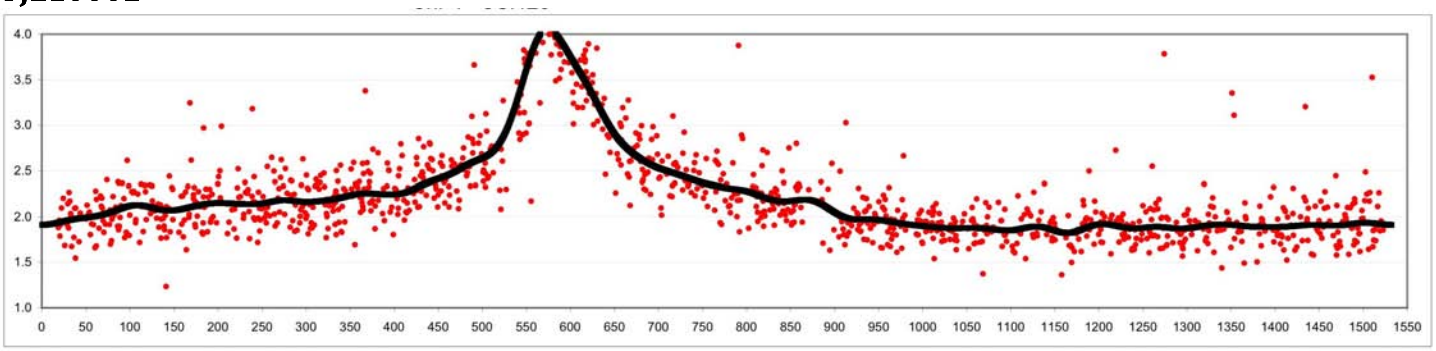

Figure 7

YJL9175

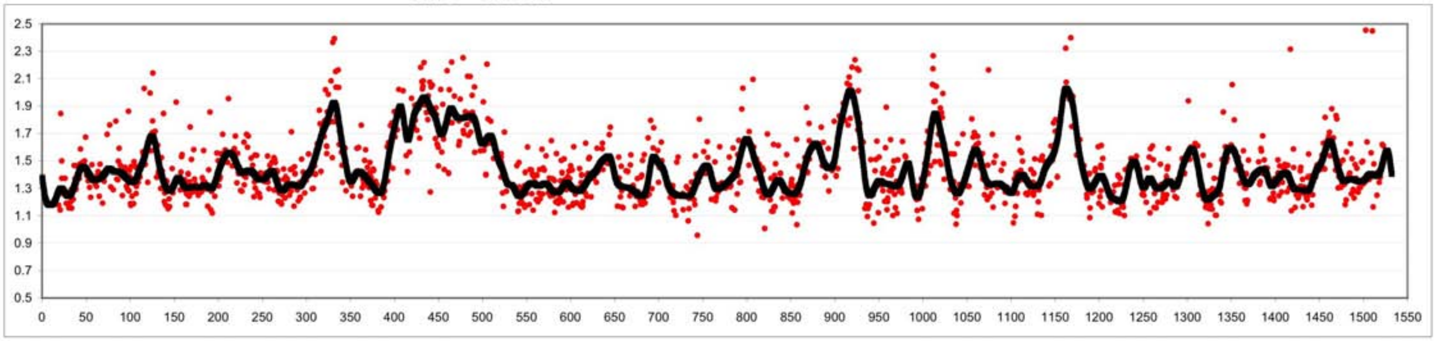

YJL9175

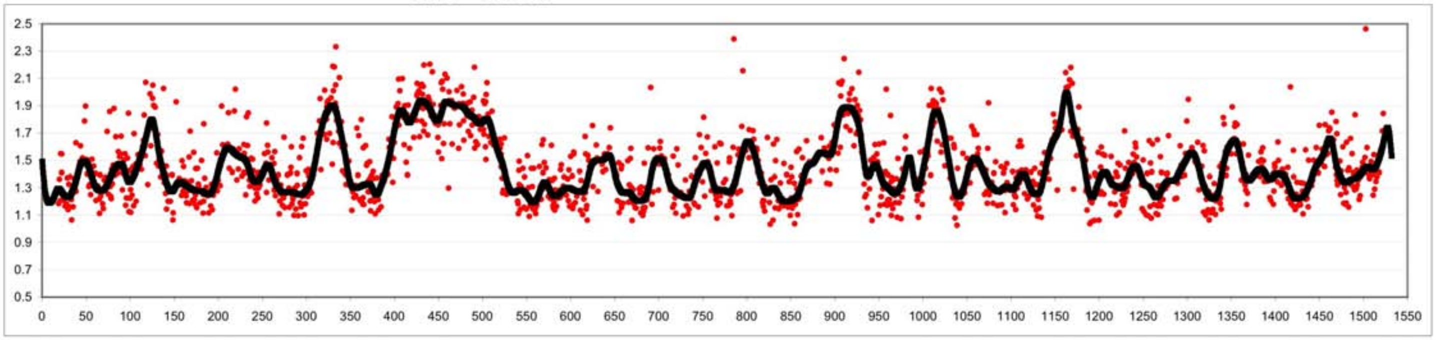

YJL9248

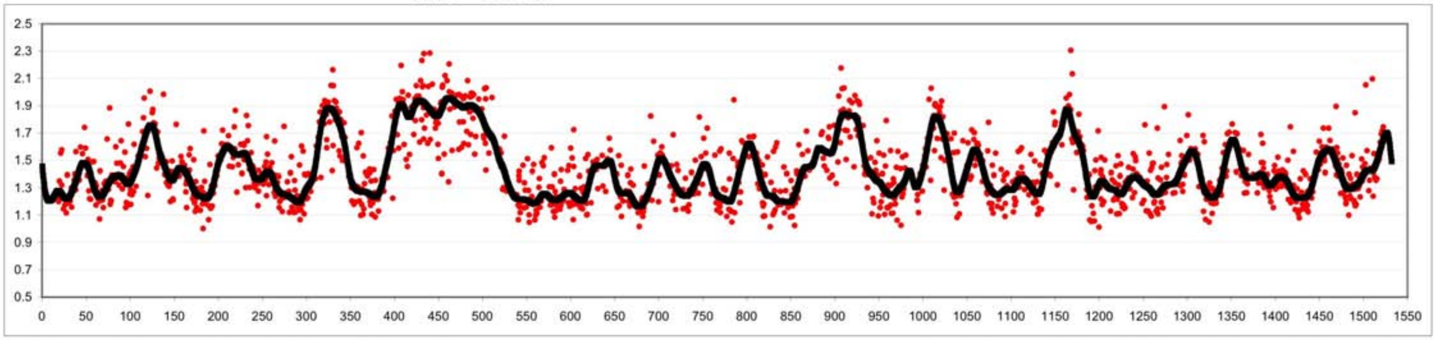

YJL9248

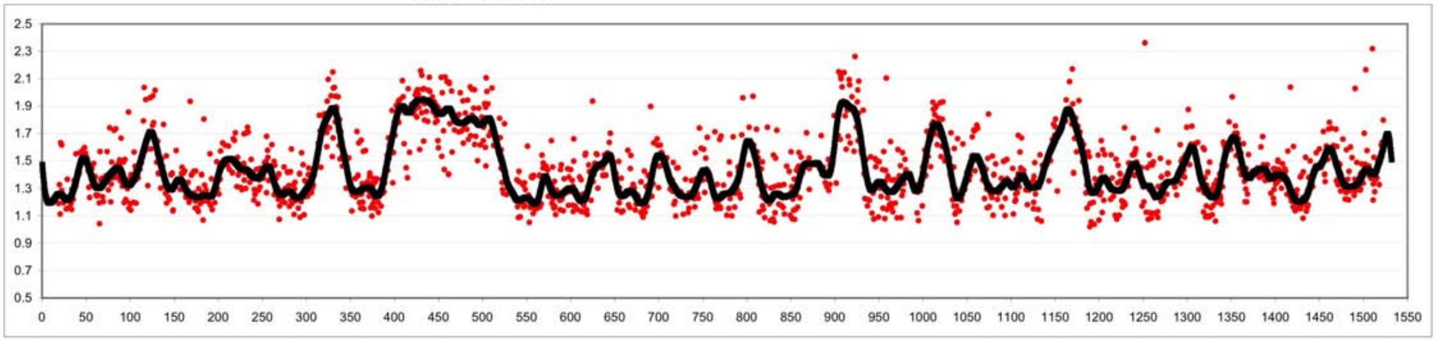

YJL9177

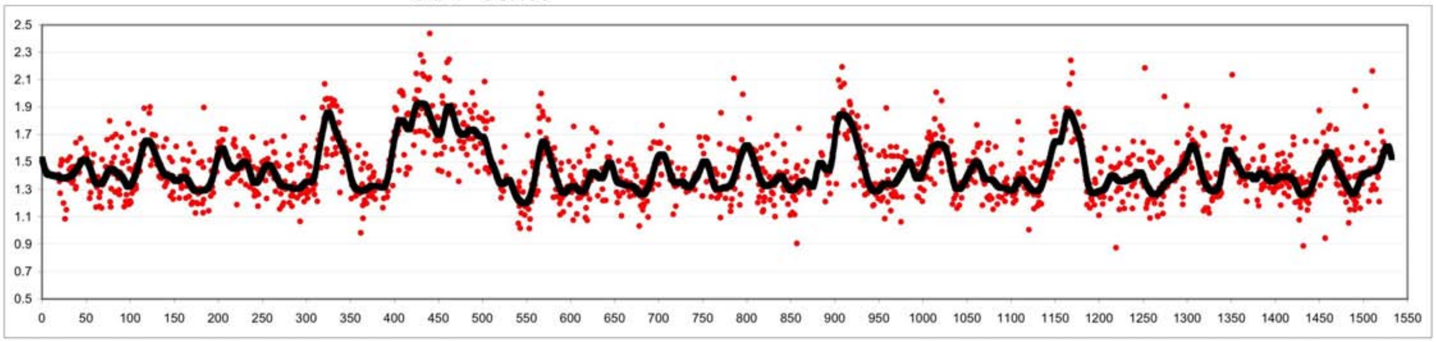

YJL9177

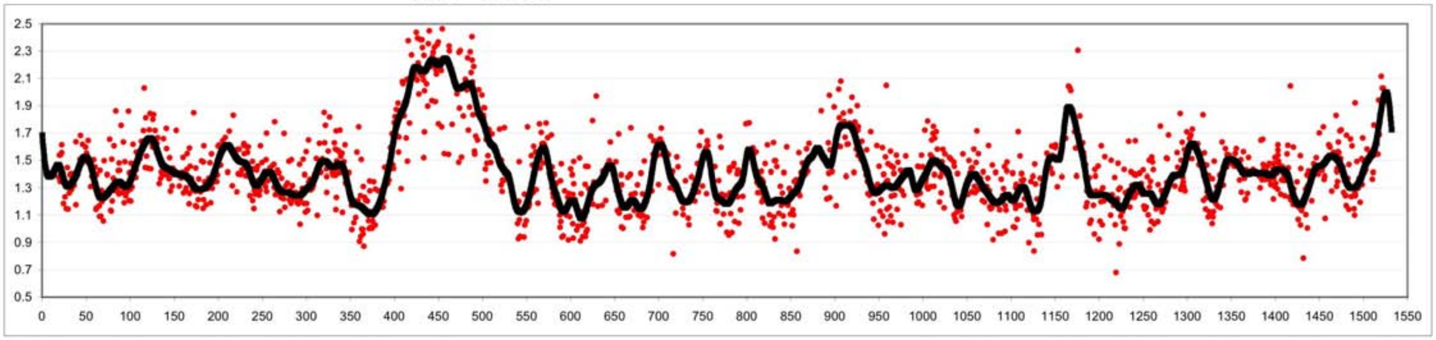

YJL9229

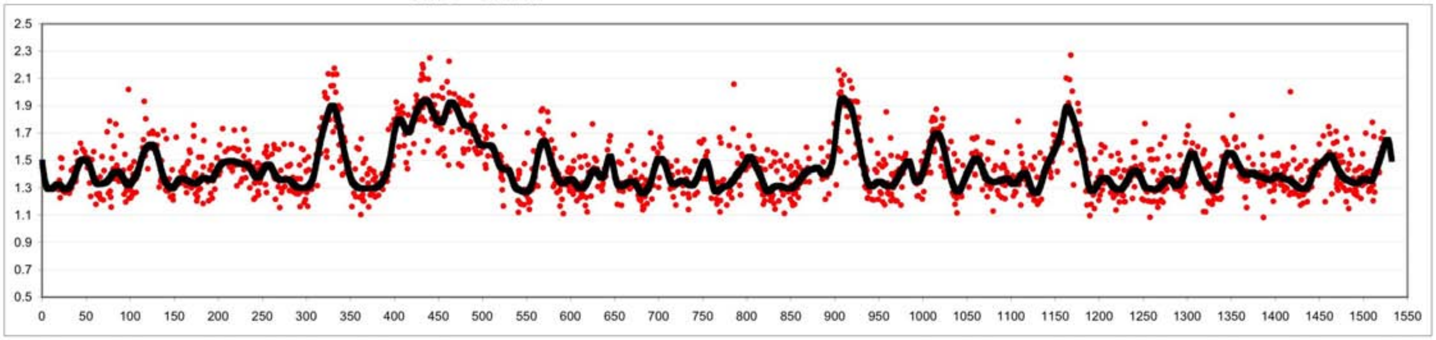

YJL9229

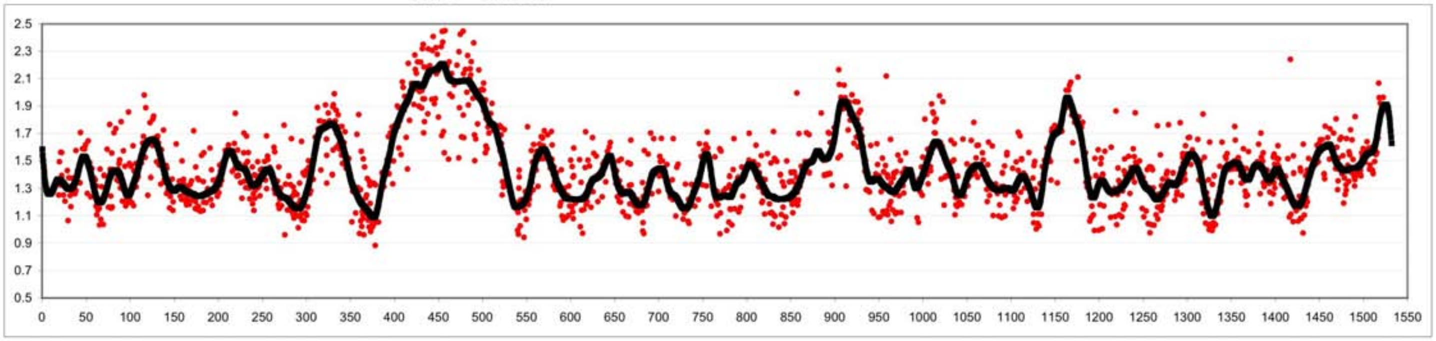

YJL9179

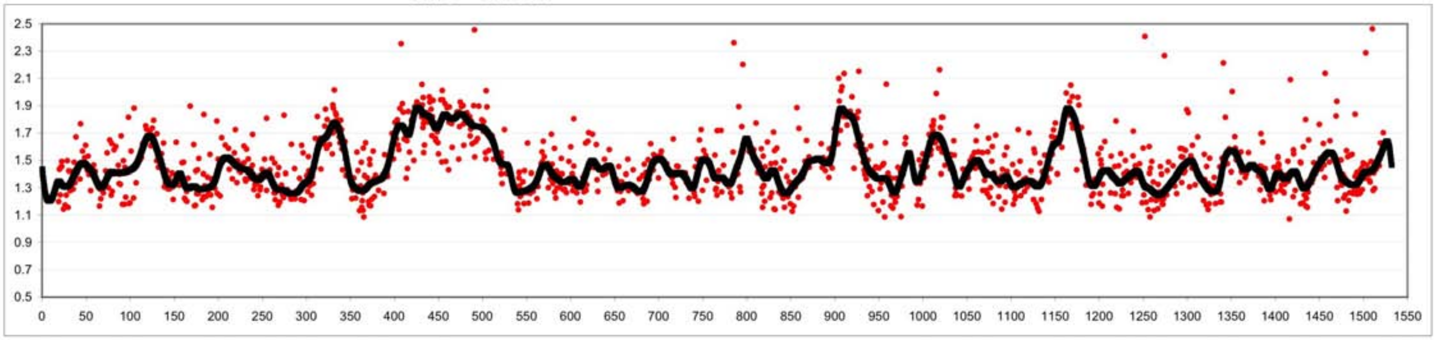

YJL9179

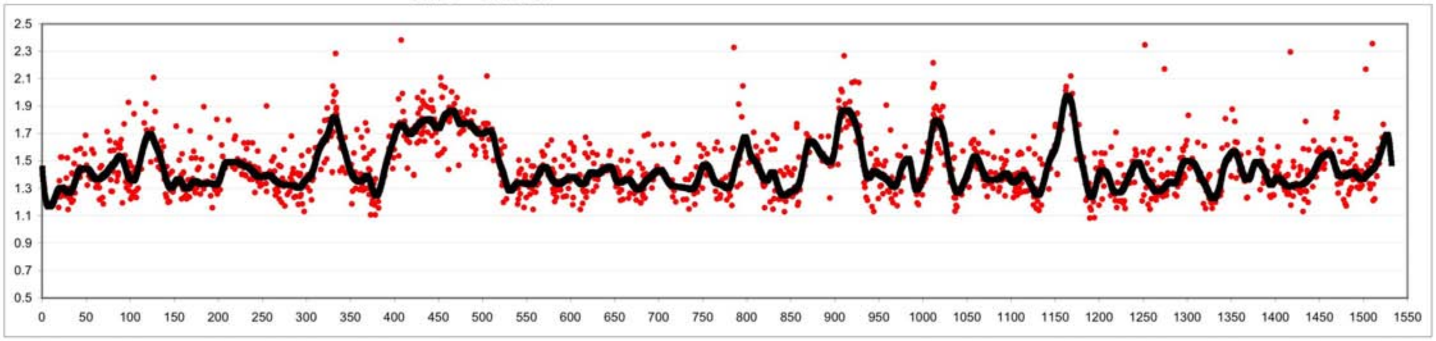

YJL9233

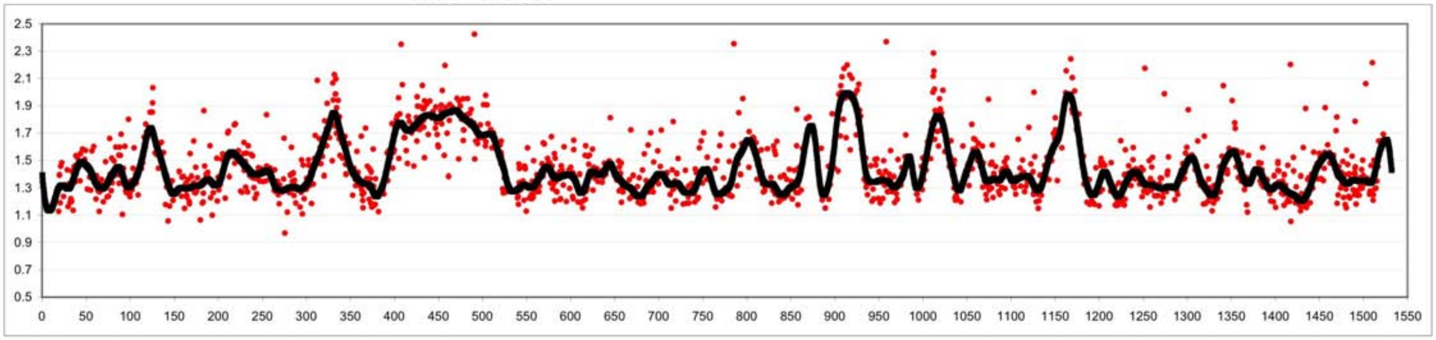

YJL9233

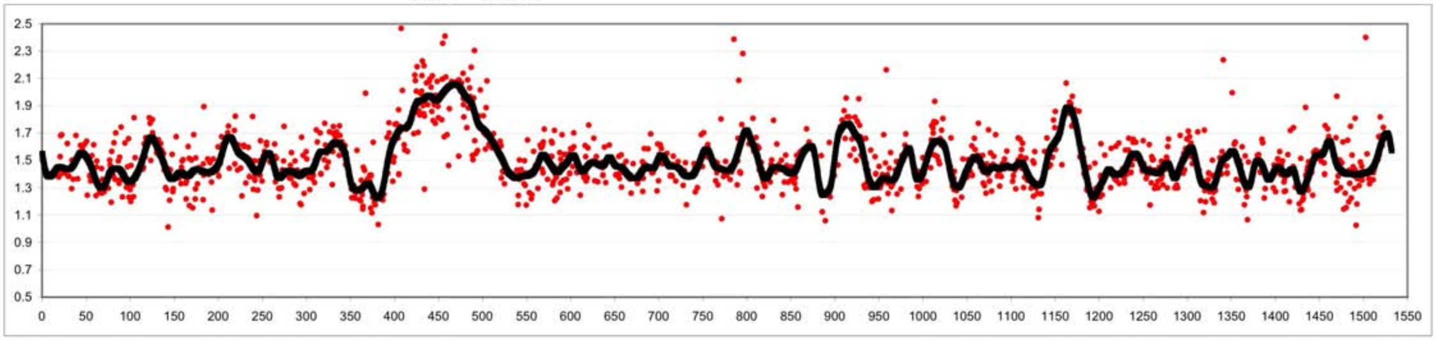

Supplemental Figure 1

YJL8923

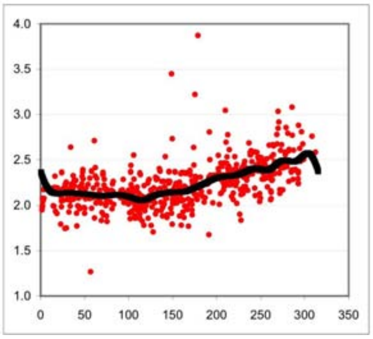

YJL8924

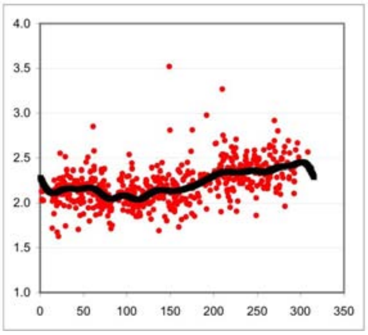

YJL3758

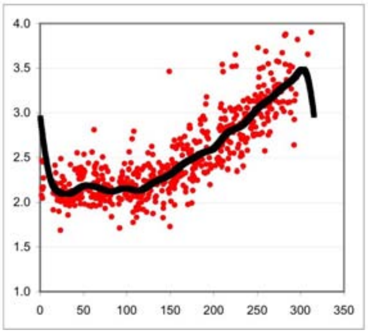

YJL3758

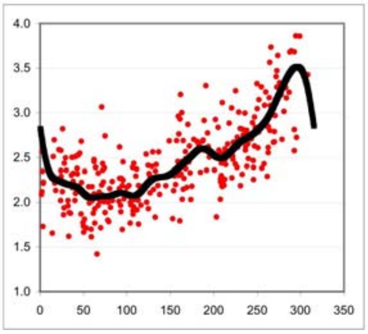

Supplemental Figure 2C
YJL8398 – Positive Control

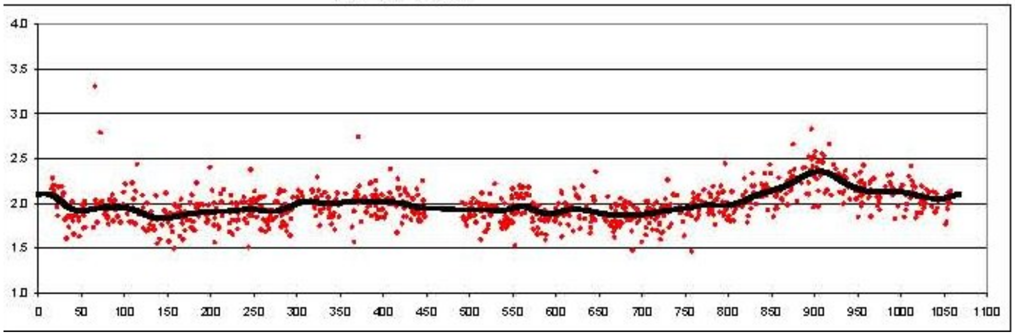

YJL8398 – Positive Control

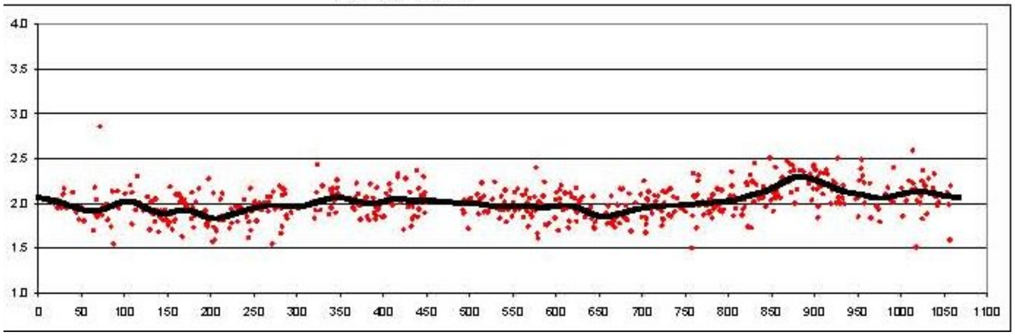

YJL8398 – Positive Control

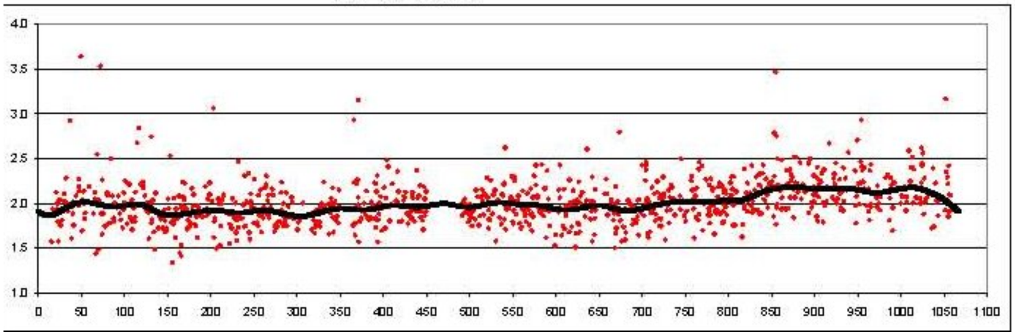

YJL8398 – Positive Control

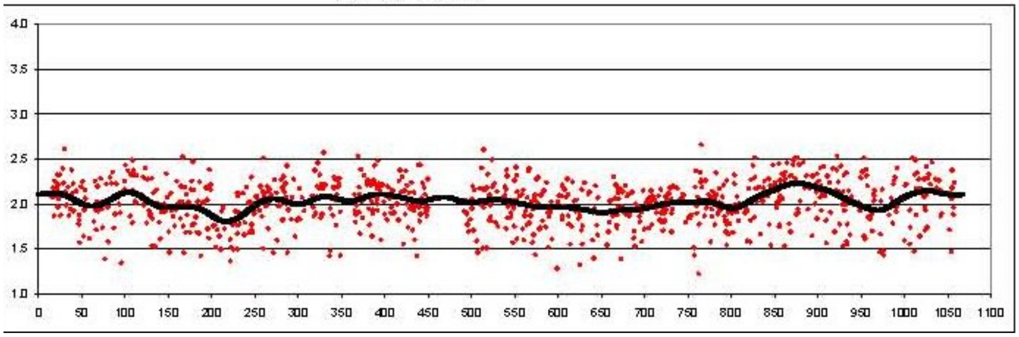

YJL8398 – Positive Control

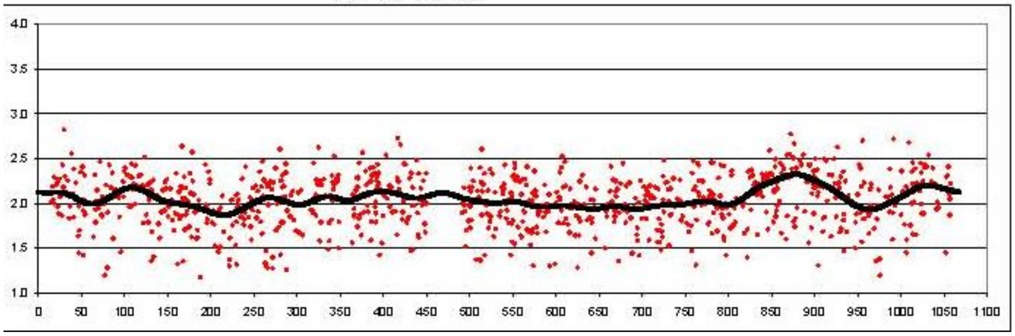

YJL8398 – Positive Control

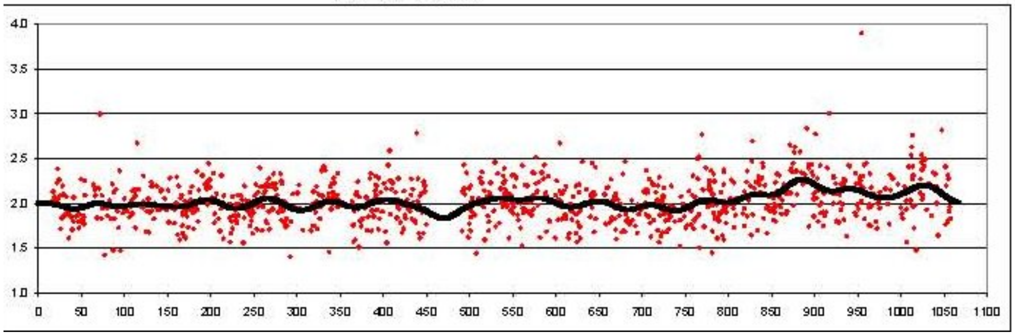

YJL8398 – Positive Control

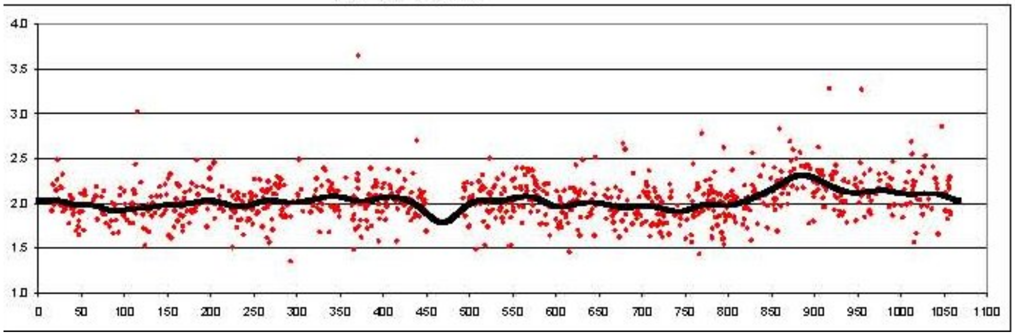

YJL8398 – Positive Control

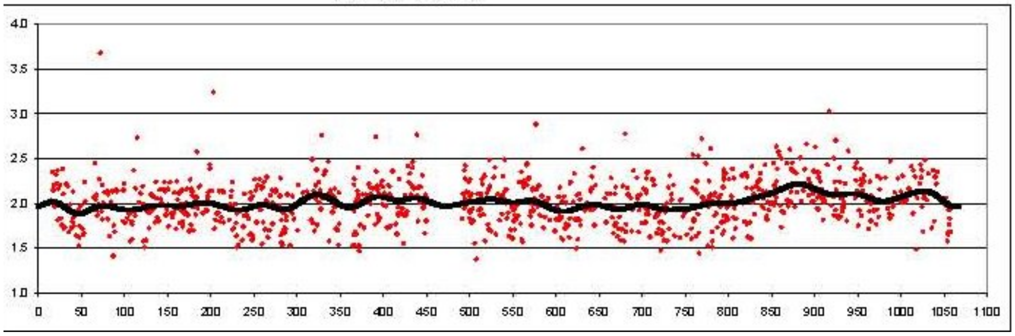

YJL8398 – Positive Control

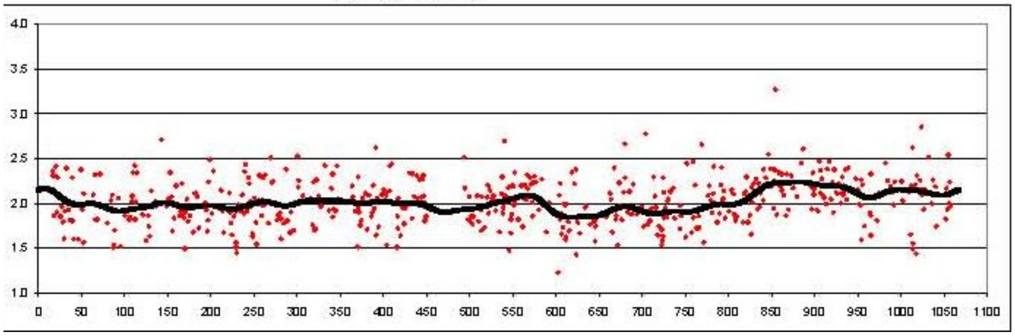

YJL8398 – Positive Control

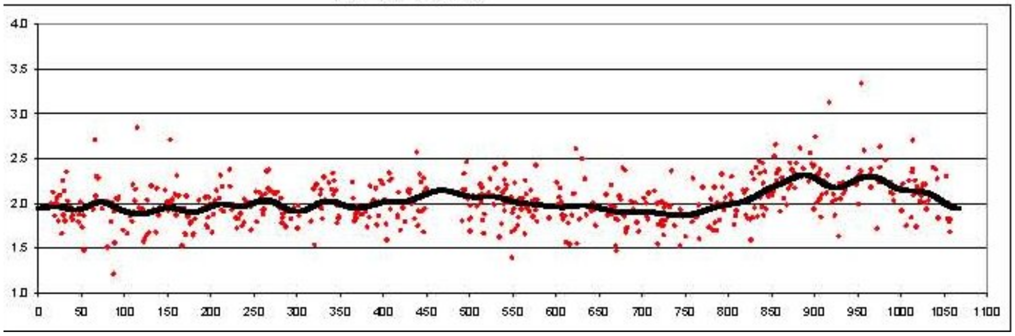

YJL9152 - Negative Control

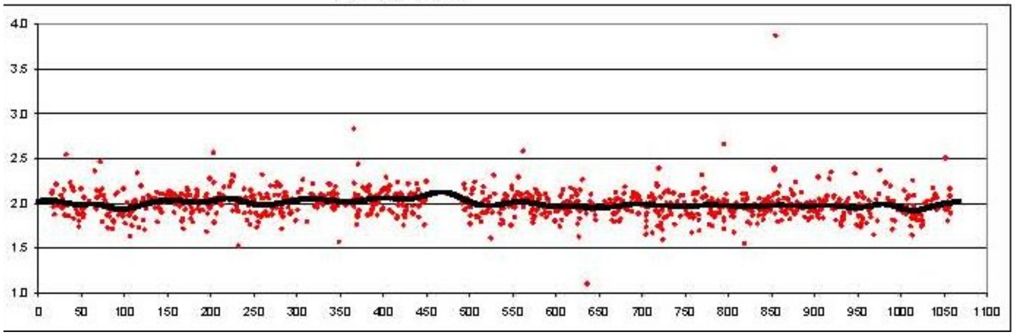

YJL9152 - Negative Control

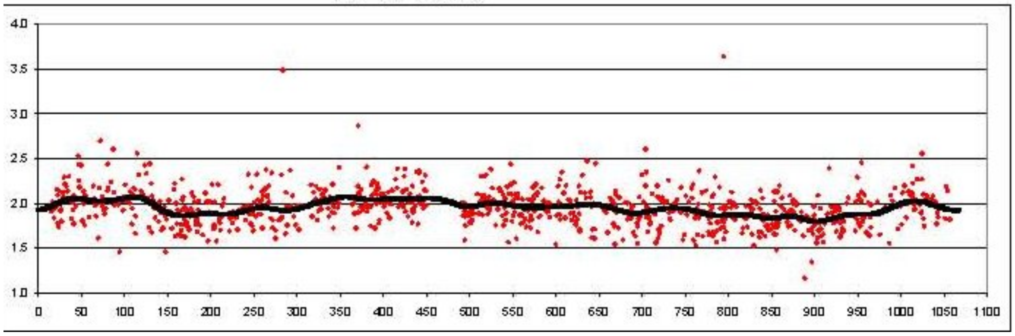

YJL9152 - Negative Control

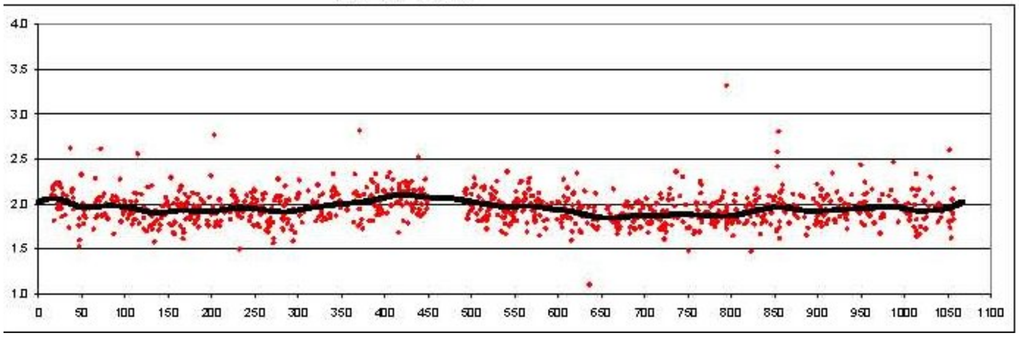

YJL9152 - Negative Control

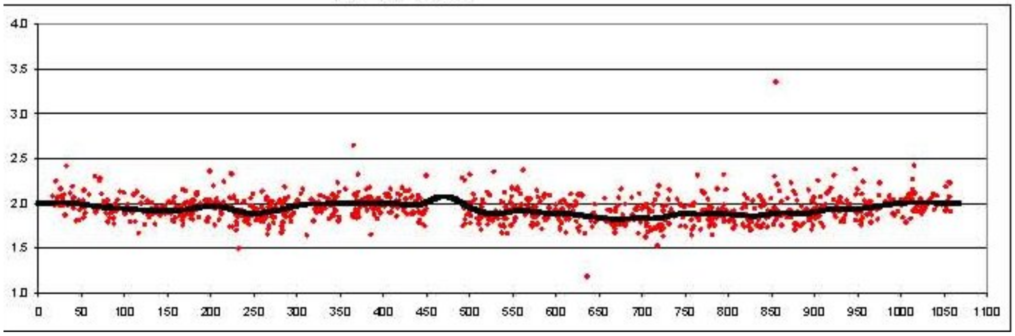

YJL9152 - Negative Control

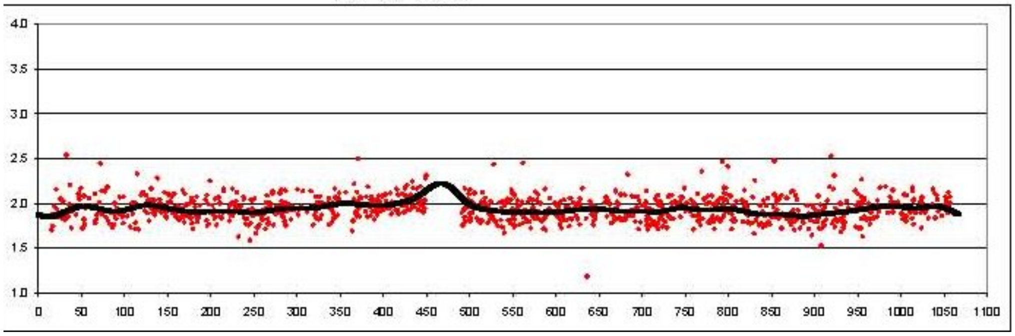

YJL9152 - Negative Control

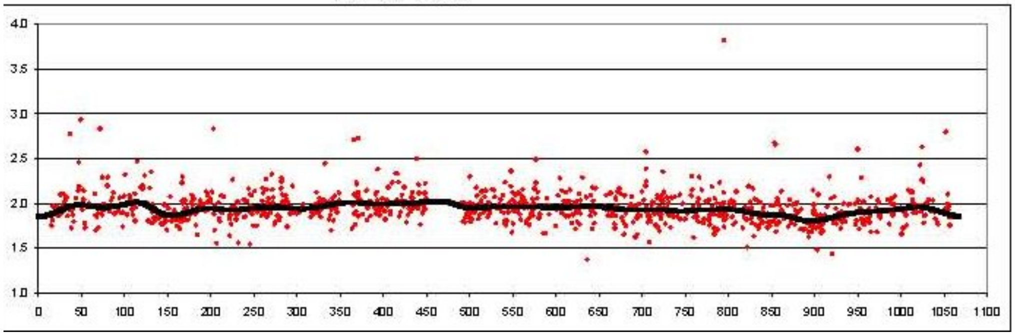

YJL9152 - Negative Control

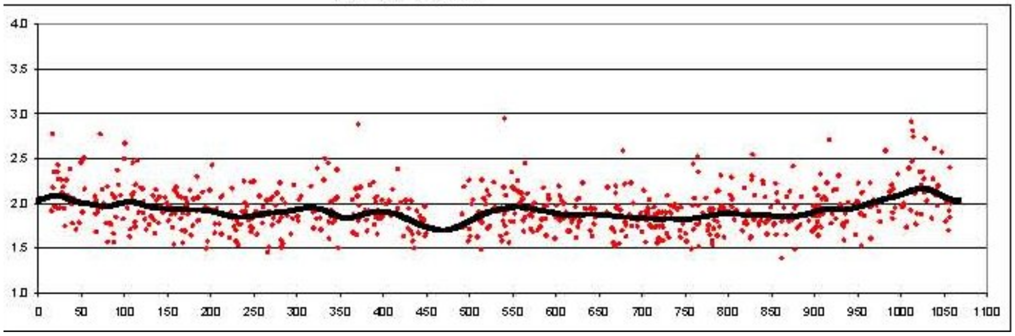

YJL6893

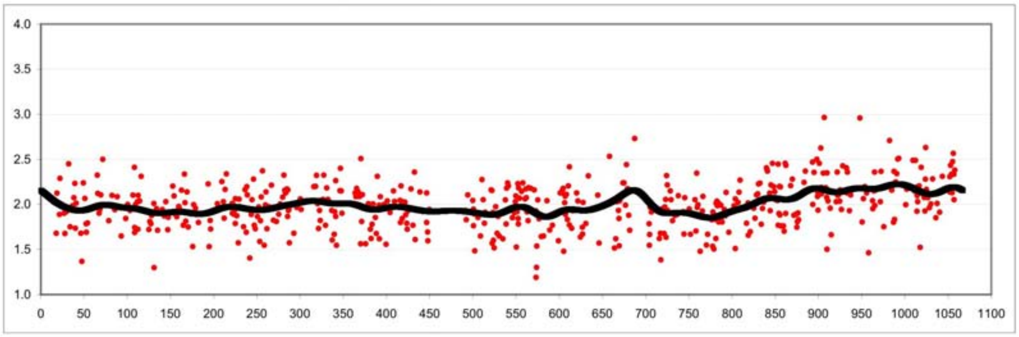

YJL6894

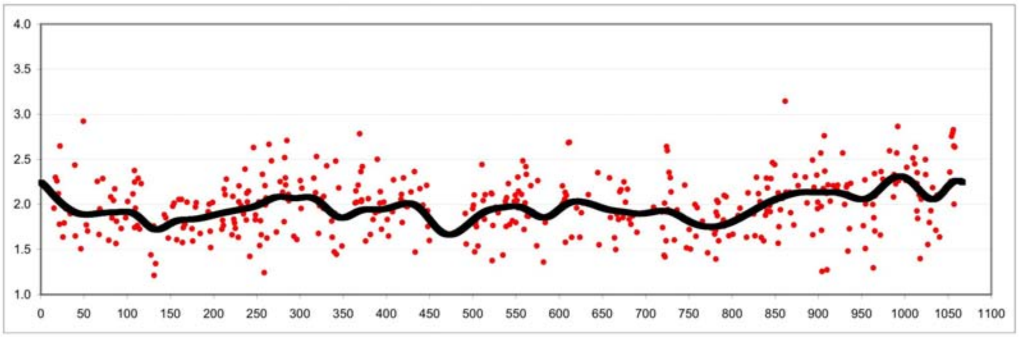

YJL6896

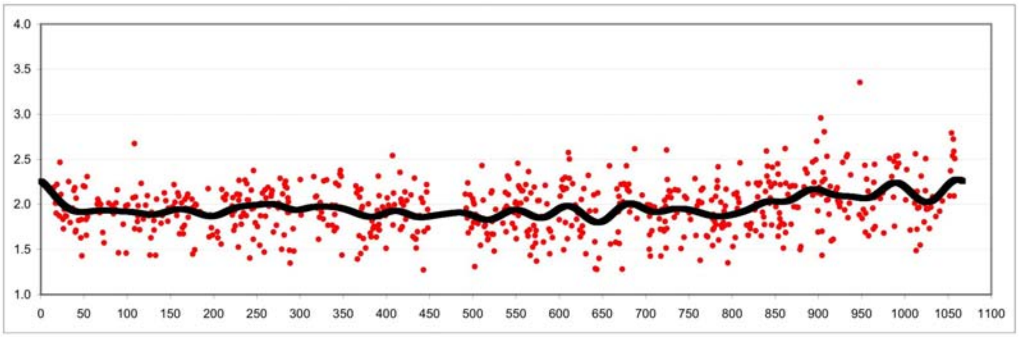

YJL6897

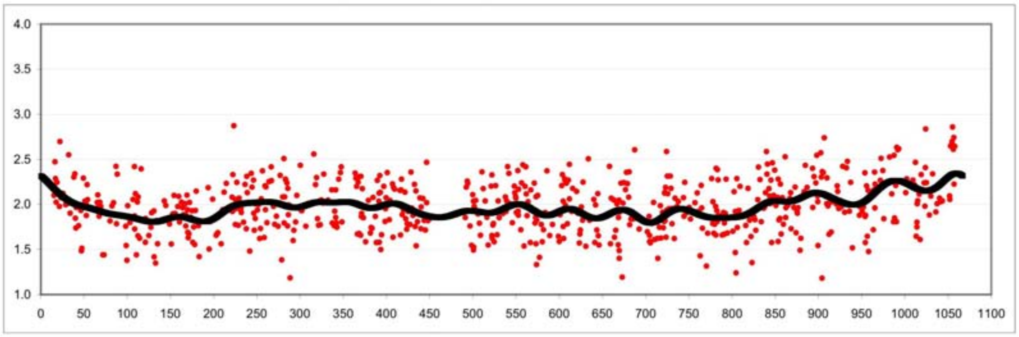

YJL6899

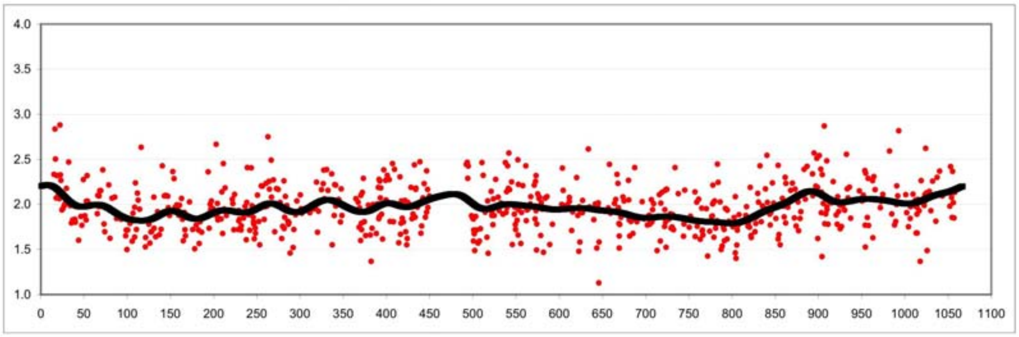

YJL6900

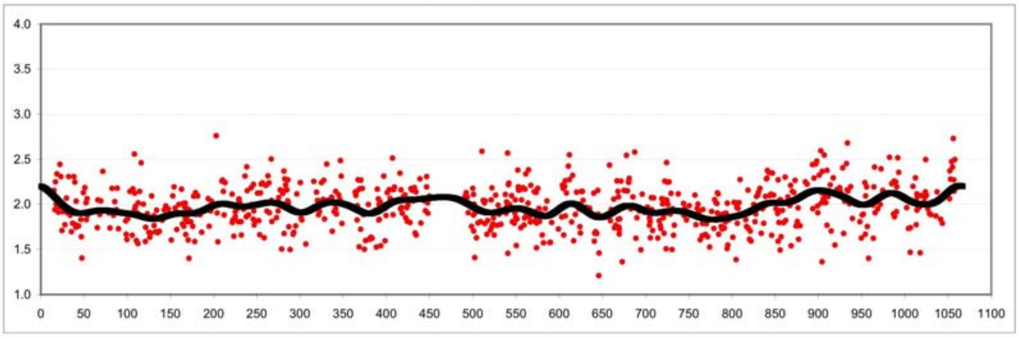

YJL6902

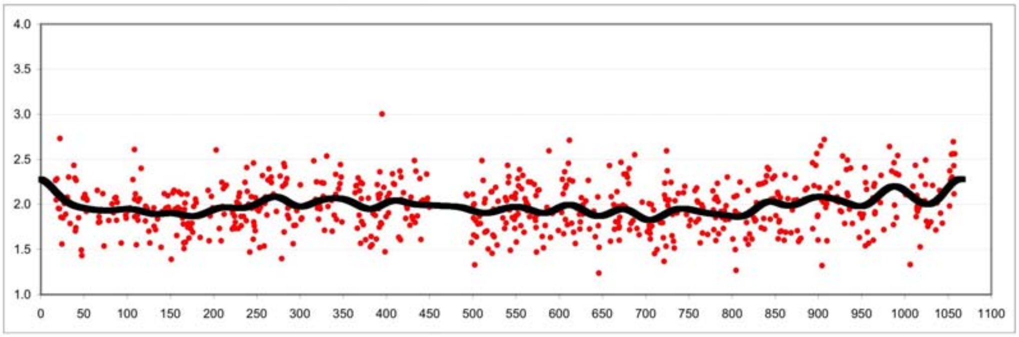

YJL6903

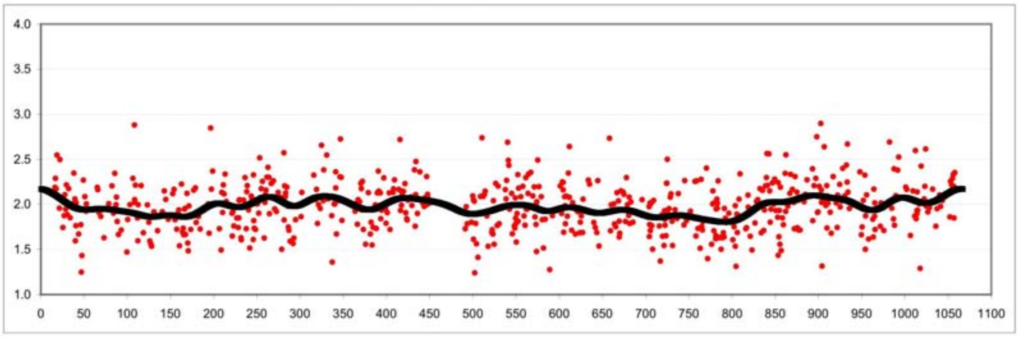

Supplemental Figure 2D

YJL8701

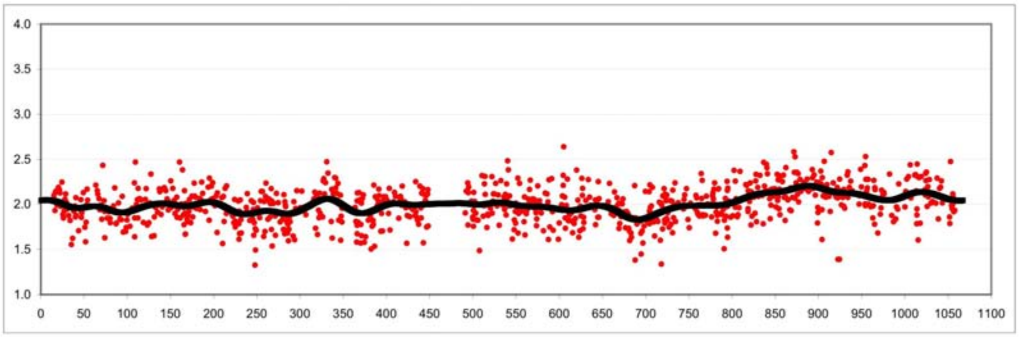

YJL8702

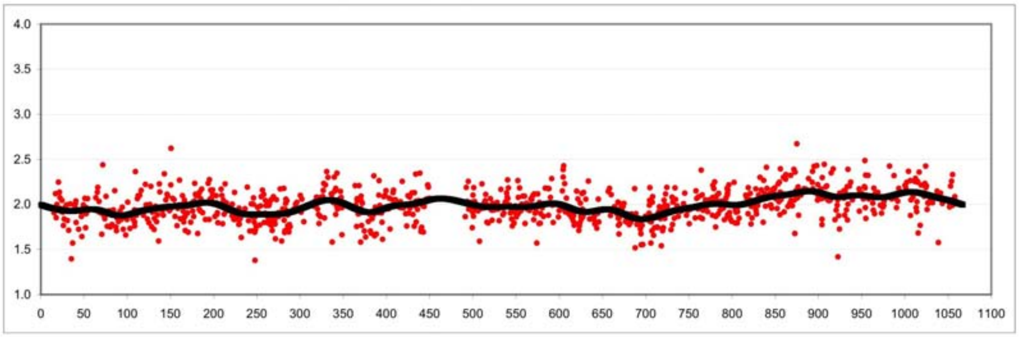

YJL8745

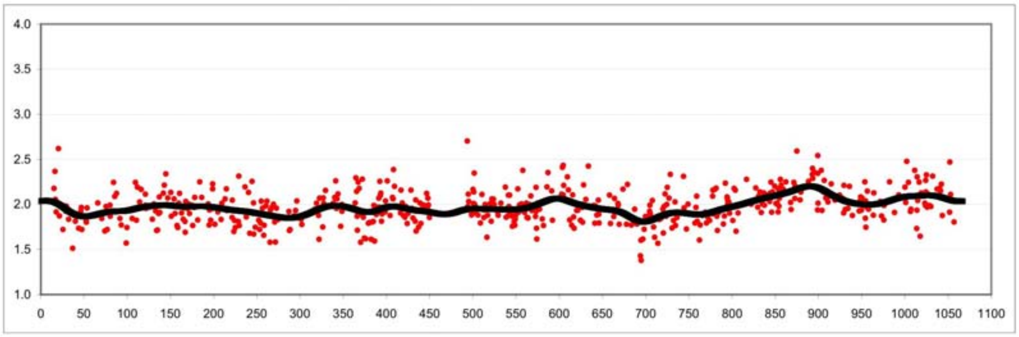

YJL8746

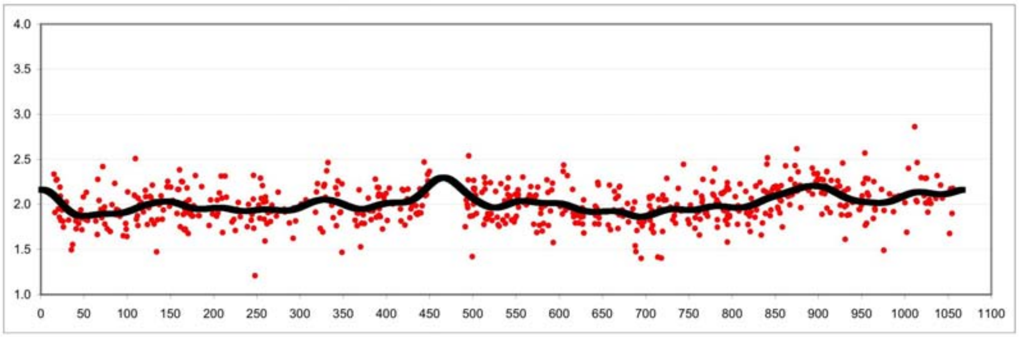

YJL8749

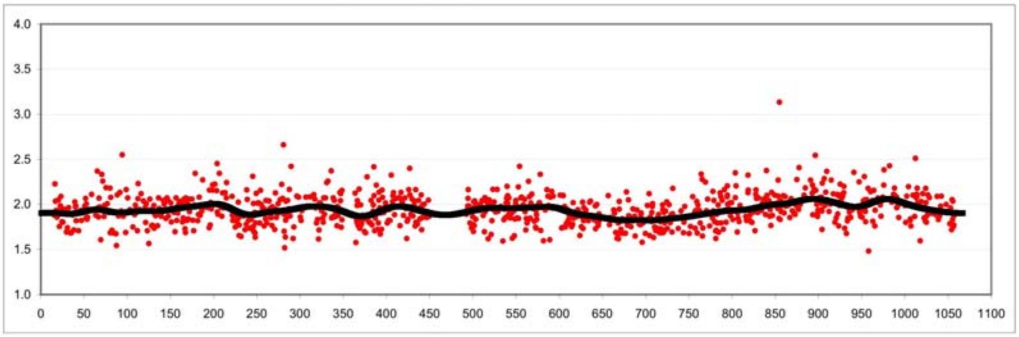

YJL8750

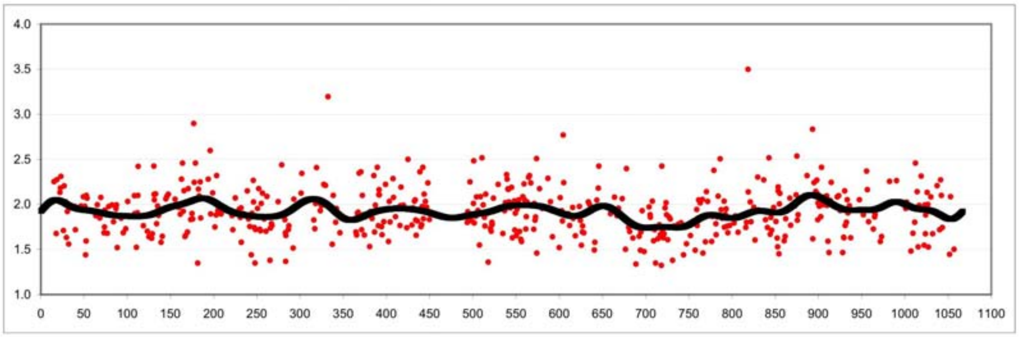

Supplemental Figure 3

YJL8526

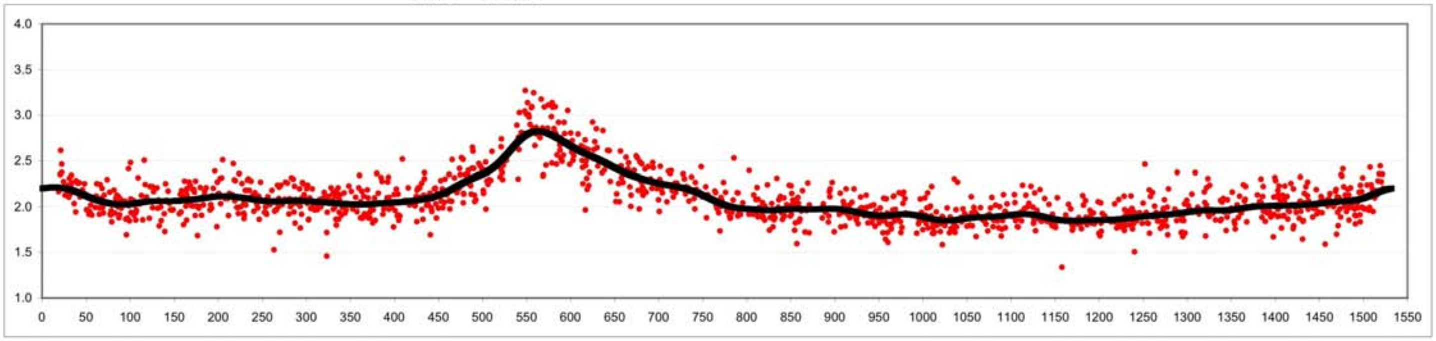

YJL8526

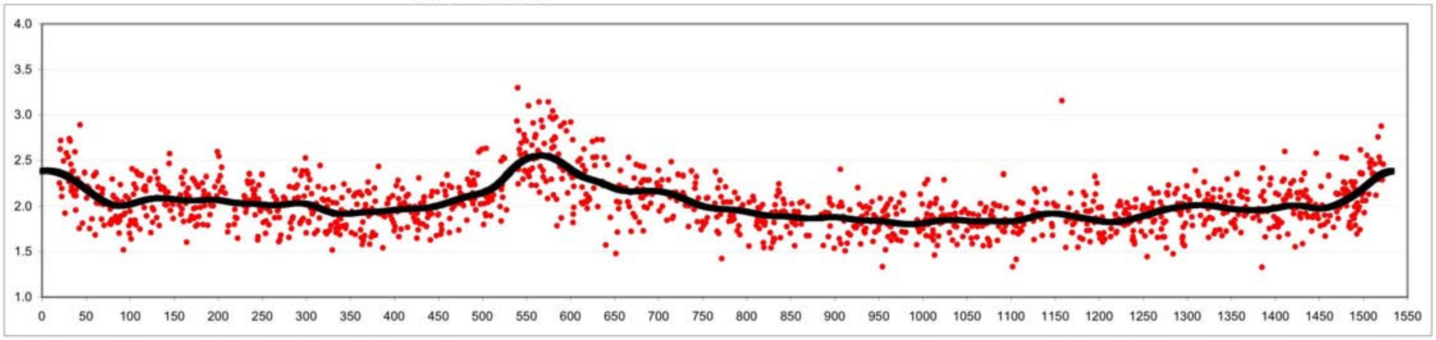

YJL8538

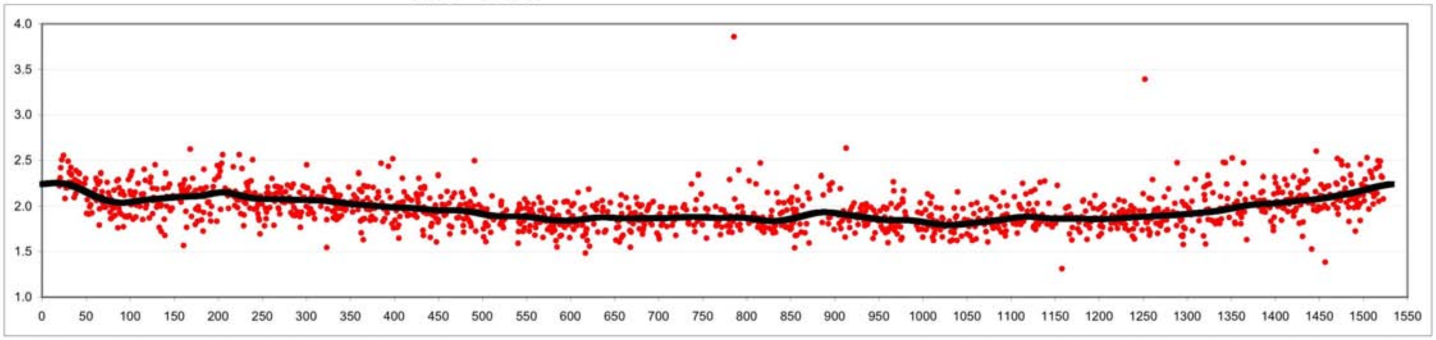

YJL8538

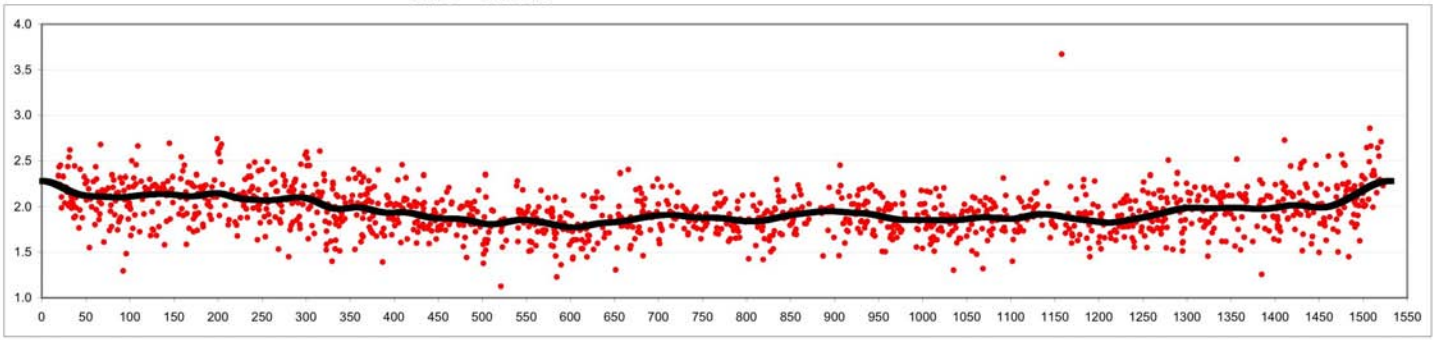

Supplemental Figure 4A
YJL8398 – control

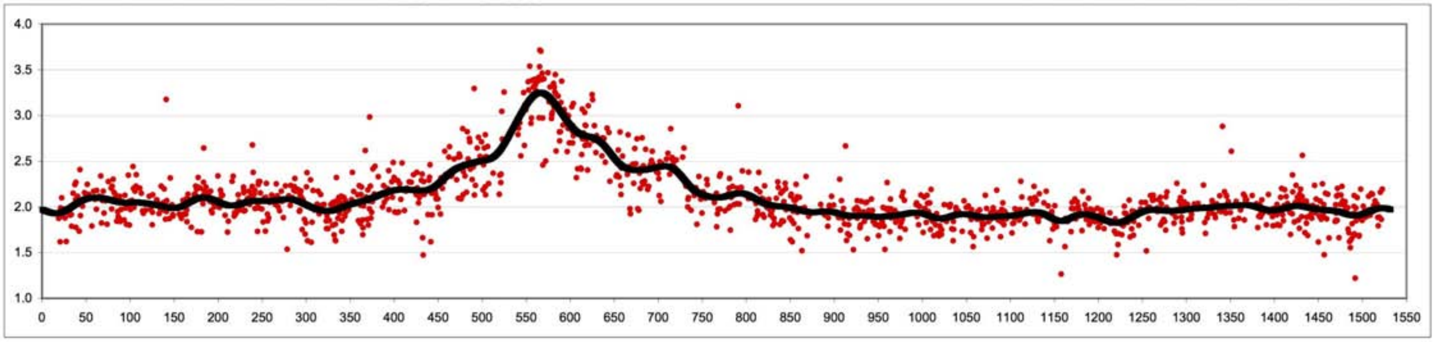

YJL8398 – control

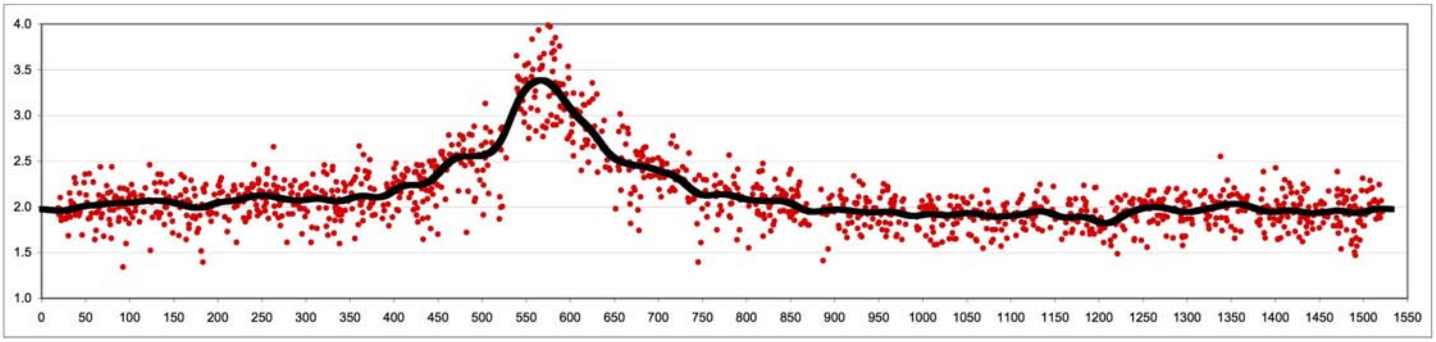

YJL8771 – Linker L9+L11+L15

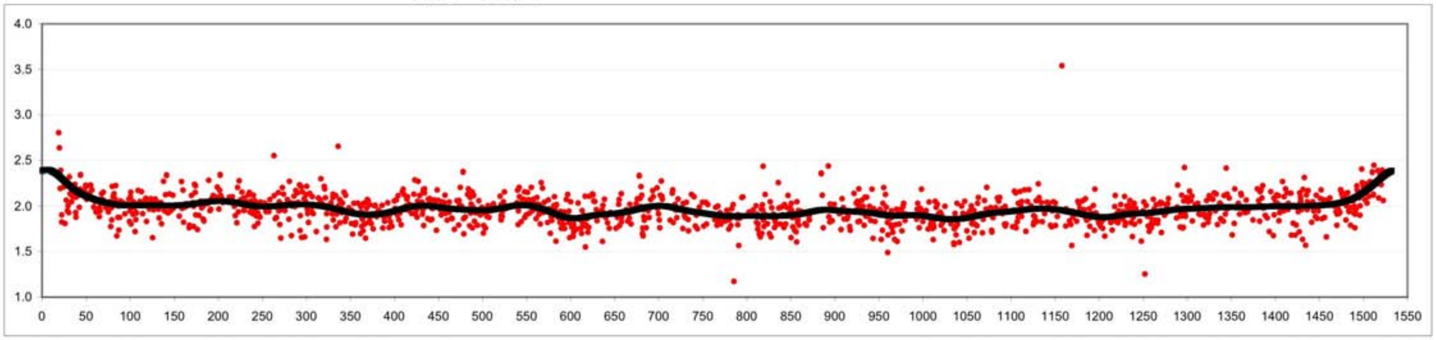

YJL8771 – Linker L9+L11+L15

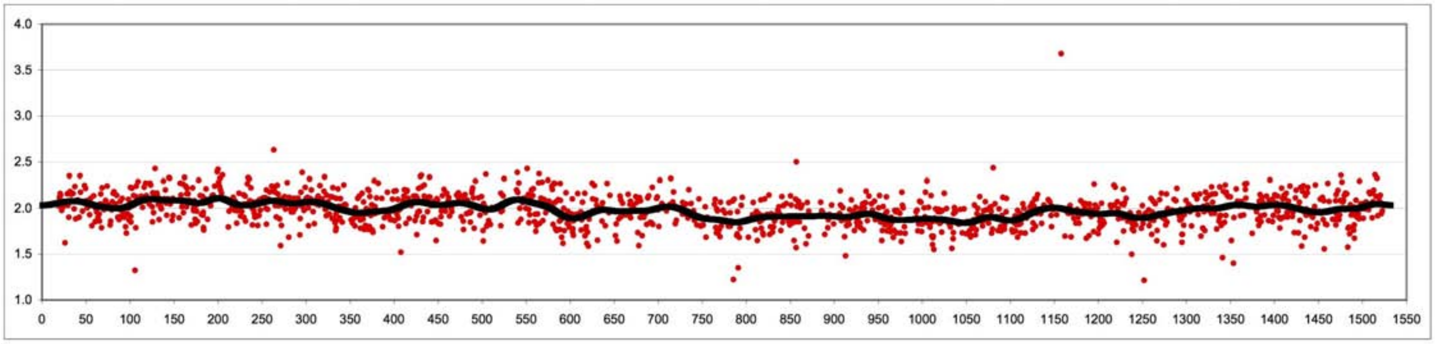

YJL8773 – Linker L9+L15

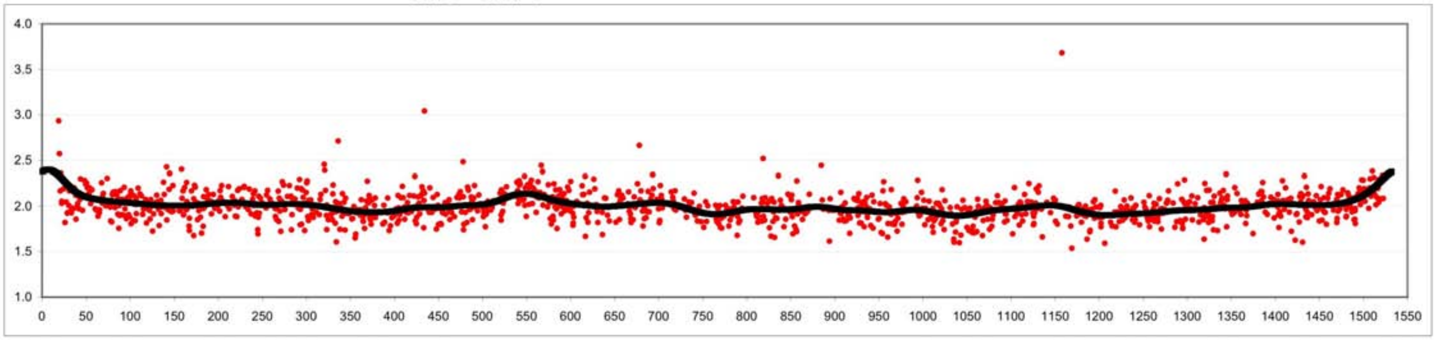

YJL8773 – Linker L9+L15

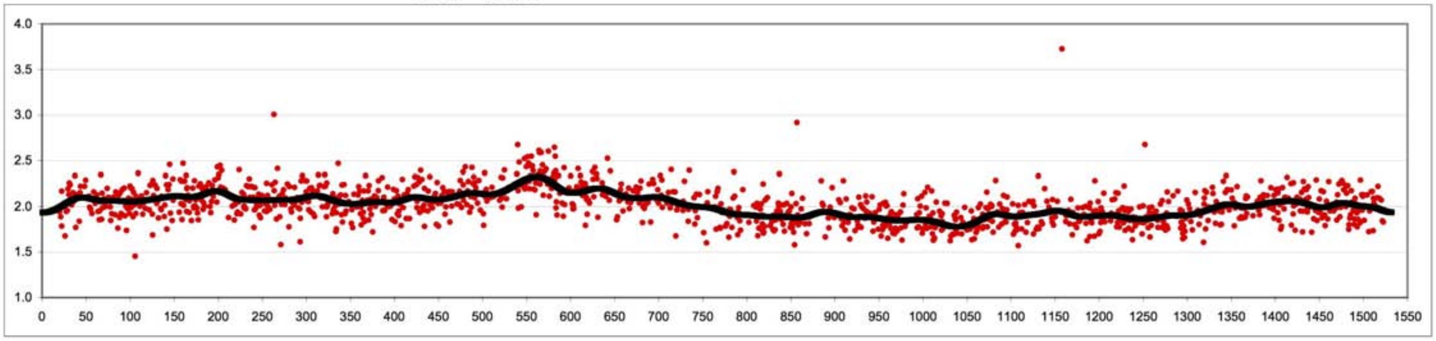

YJL8775 – Linker L11+L15

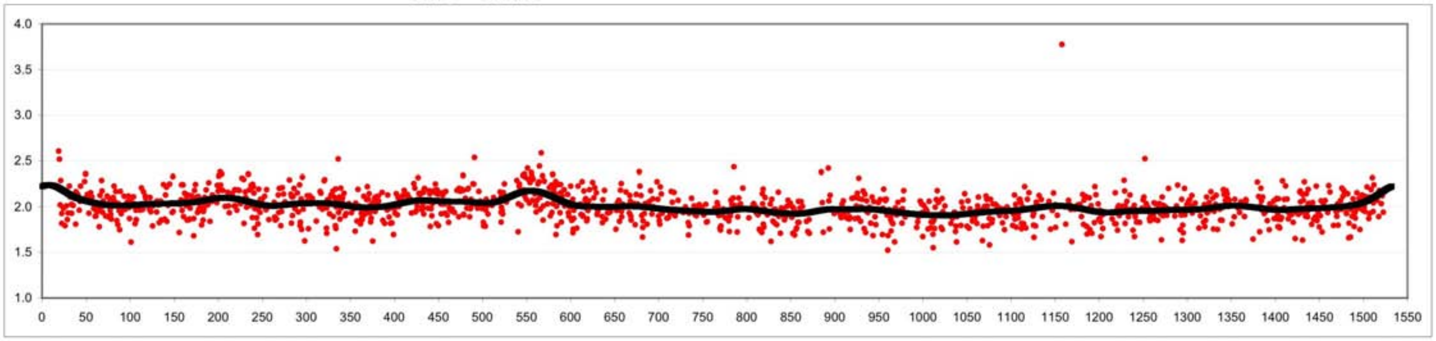

YJL8775 – Linker L11+L15

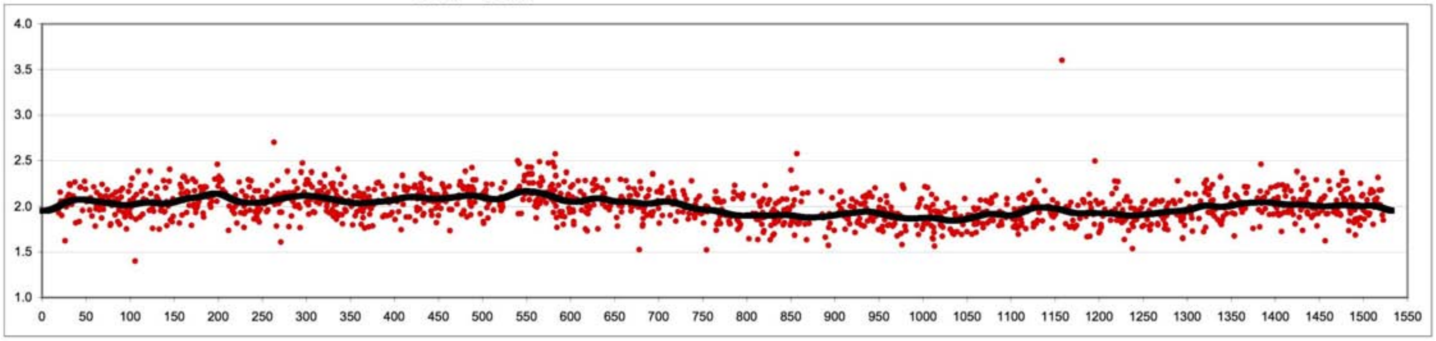

YJL8777 – Linker L9+L11

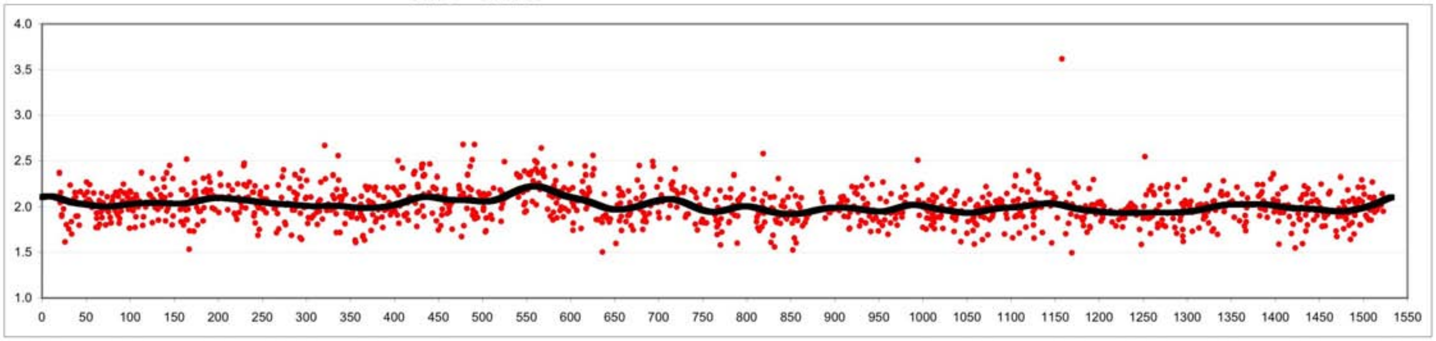

YJL8777 – Linker L9+L11

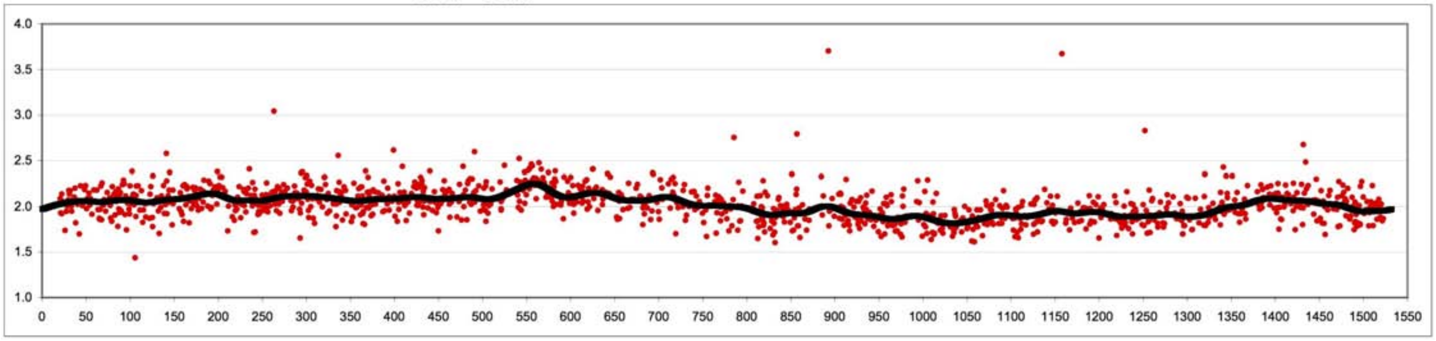

YJL8826 – Linker L13+L17

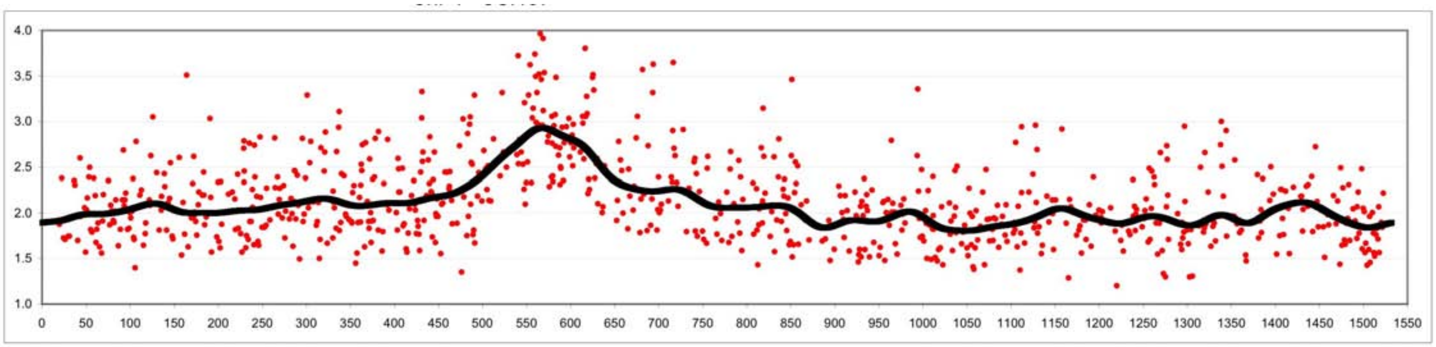

YJL8826 – Linker L13+L17

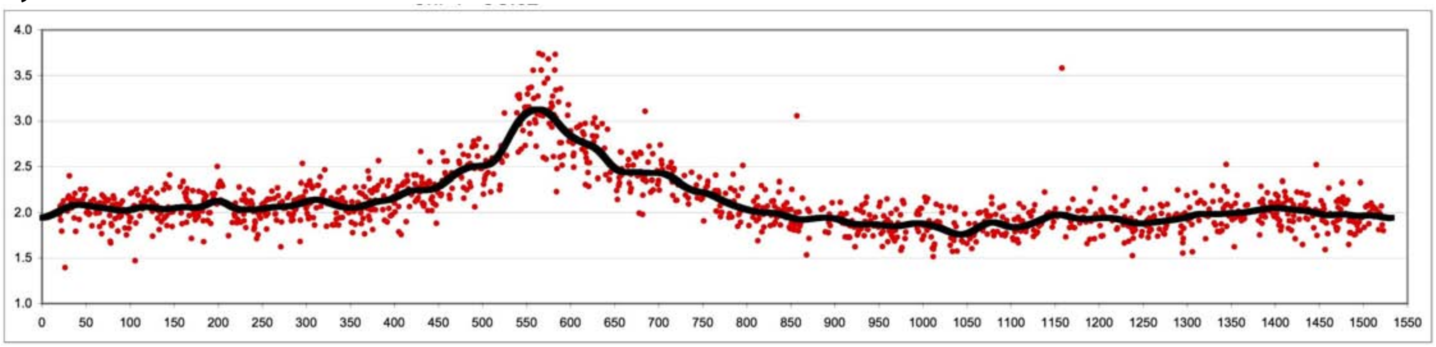

YJL8828 – Linker L21+L23

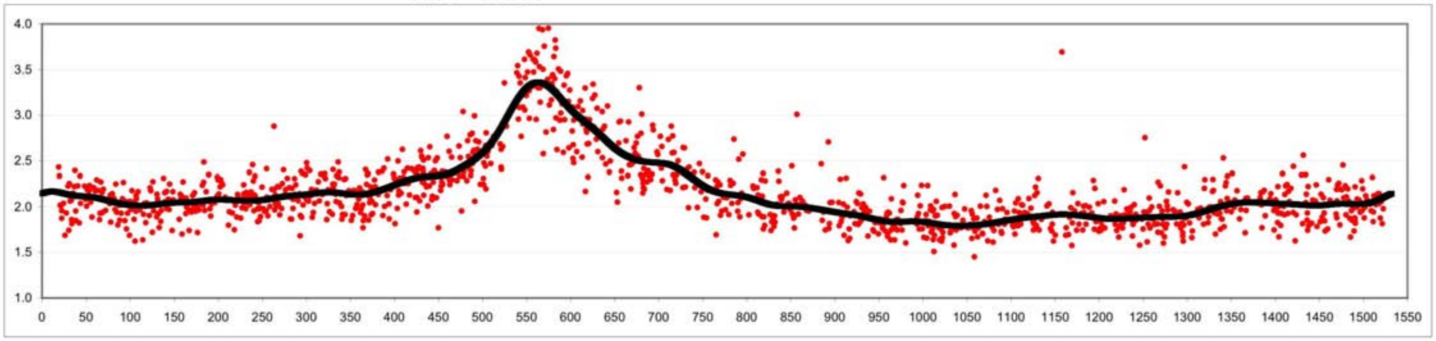

YJL8828 – Linker L21+L23

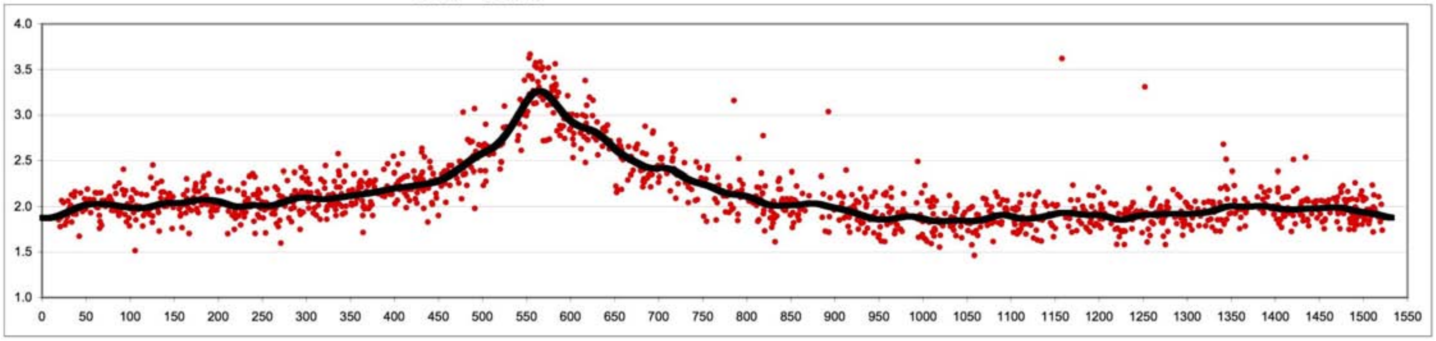

YJL8830 – Linker L9+L17

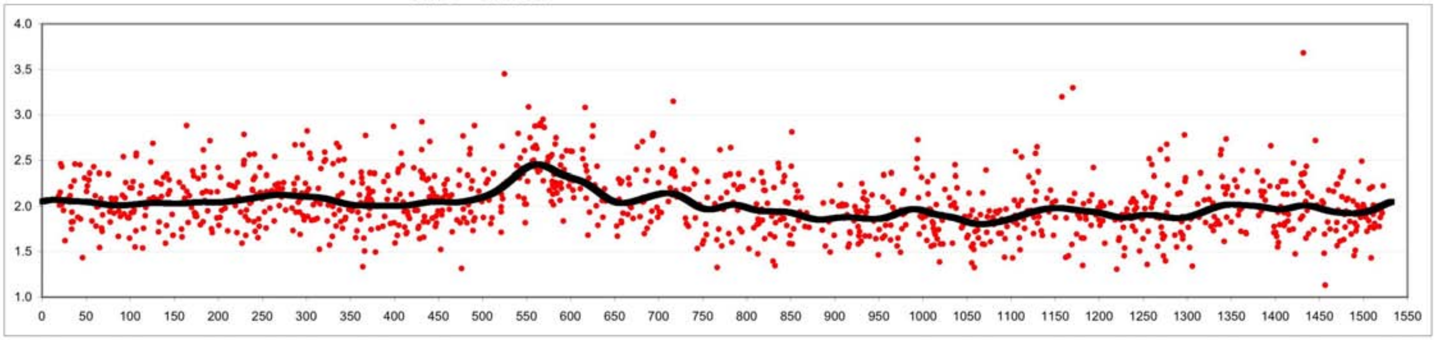

YJL8830 – Linker L9+L17

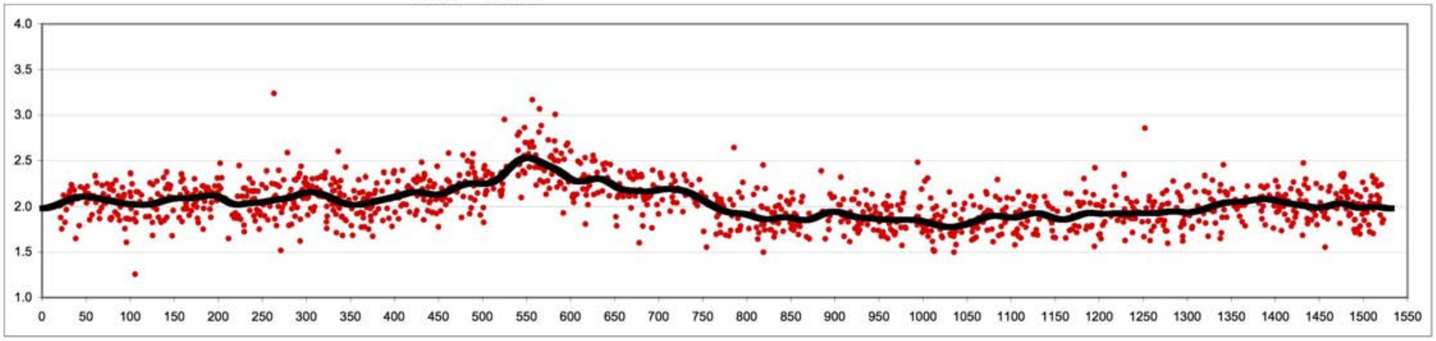

YJL8832 – Linker L11+L17

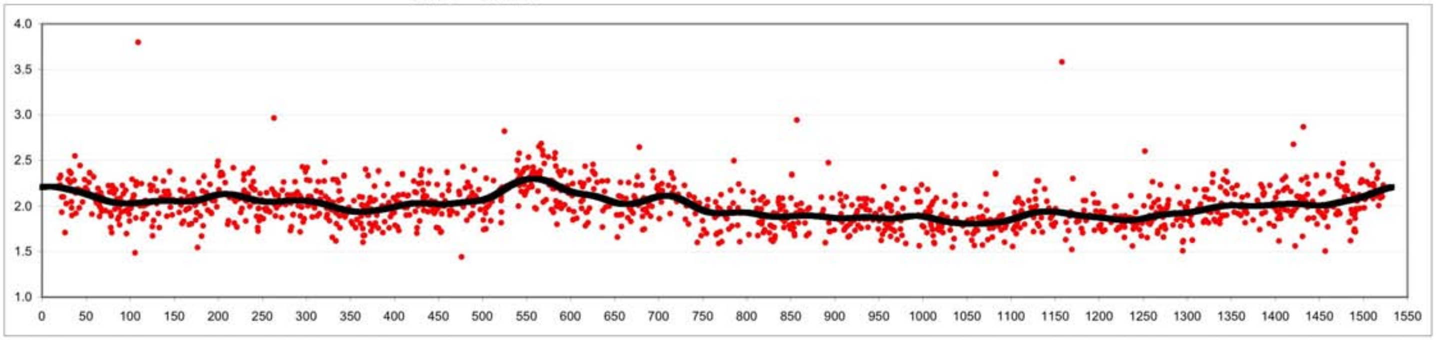

YJL8832 – Linker L11+L17

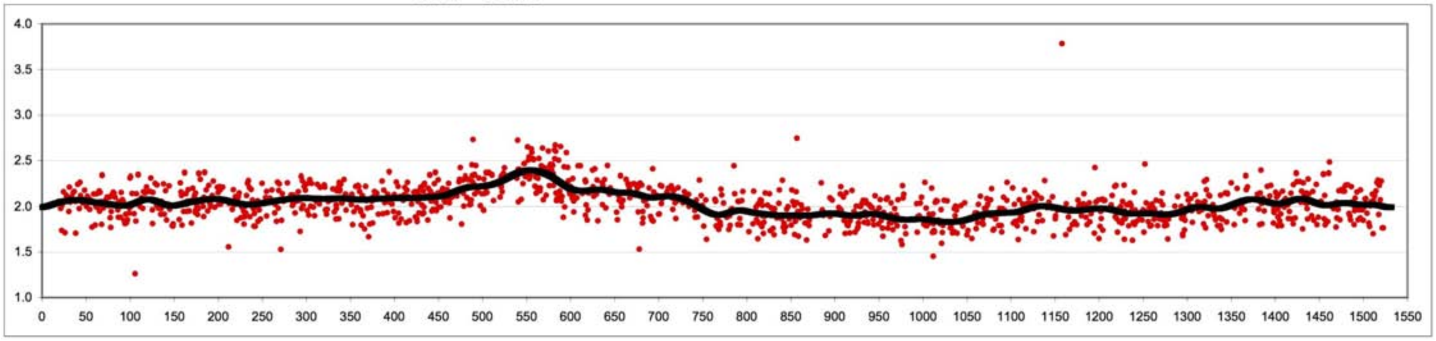

YJL8834 – Linker L15+L17

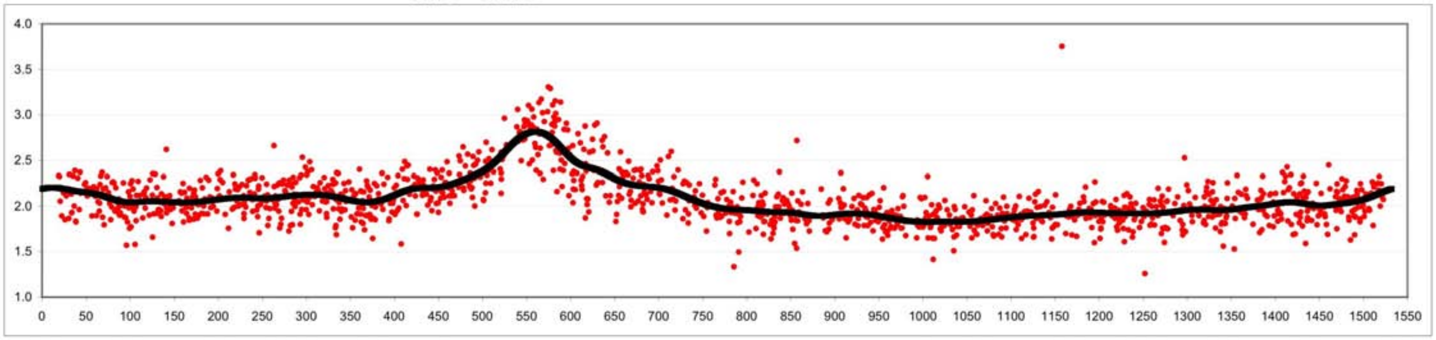

YJL8834 – Linker L15+L17

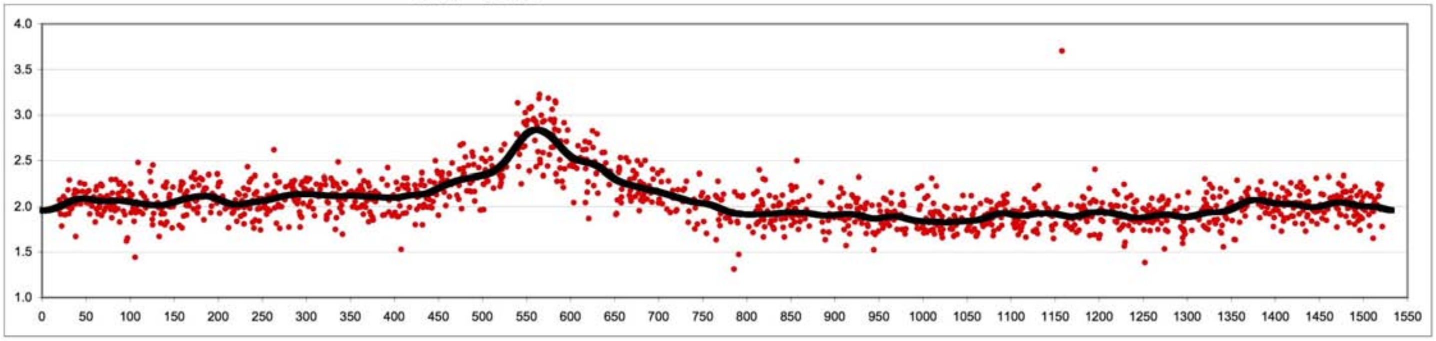

YJL8836 – Linker L9+L13

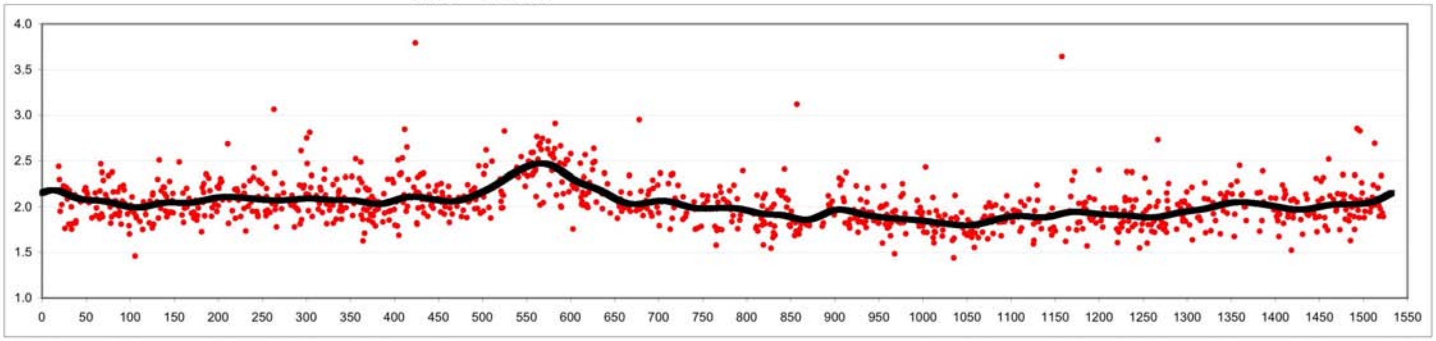

YJL8836 – Linker L9+L13

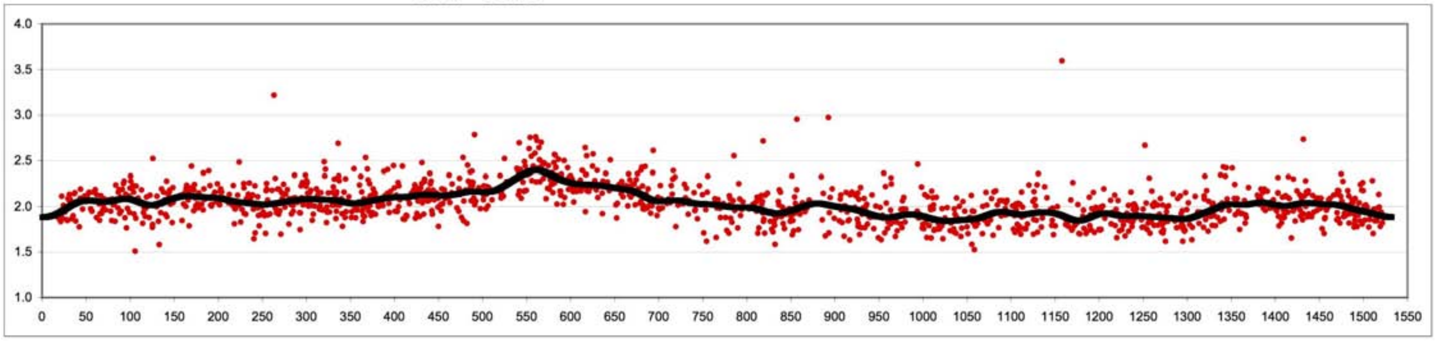

Supplemental Figure 4B
YJL8838

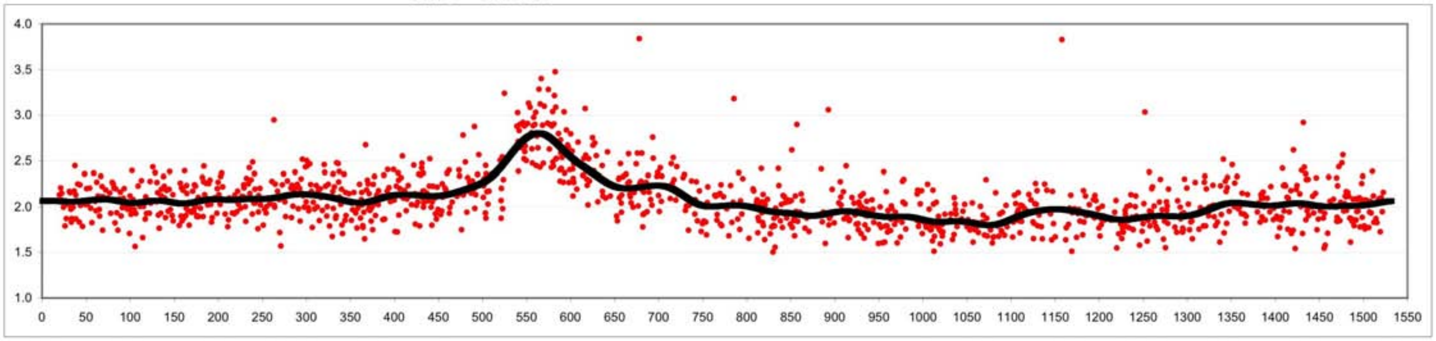

YJL8838

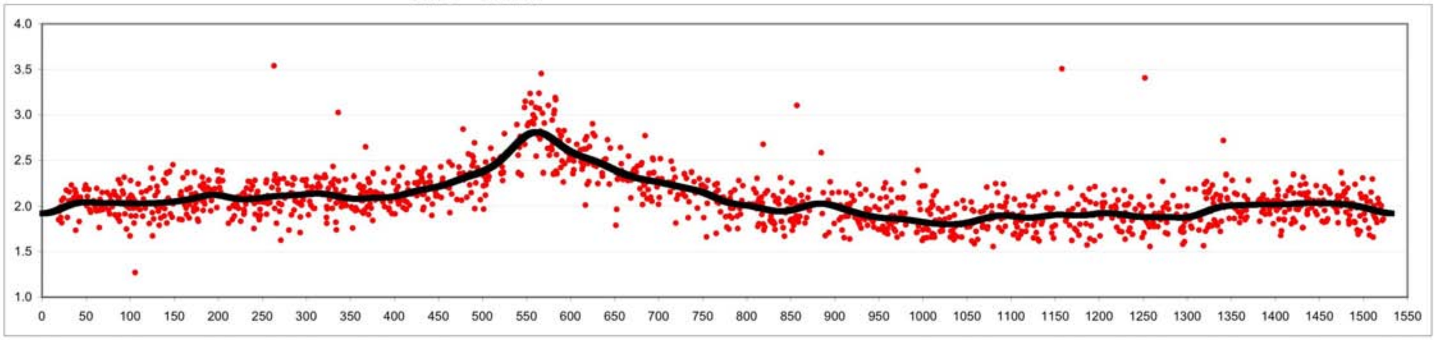

Supplemental Figure 4C
YJL9566 – ARS1238 control

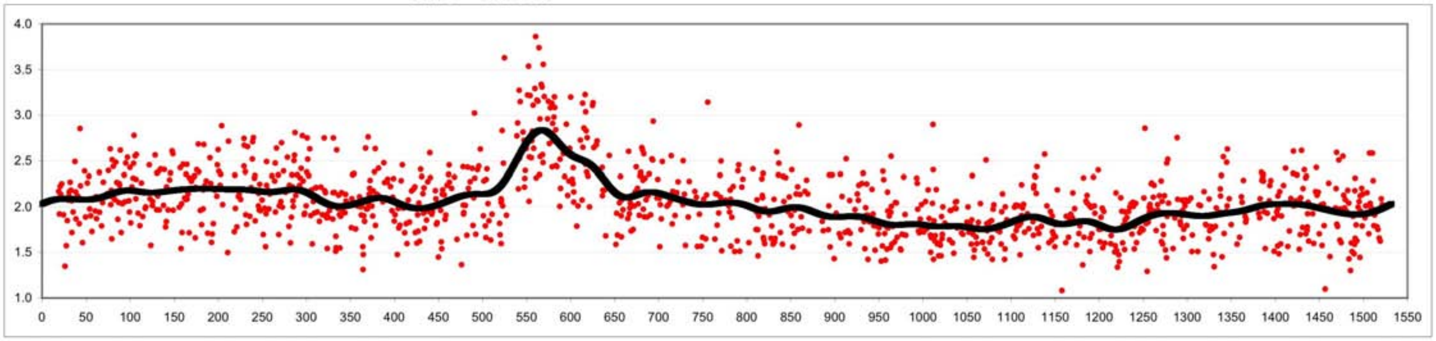

YJL9567 – ARS1238 control

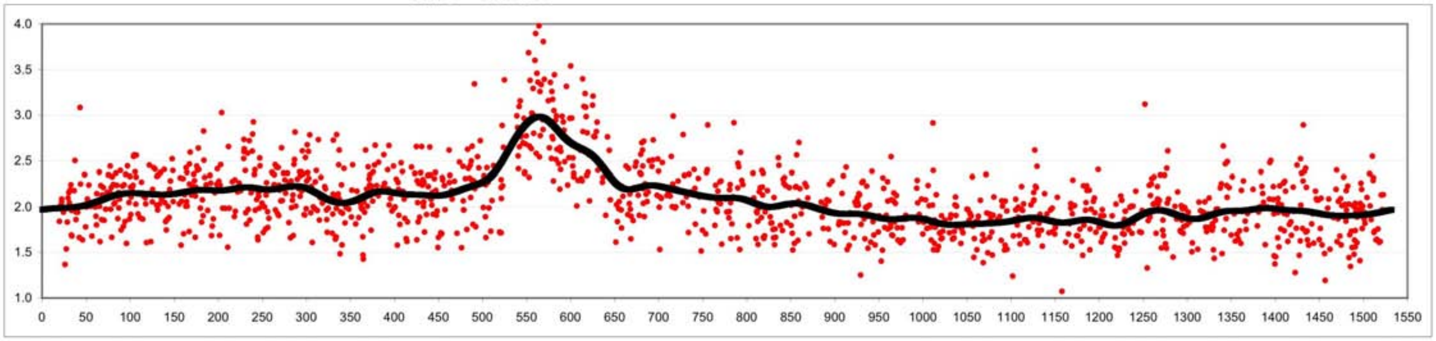

YJL10271 – ARS1238 Linker A

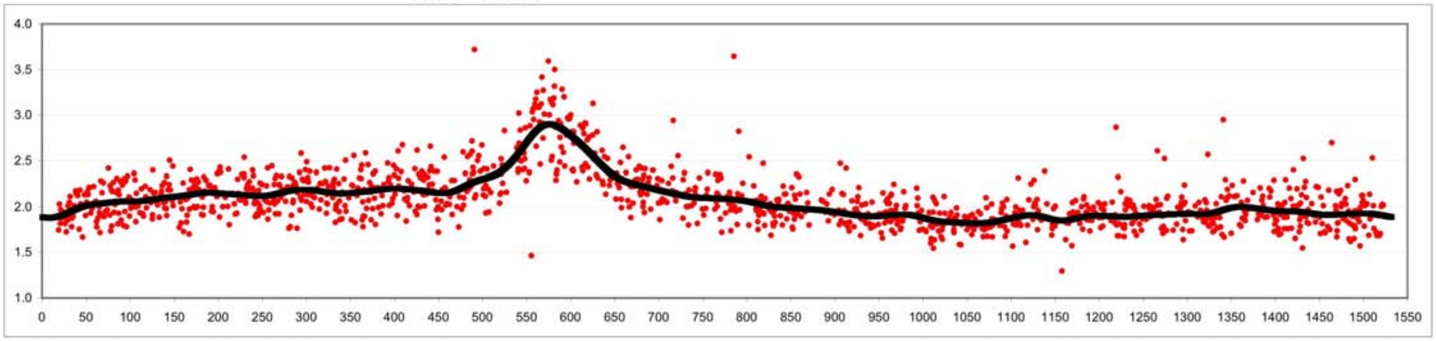

YJL10272 – ARS1238 Linker A

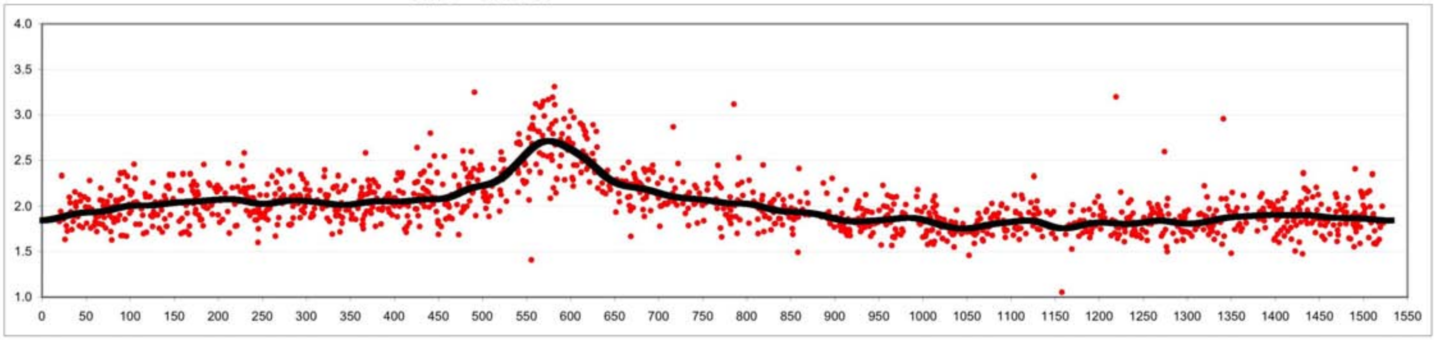

YJL10273 – ARS1238 Linker B

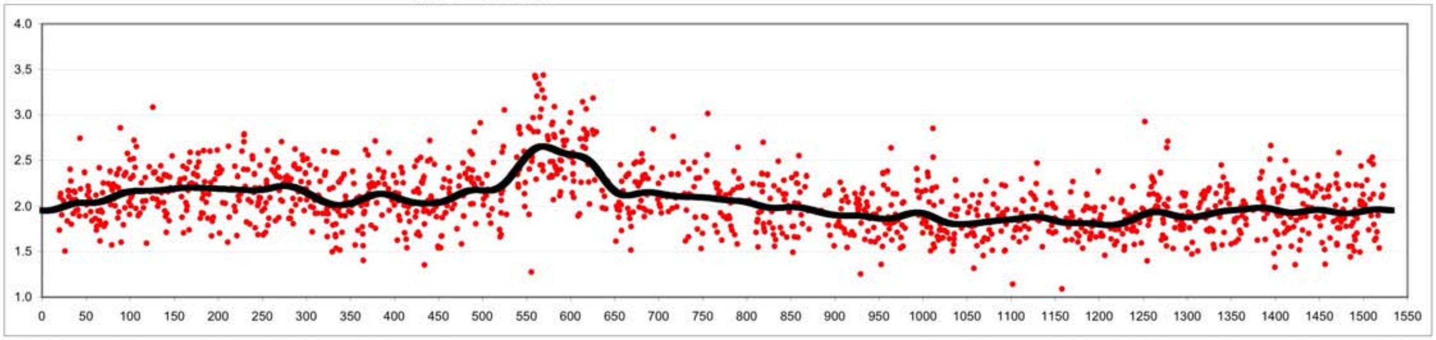

YJL10274 – ARS1238 Linker B

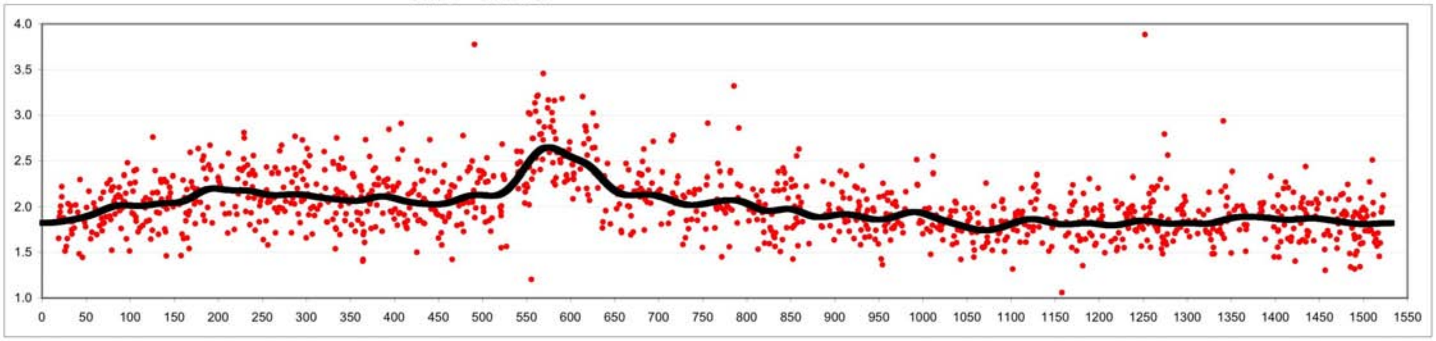

YJL10275 – ARS1238 Linker C

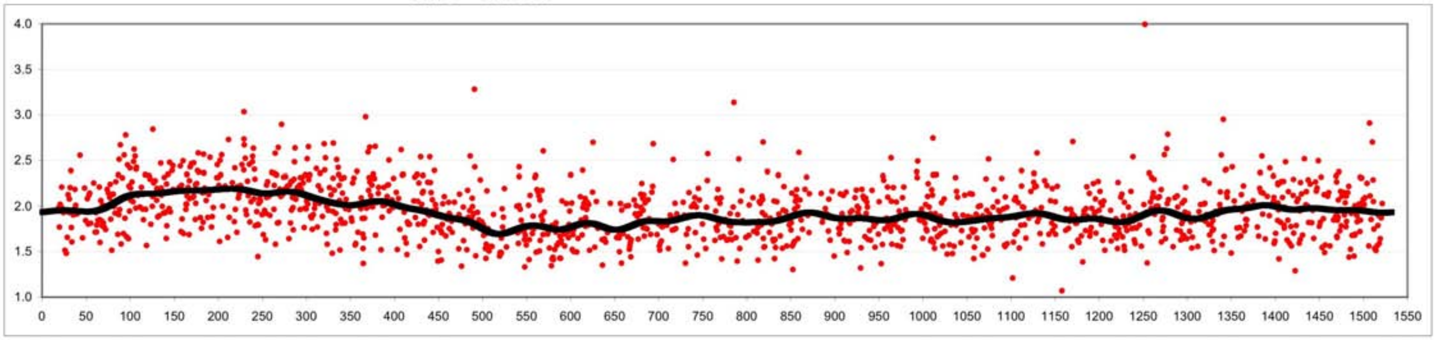

YJL10276 – ARS1238 Linker C

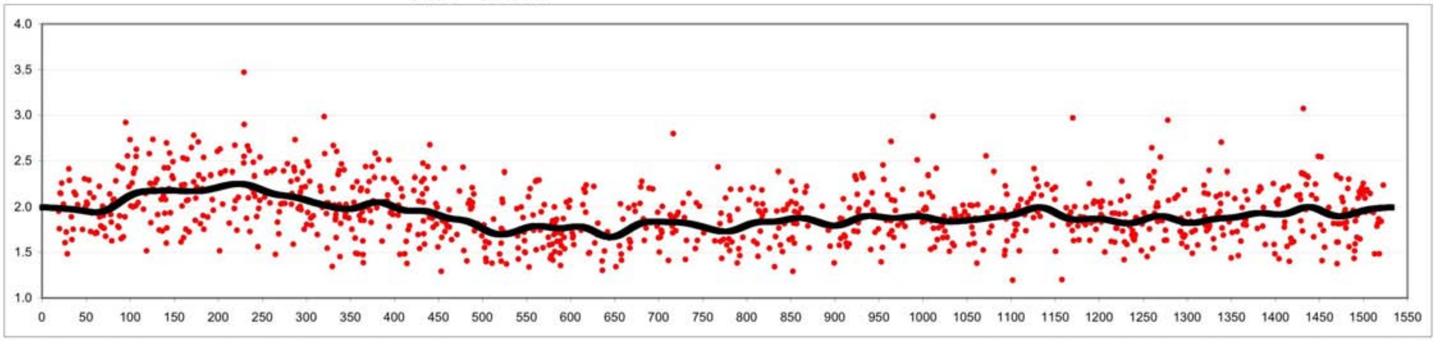

YJL10277 – ARS1238 Linker D

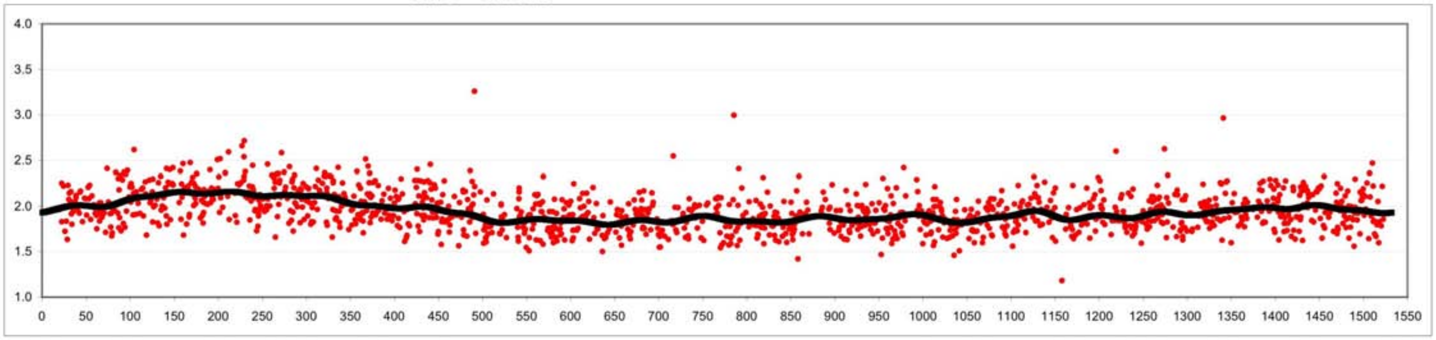

YJL10278 – ARS1238 Linker D

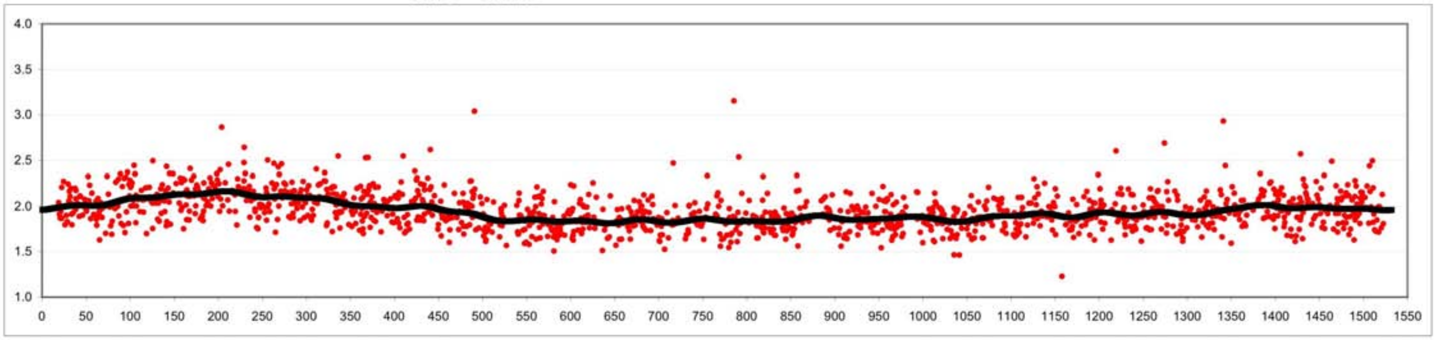

YJL10279 – ARS1238 Linker E

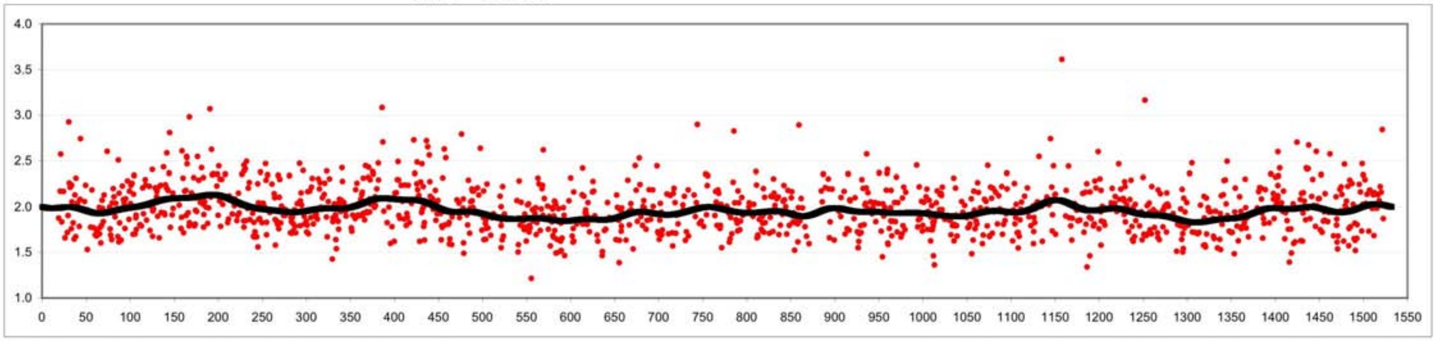

YJL10280 – ARS1238 Linker E

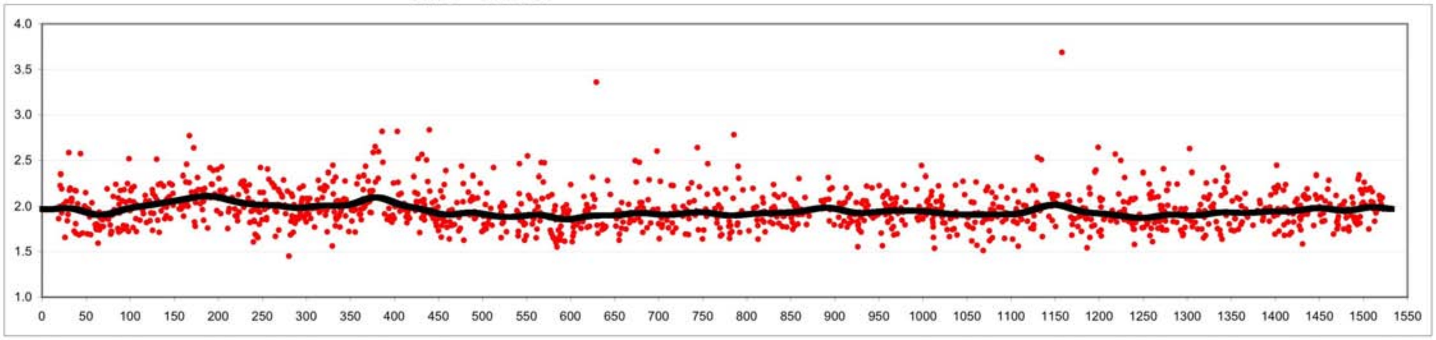

YJL10281 – ARS1238 Linker F

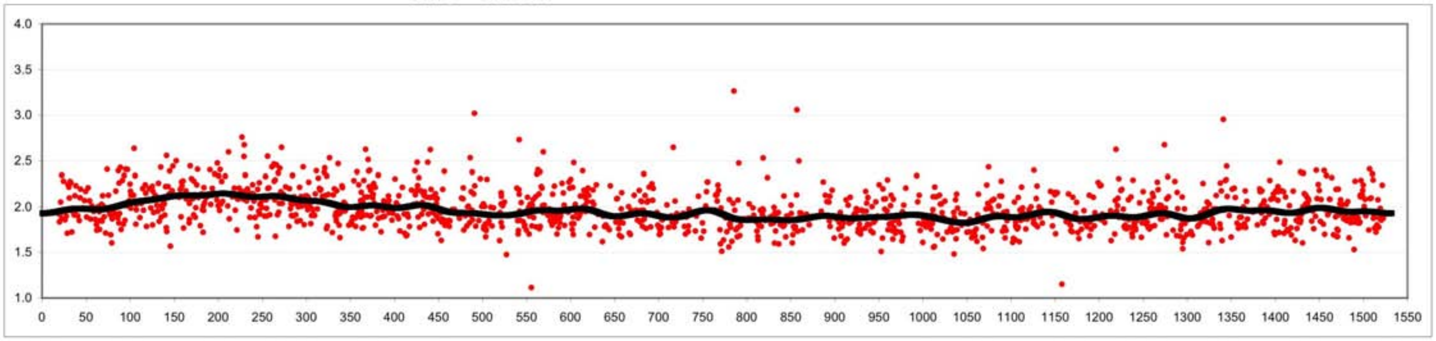

YJL10282 – ARS1238 Linker F

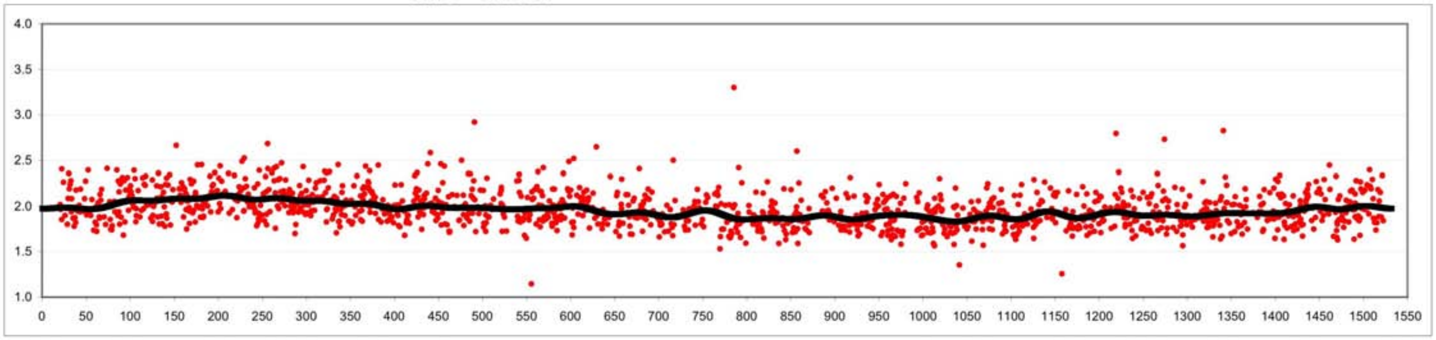

YJL10283 – ARS1238 Linker G

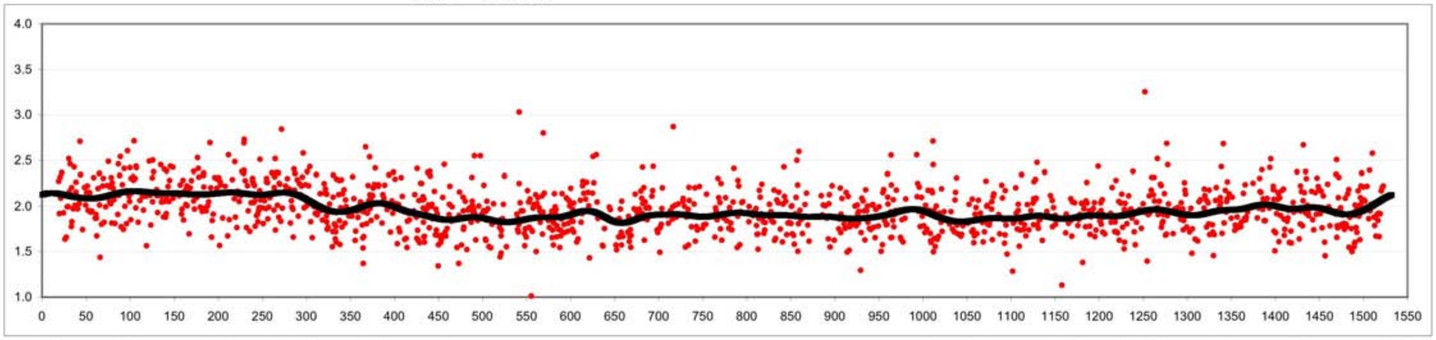

YJL10284 – ARS1238 Linker G

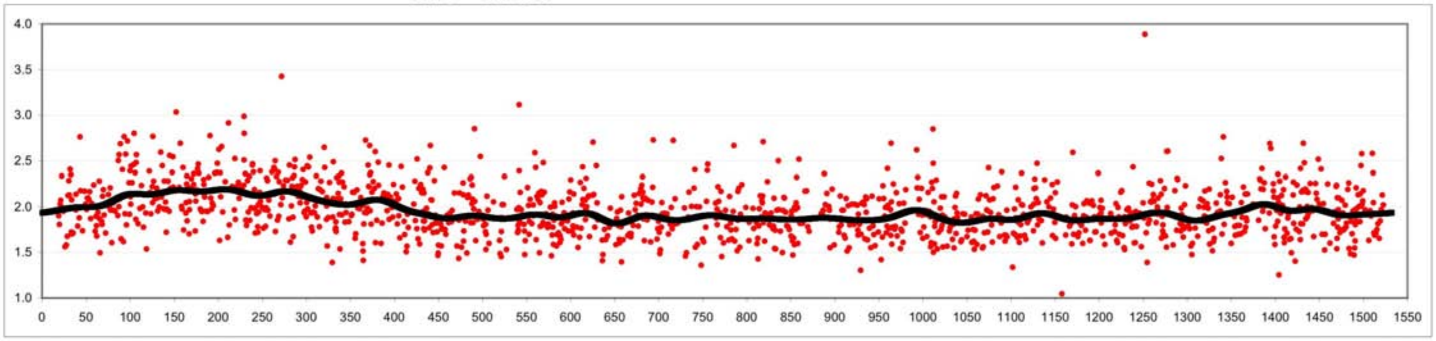

YJL10285 – ARS1238 Linker H

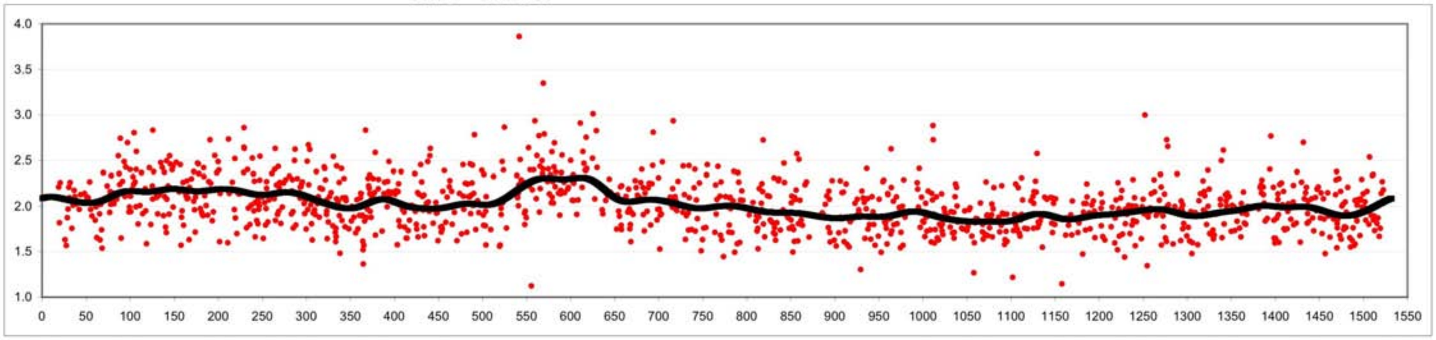

YJL10286 – ARS1238 Linker H

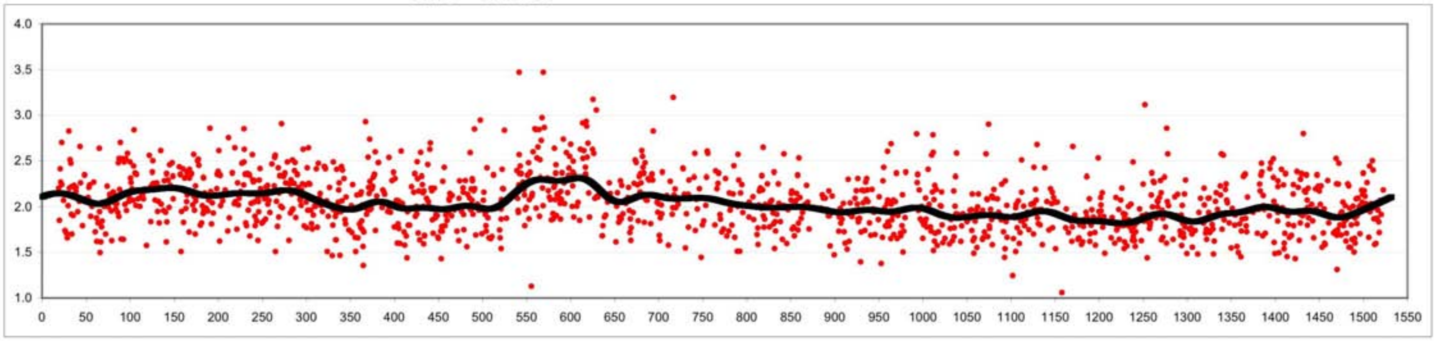

Supplemental Figure 5
YJL8398 – control

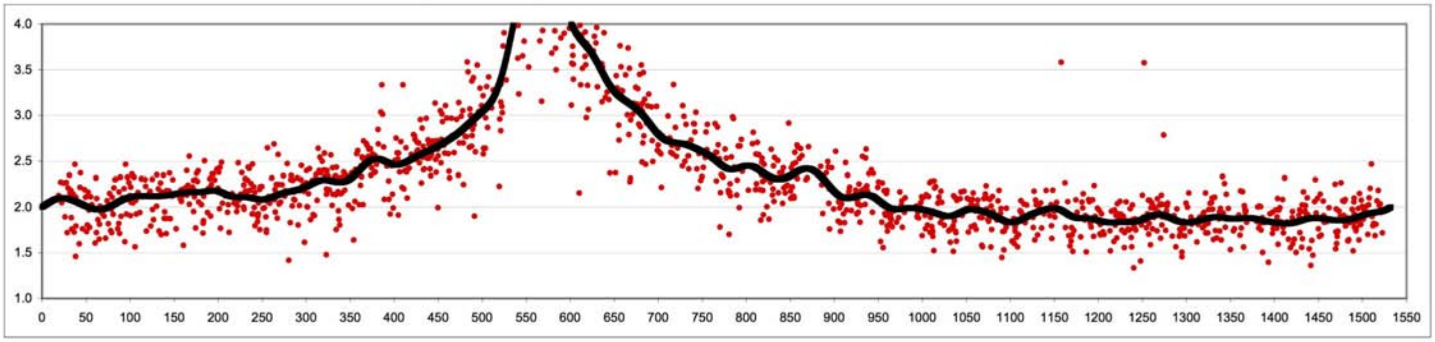

YJL8399 – control

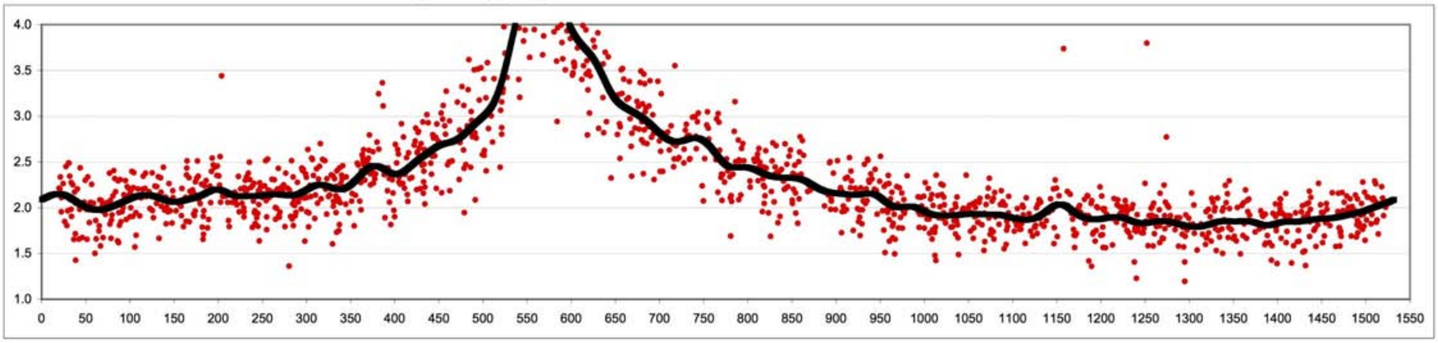

YJL10319 – C-D Reverse

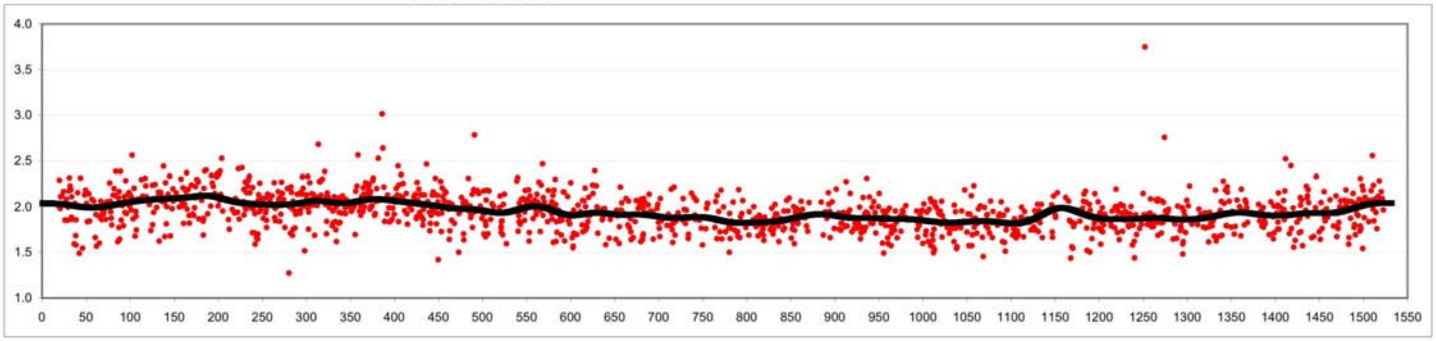

YJL10320 – C-D Reverse

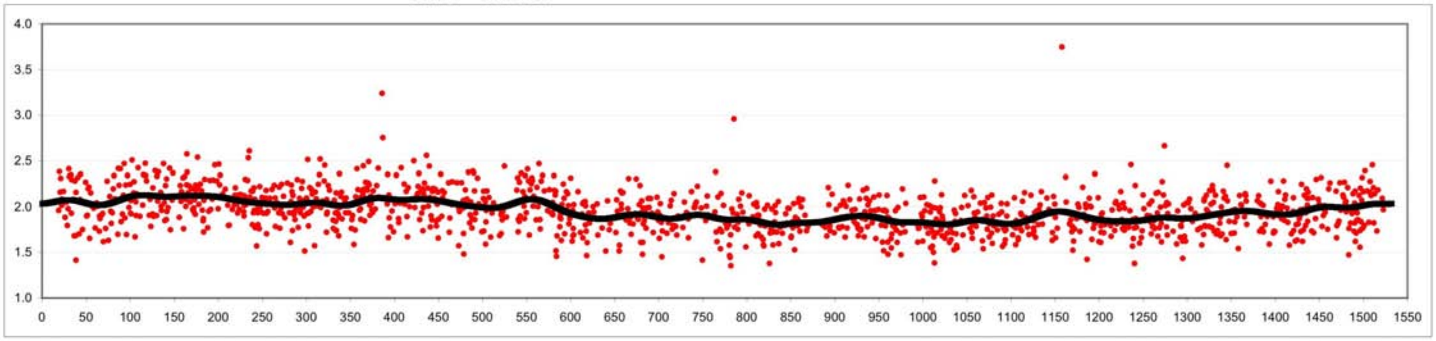

YJL10321 – C-D Transversion

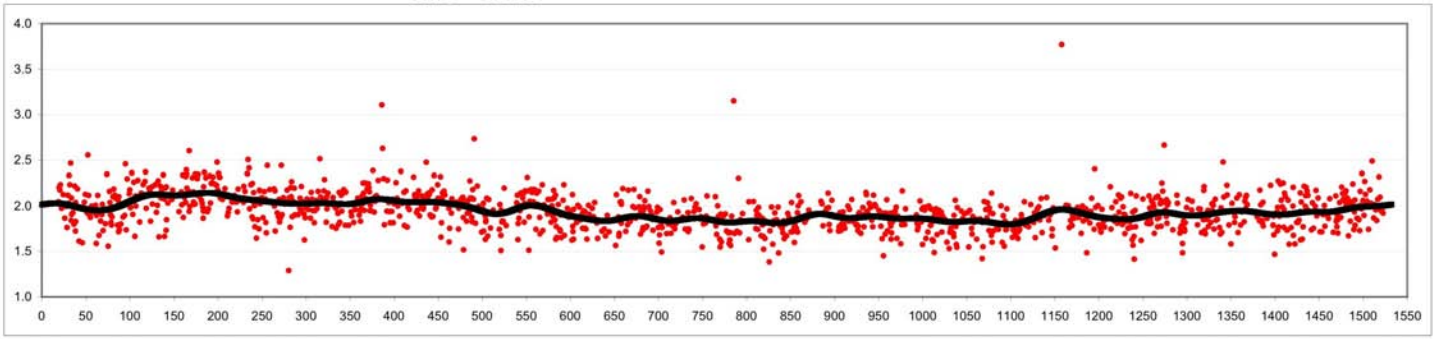

YJL10322 - C-D Transversion

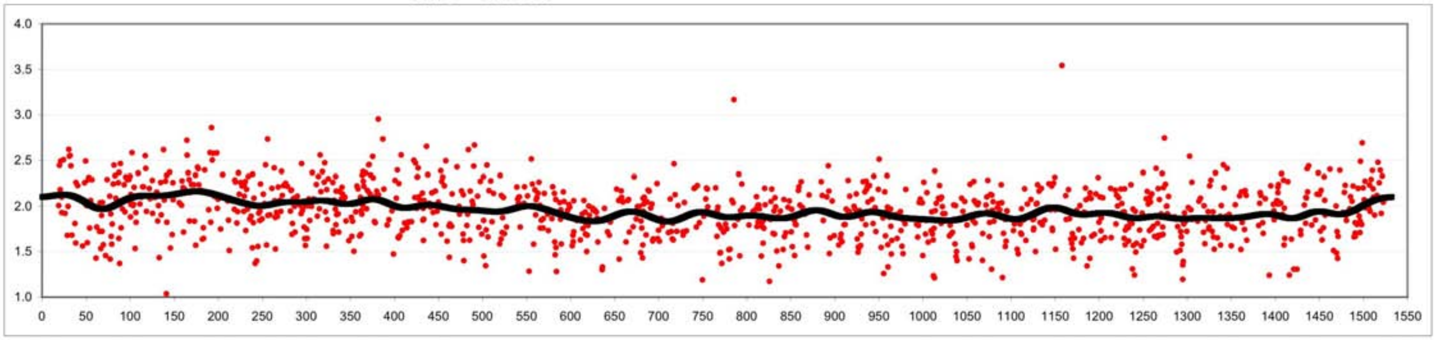

YJL10323 - C-D Scramble

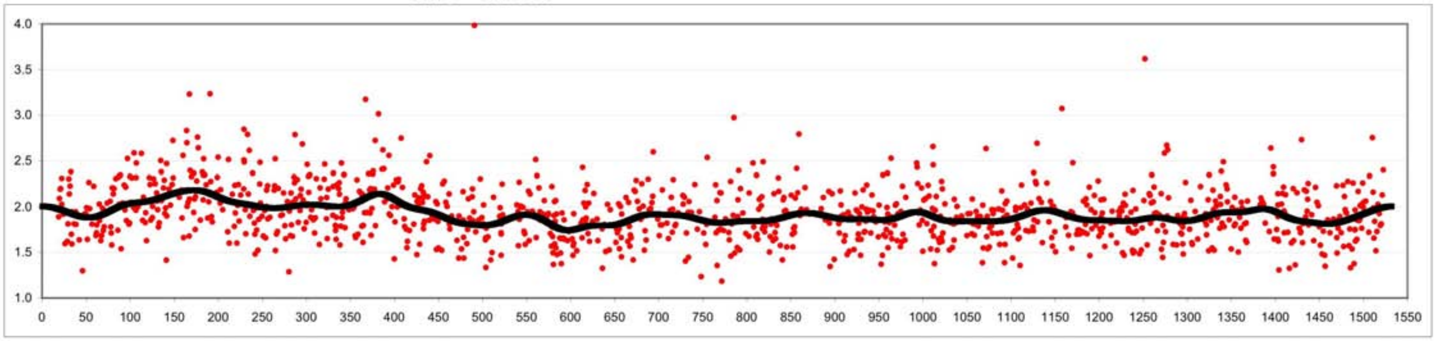

YJL10324 - C-D Scramble

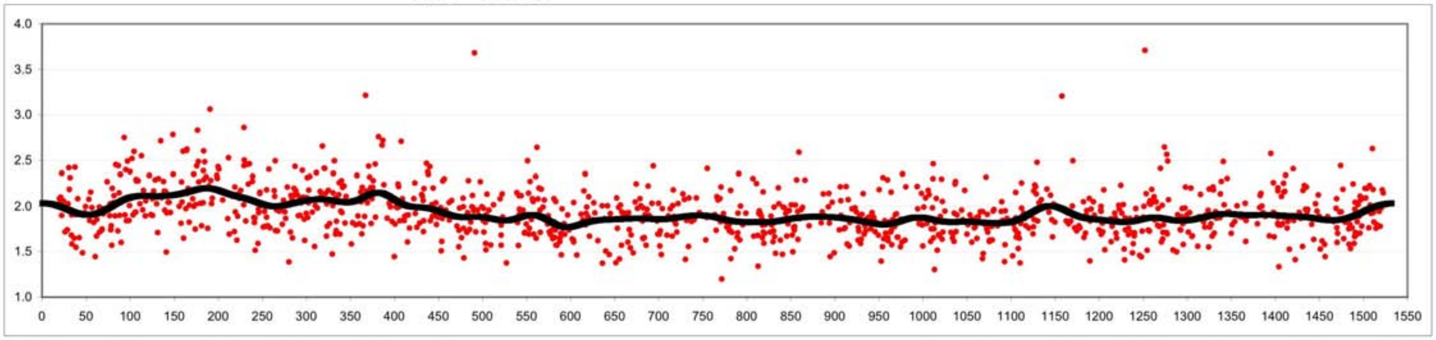

YJL10325 – C Transversion

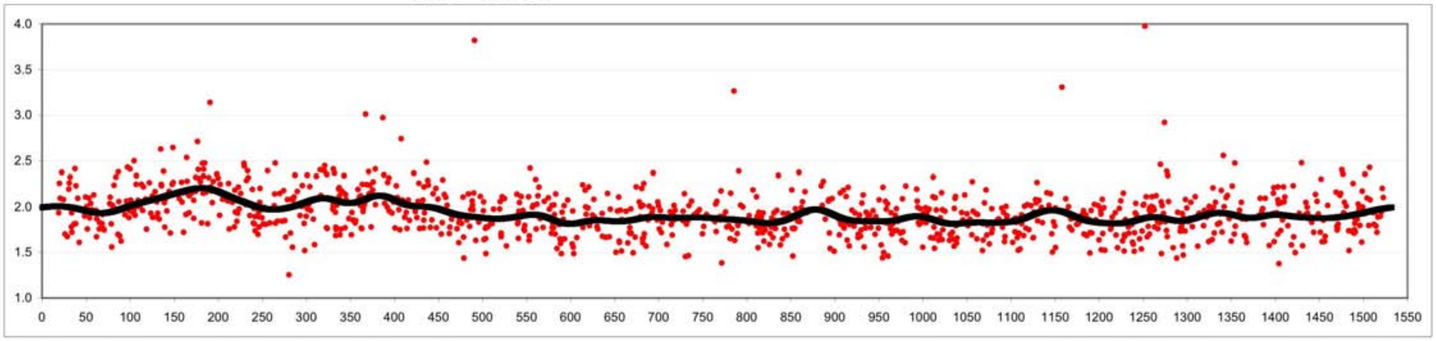

YJL10326 - C Transversion

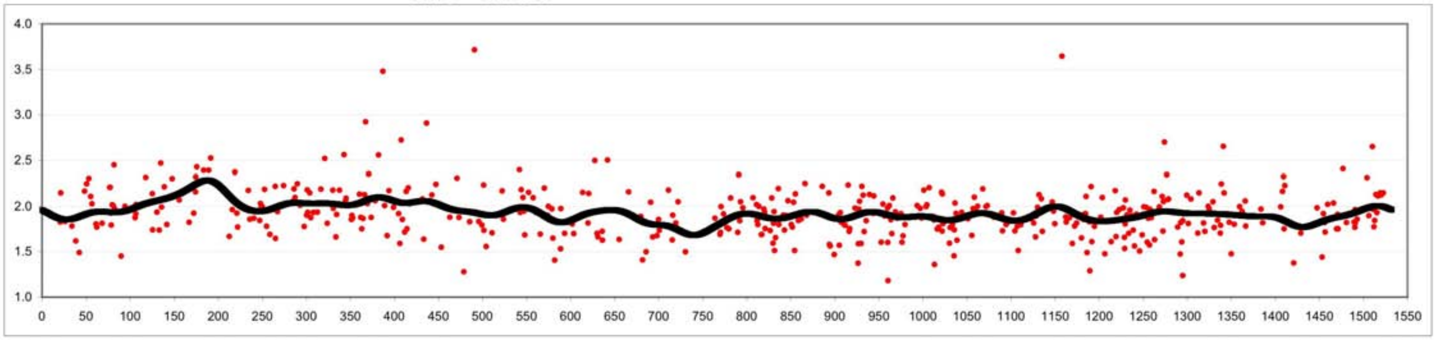

YJL10327 - D Transversion

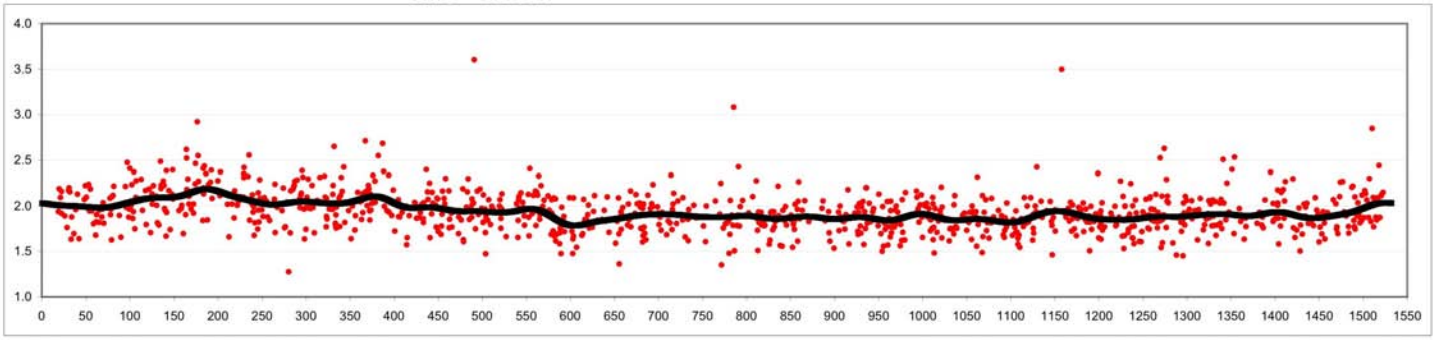

YJL10328 - D Transversion

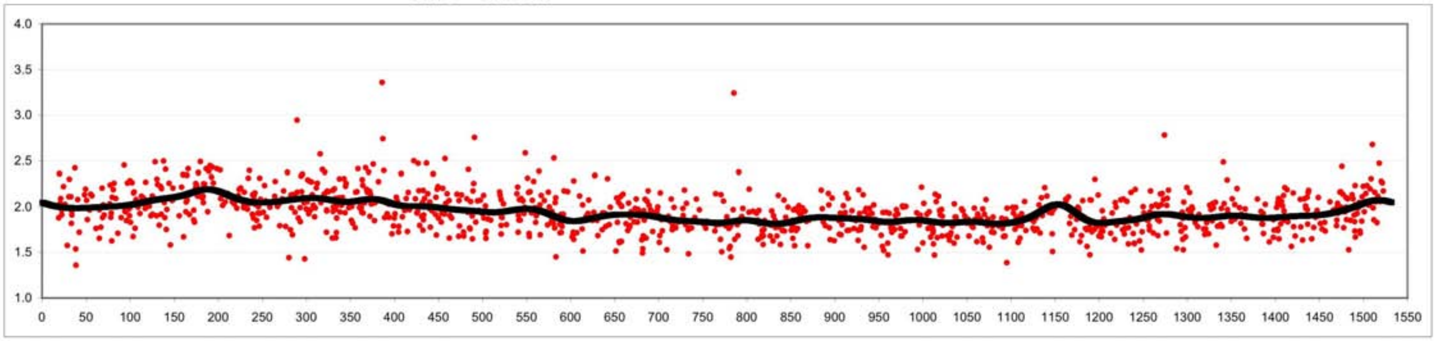

YJL10329 – C1 Transversion

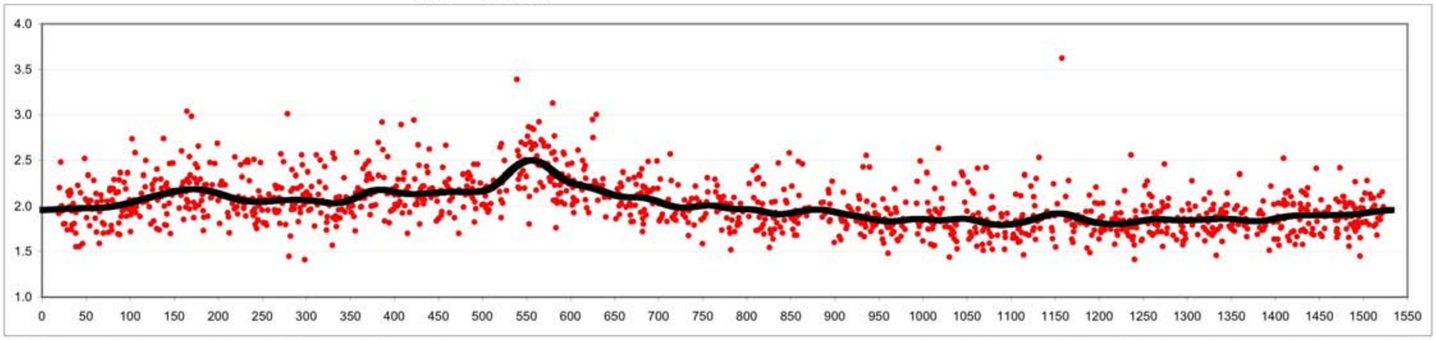

YJL10330 – C1 Transversion

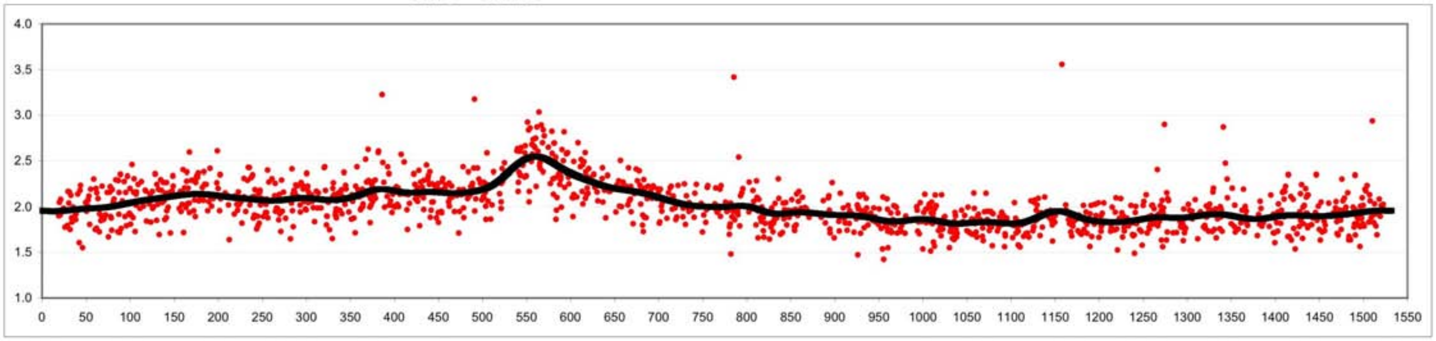

YJL10331 – C2 Transversion

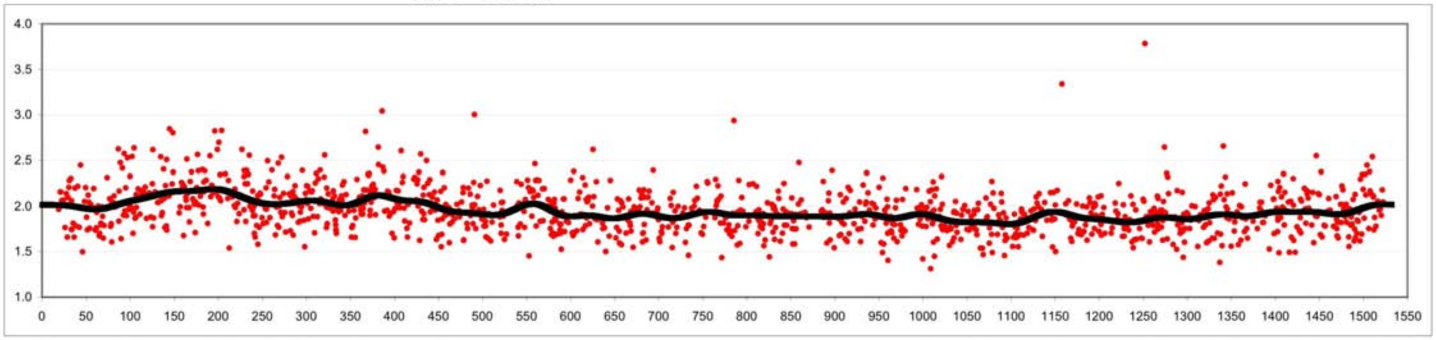

YJL10332 – C2 Transversion

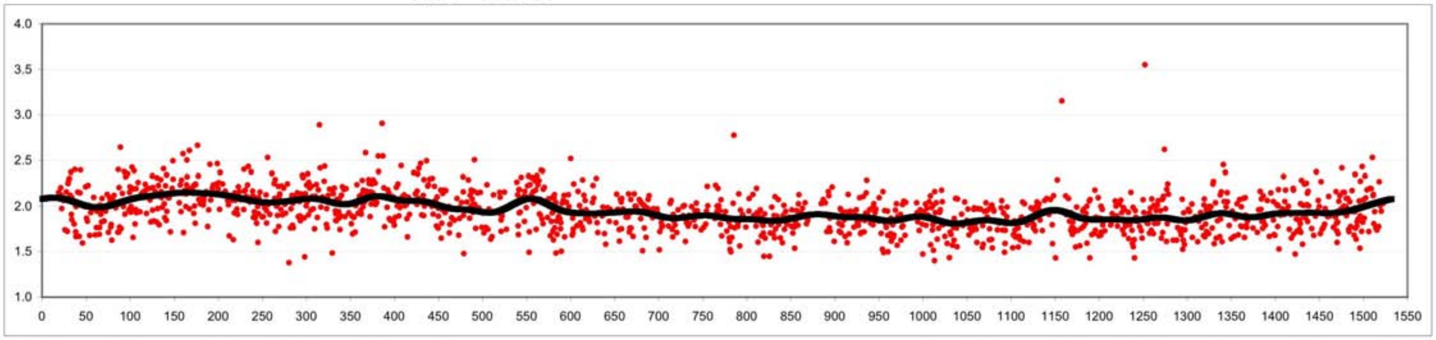

YJL10333 – D1 Transversion

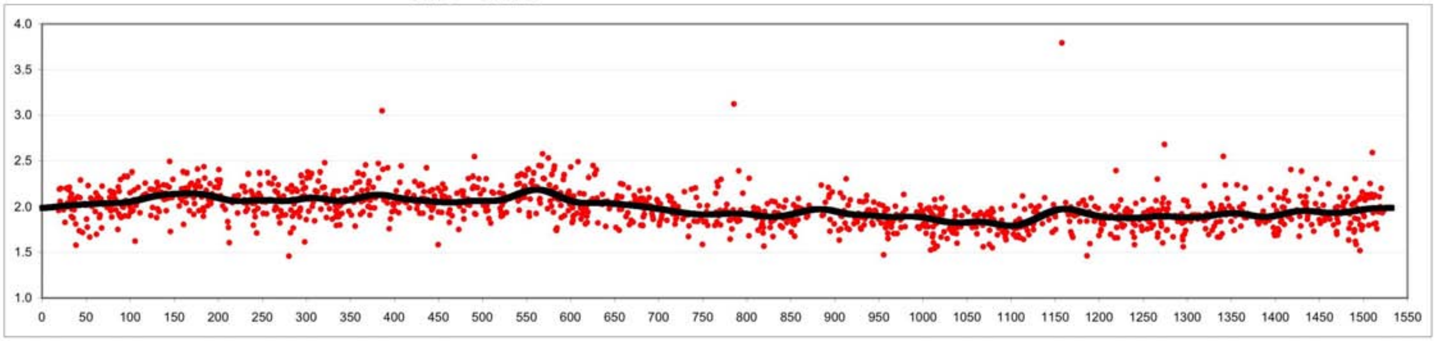

YJL10334 - D1 Transversion

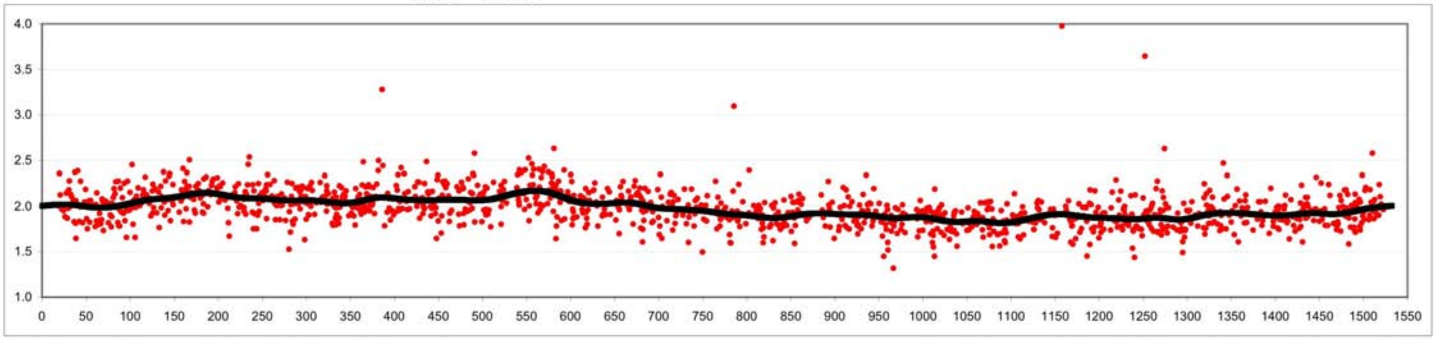

YJL10335 – D2 Transversion

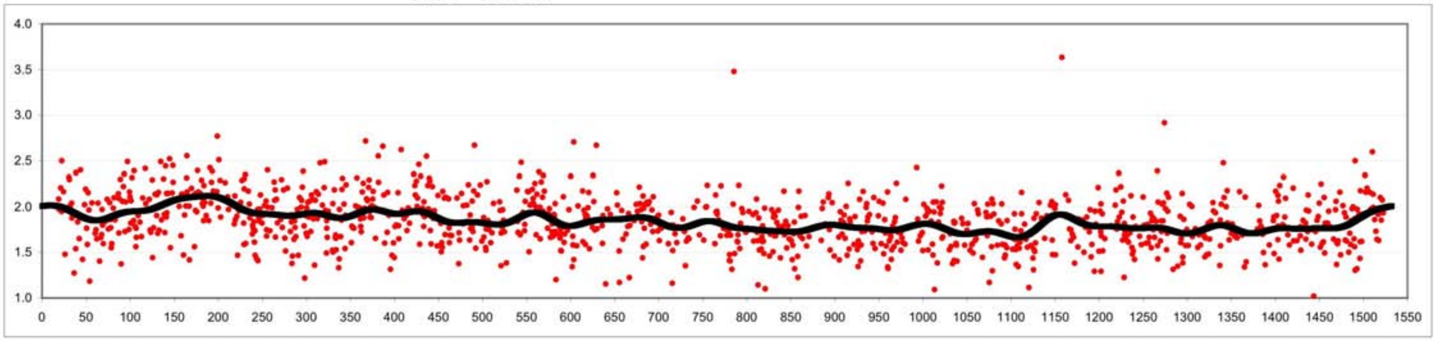

YJL10336 – D2 Transversion

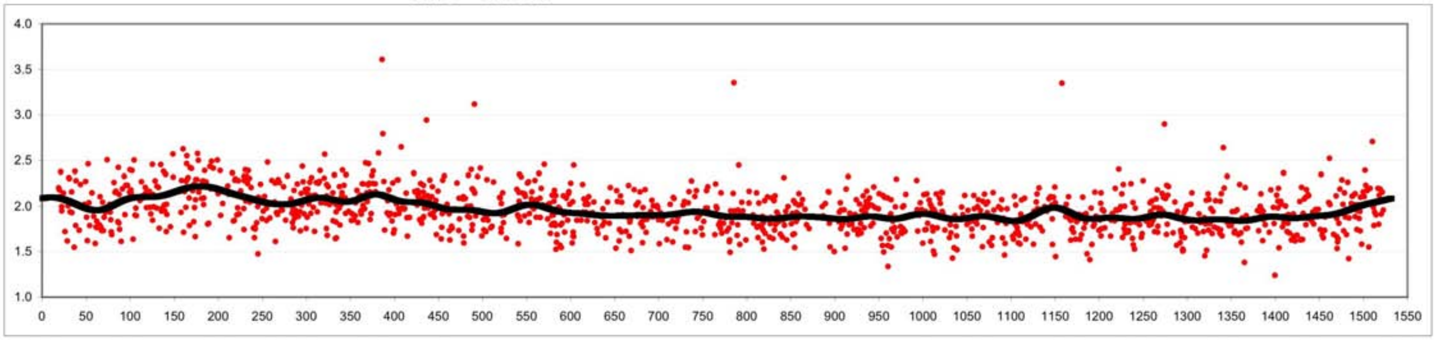

Supplemental Figure 6A

YJL10160

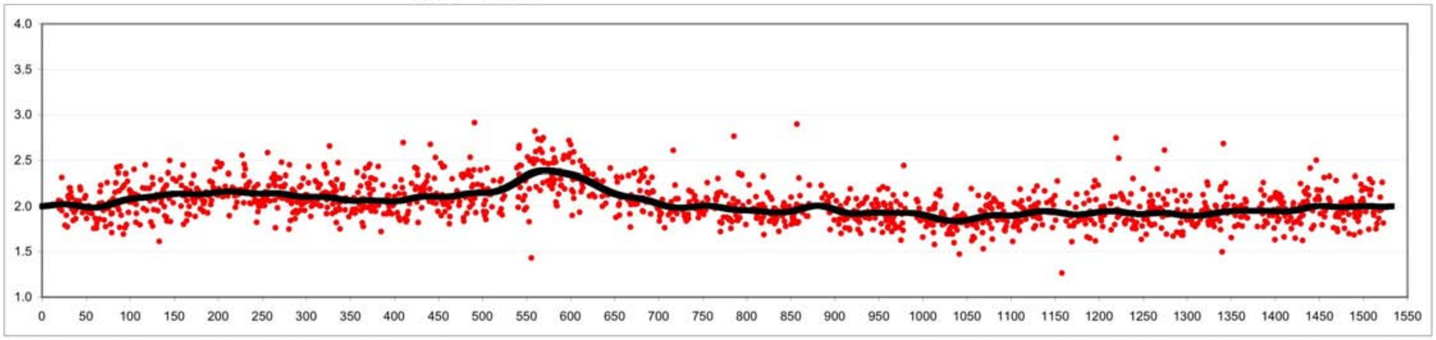

YJL10161

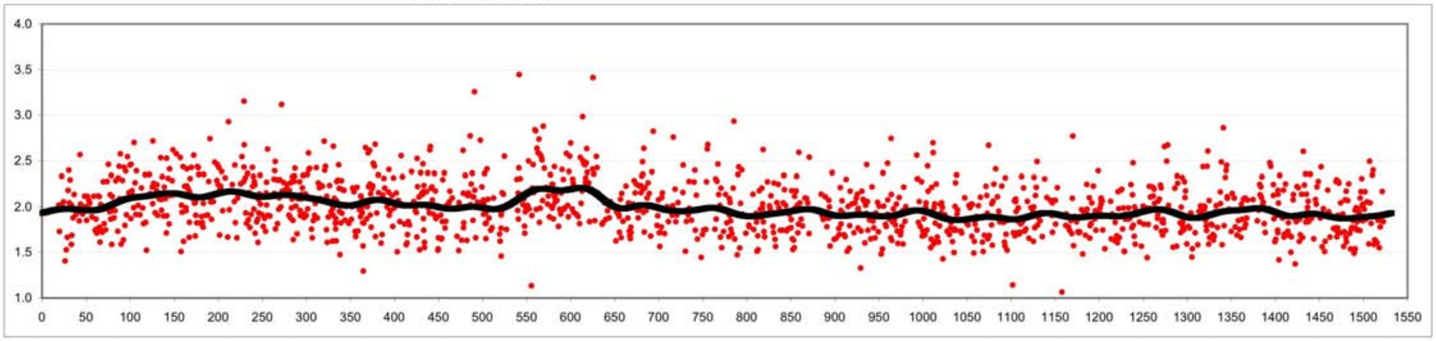

Supplemental Figure 6B

YJL9082 – 6 hour

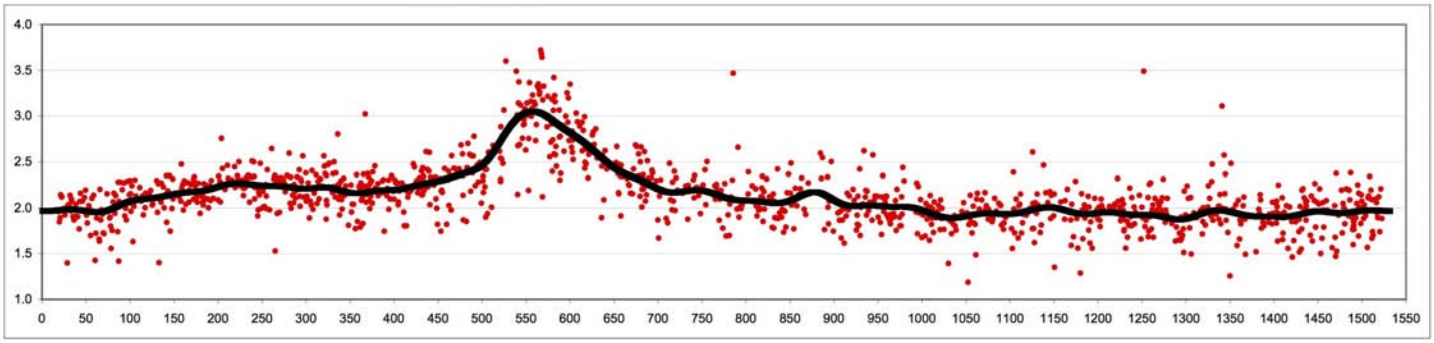

YJL9082 – 6 hour

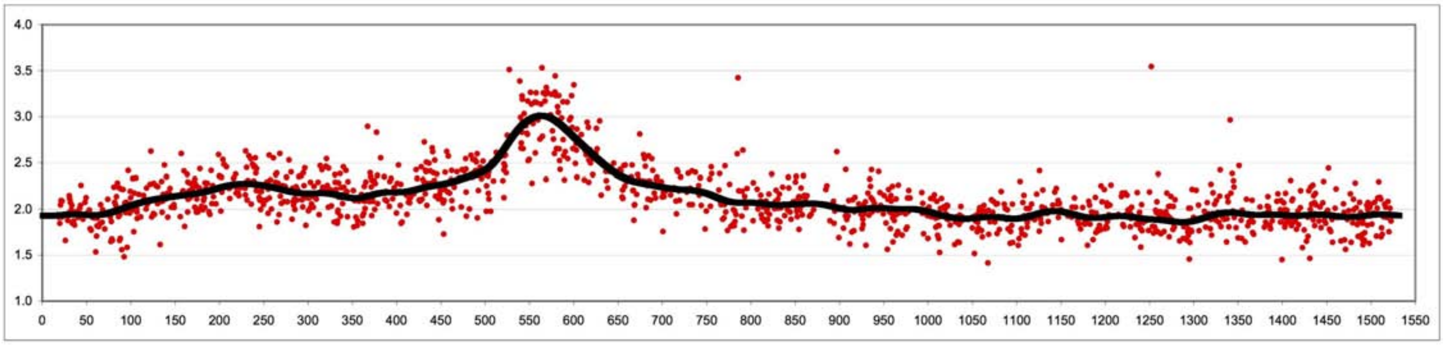

YJL9084

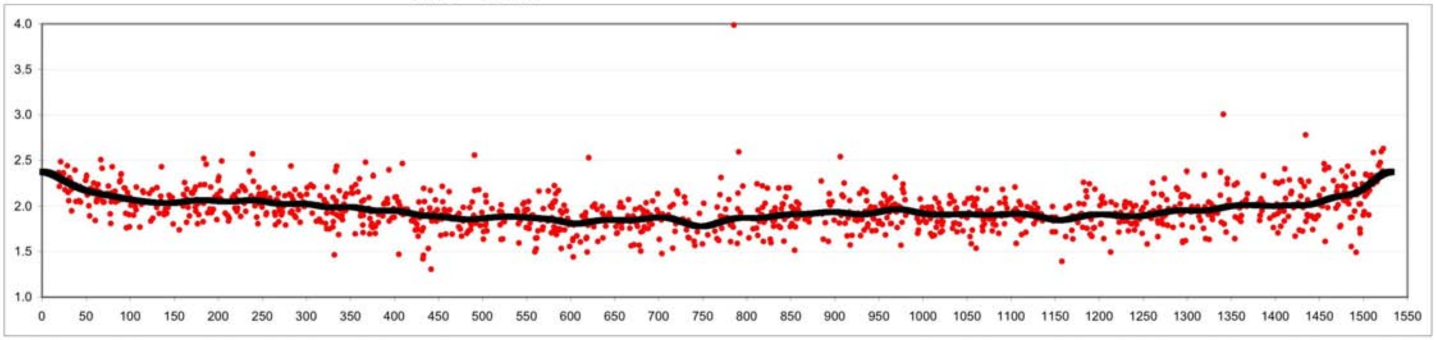

YJL9084

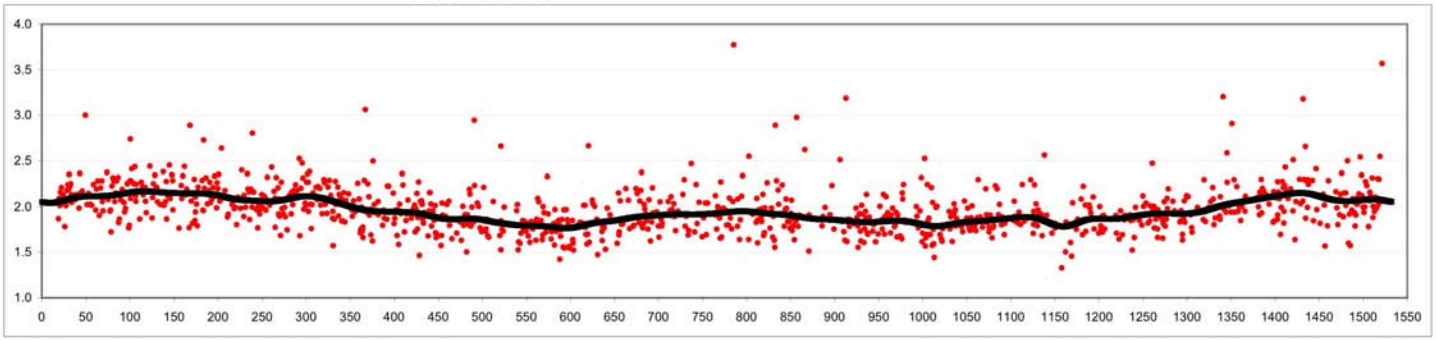

YJL9086

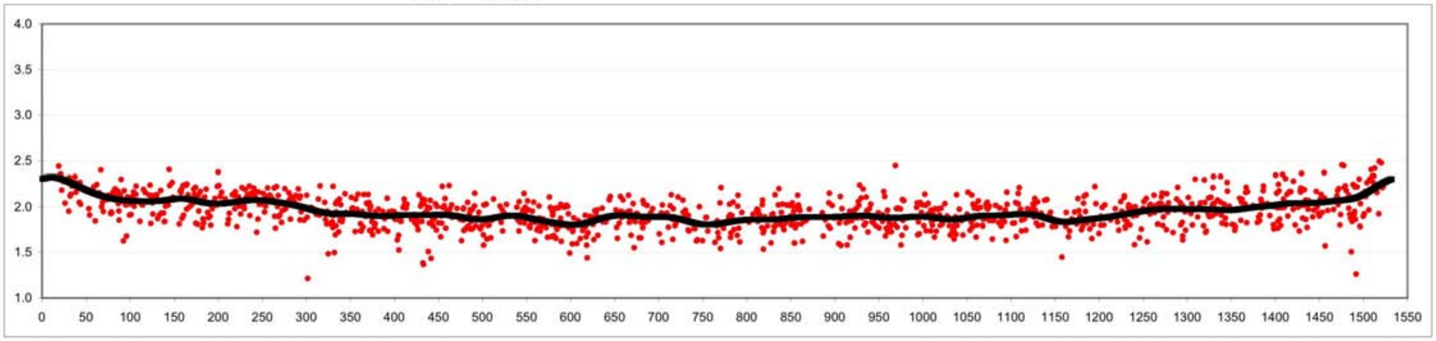

YJL9086

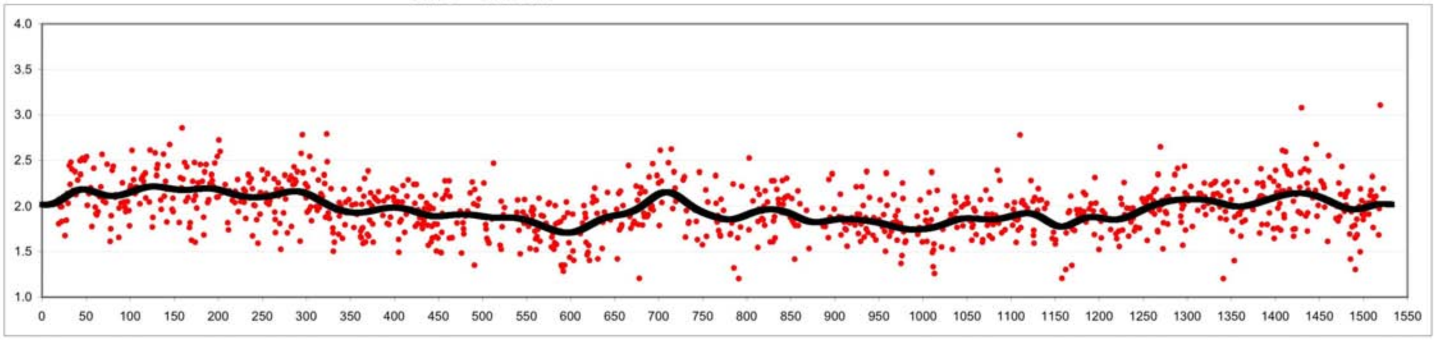

YJL9088 – 6 hour

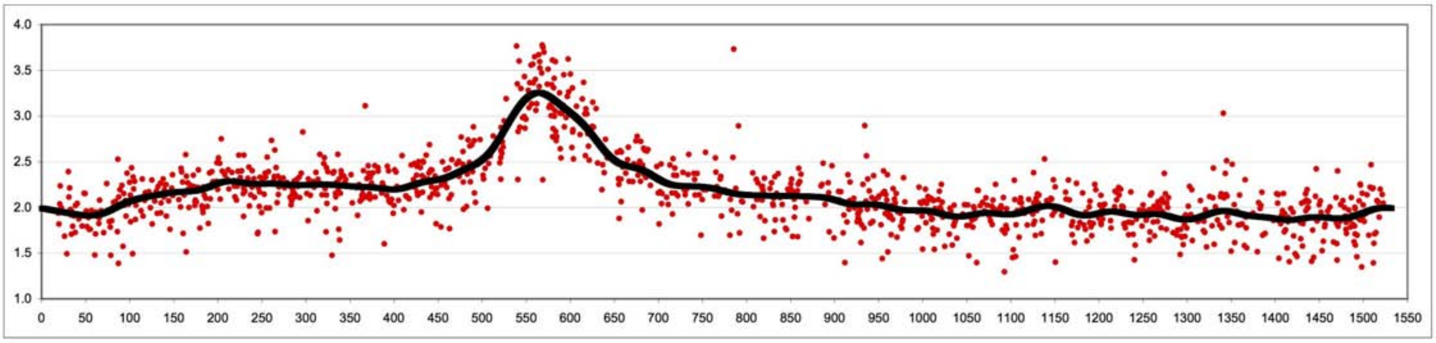

YJL9088 – 6 hour

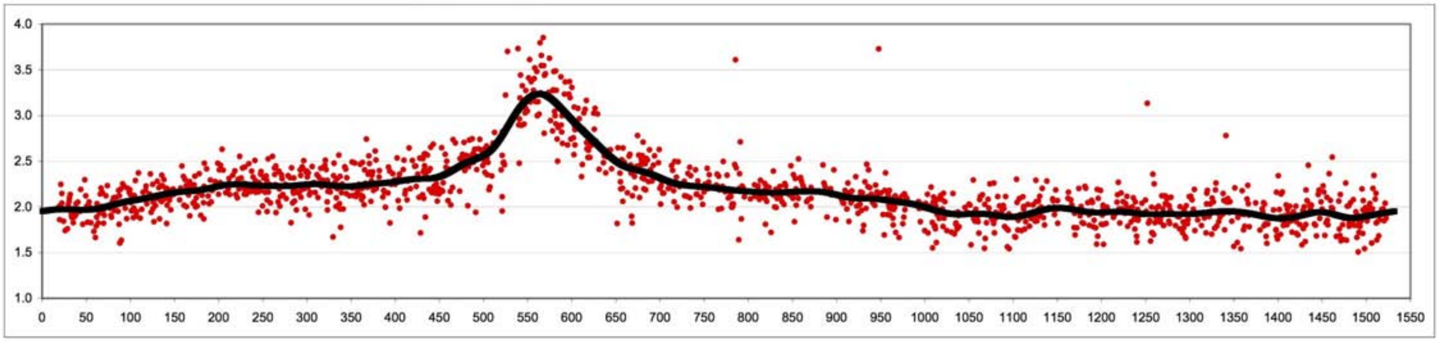

Supplement: Document S1 — Raw normalized data (red dots) and smoothed line (black line) used to generate composite profiles or percent re-replication efficiency for all the other figures of this manuscript. (PDF) [file pgen.1004358.s011.pdf]
